# Supplementary figures and images for: Coupling Reactions of Anhydro-Aldose Tosylhydrazones with Boronic Acids
Source: Molecules. 2022 Mar 9;27(6):1795. doi: 10.3390/molecules27061795 (PMC8953641; doi:10.3390/molecules27061795)

Supplementary Materials

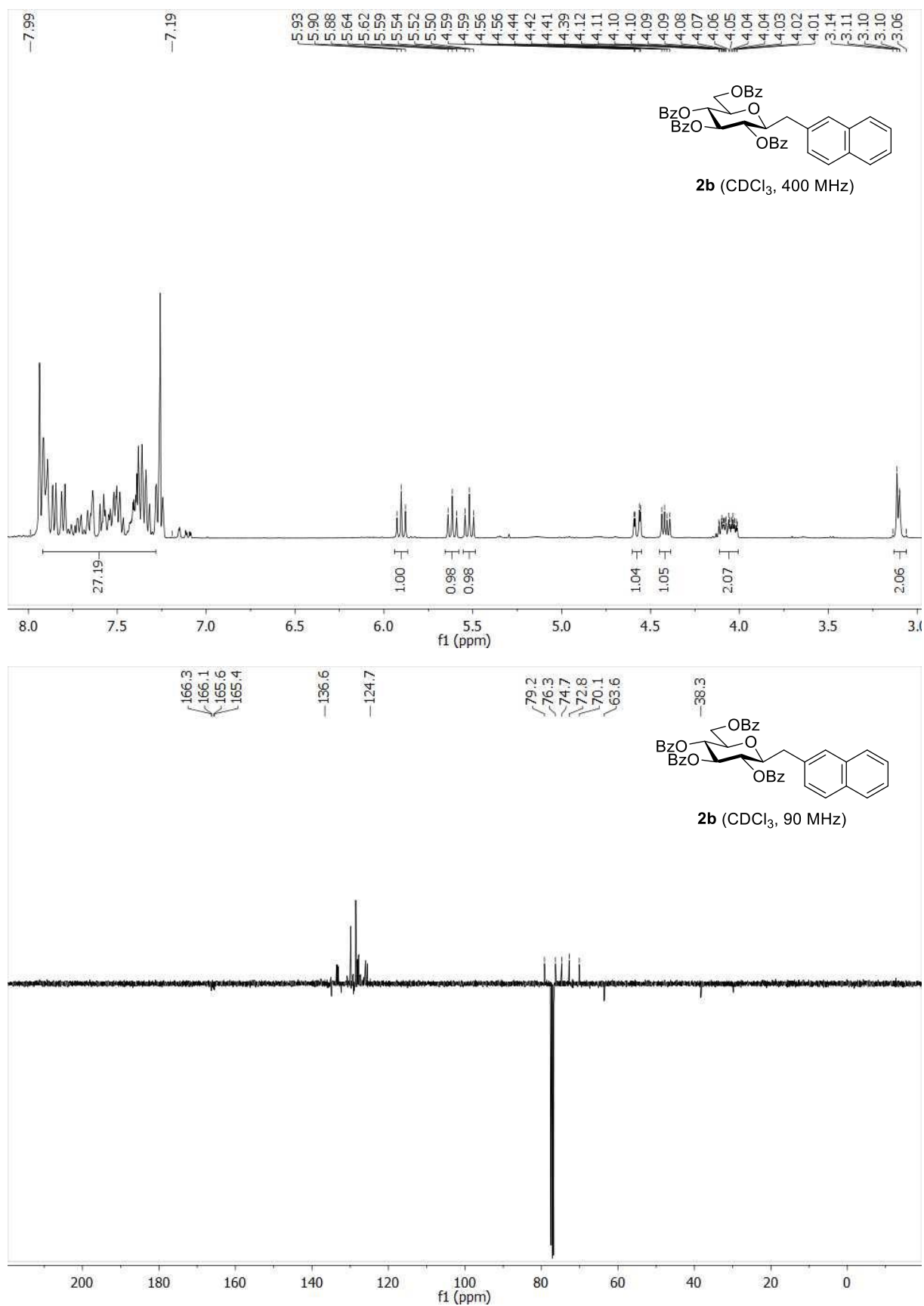

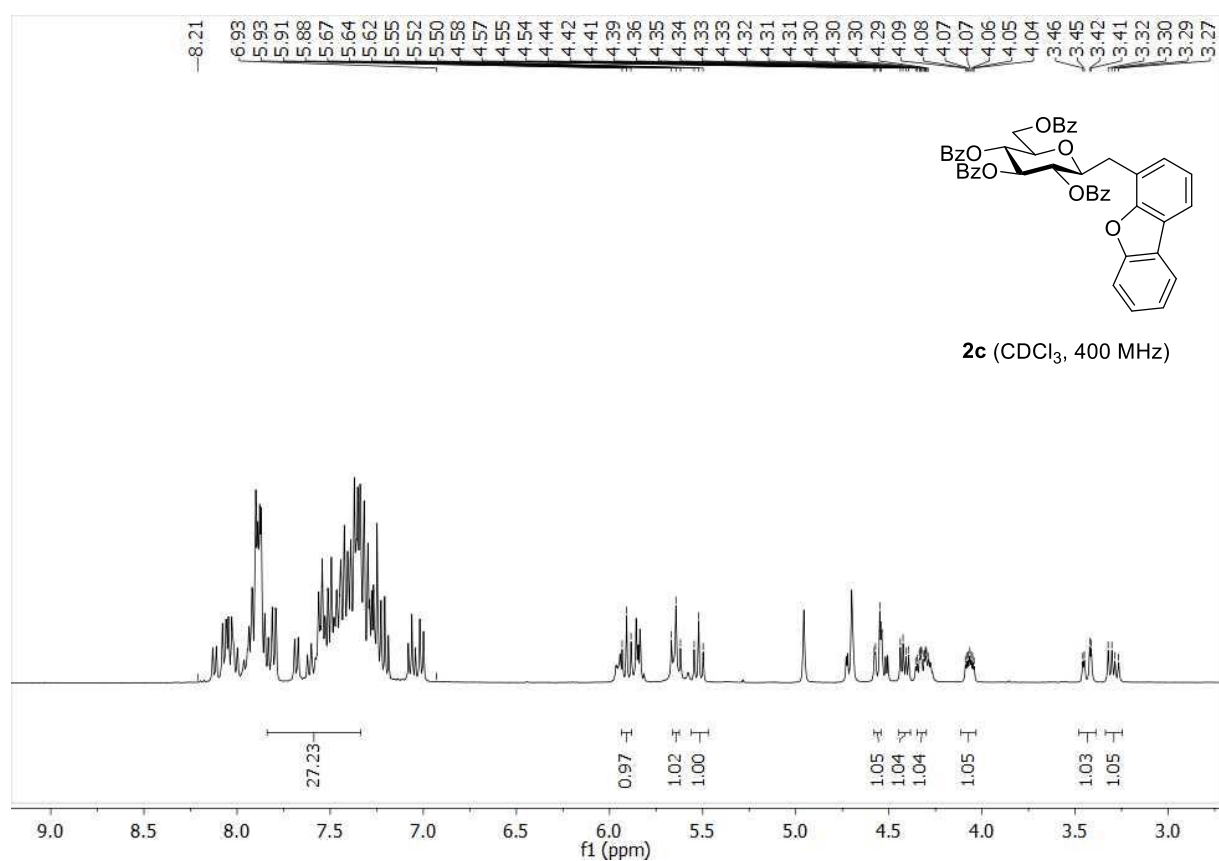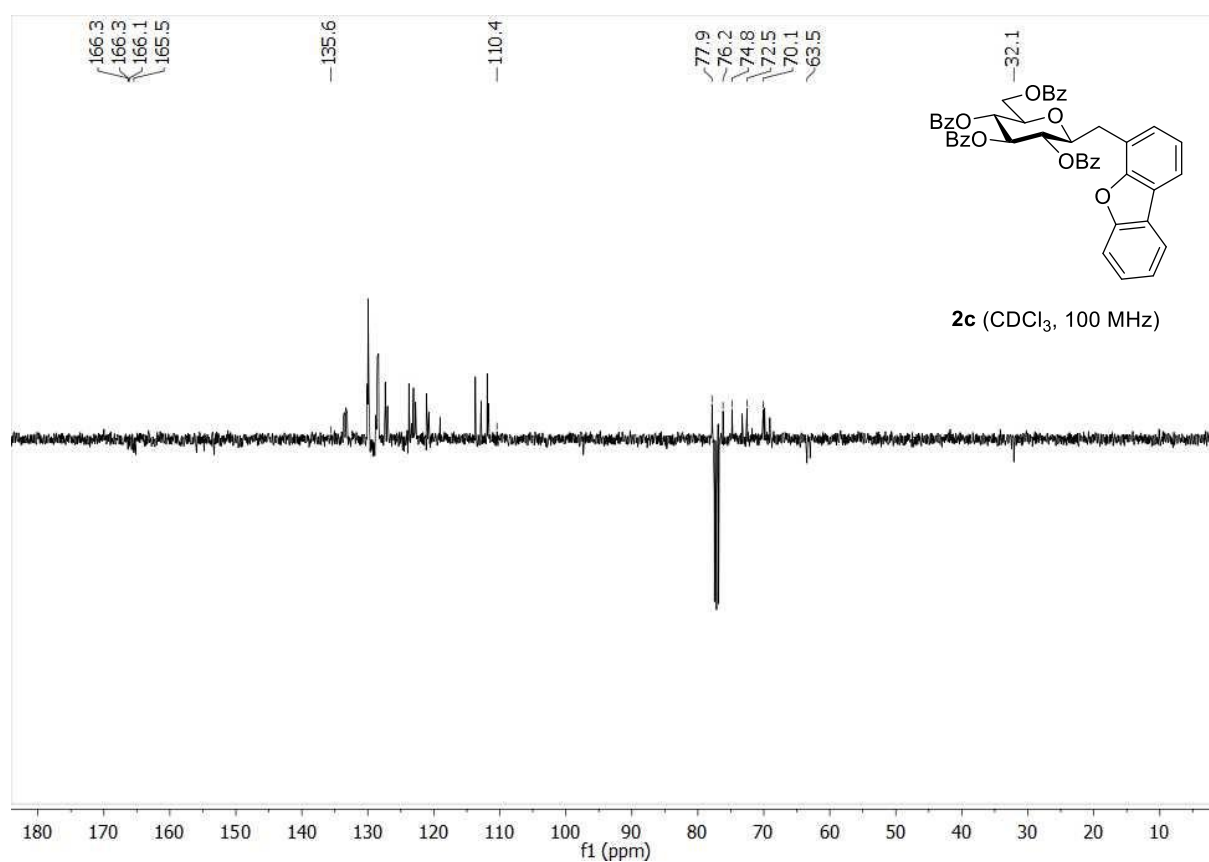

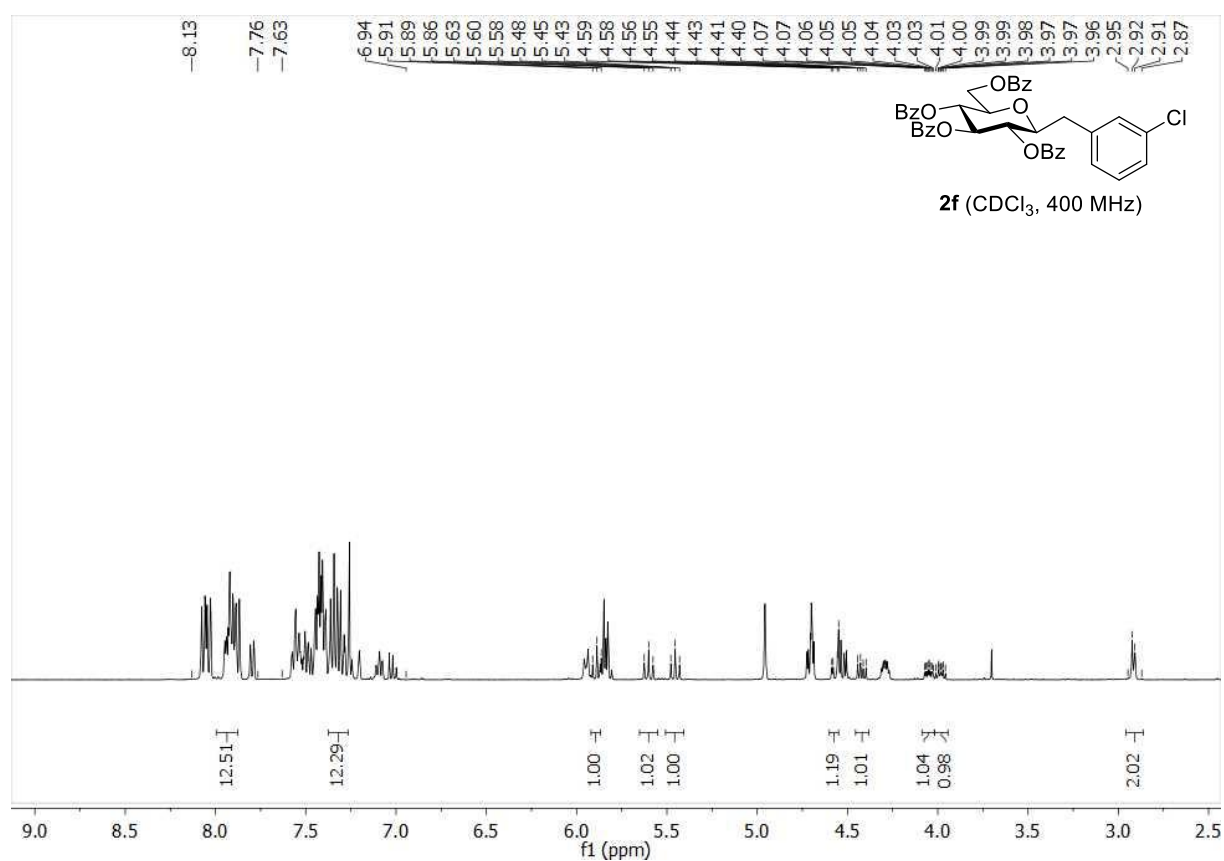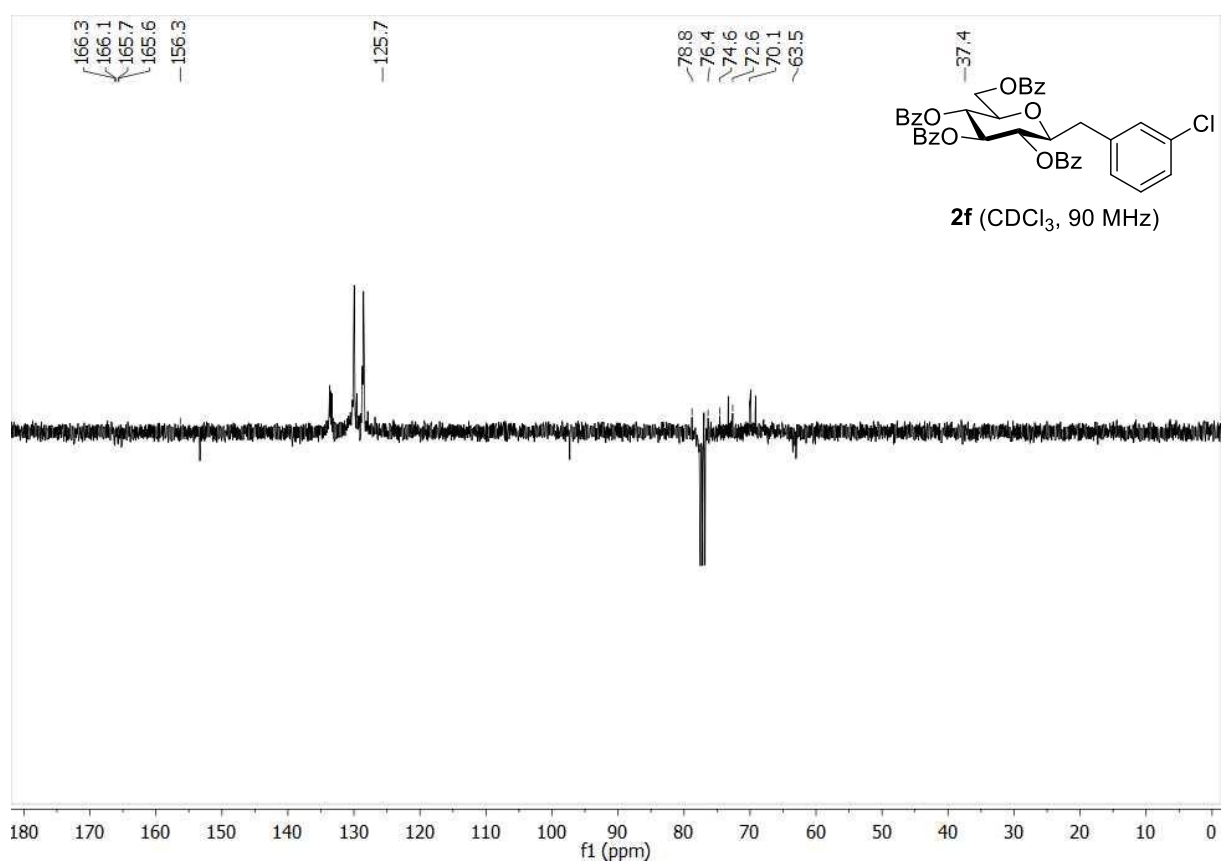

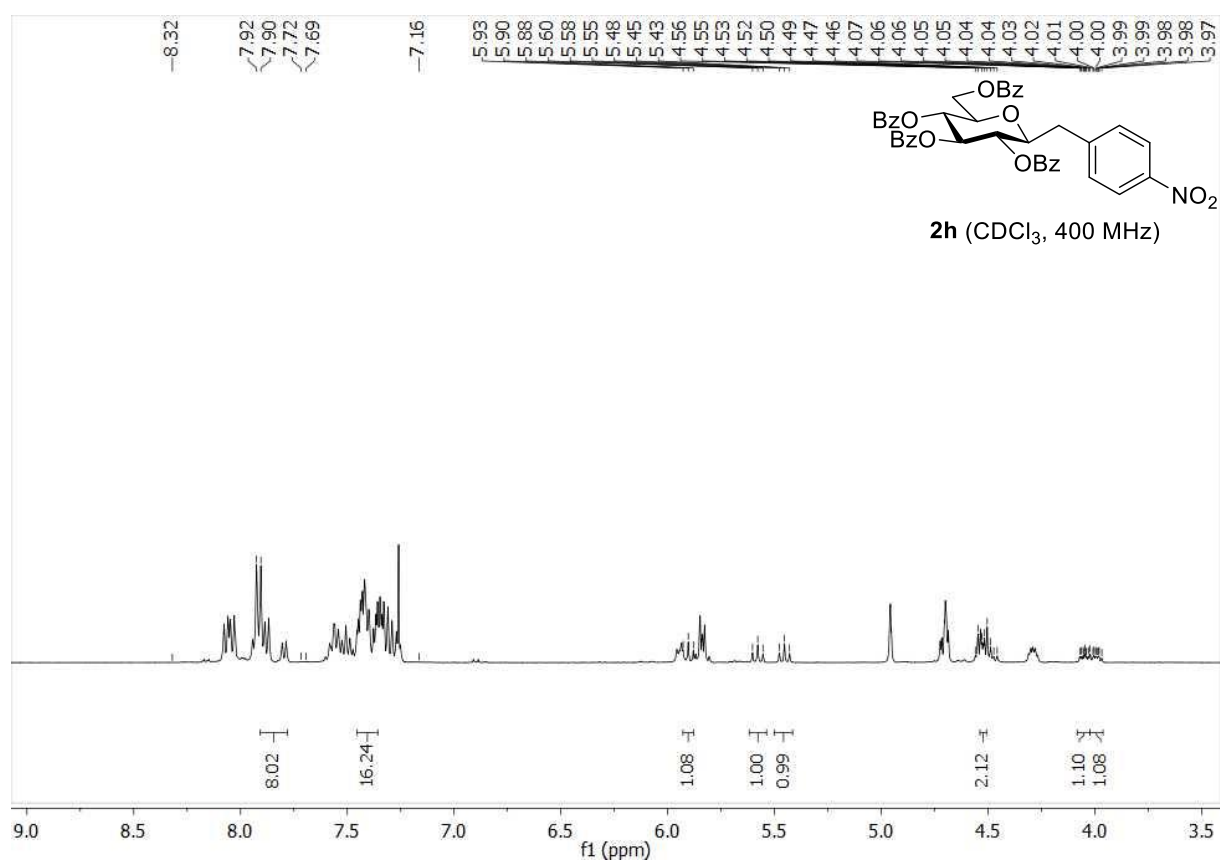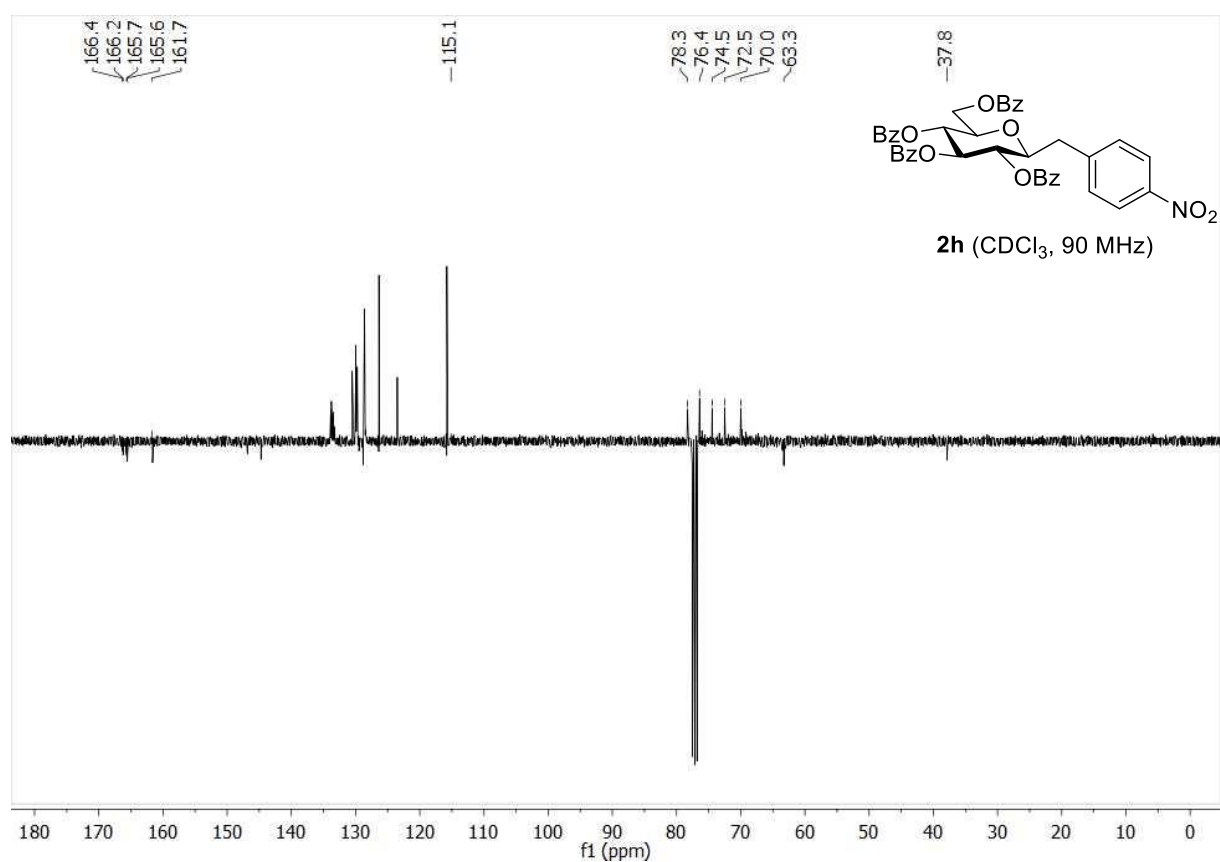

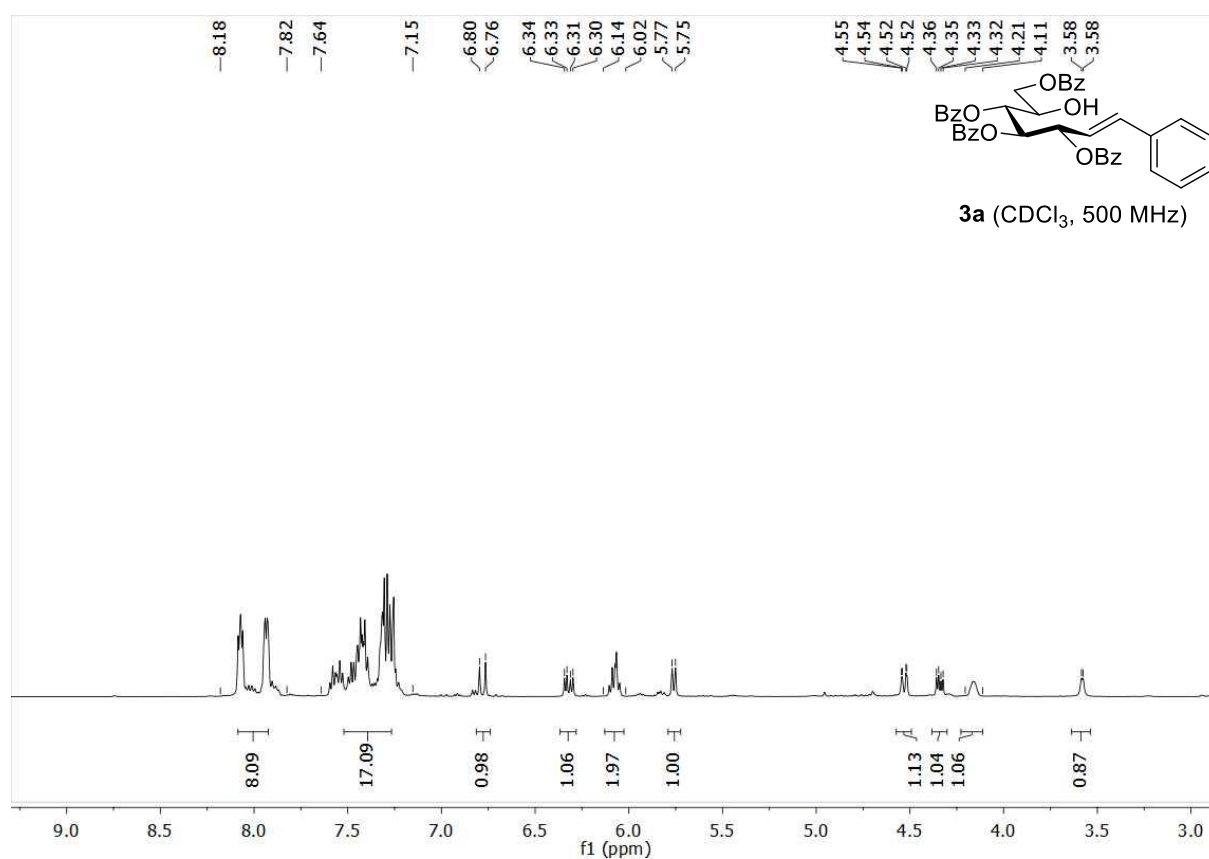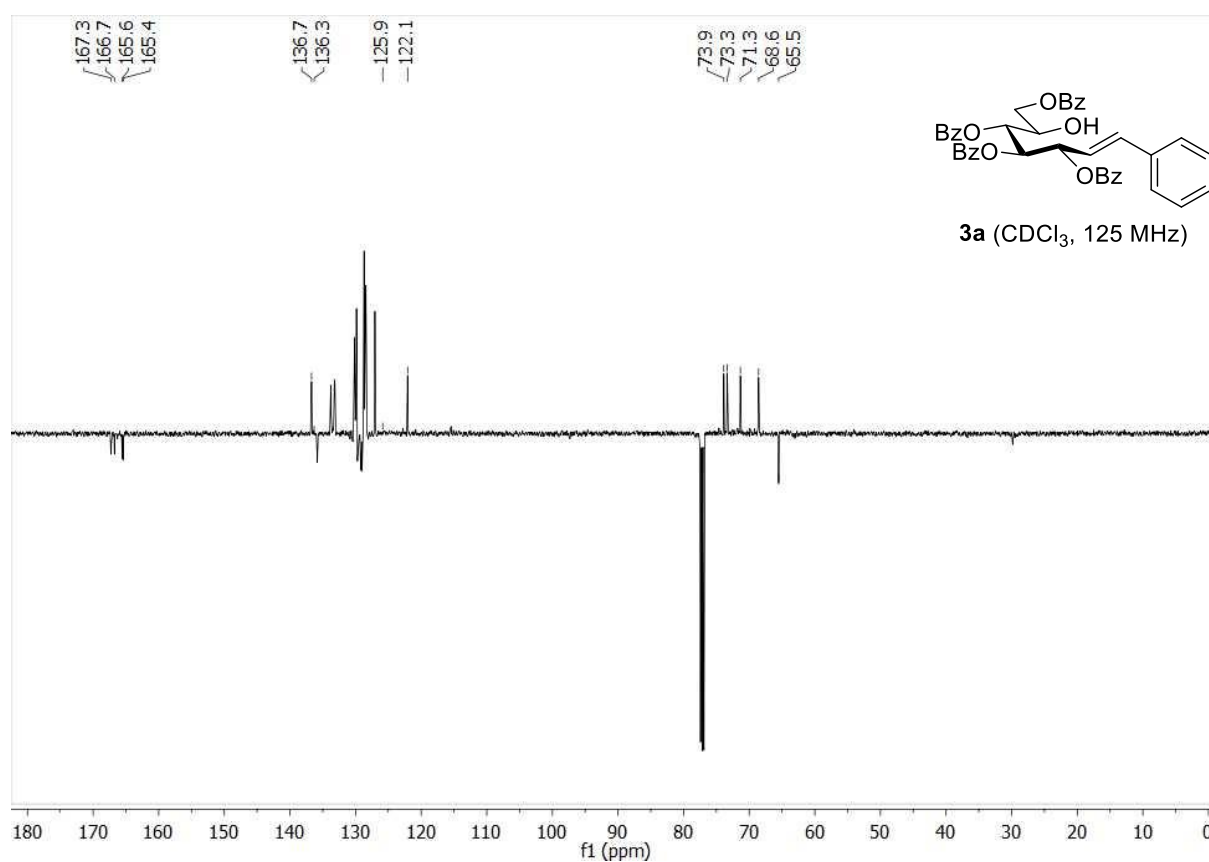

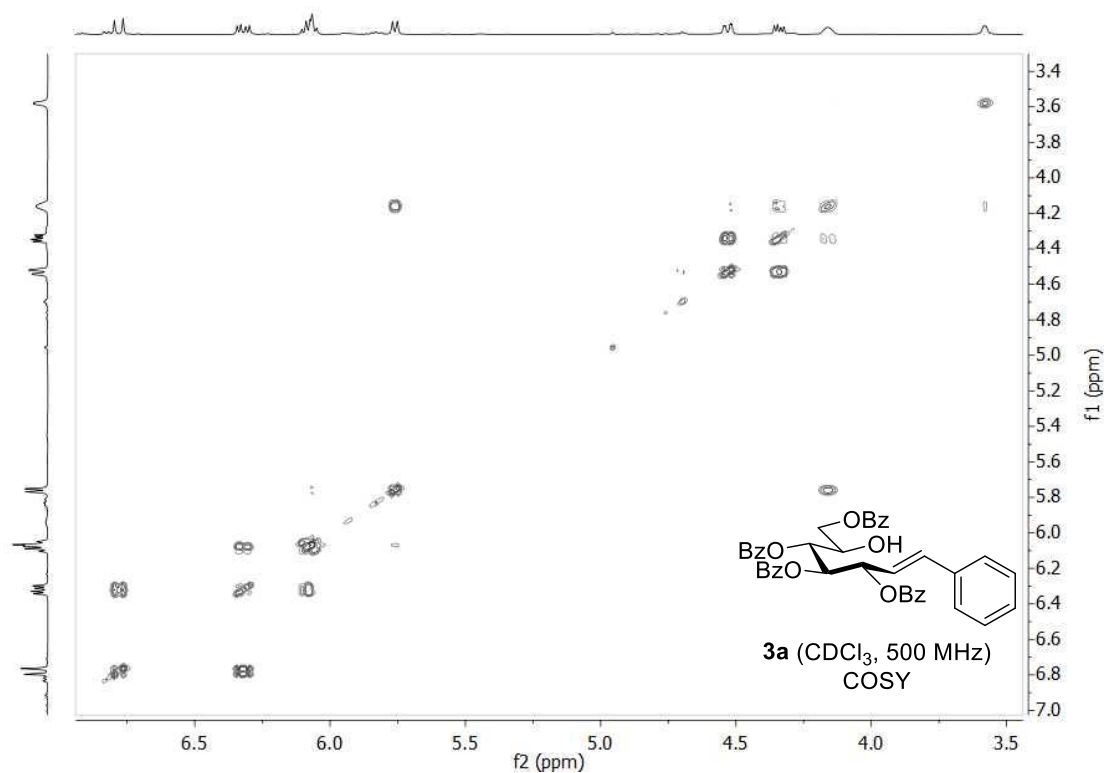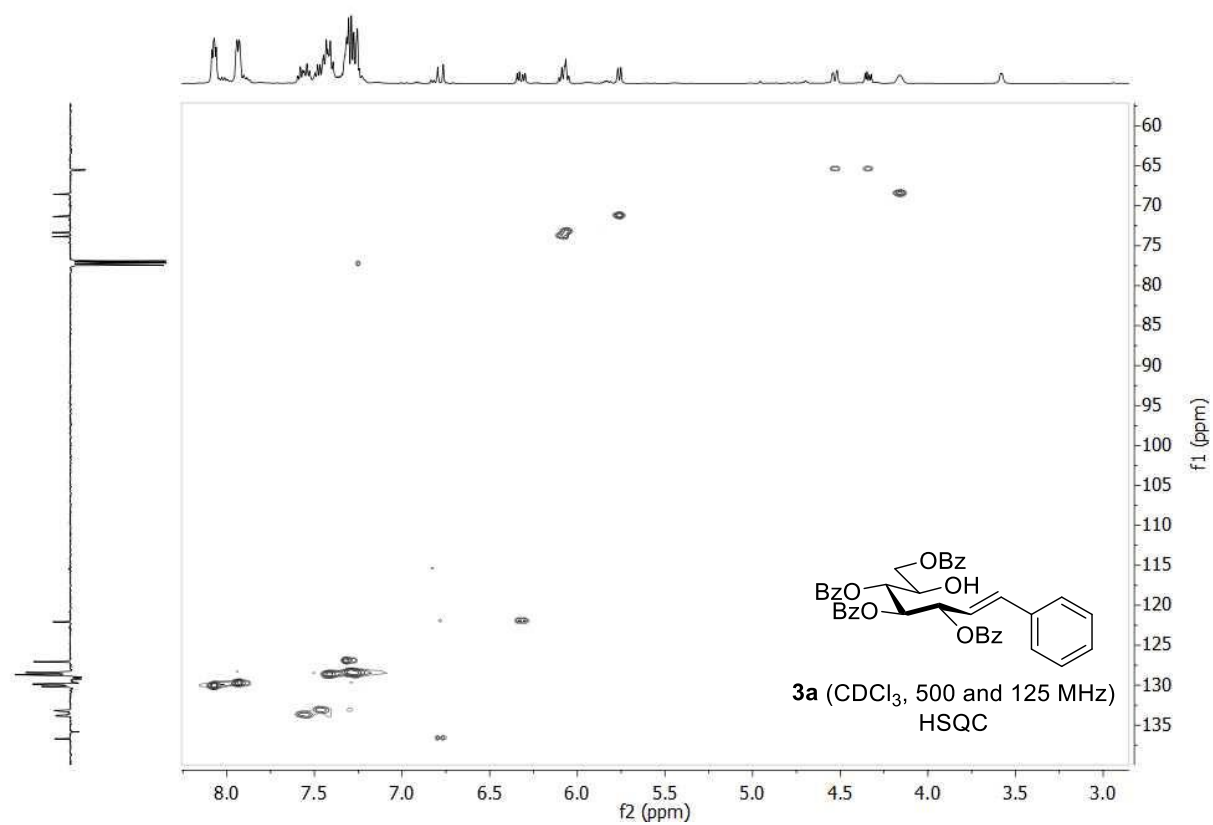

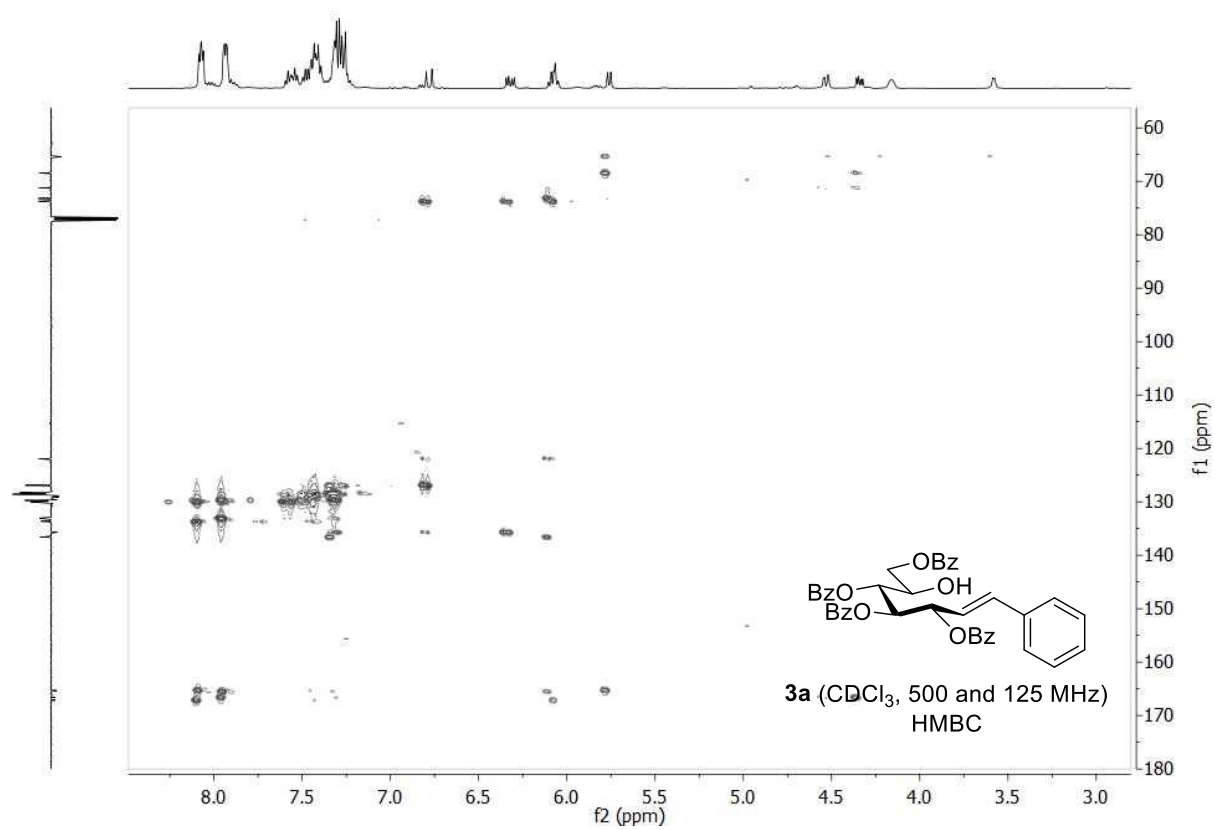

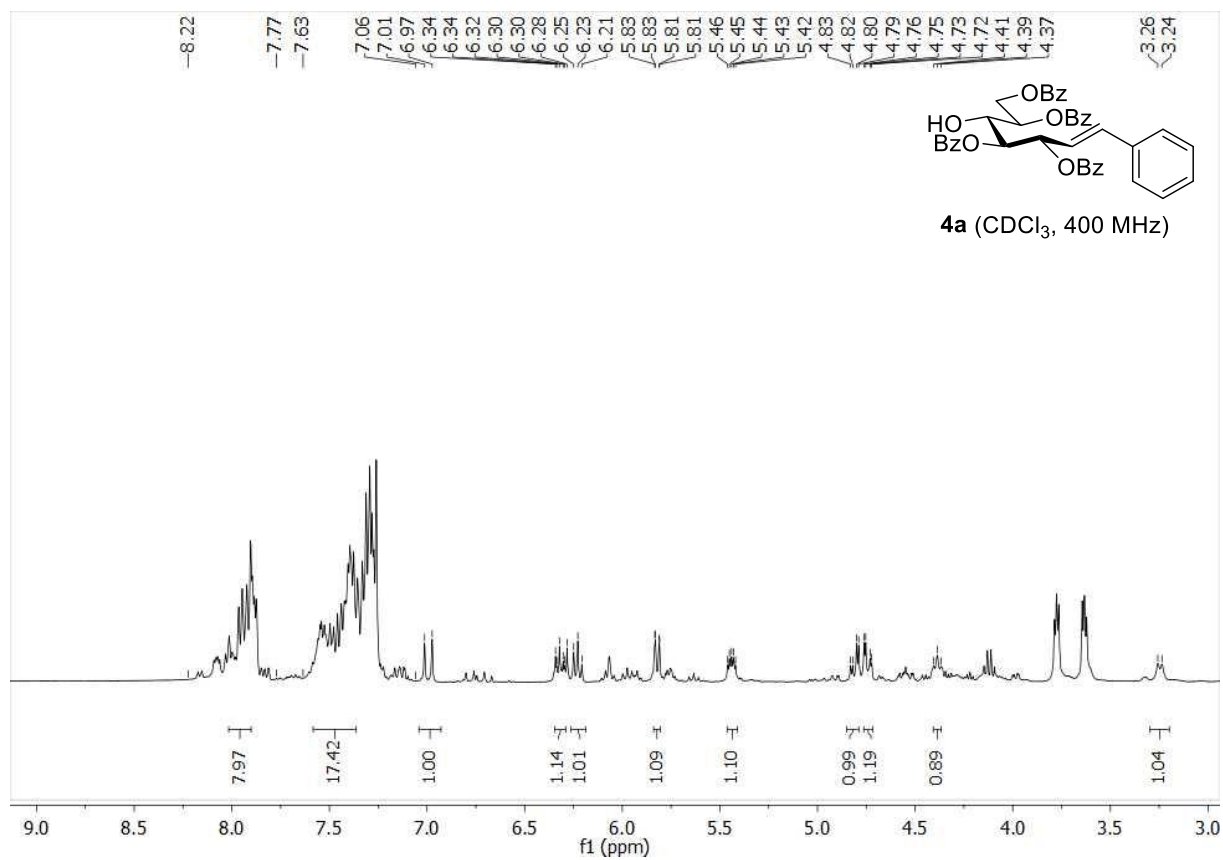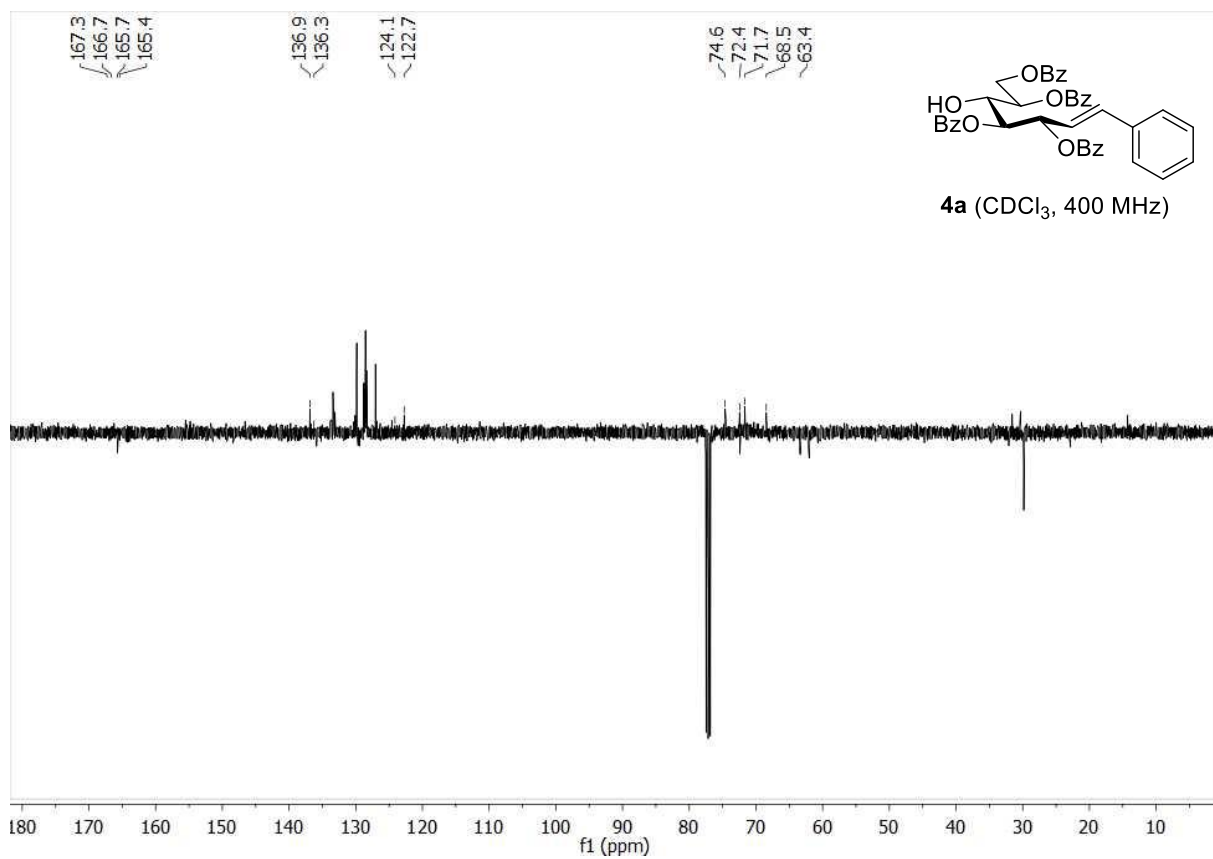

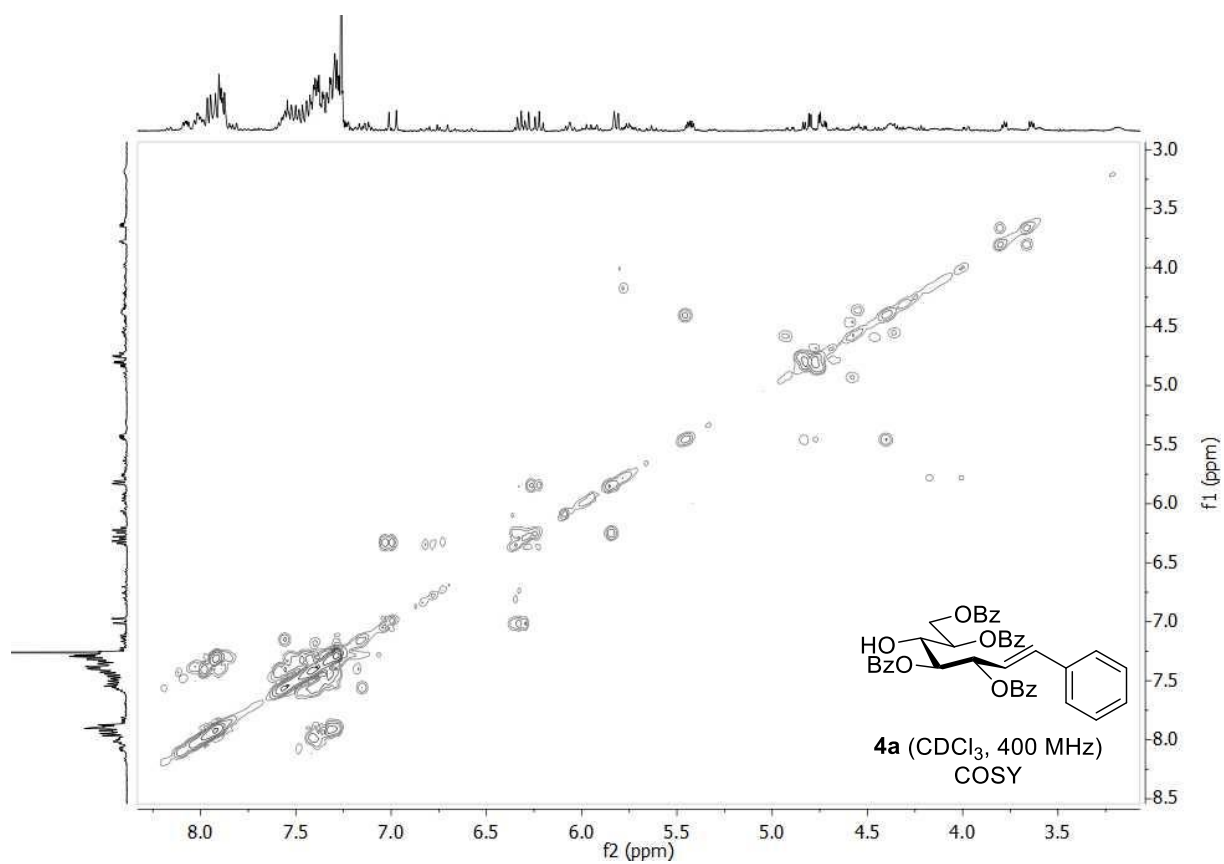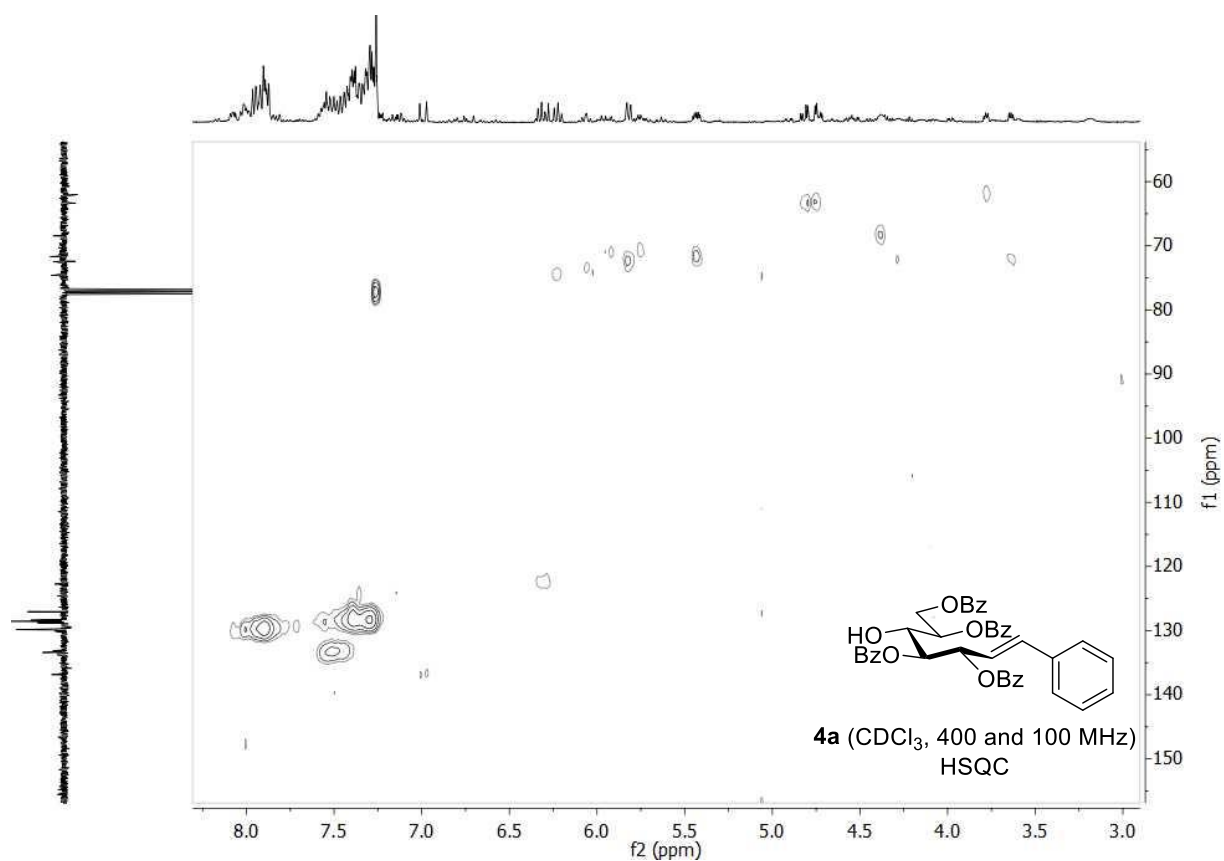

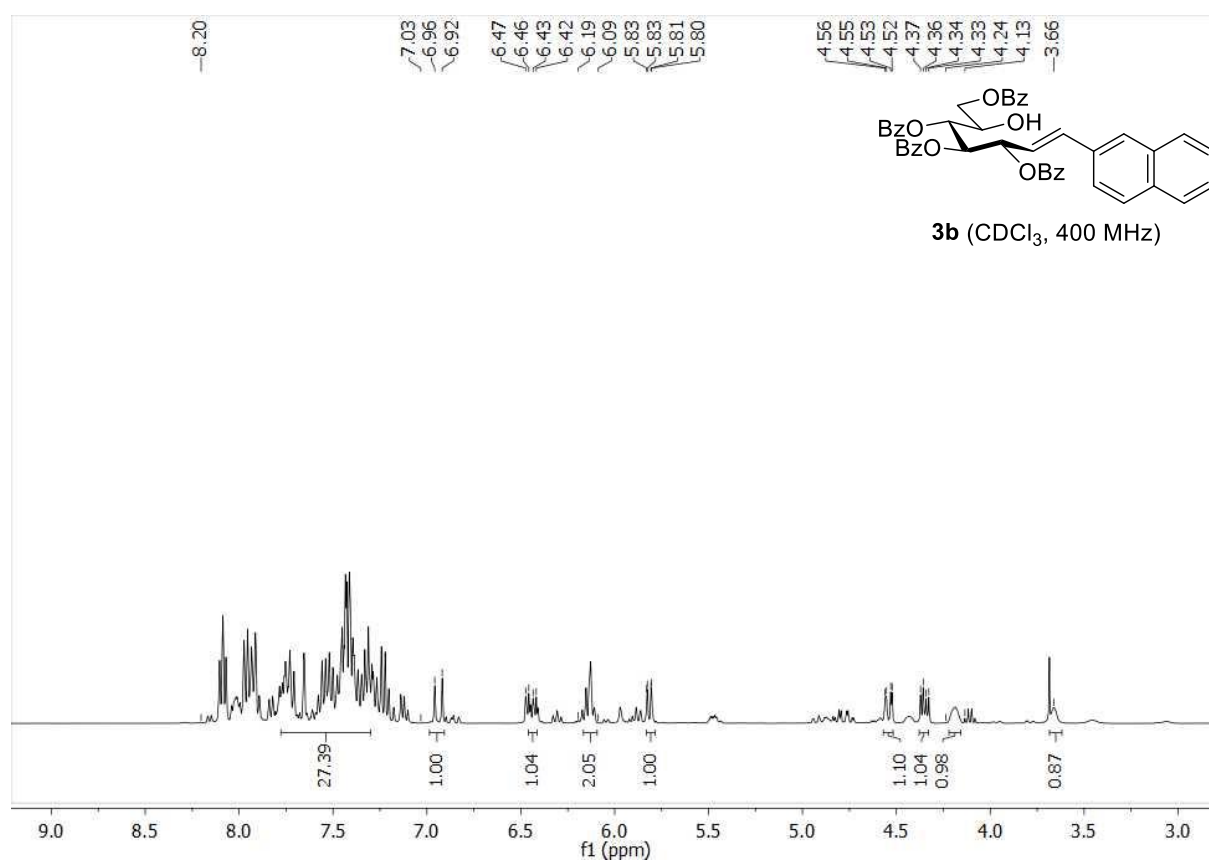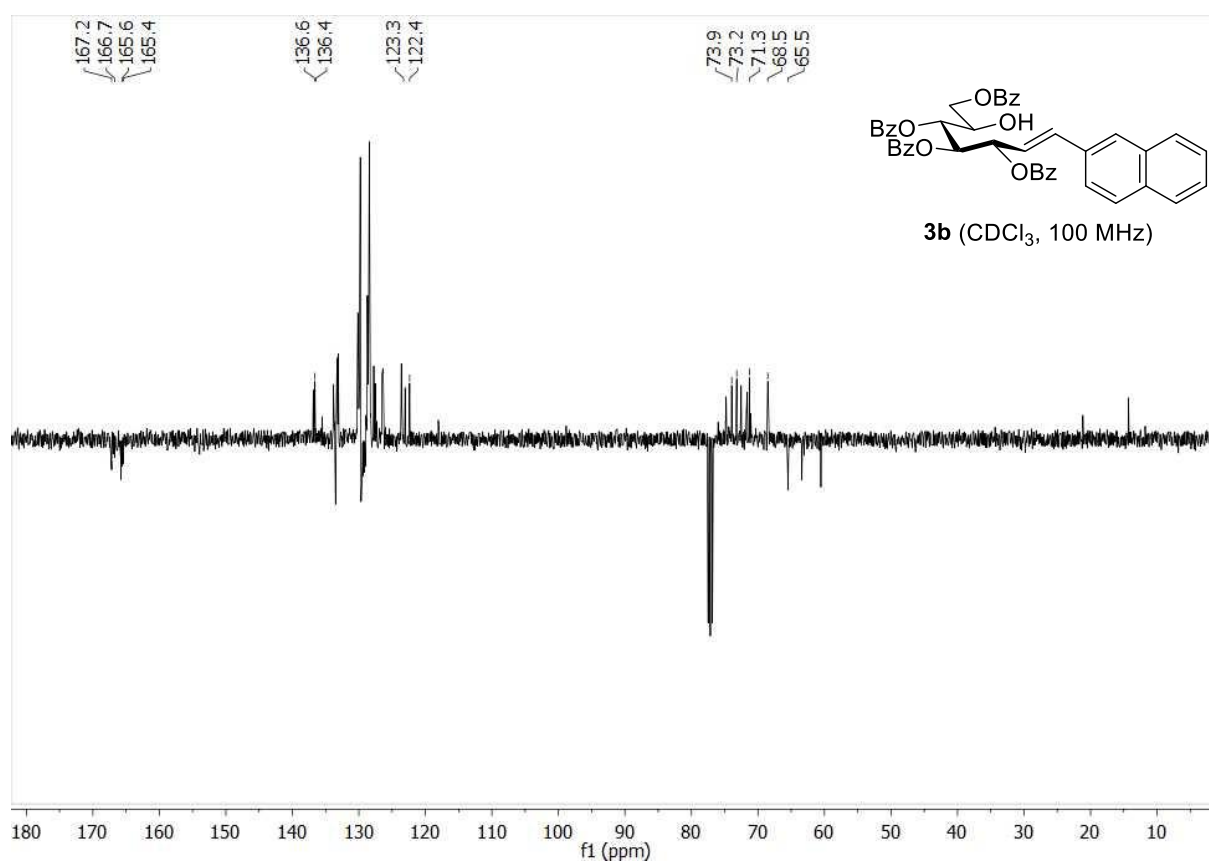

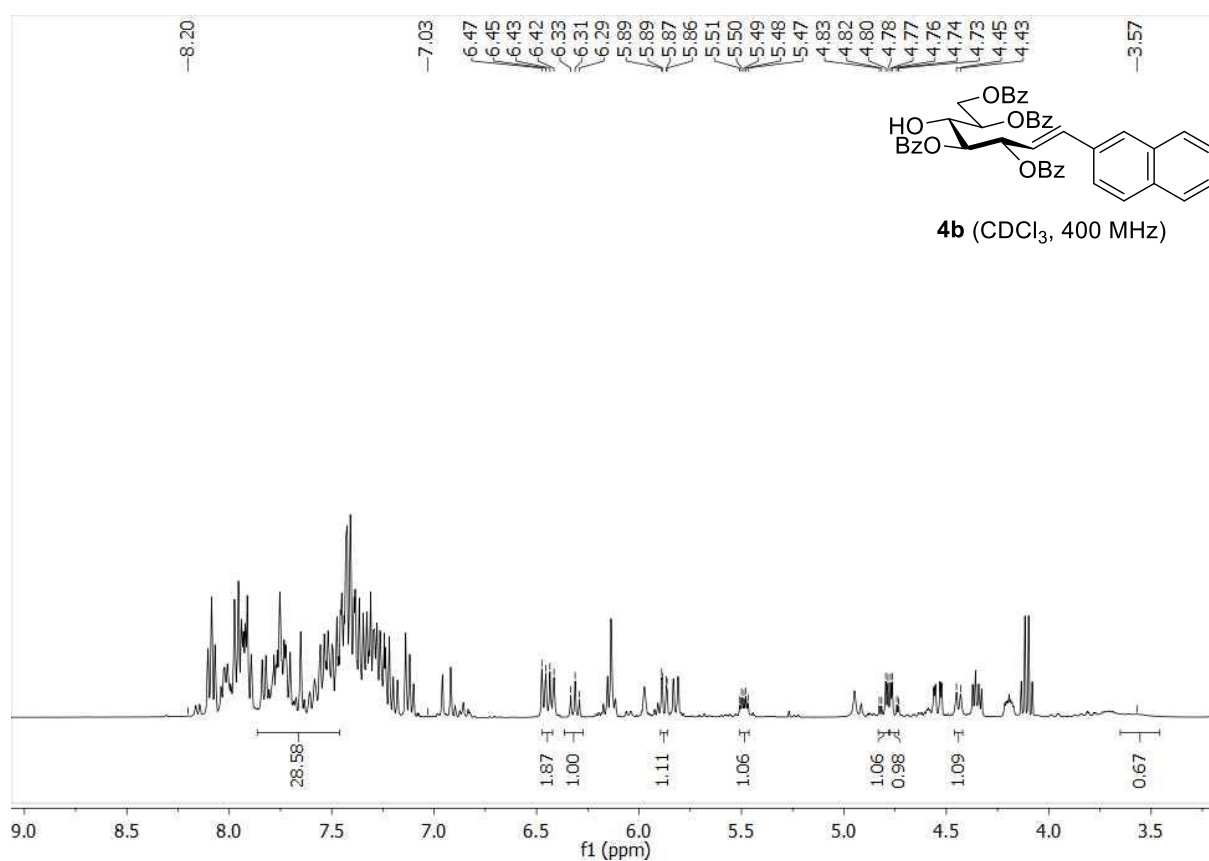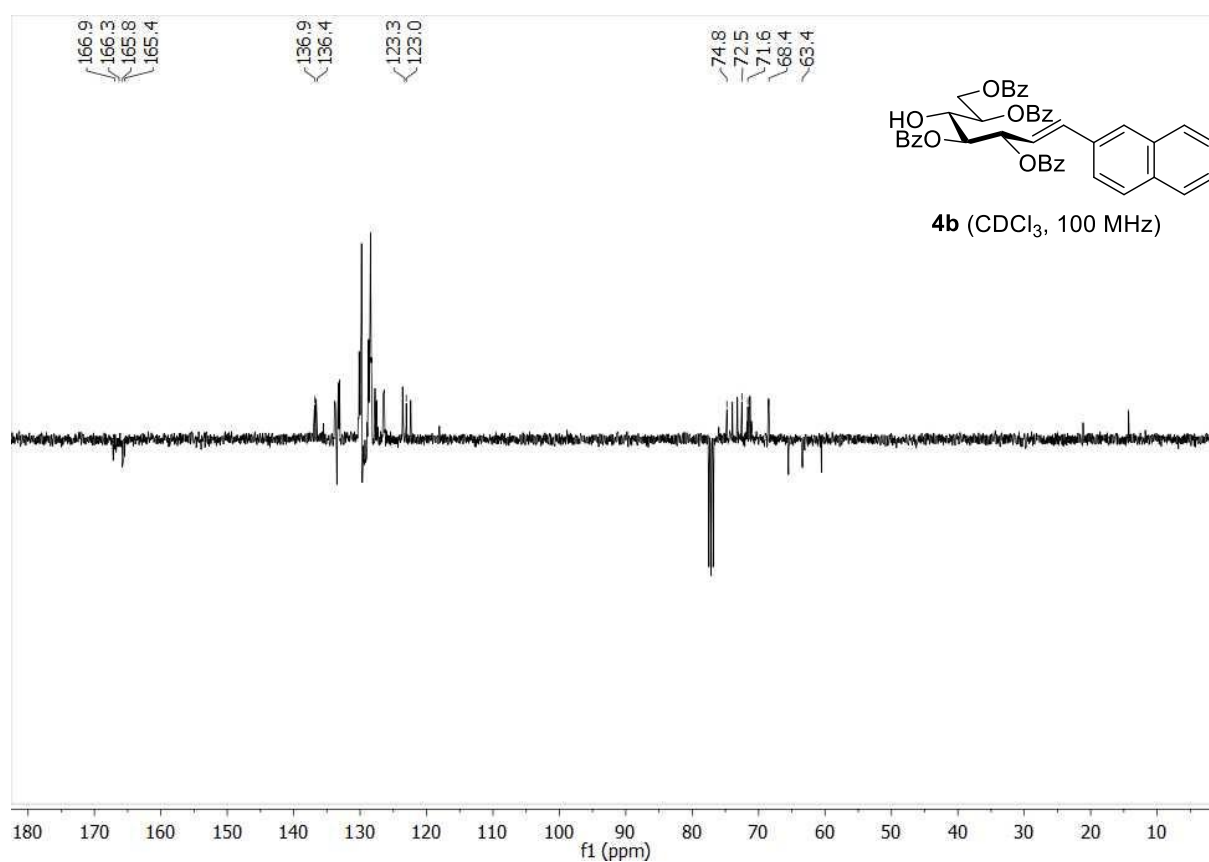

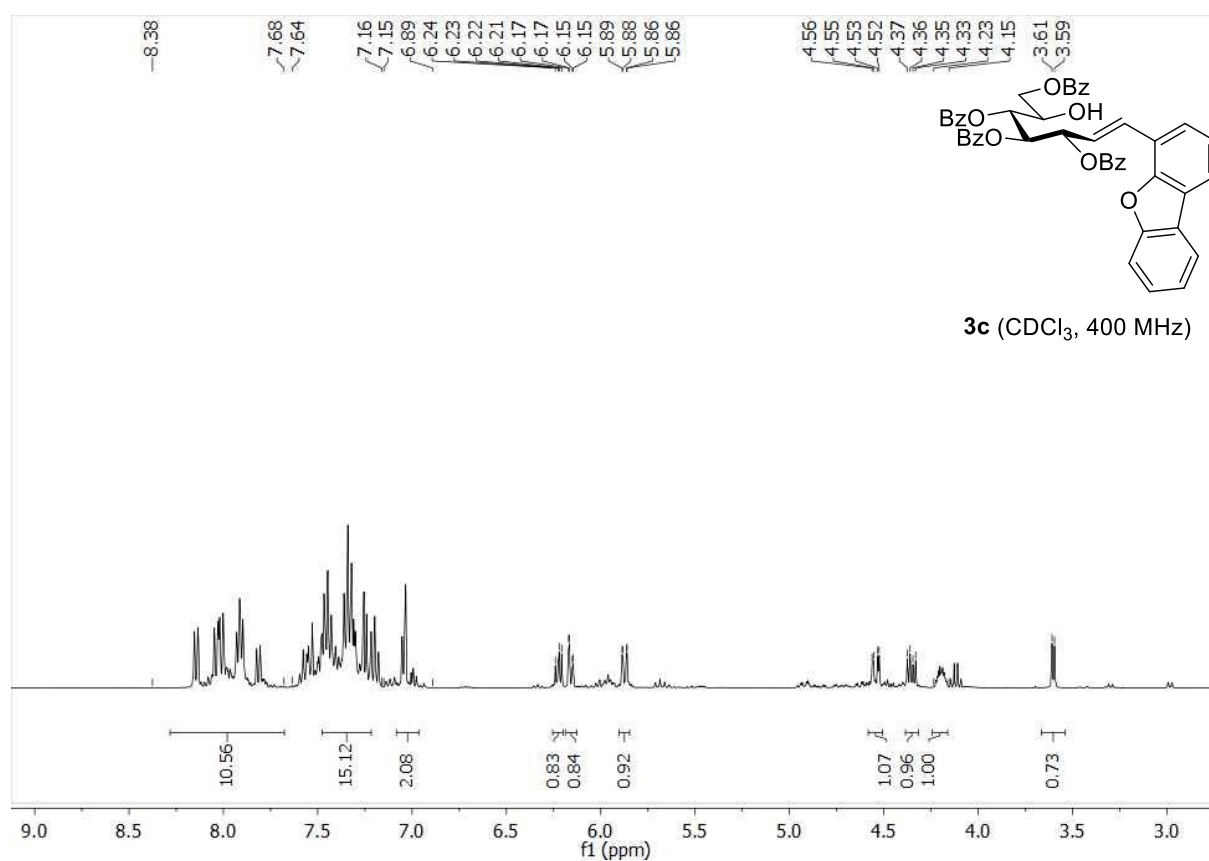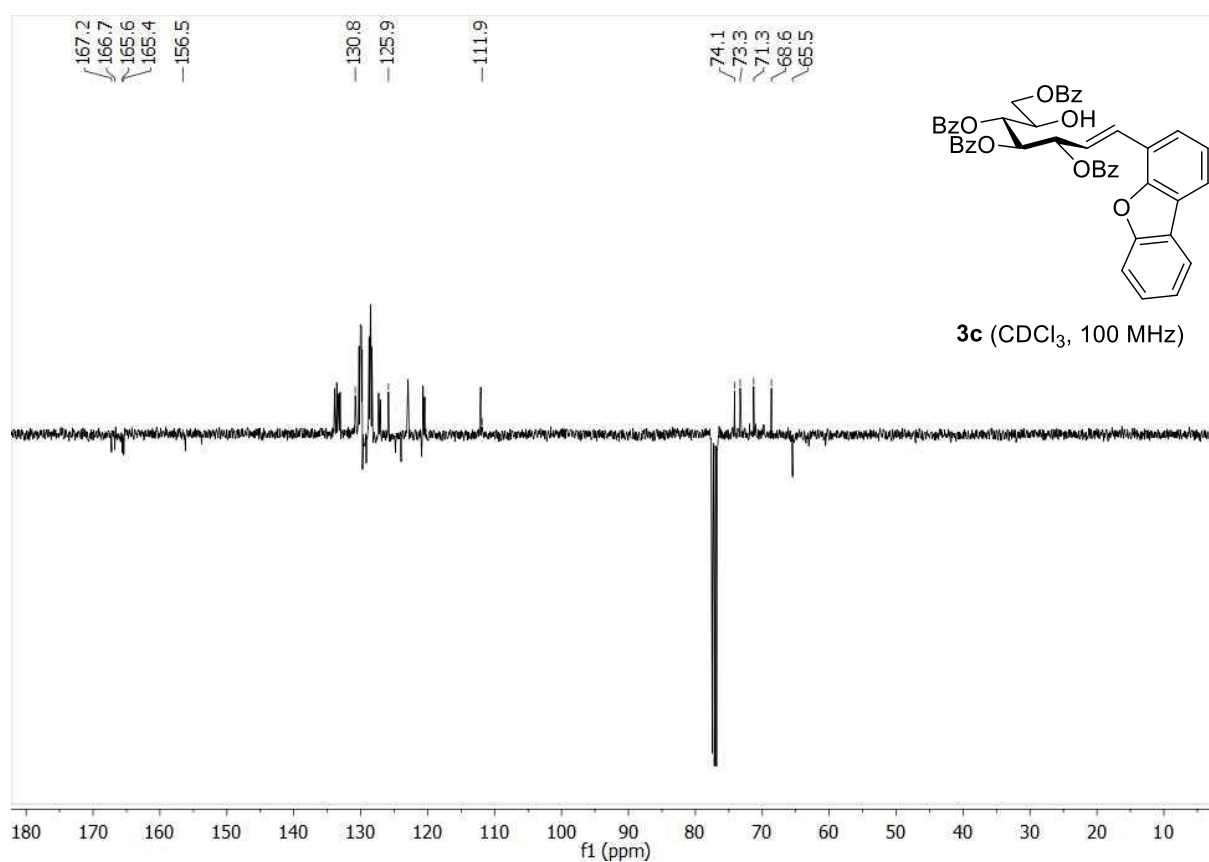

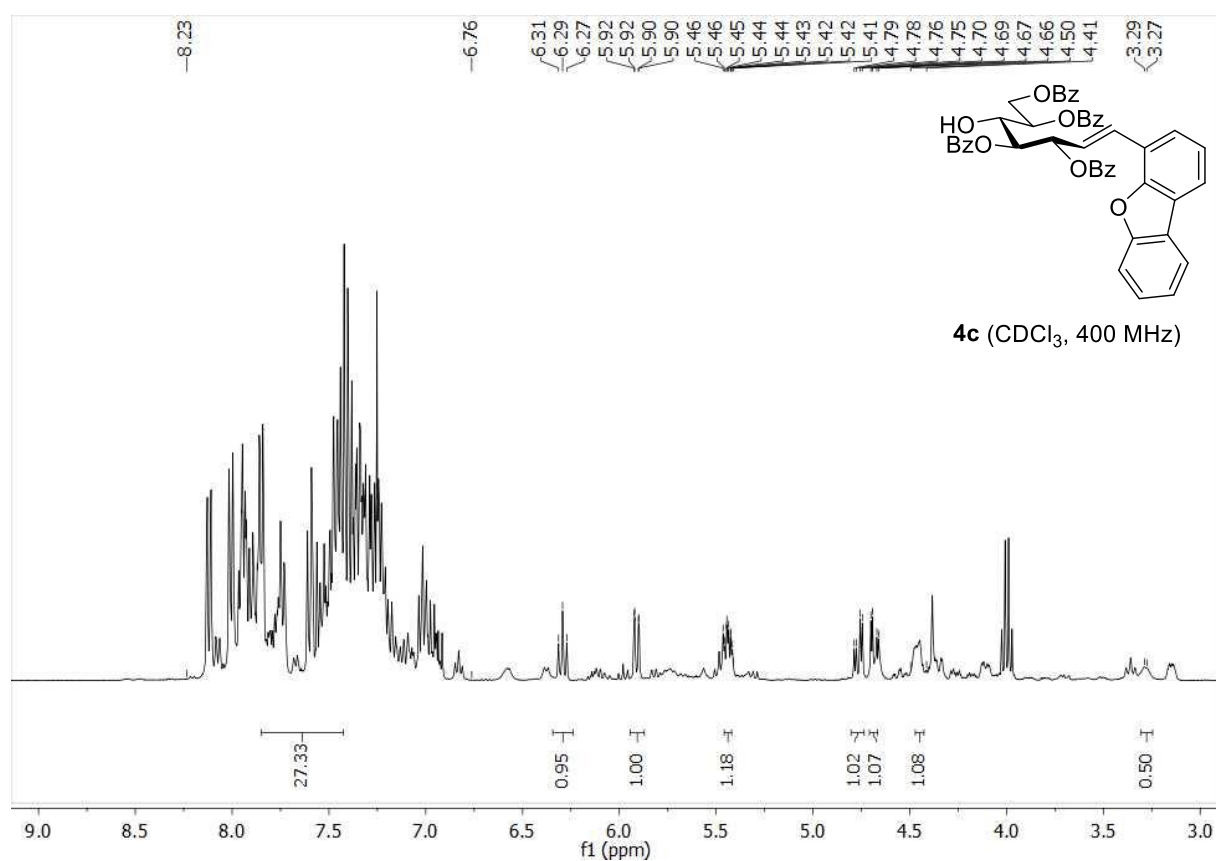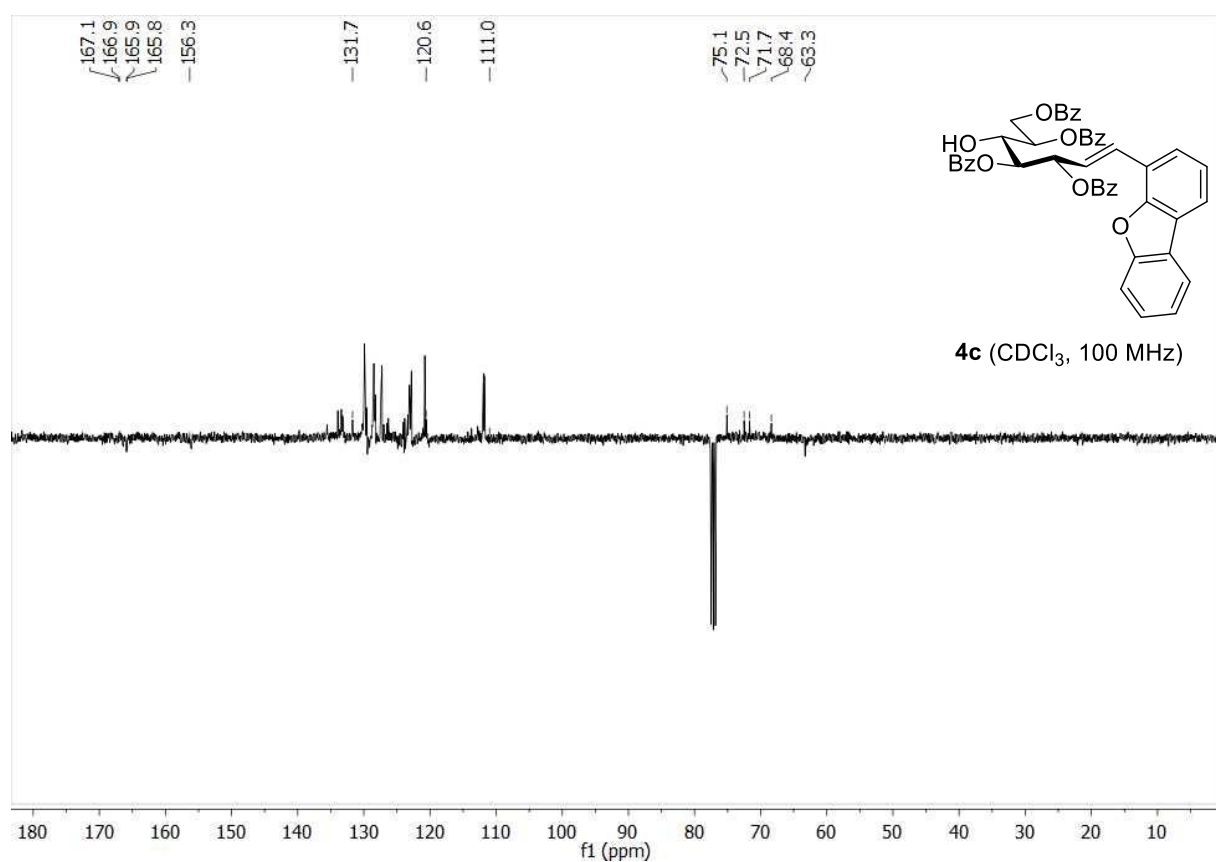

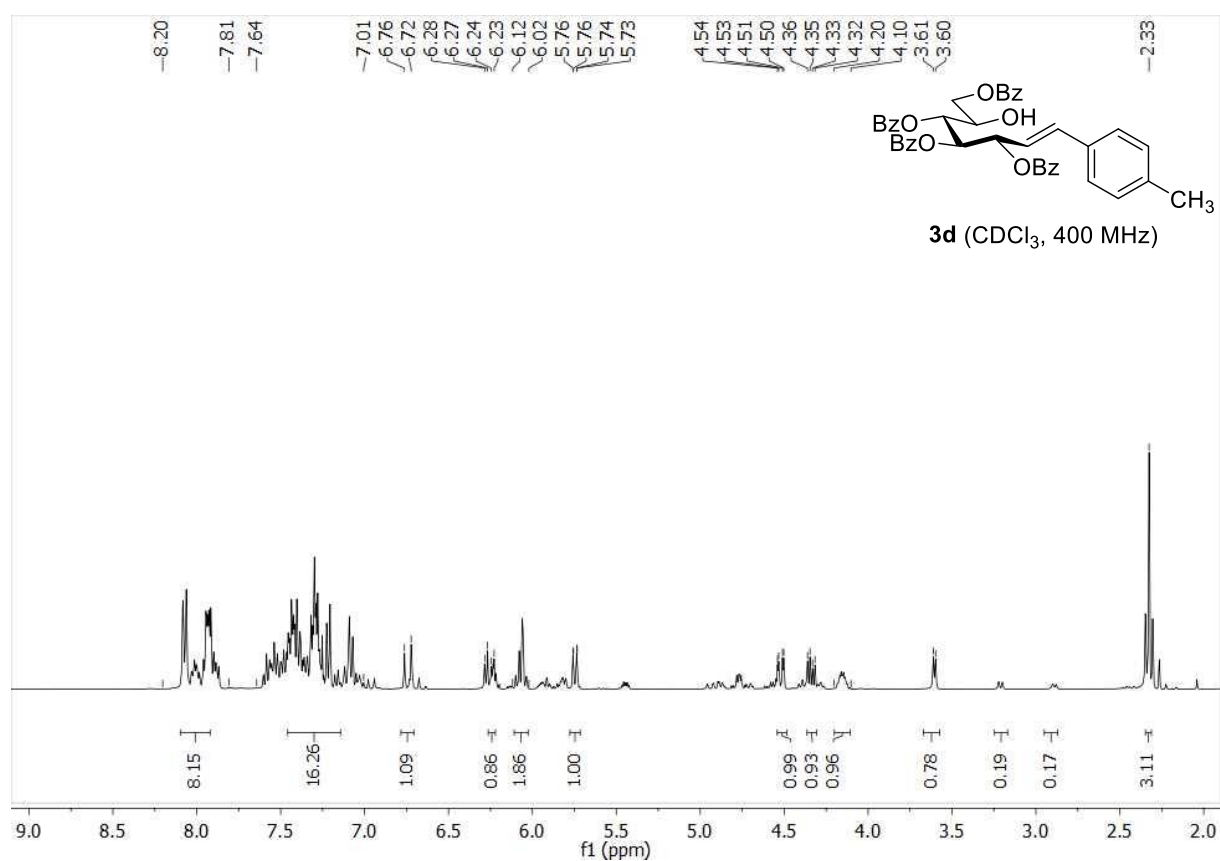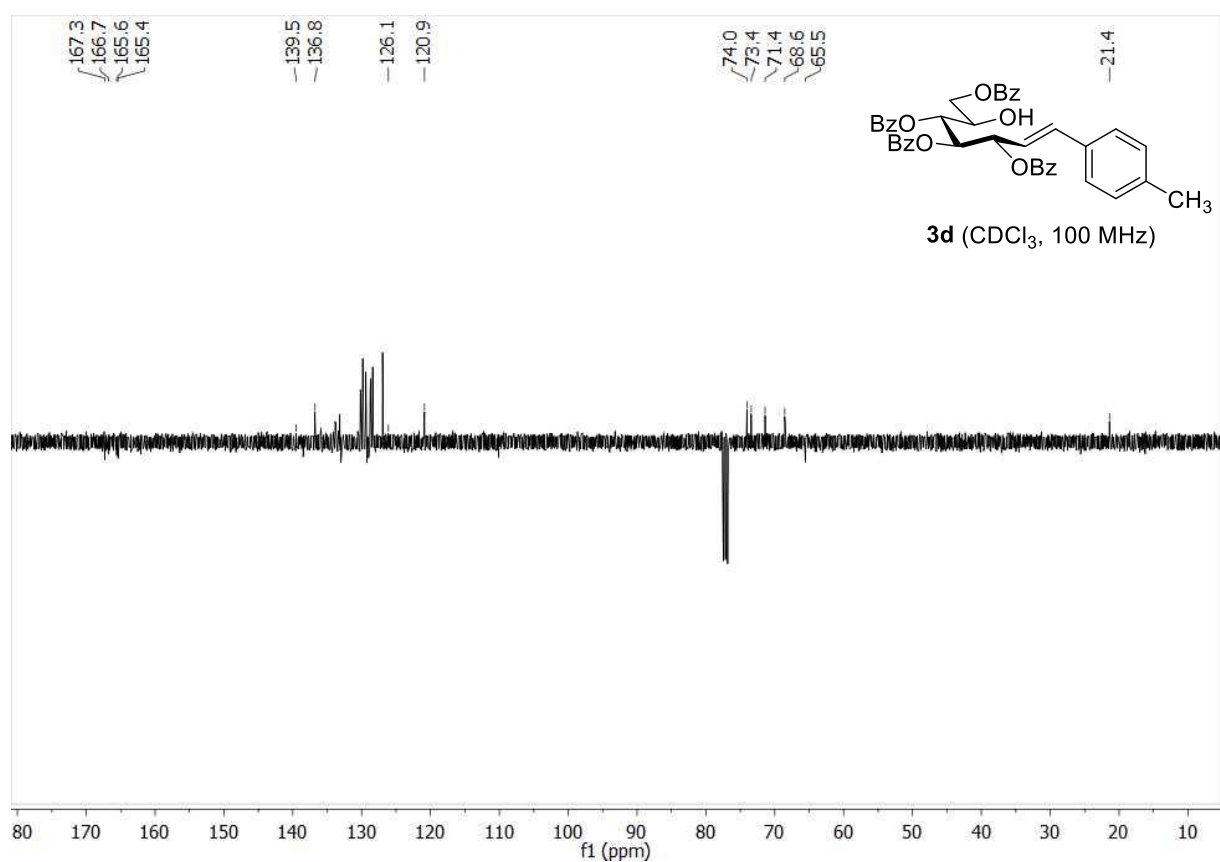

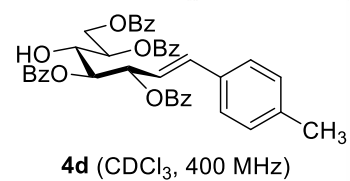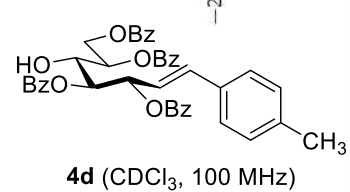

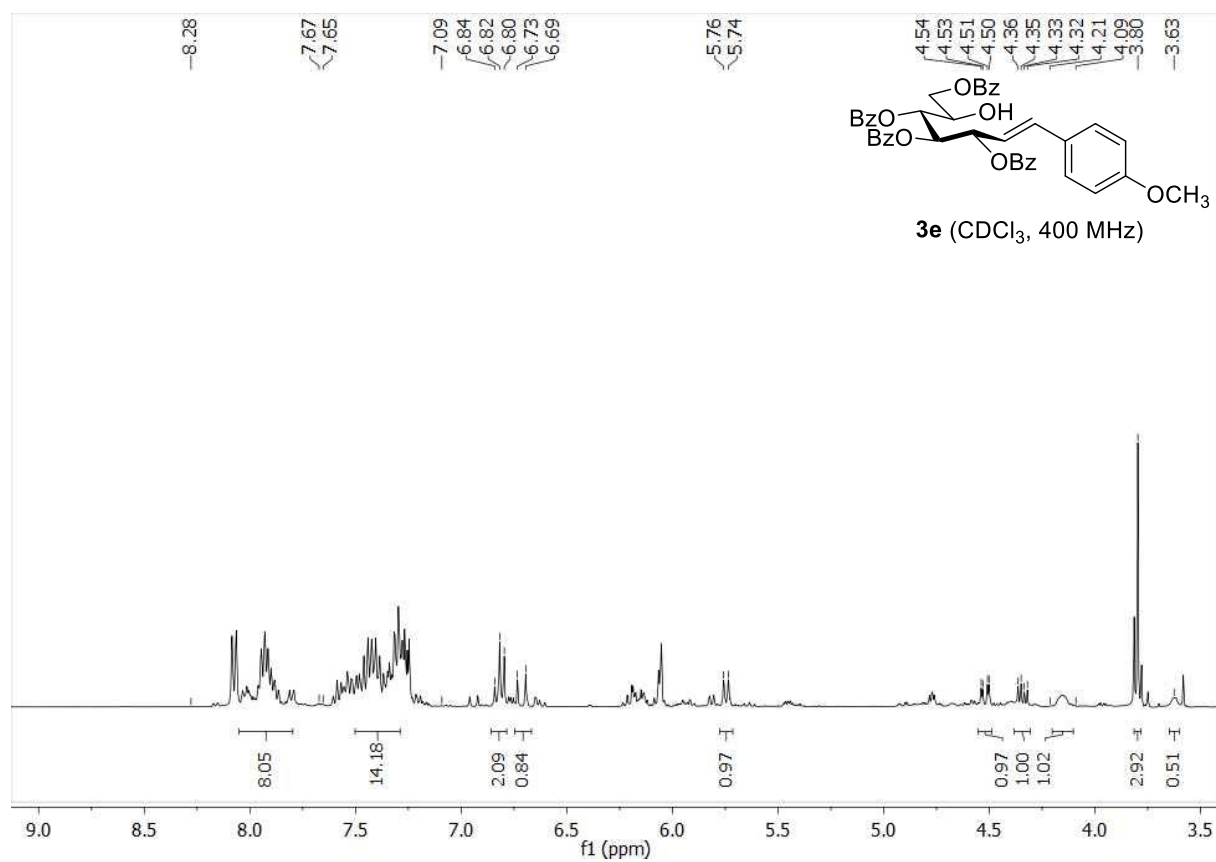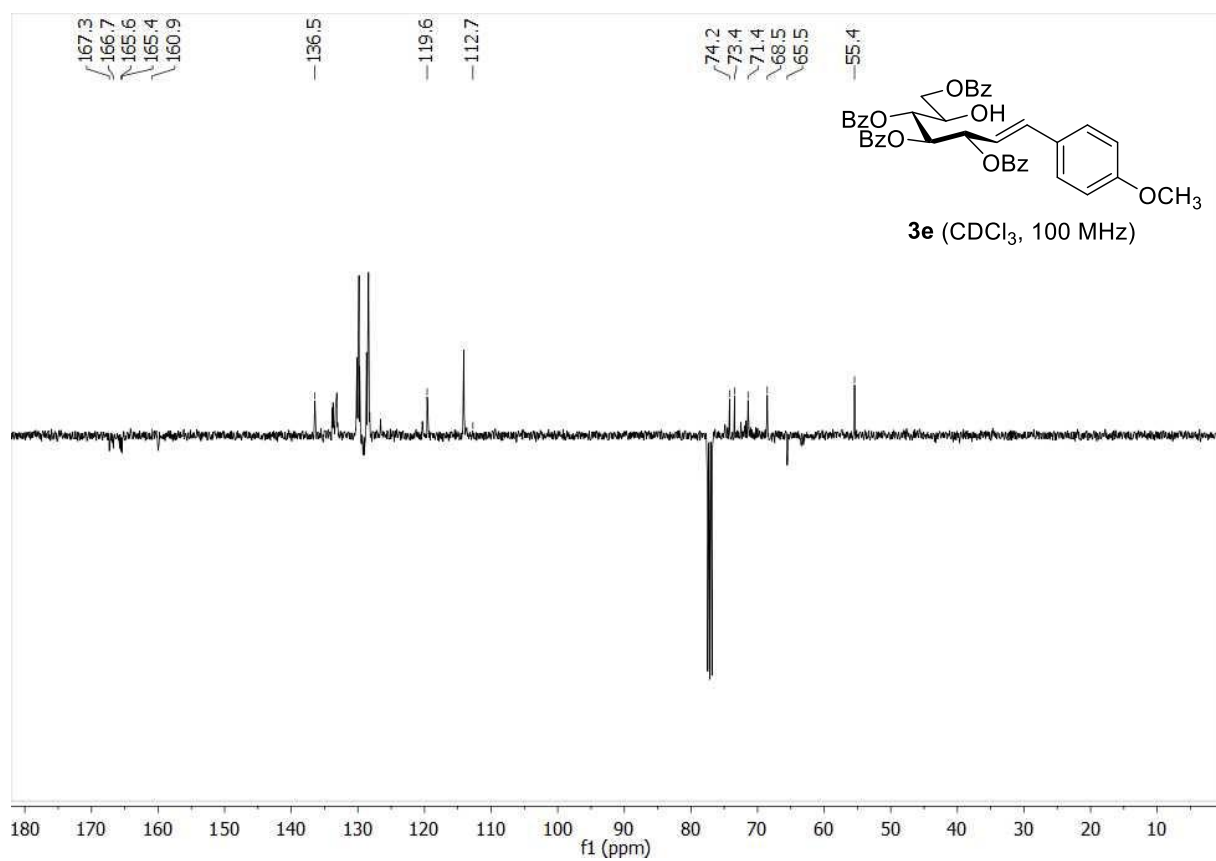

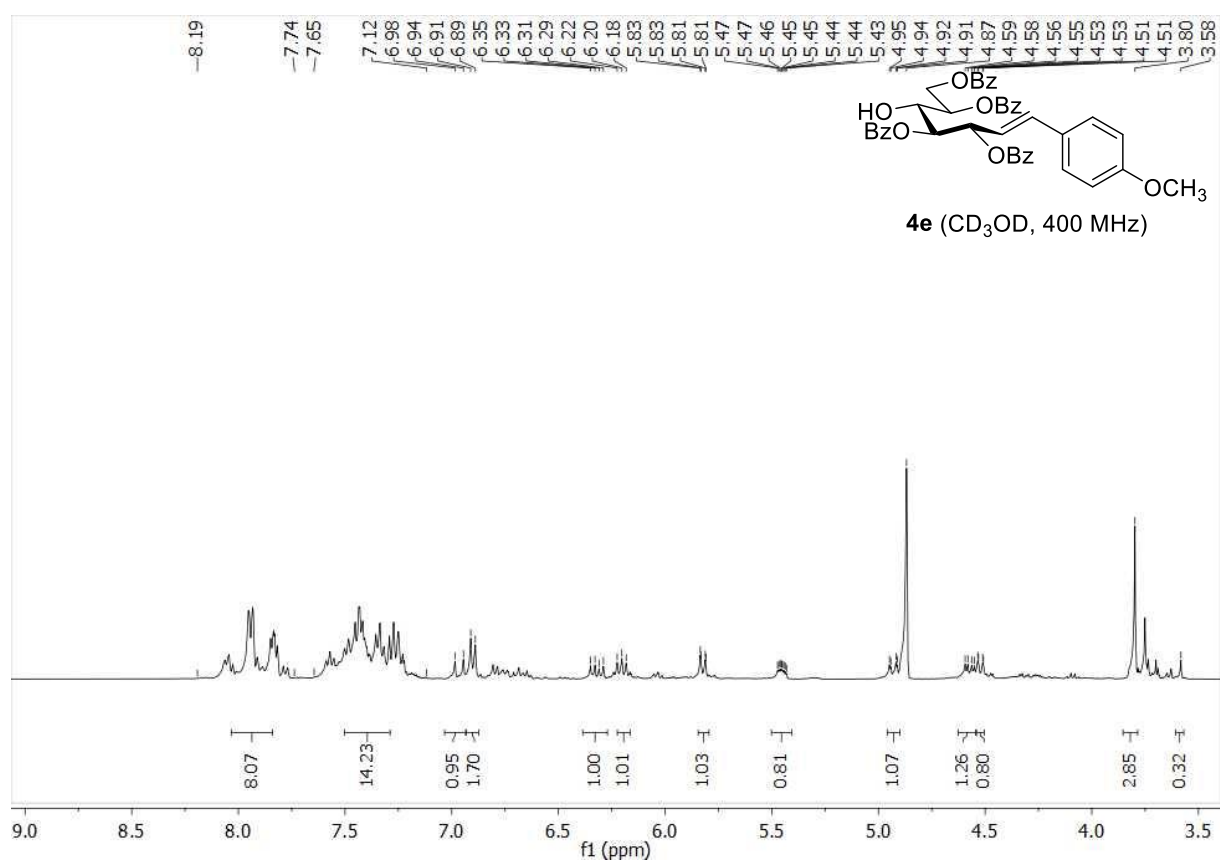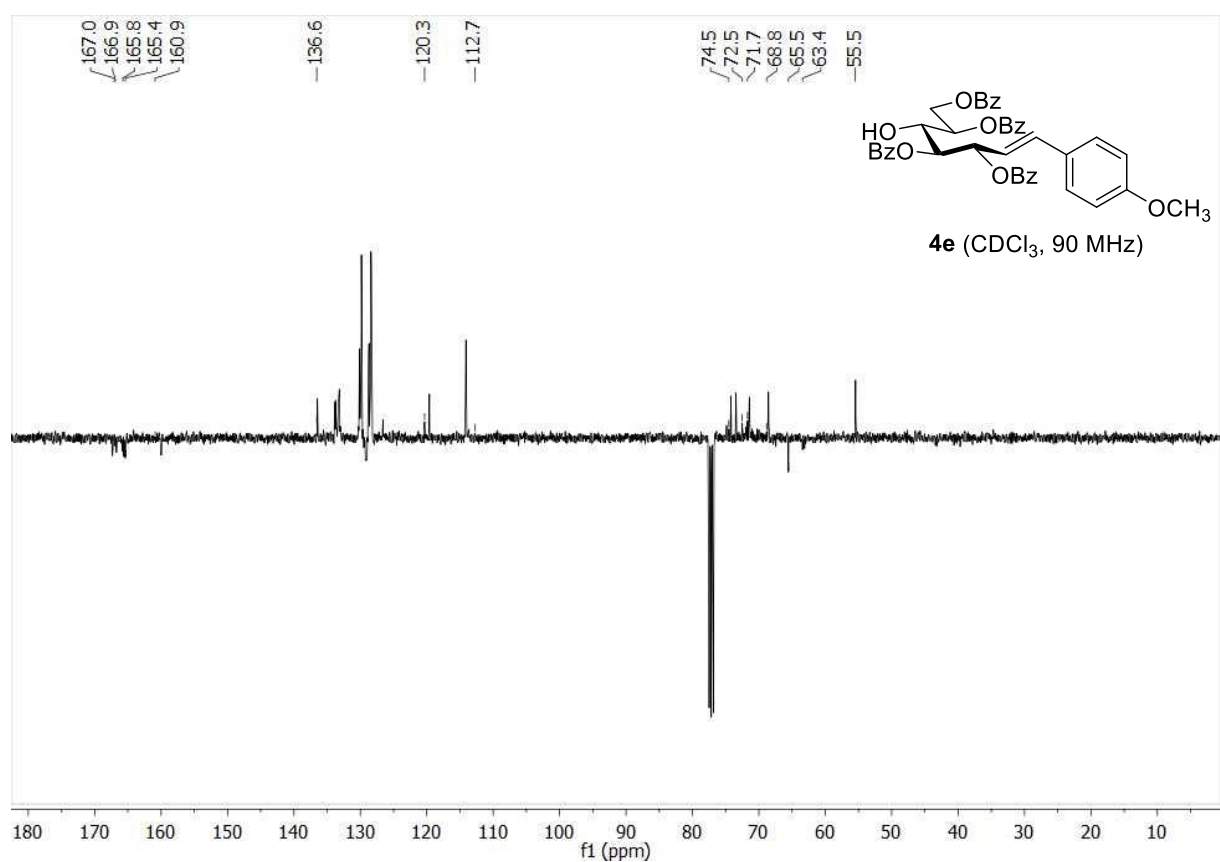

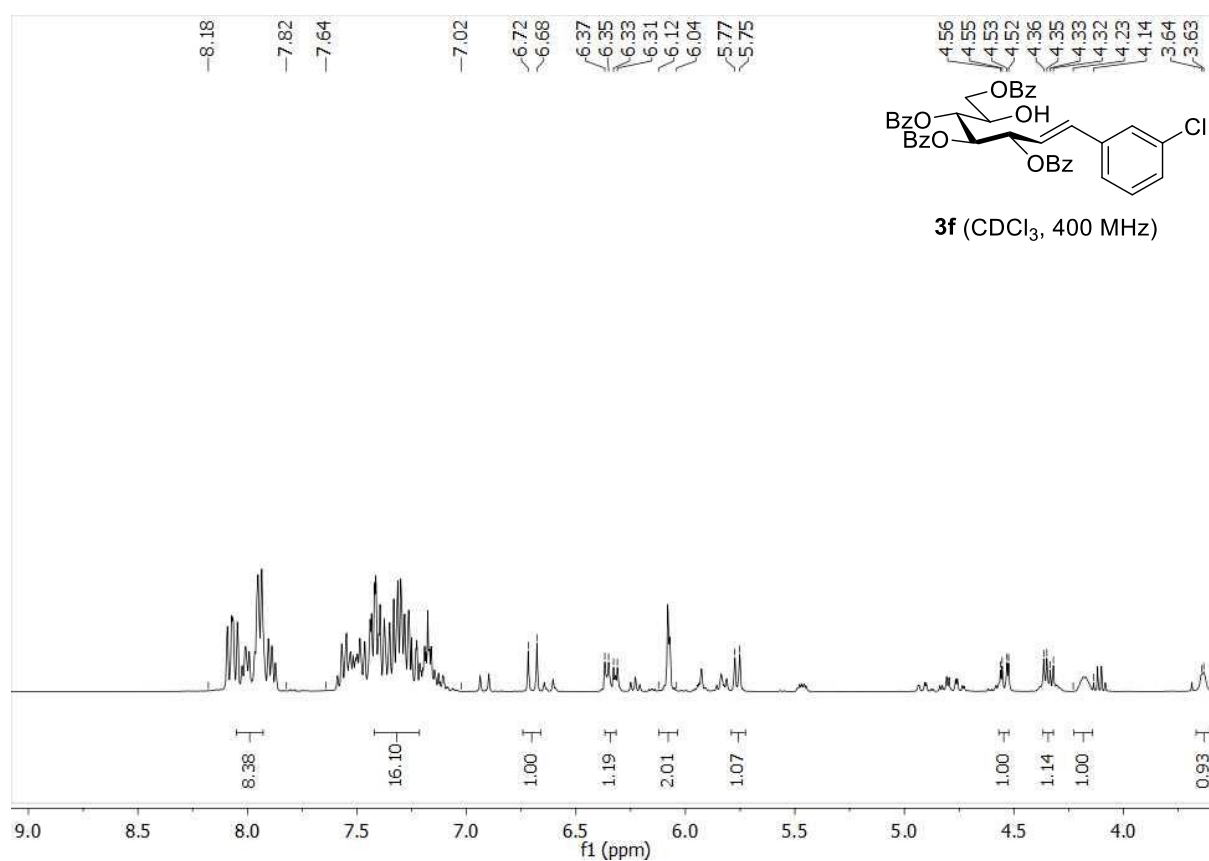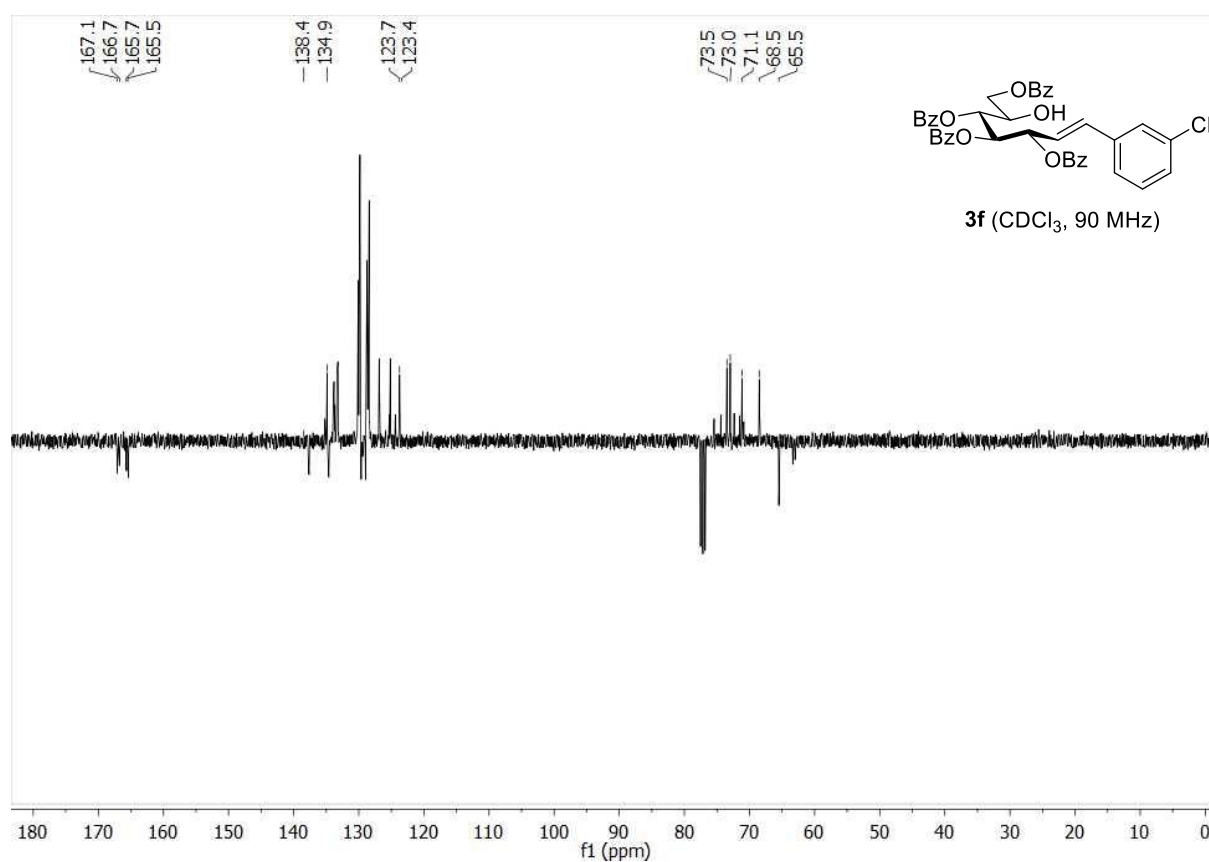

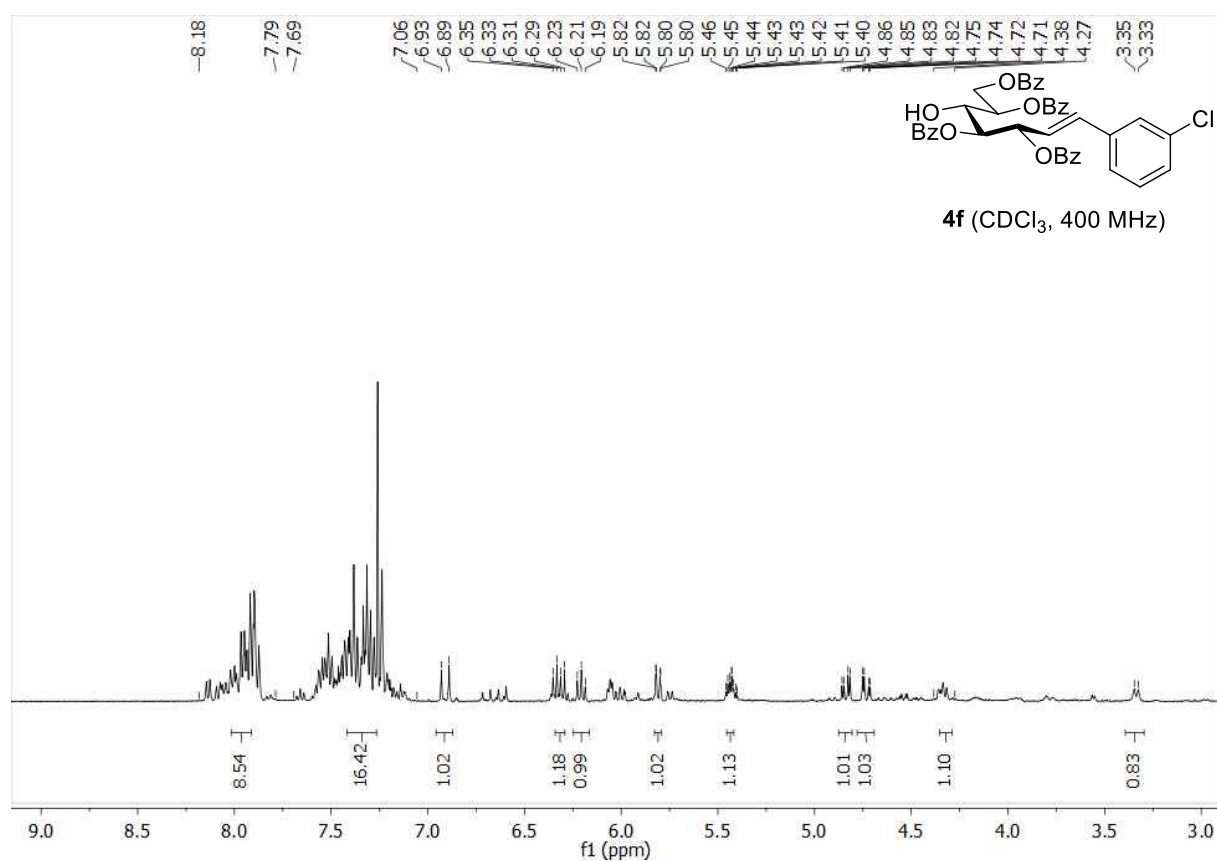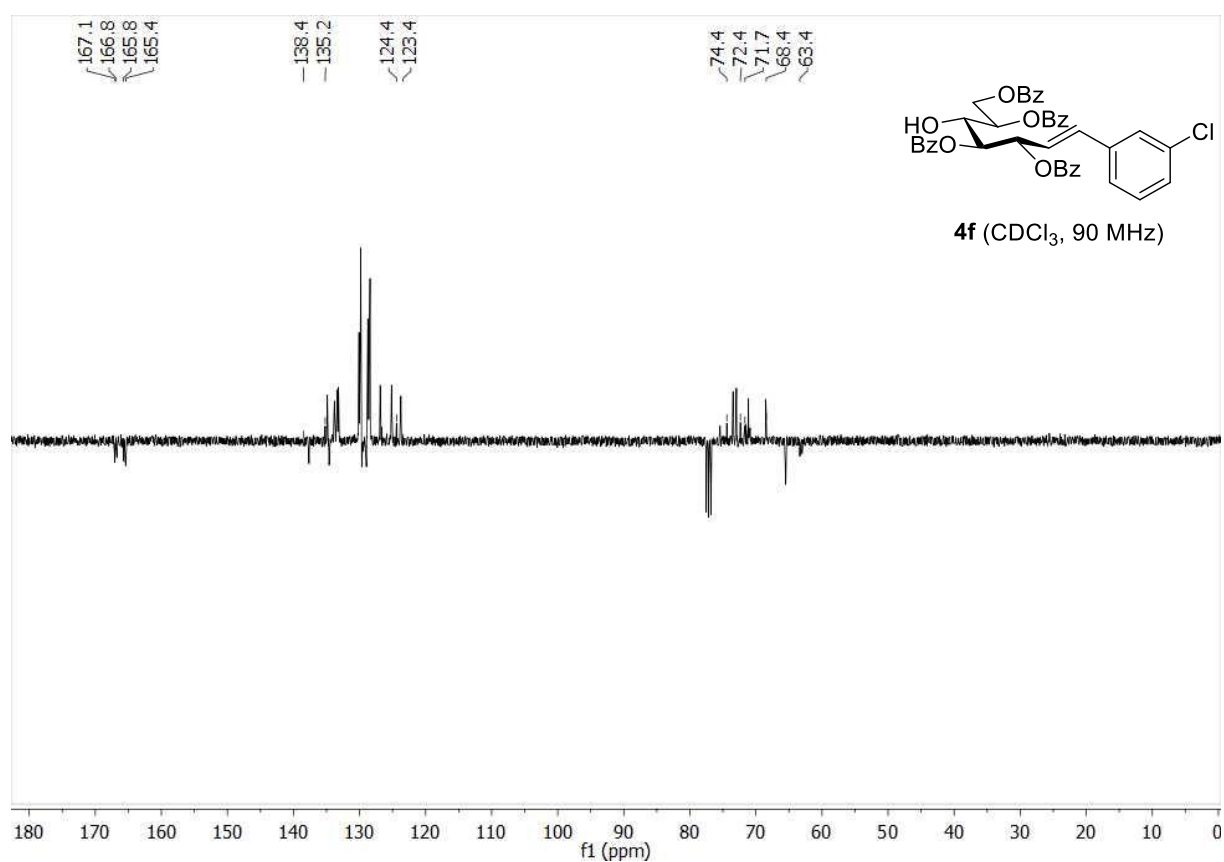

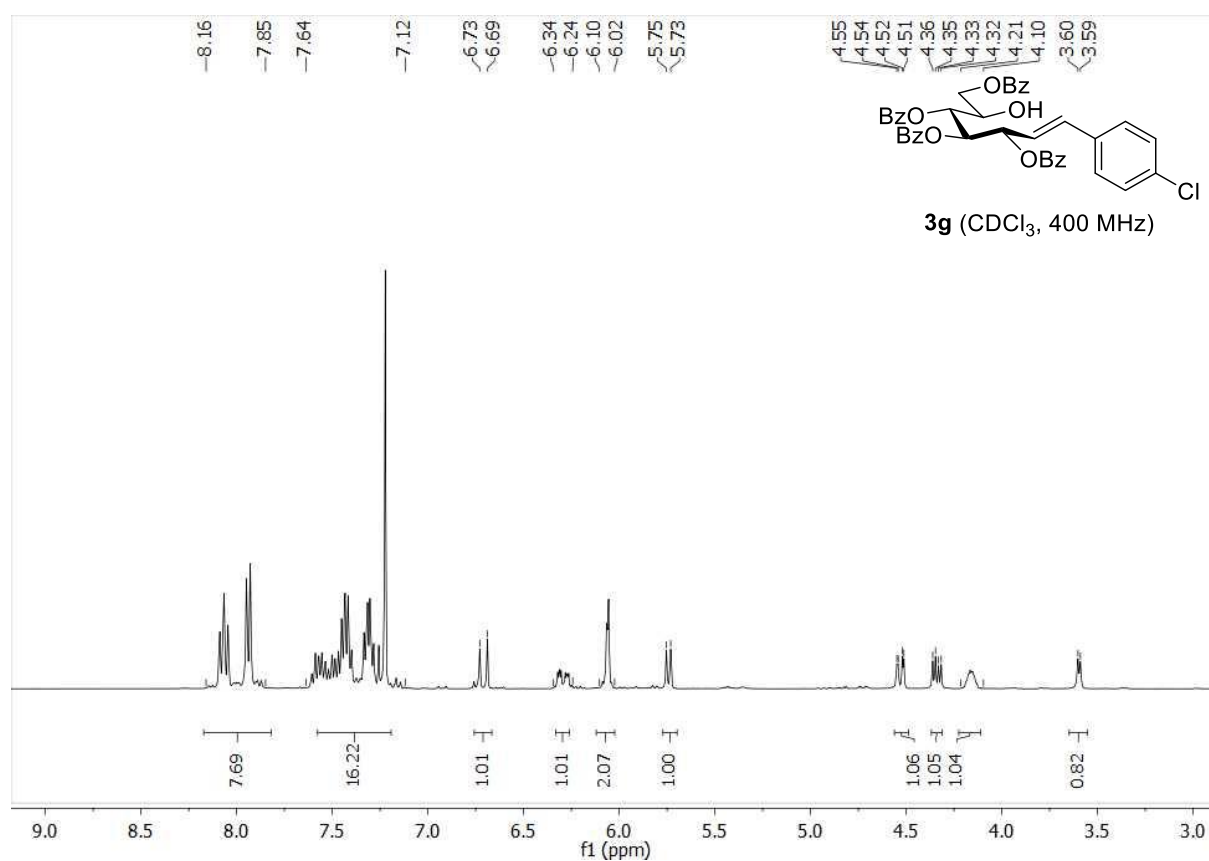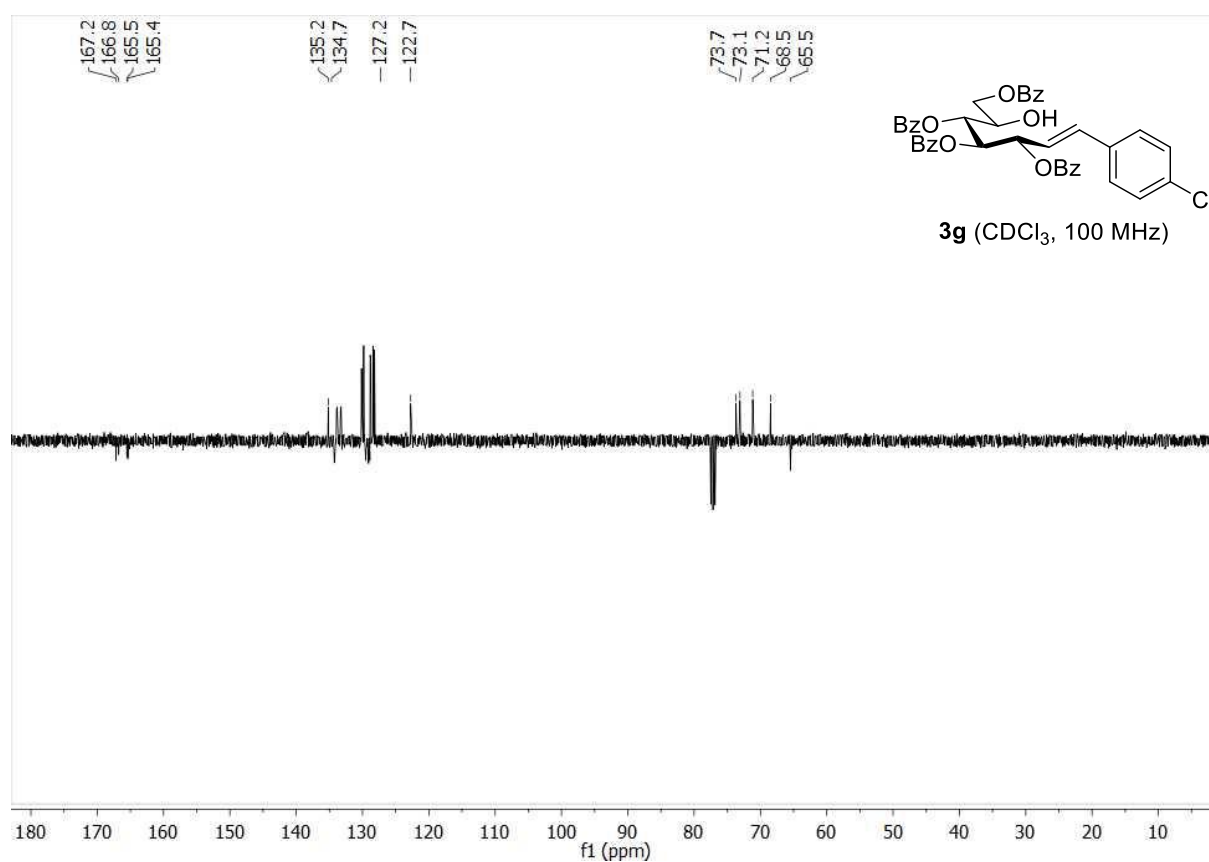

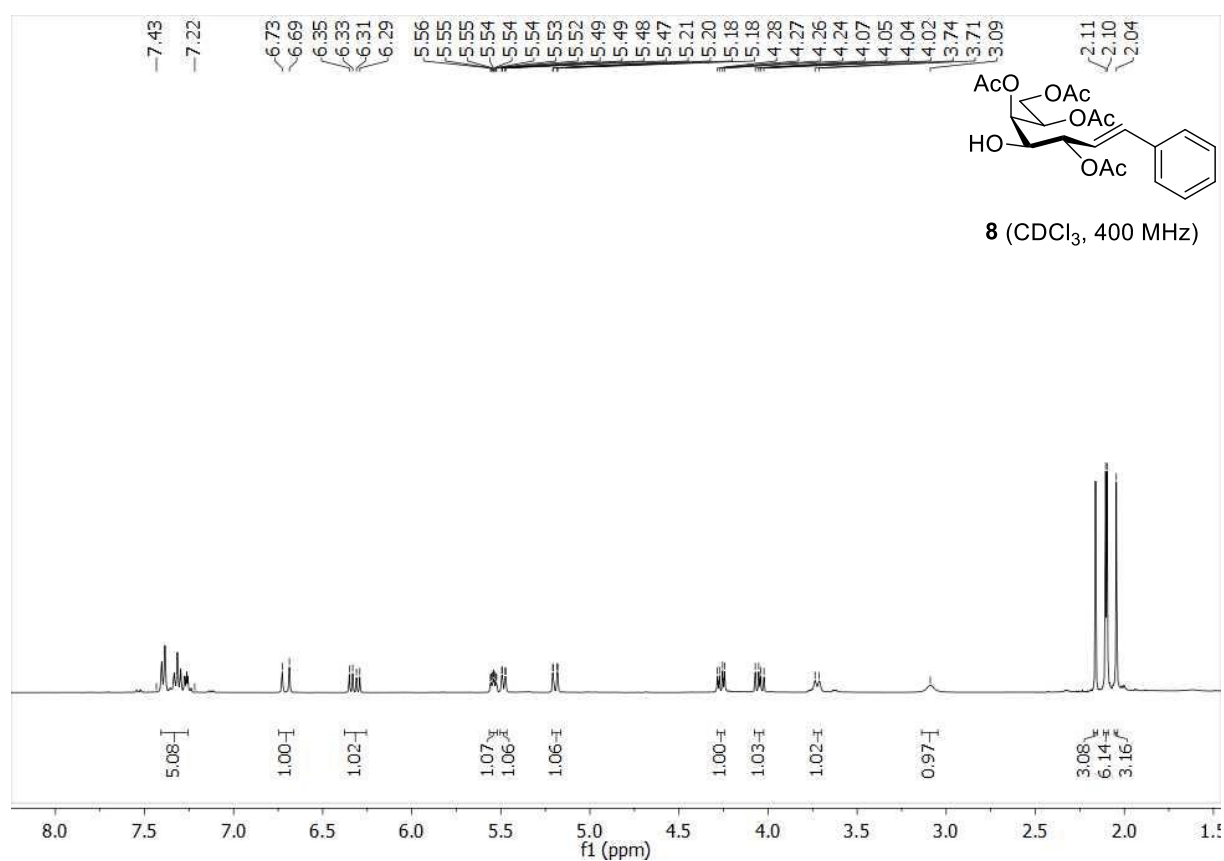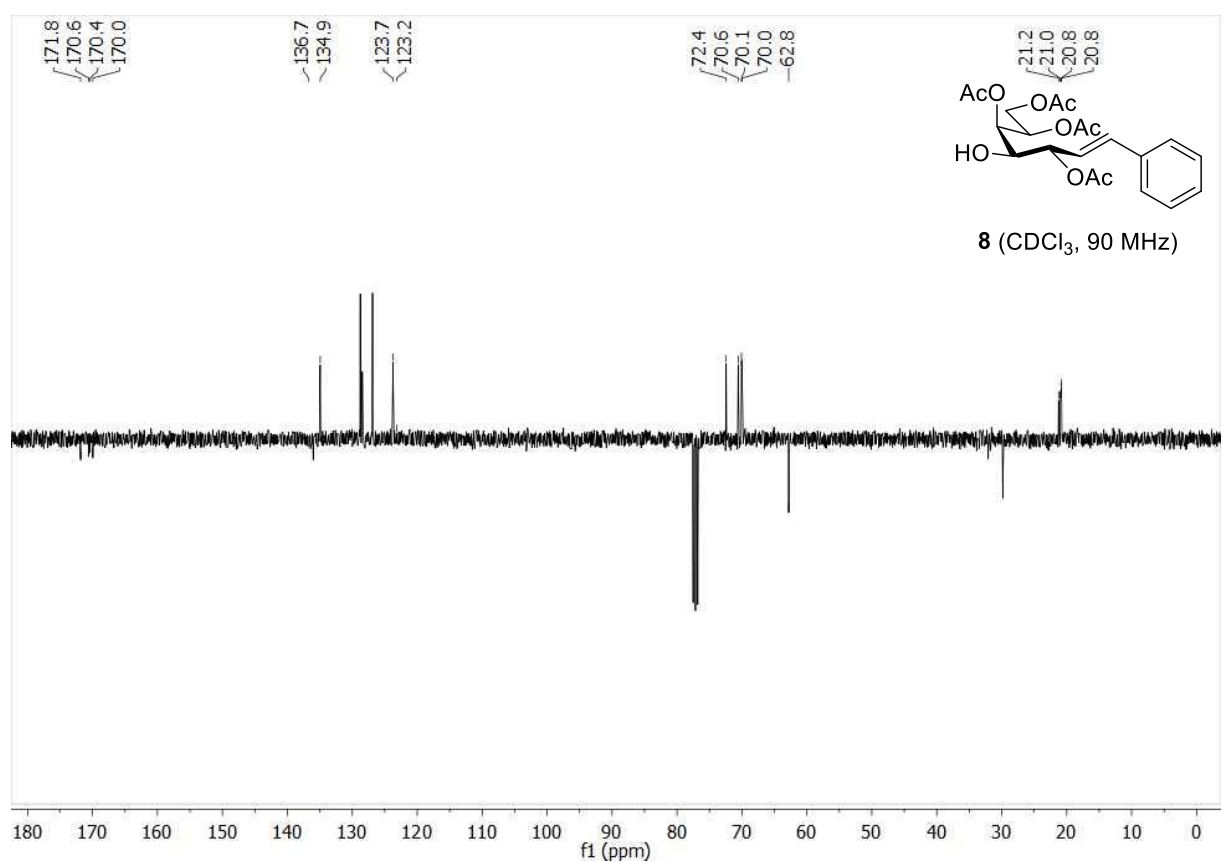

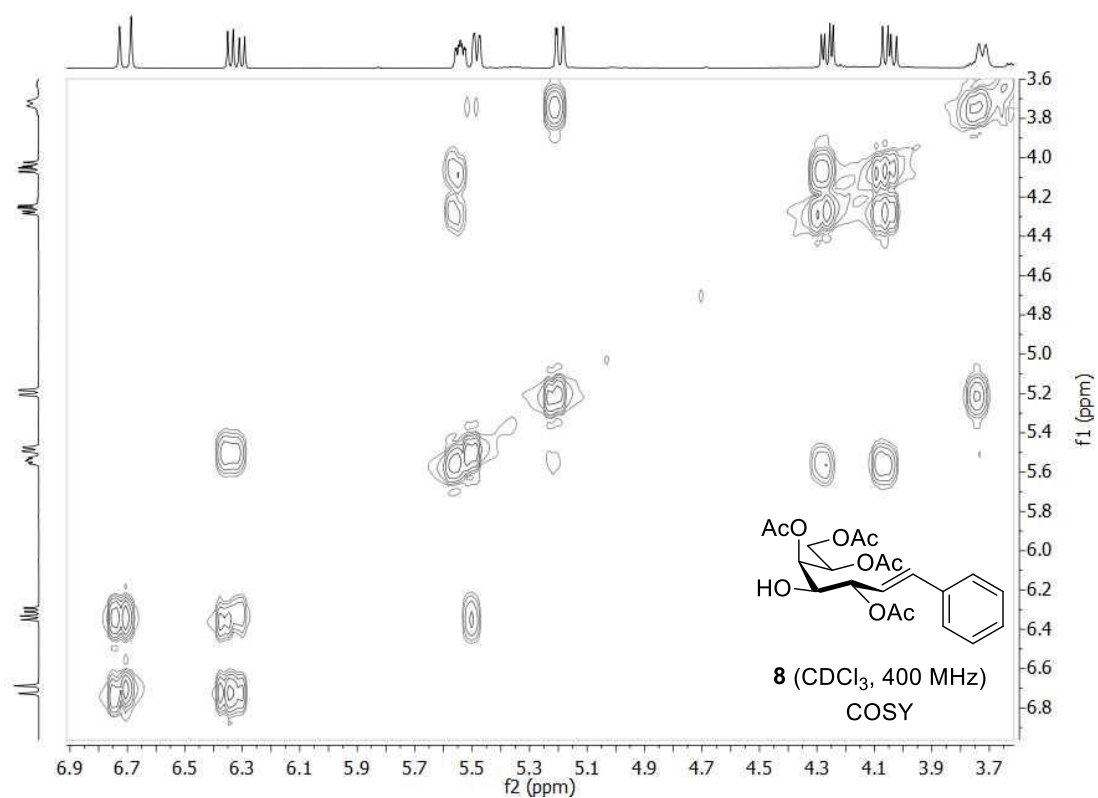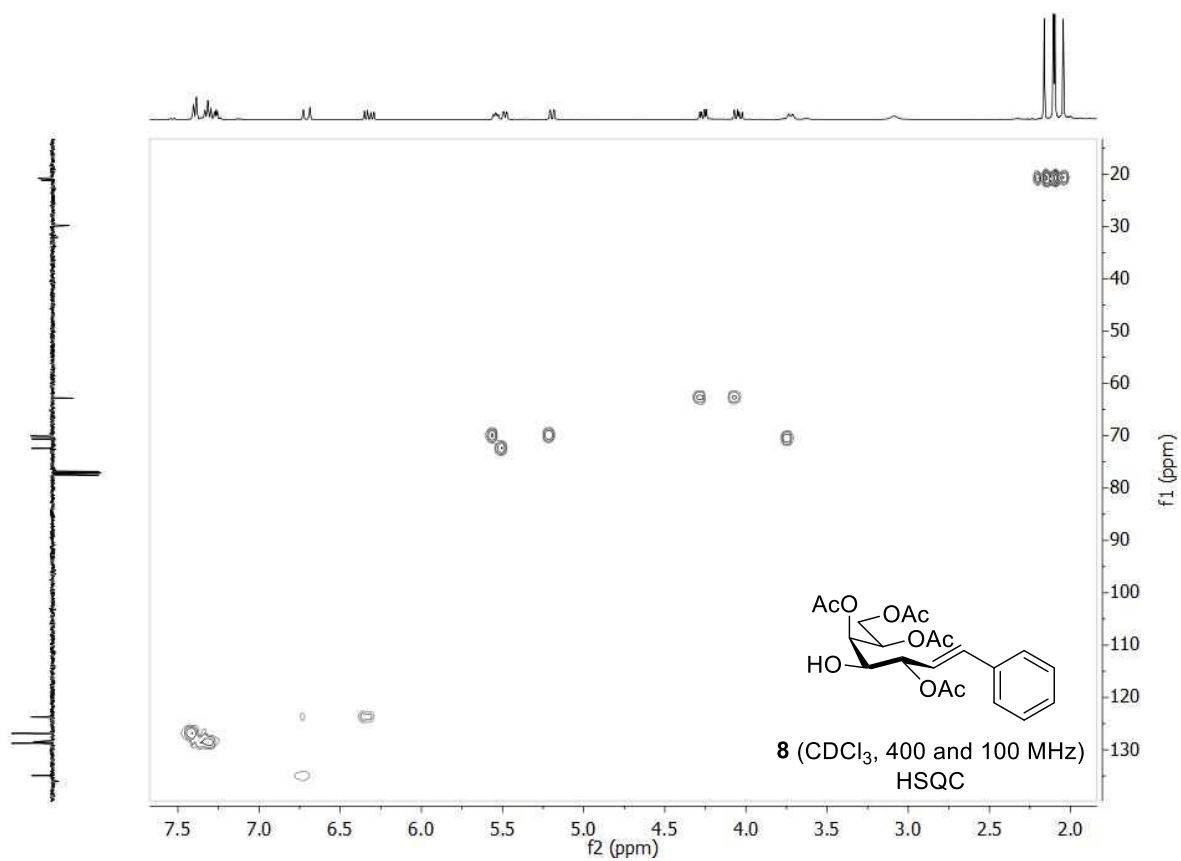

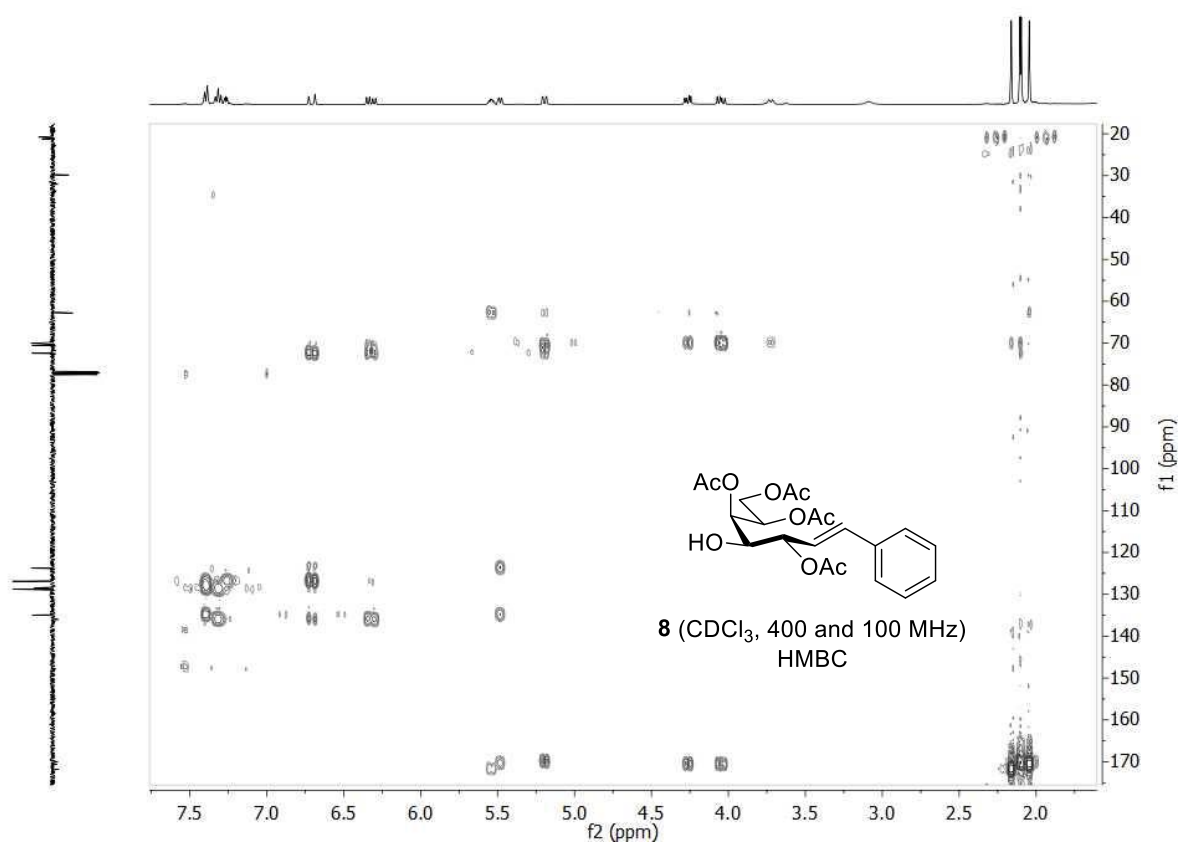

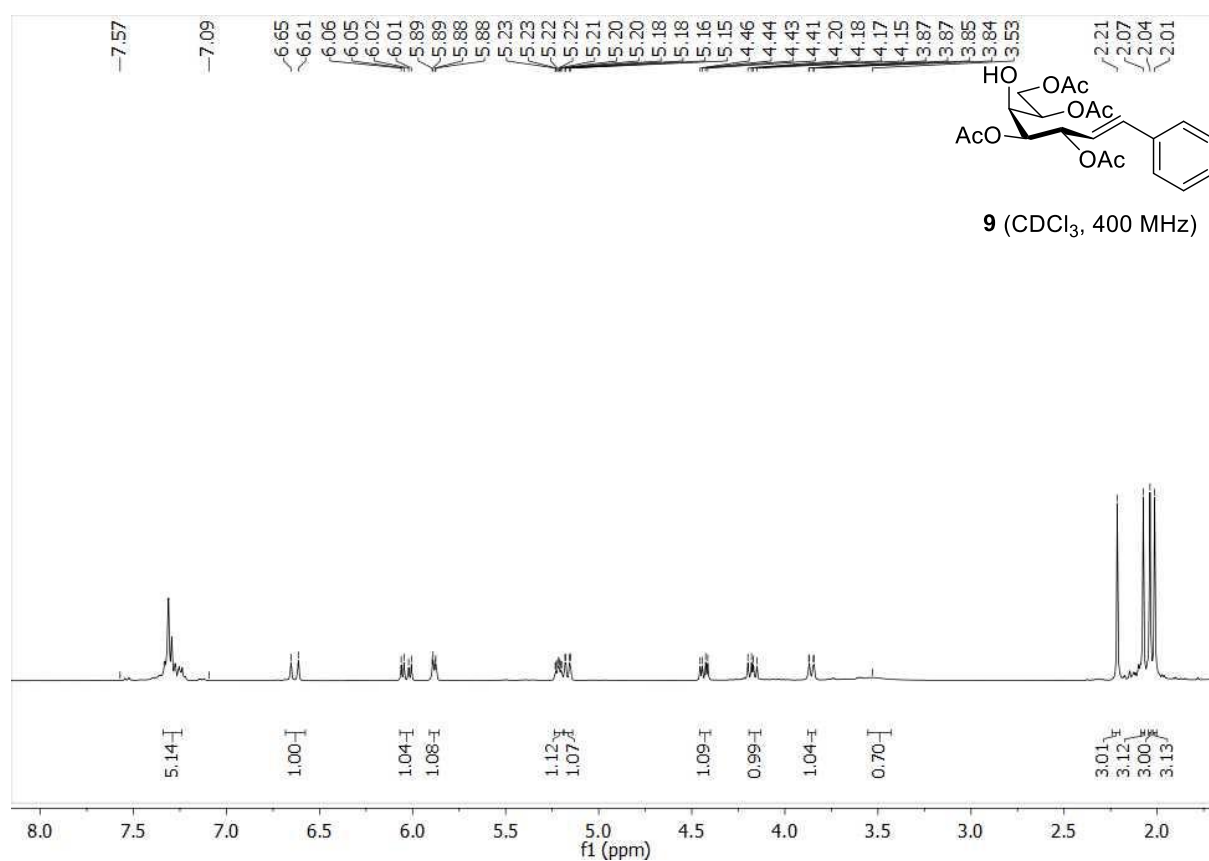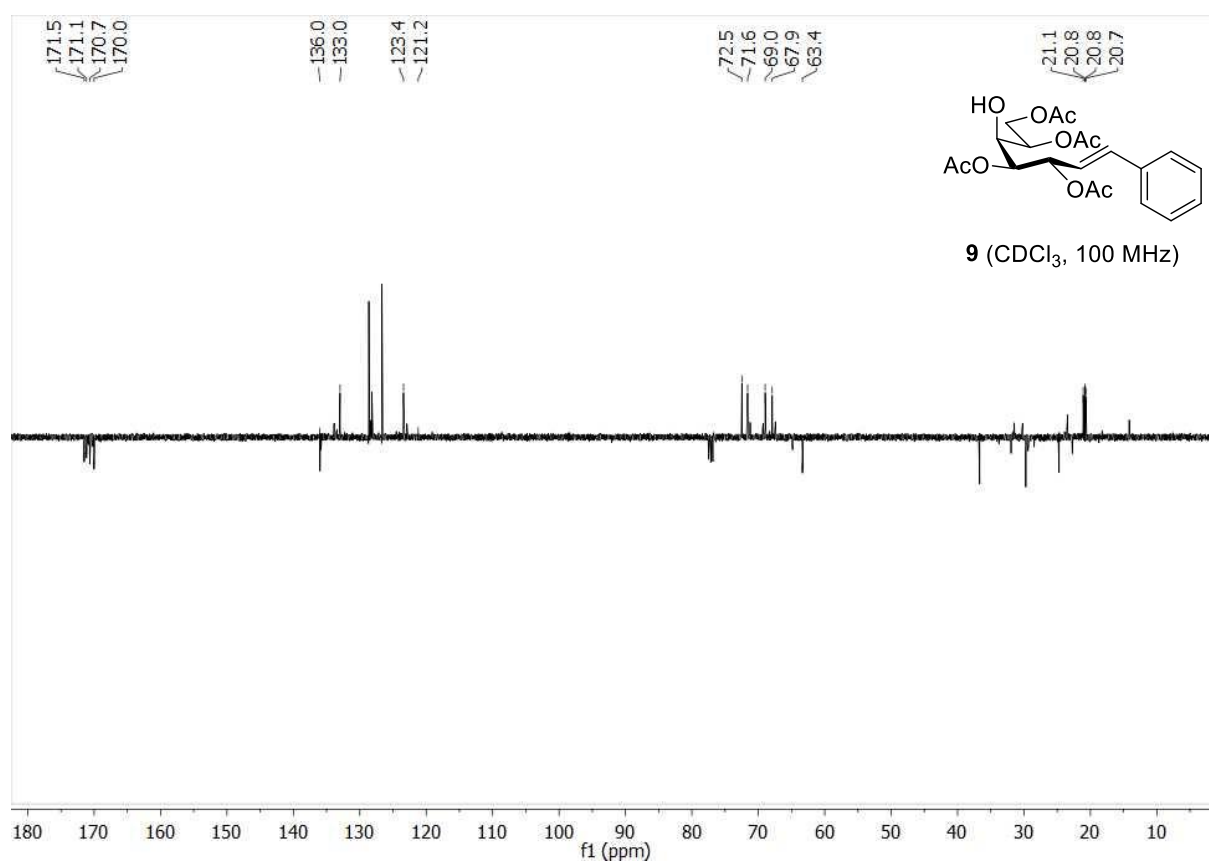

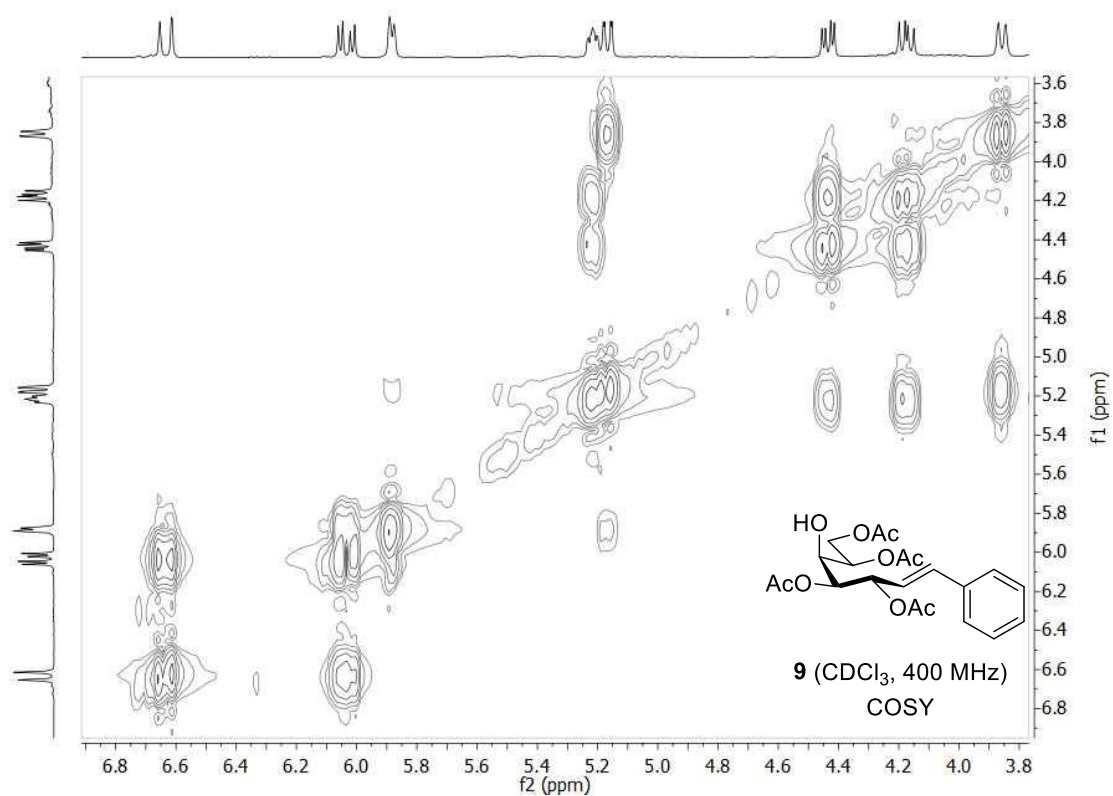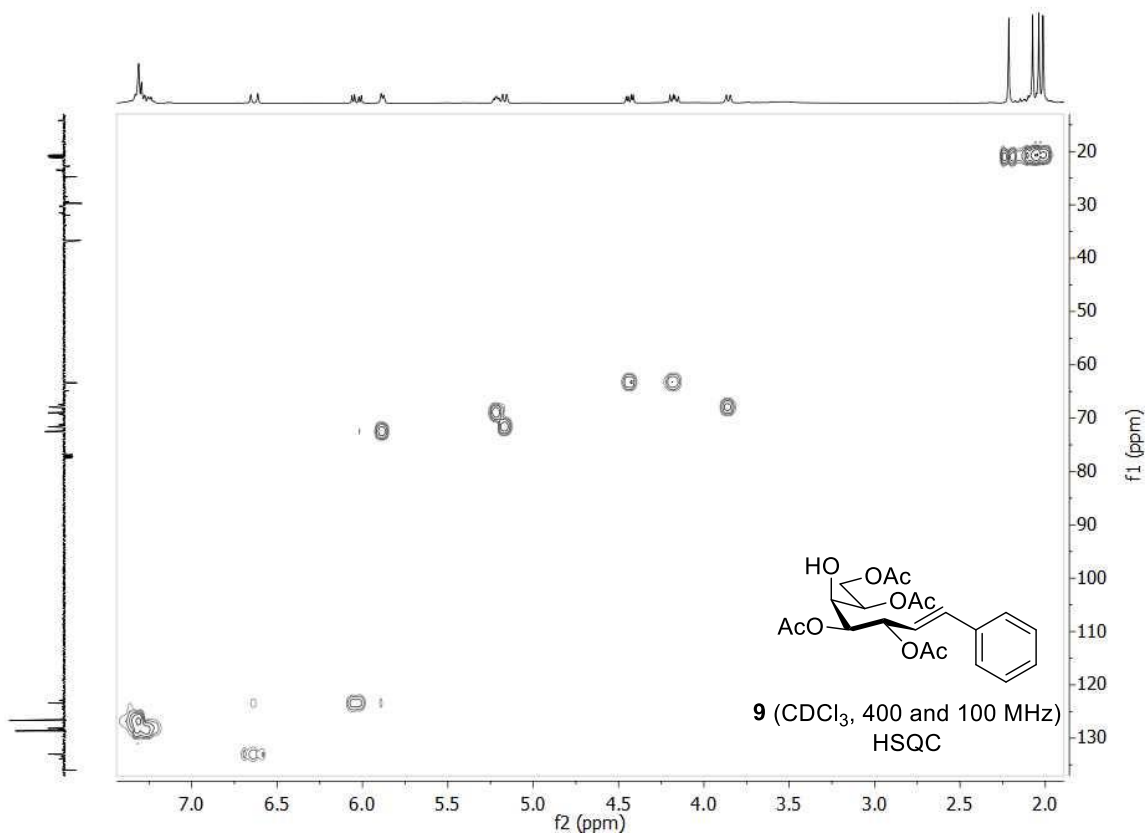

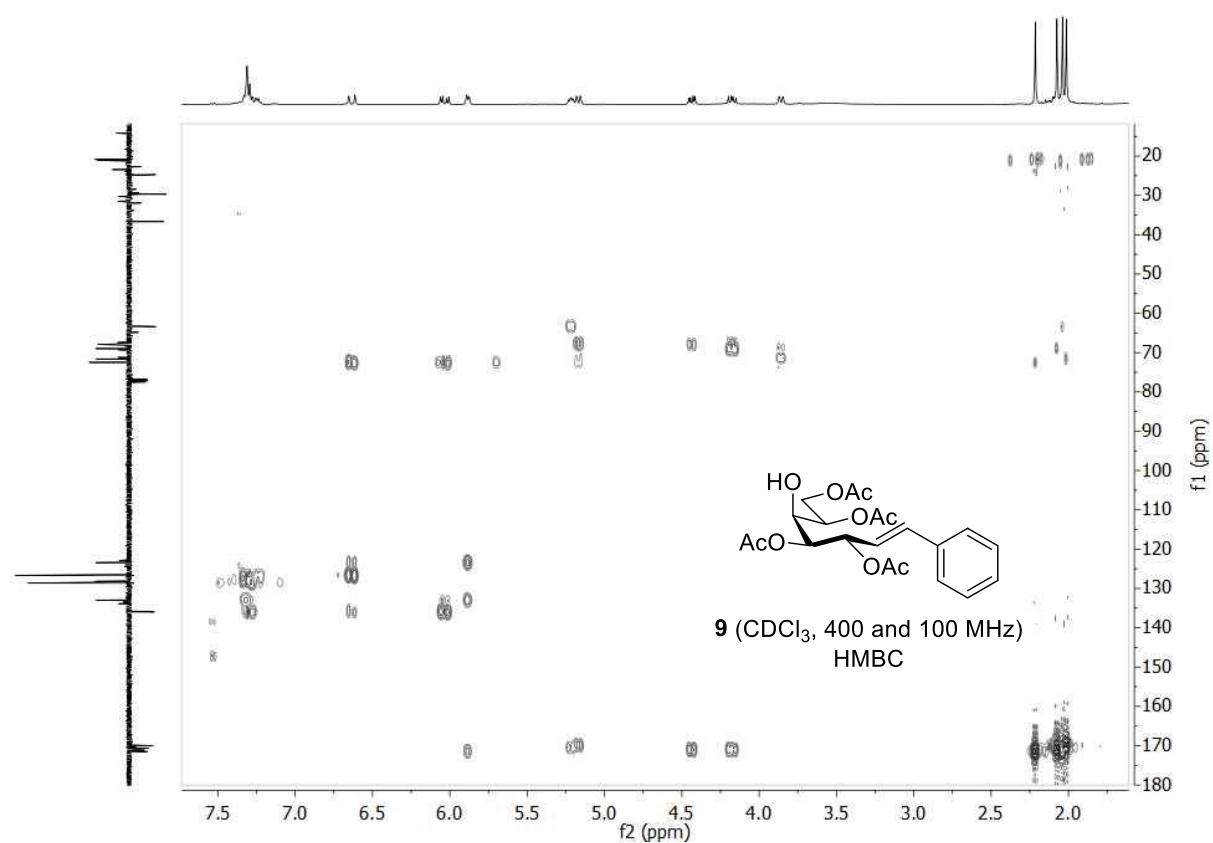

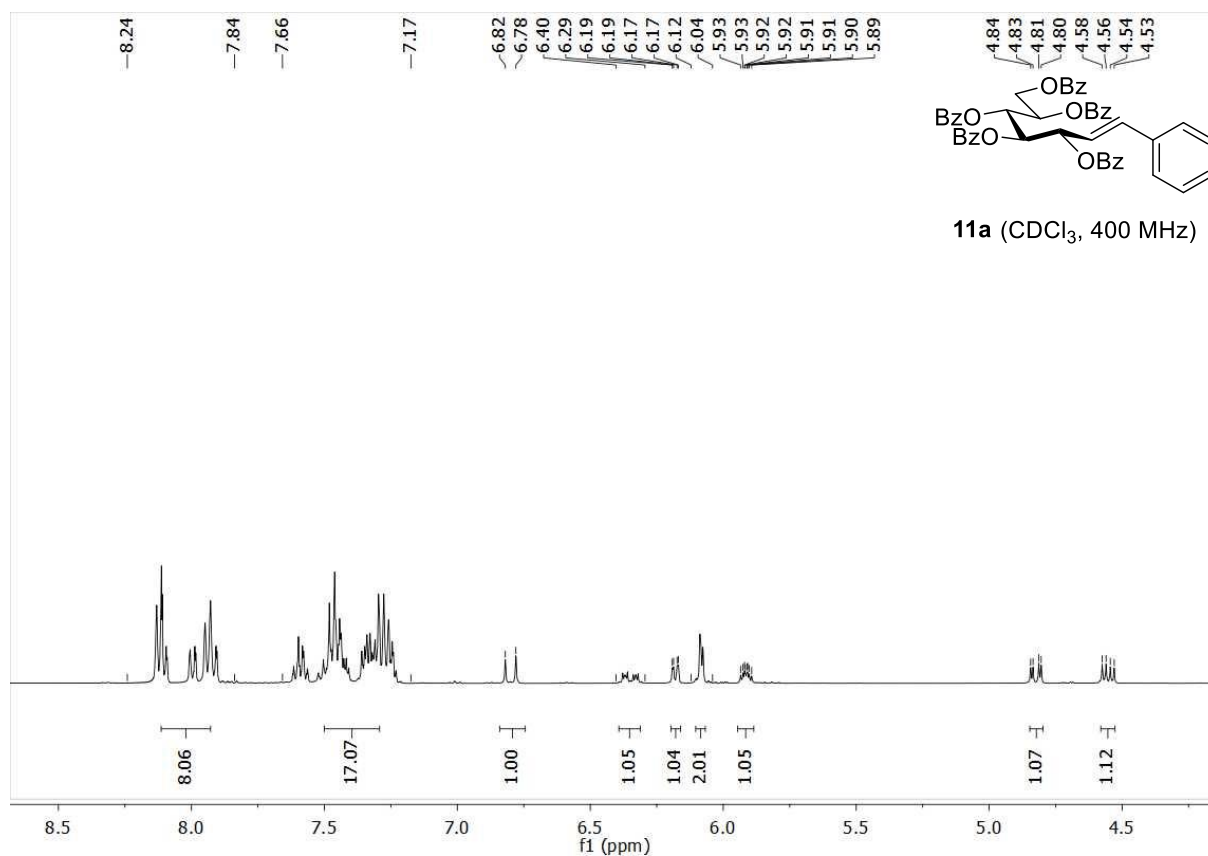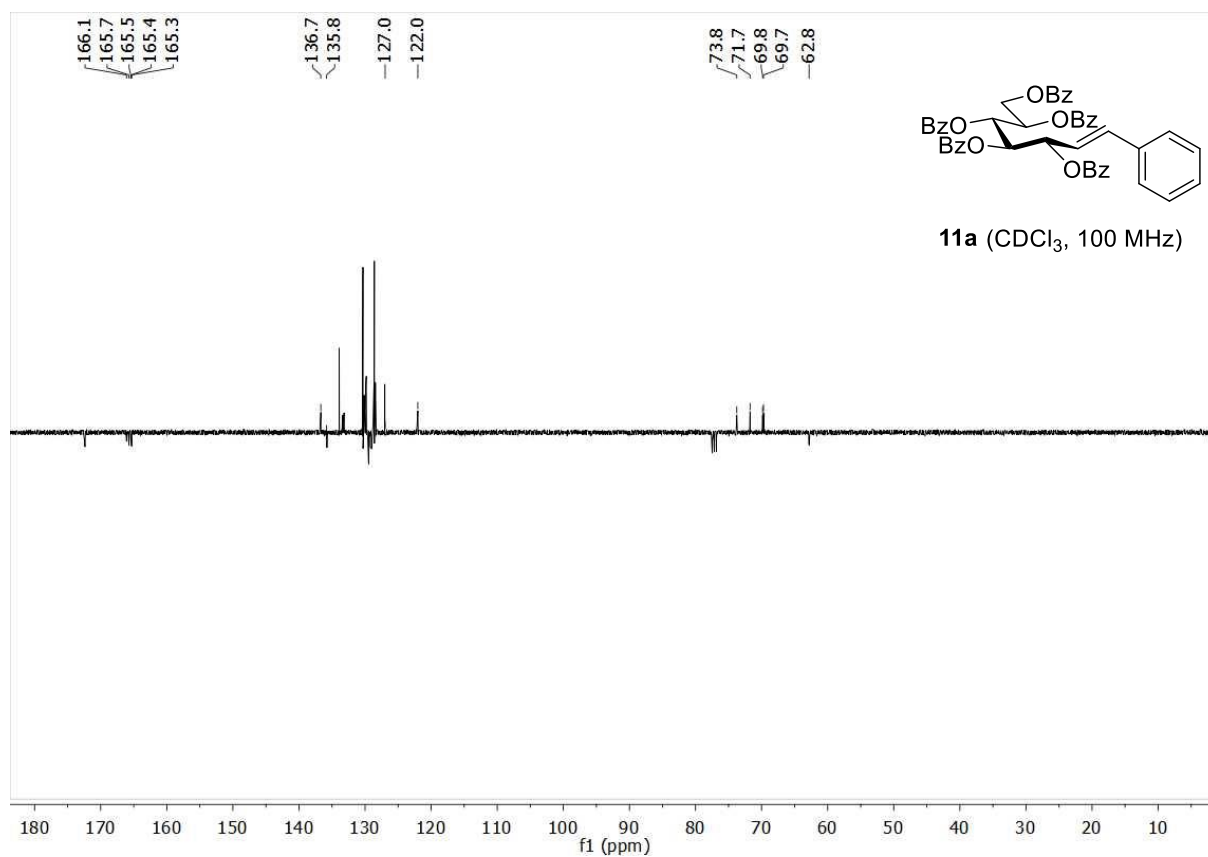

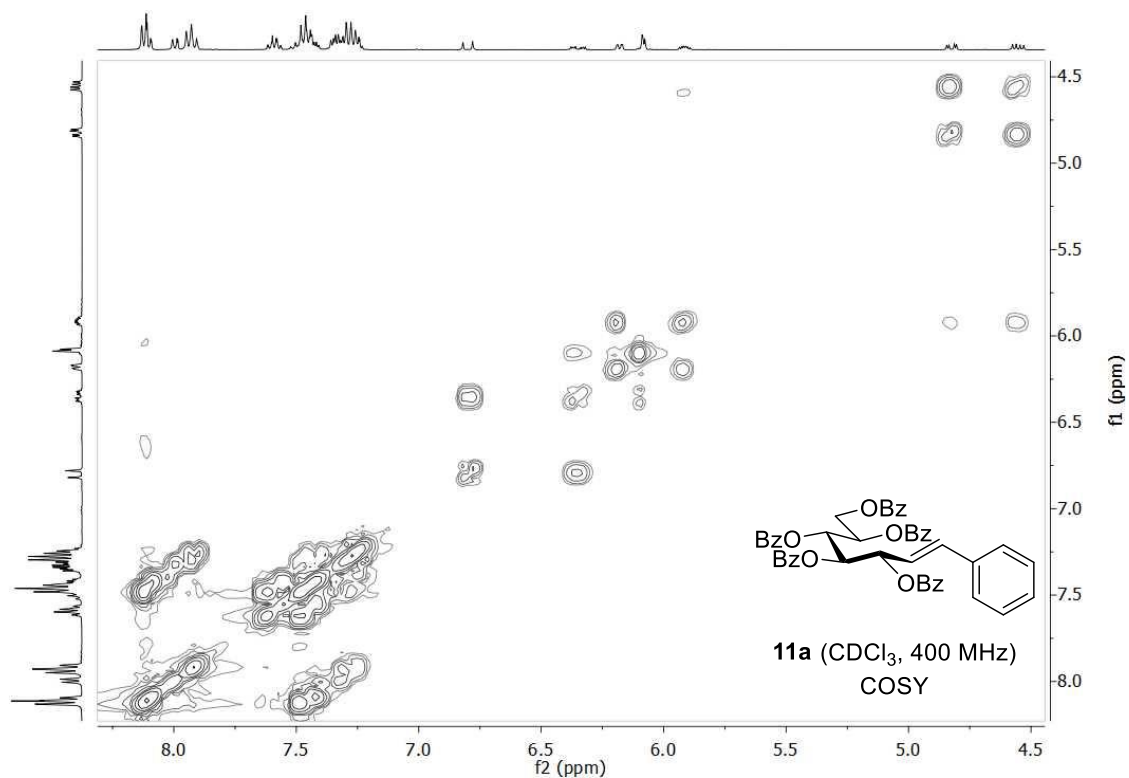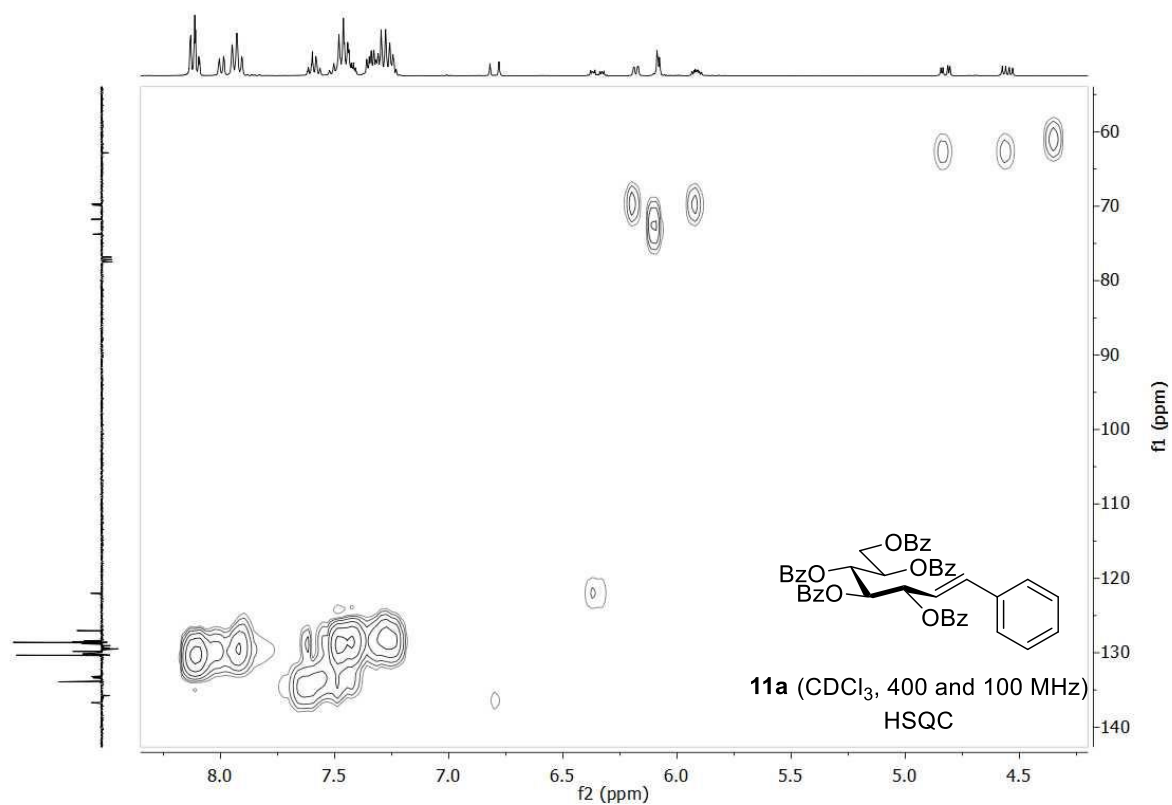

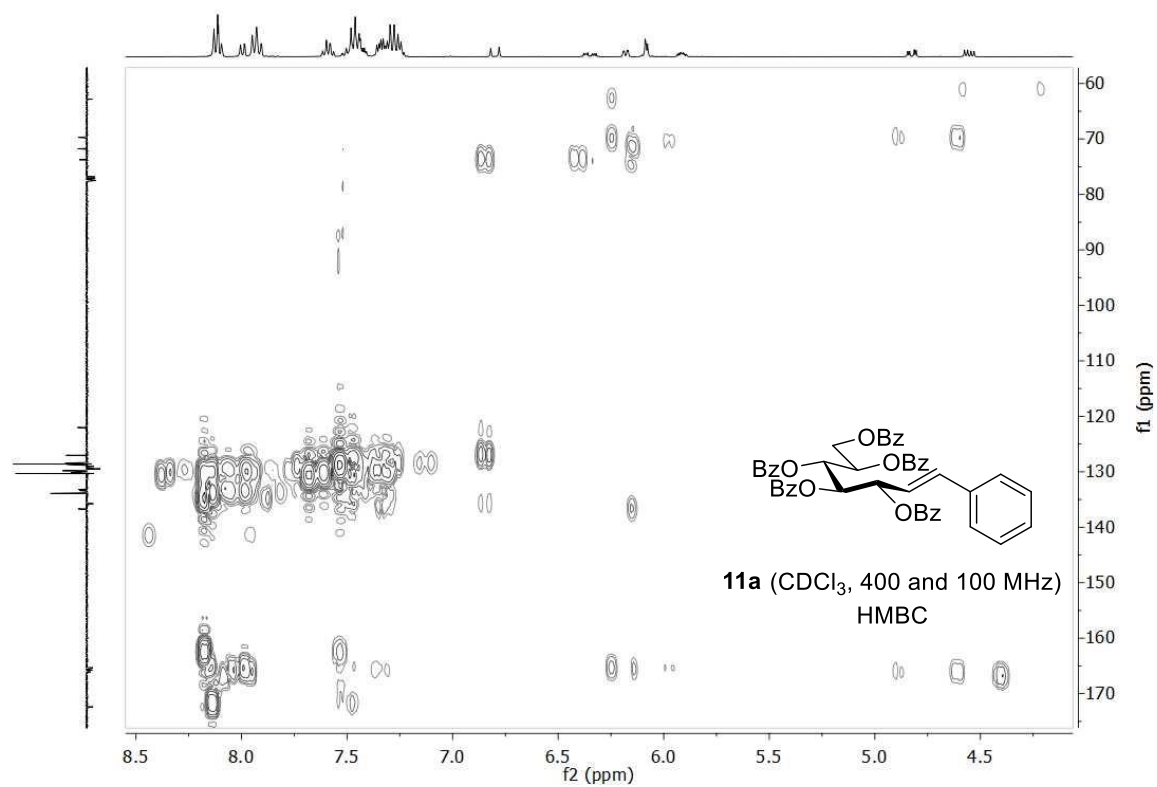

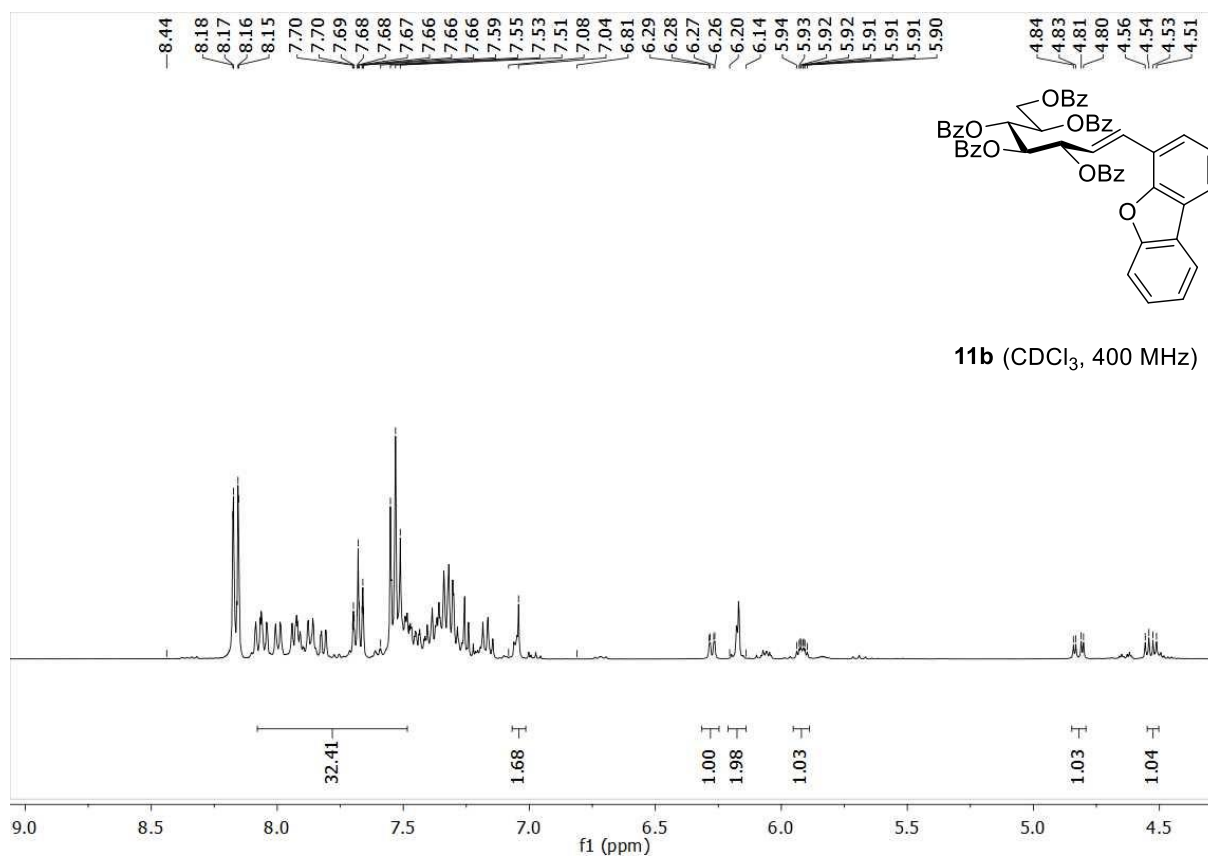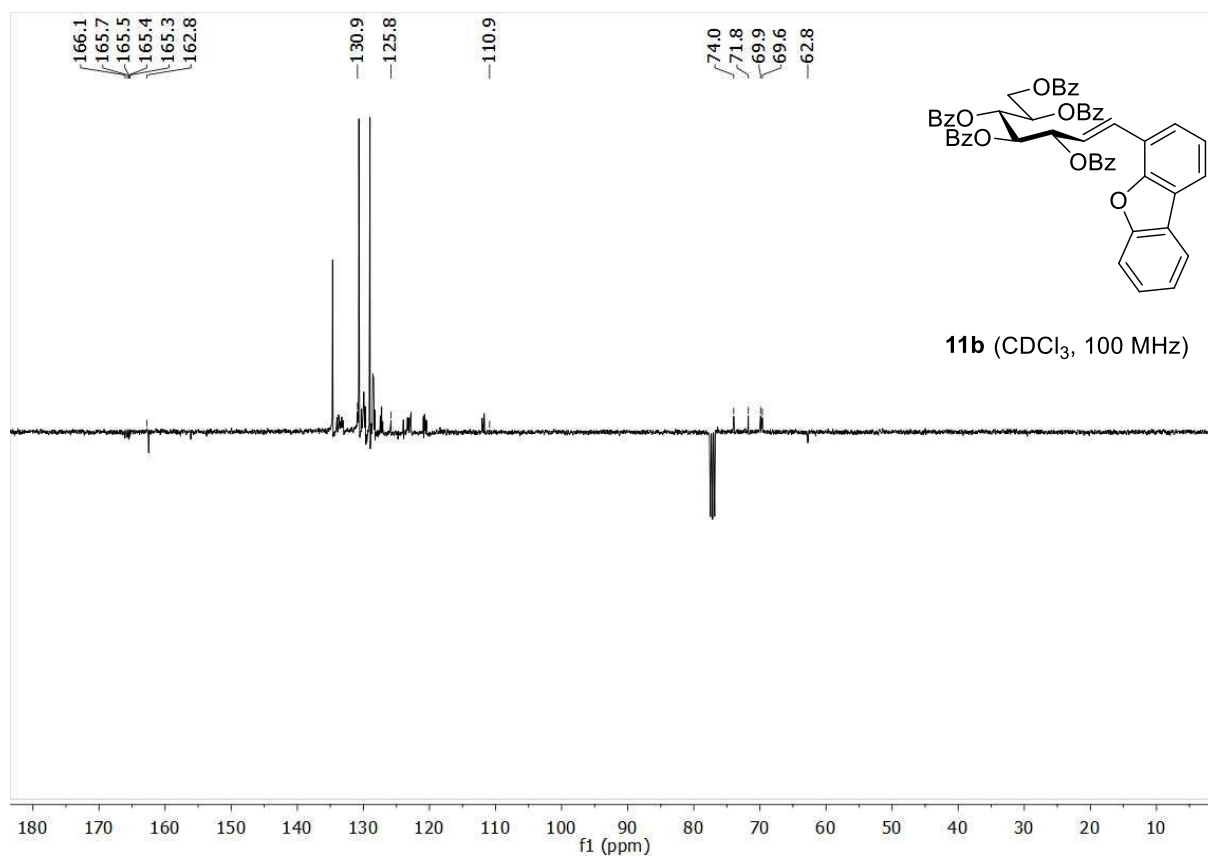

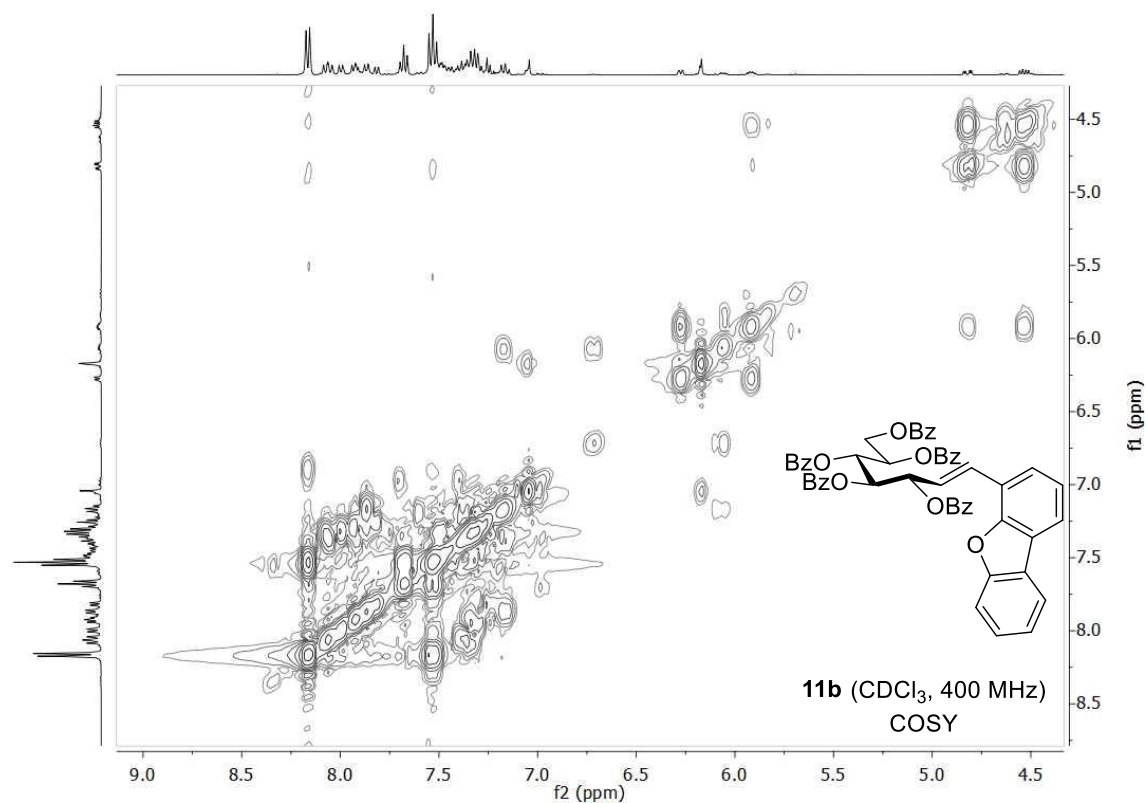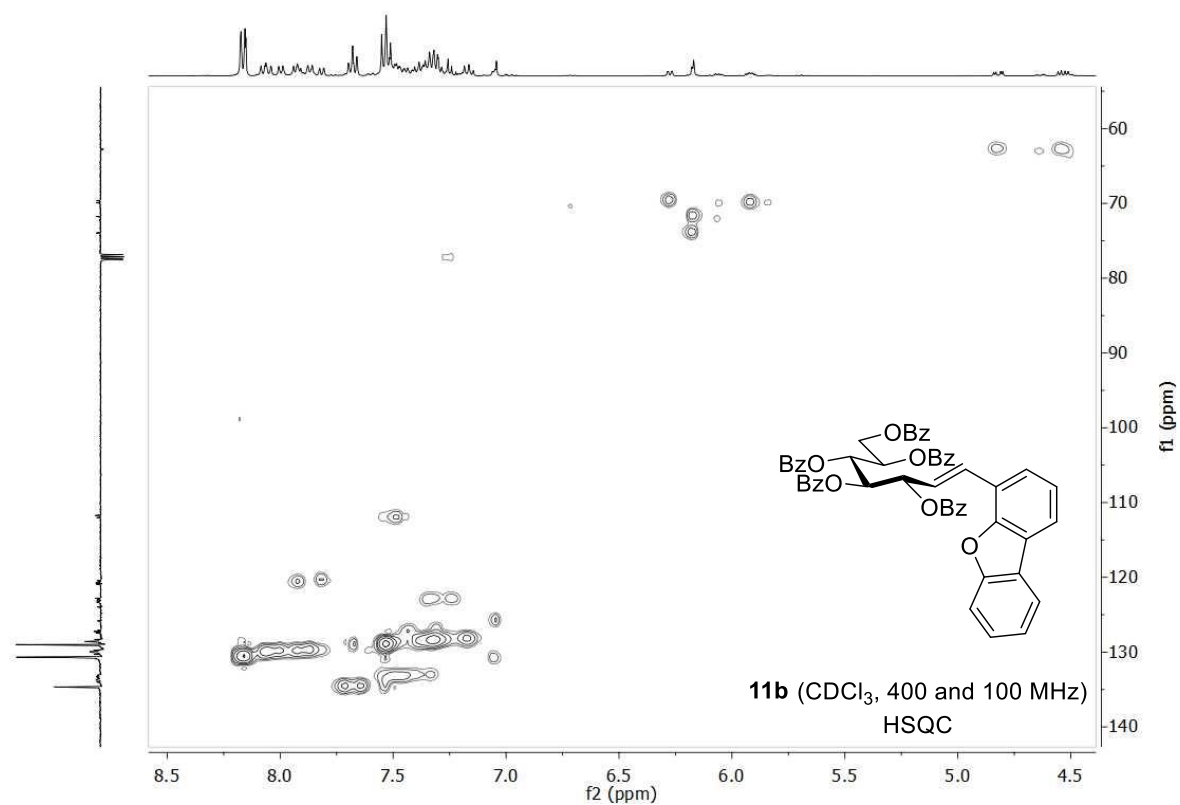

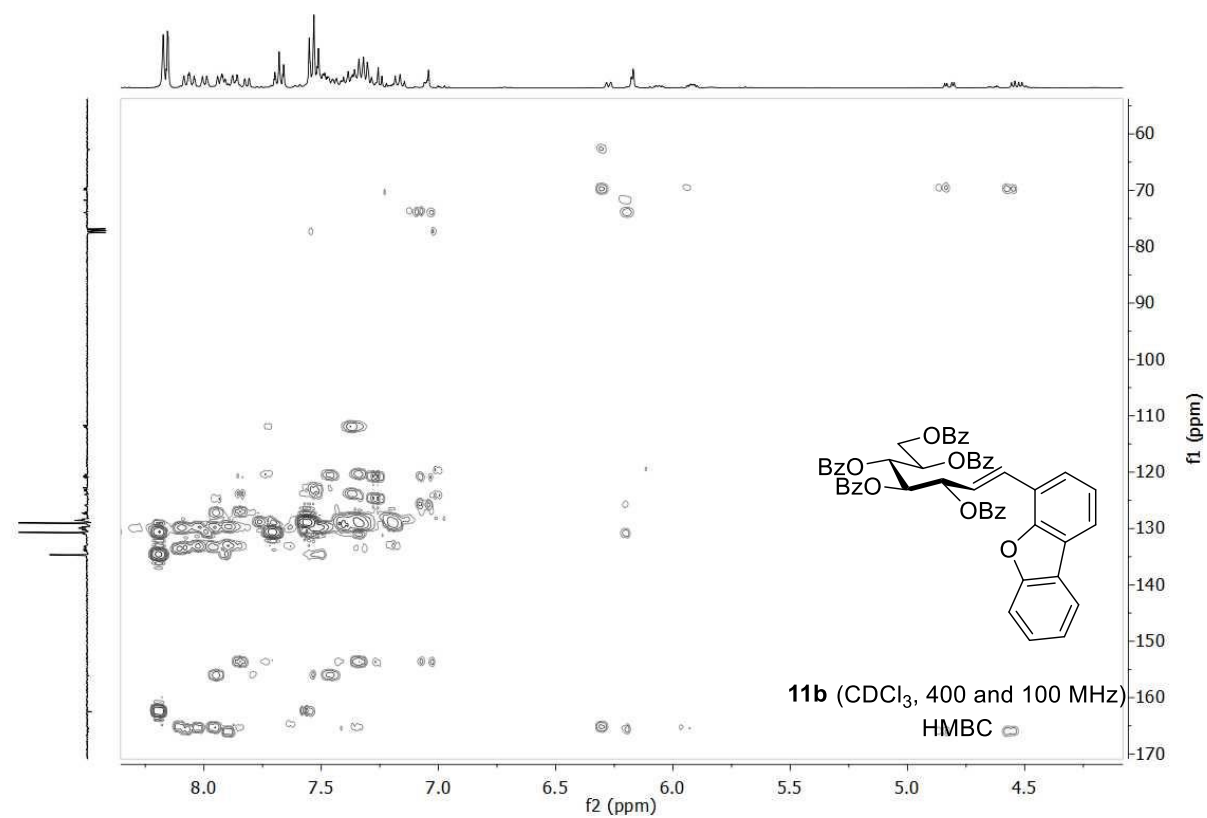

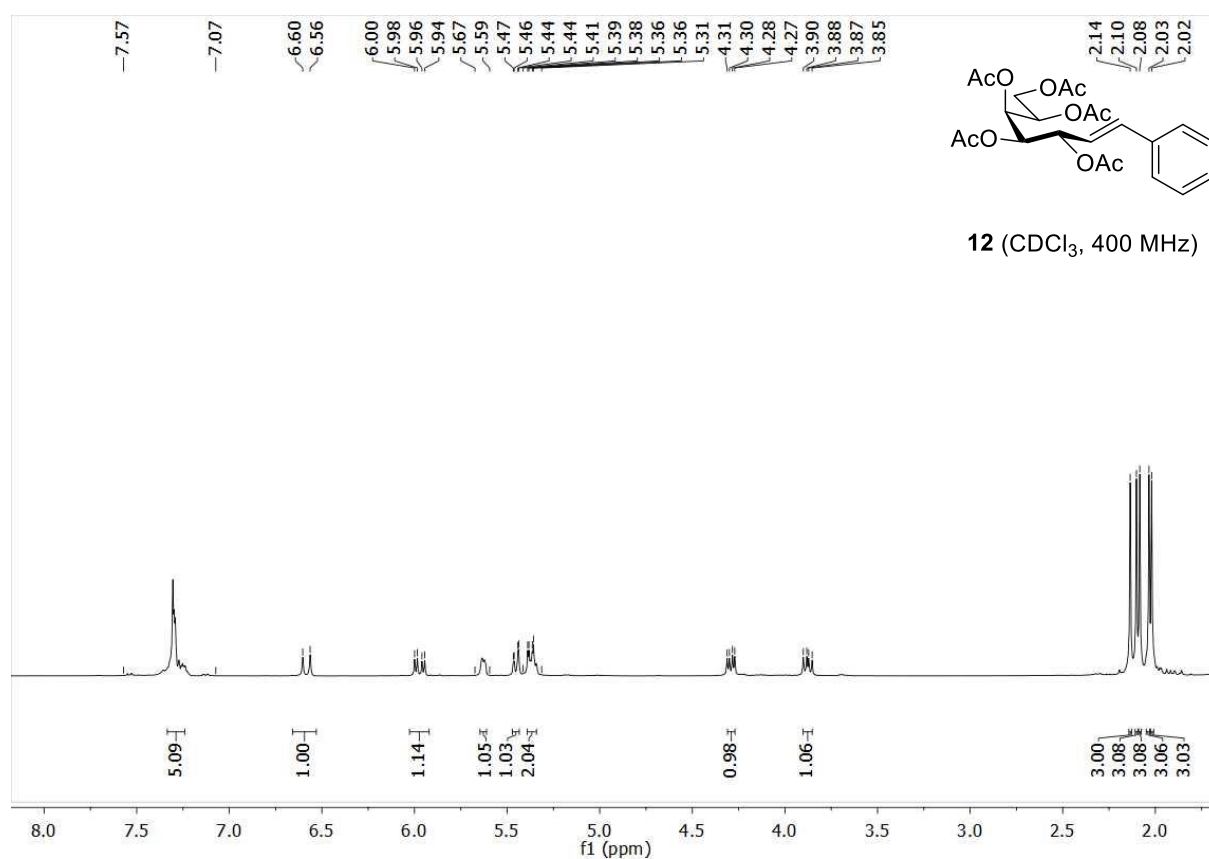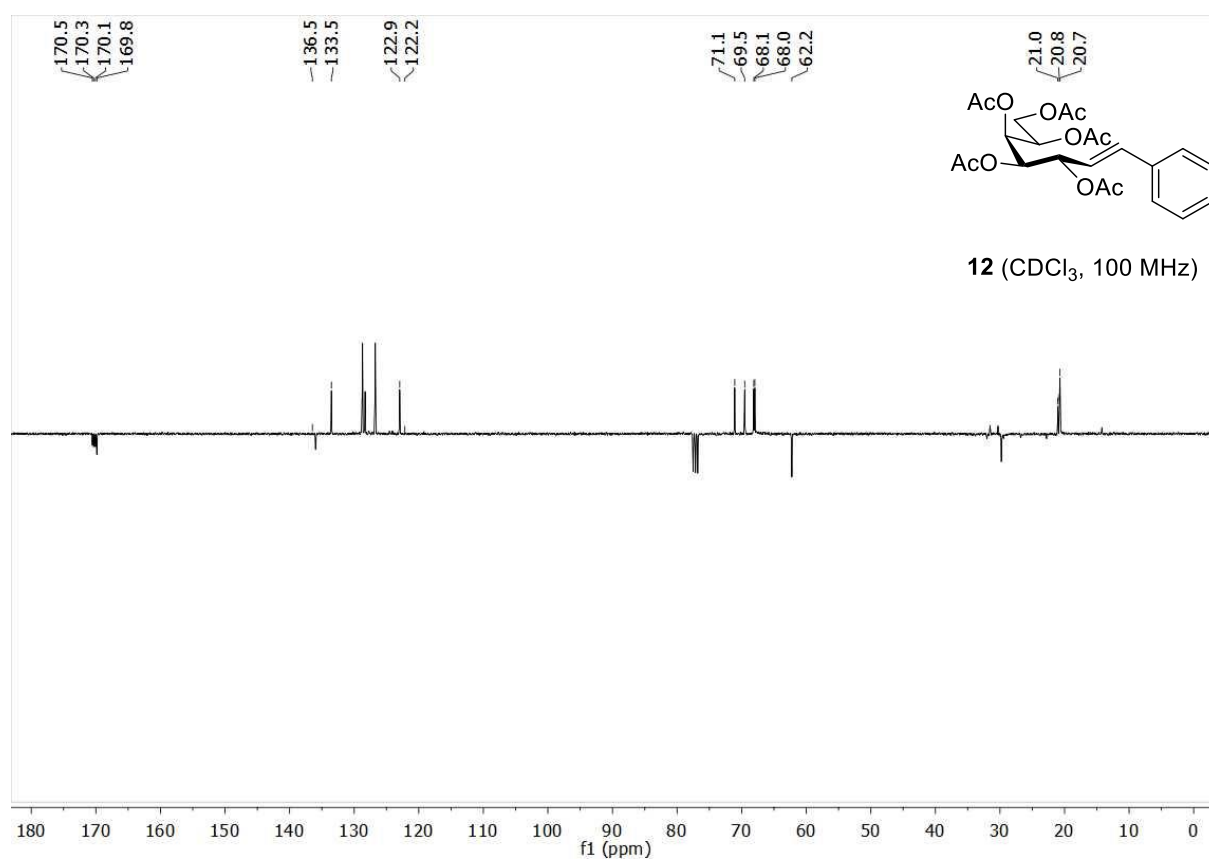

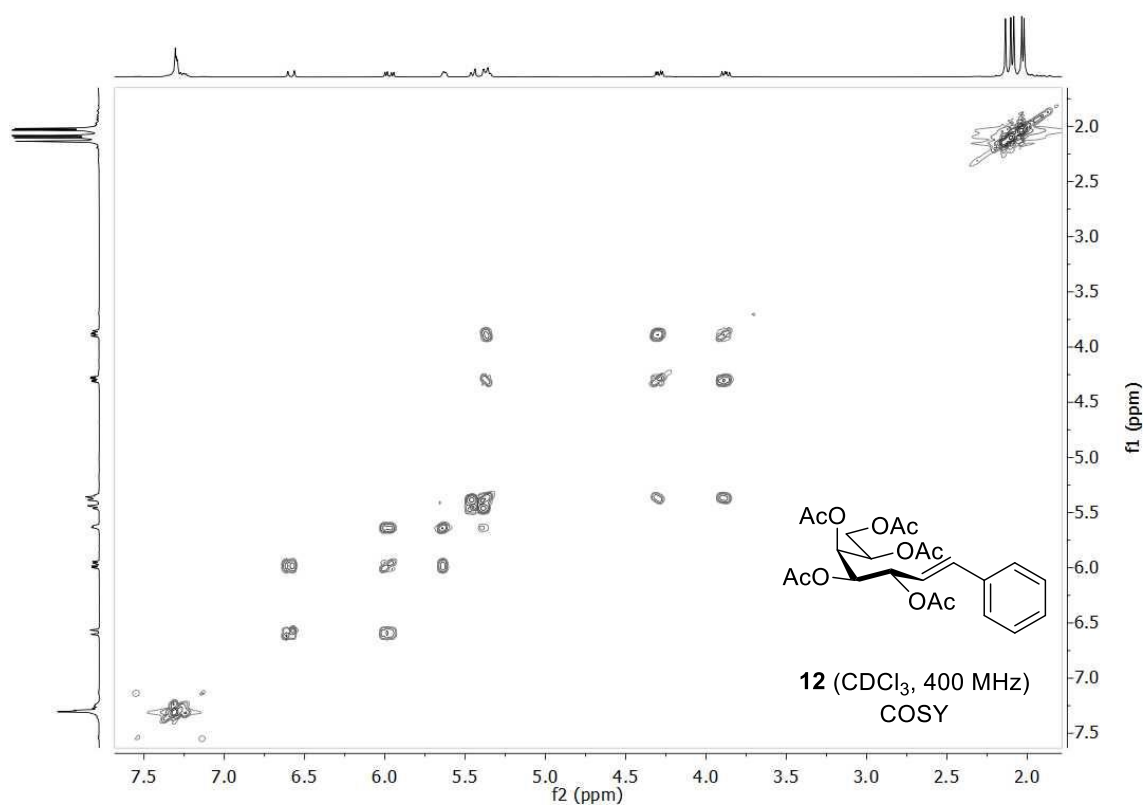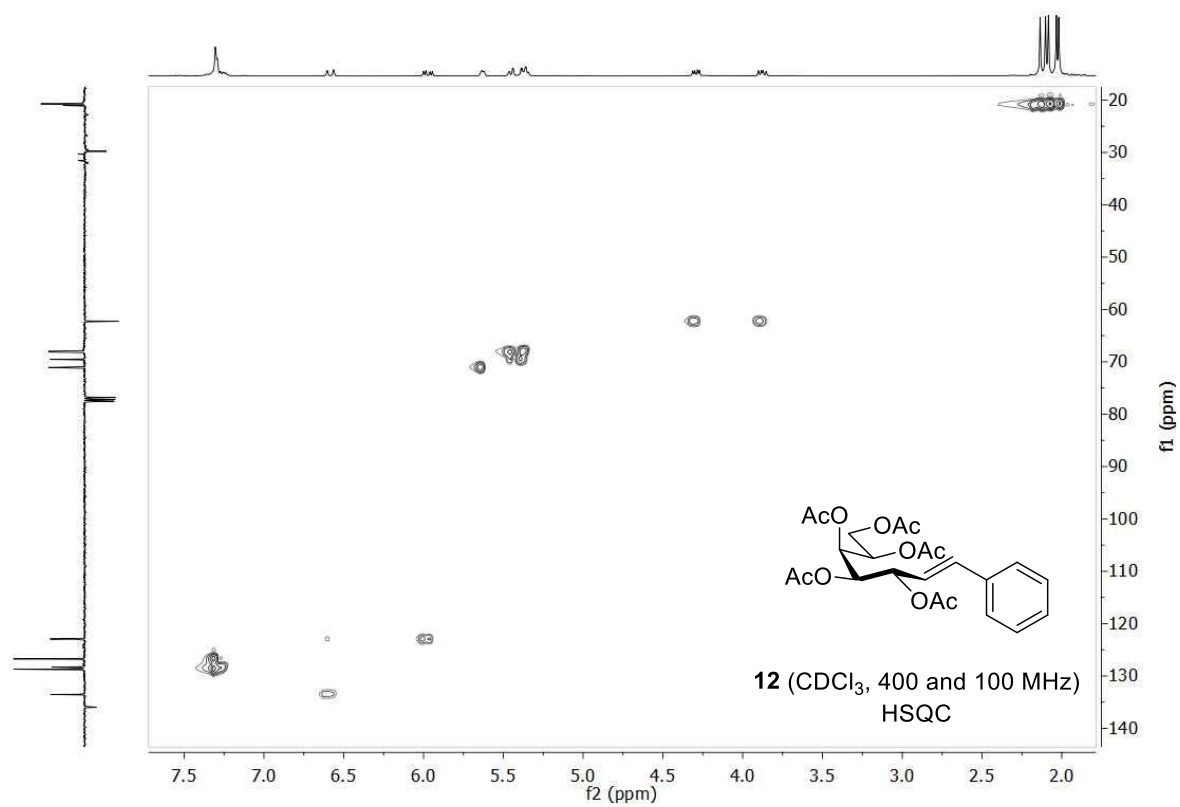

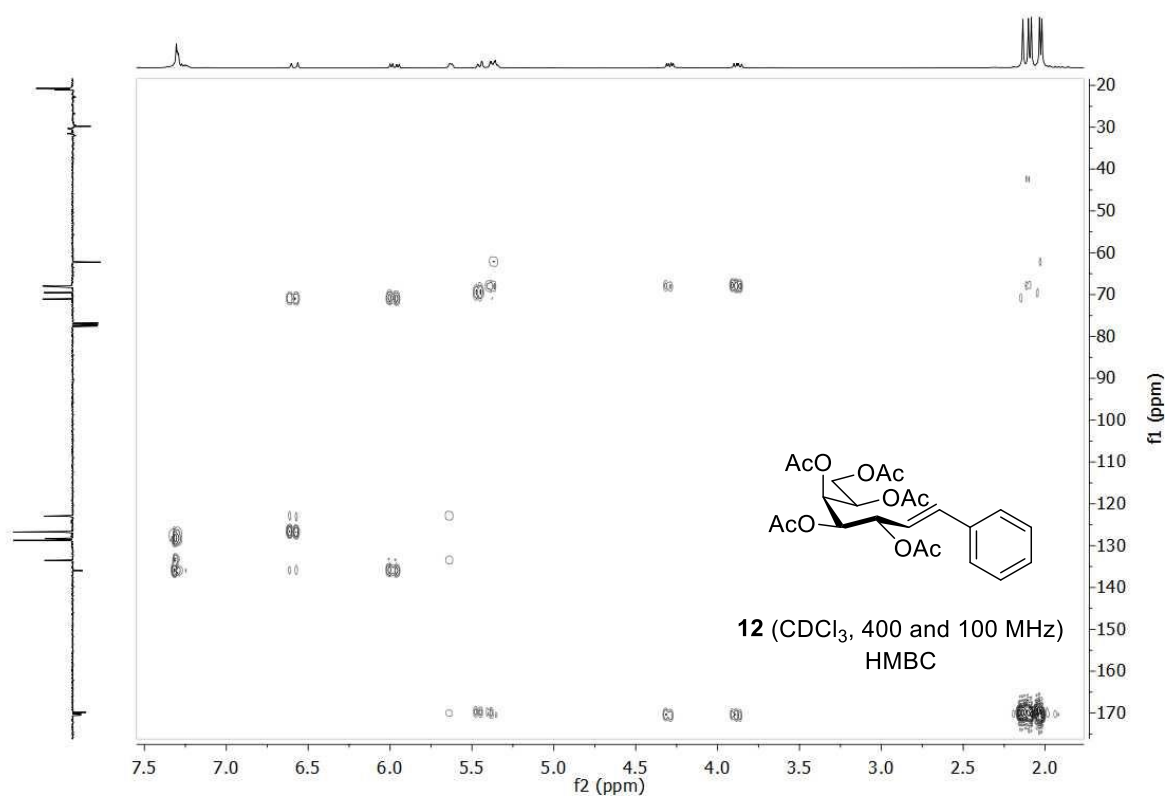

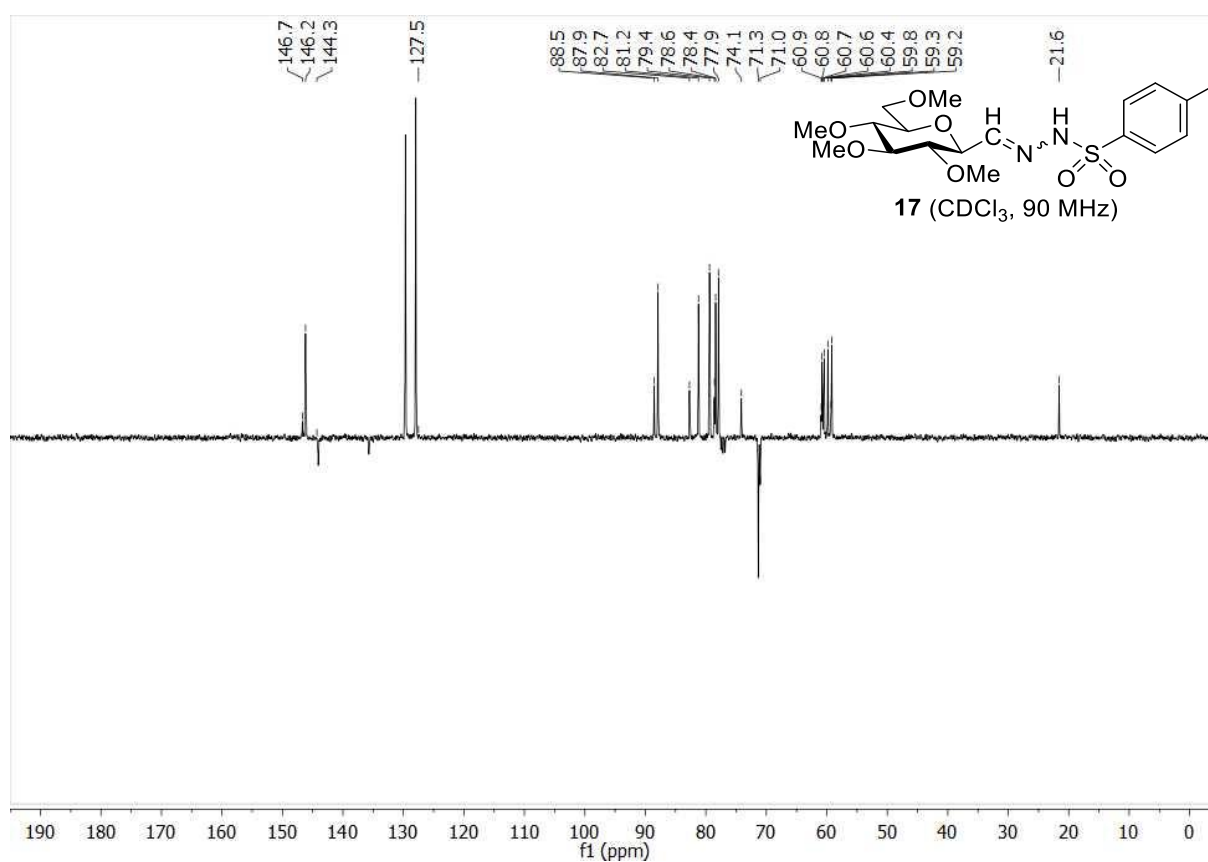

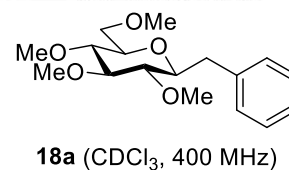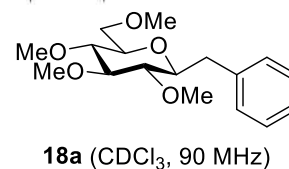

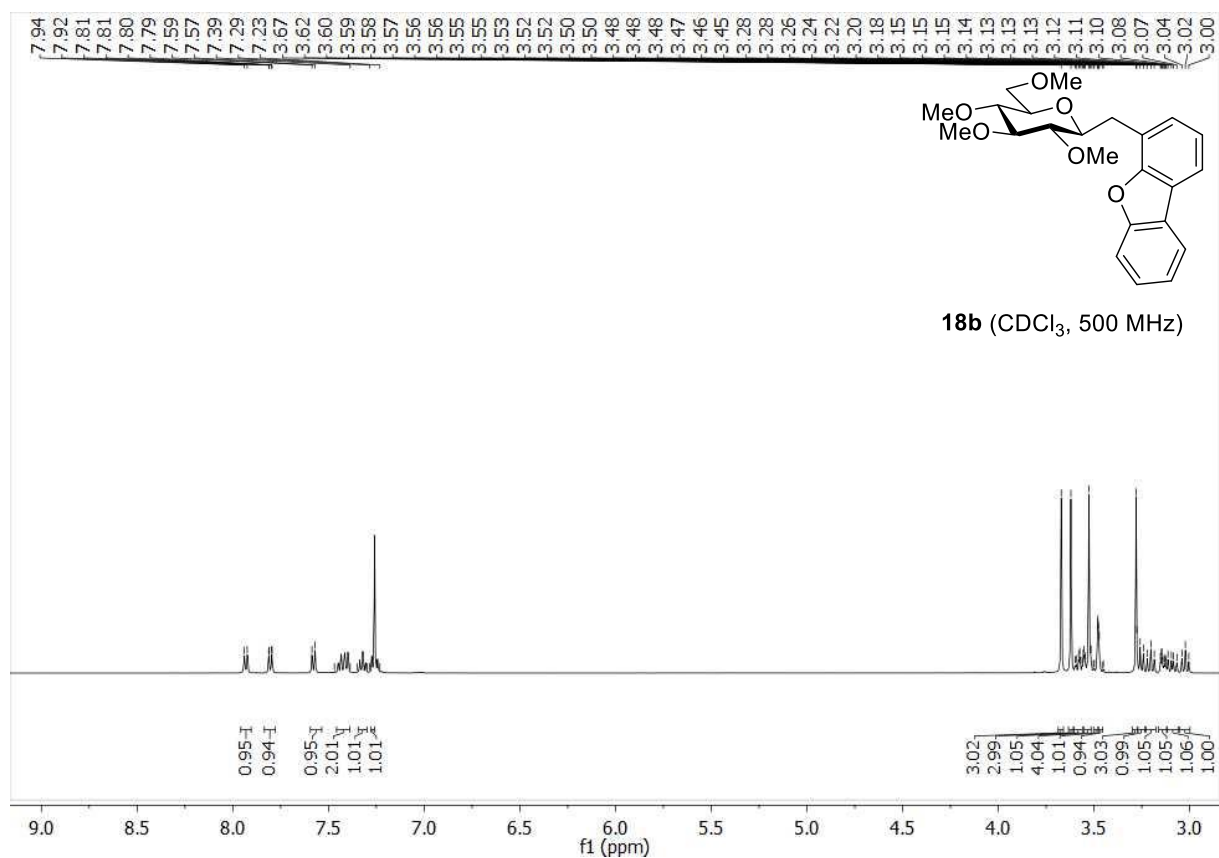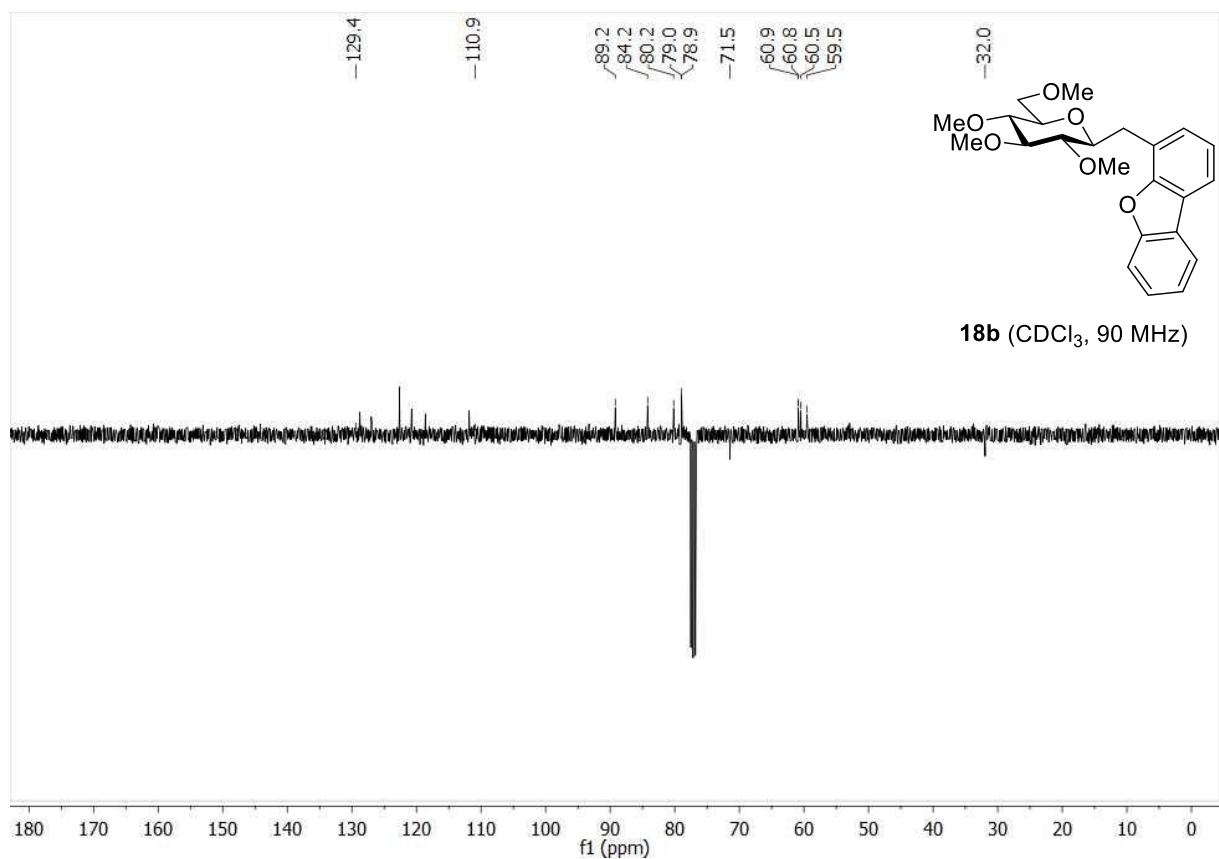

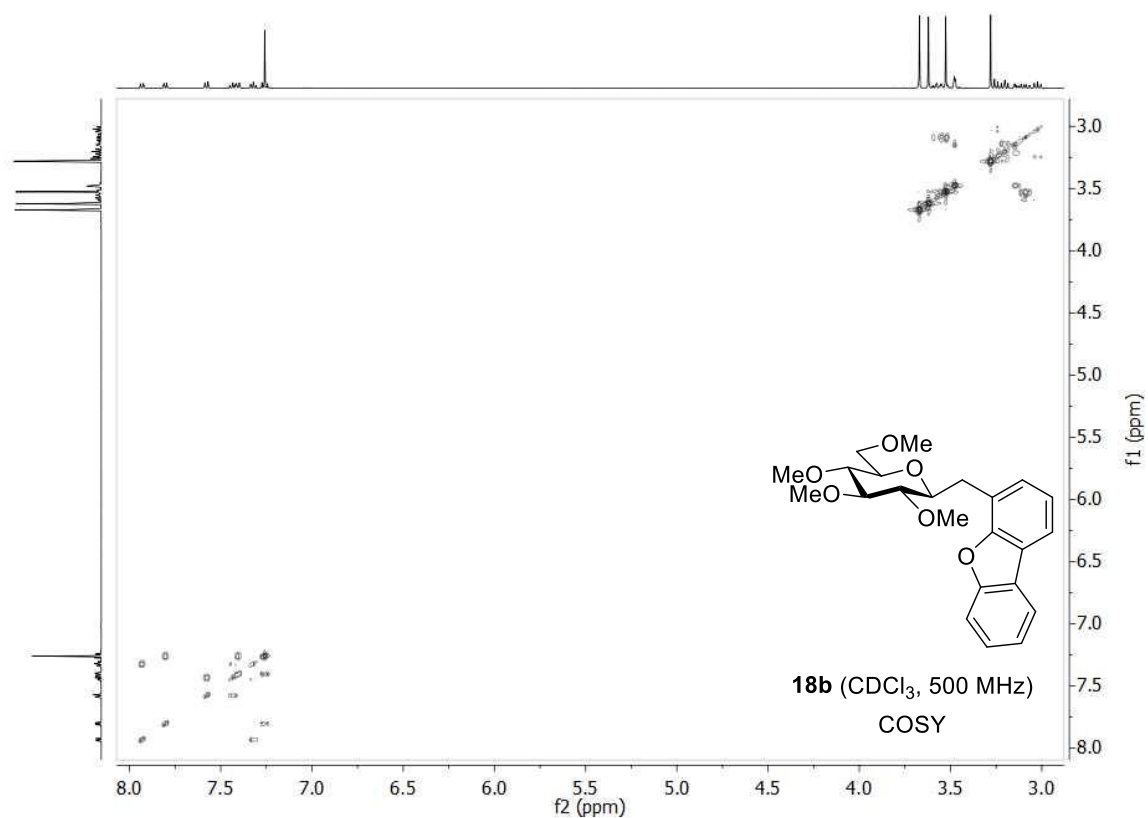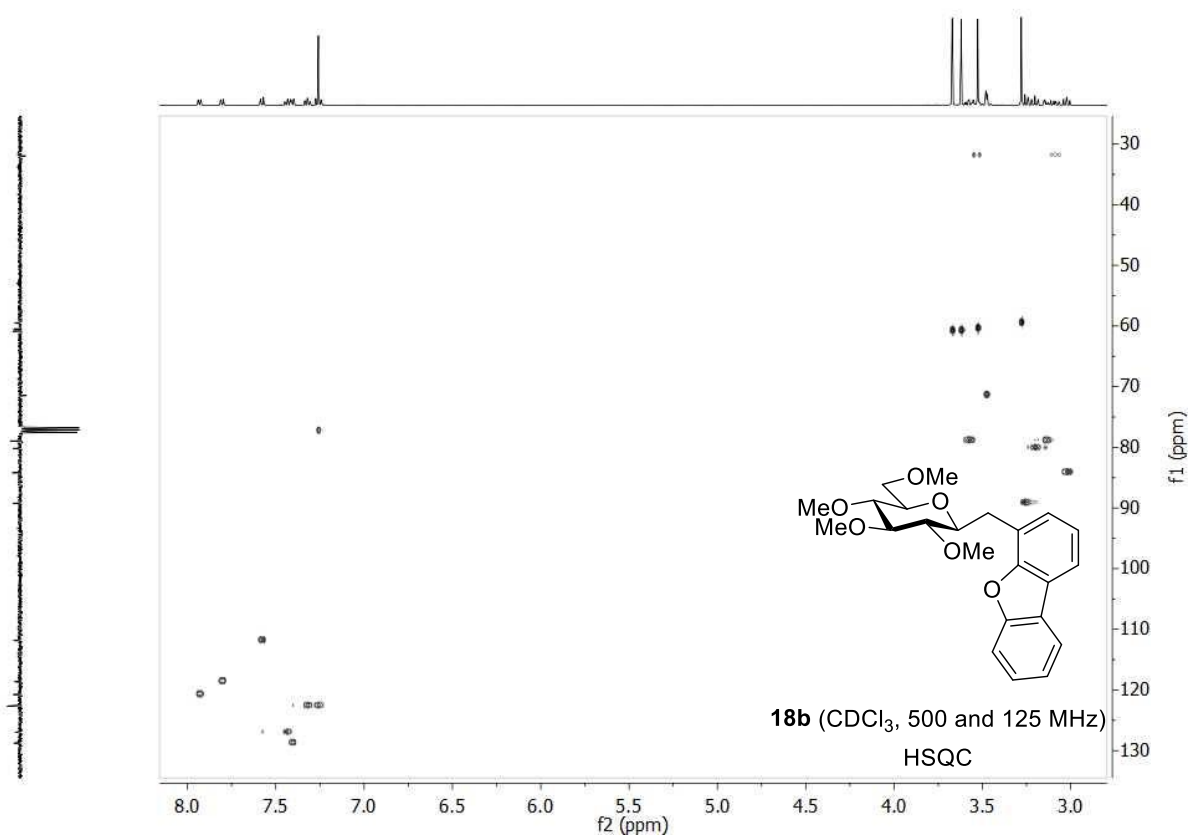

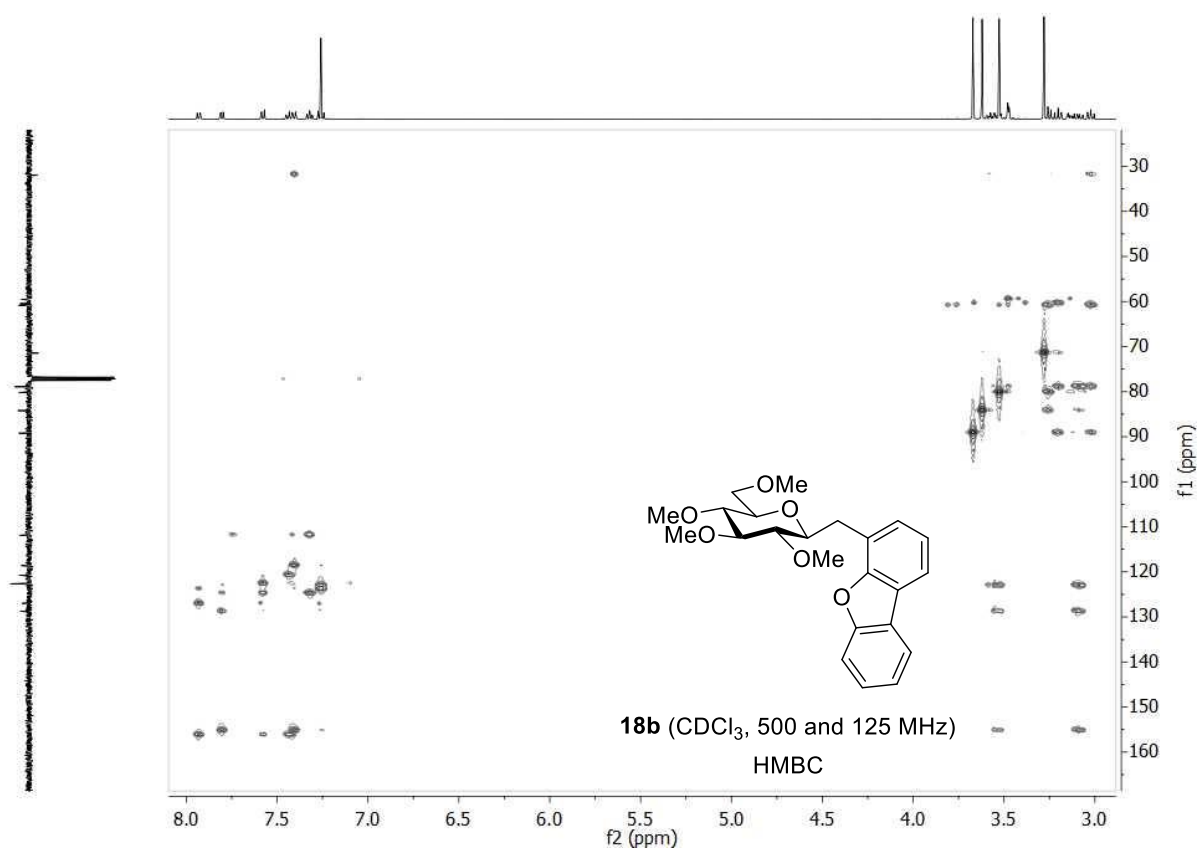

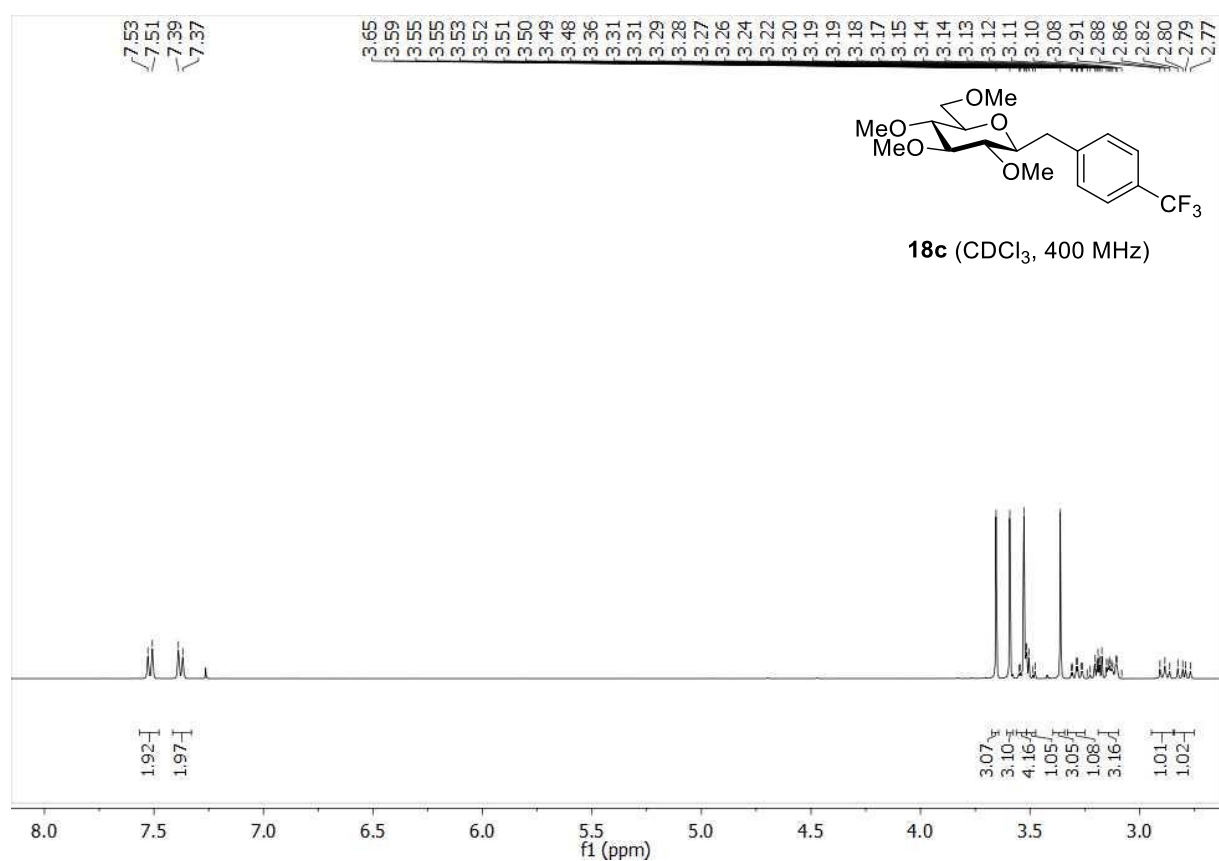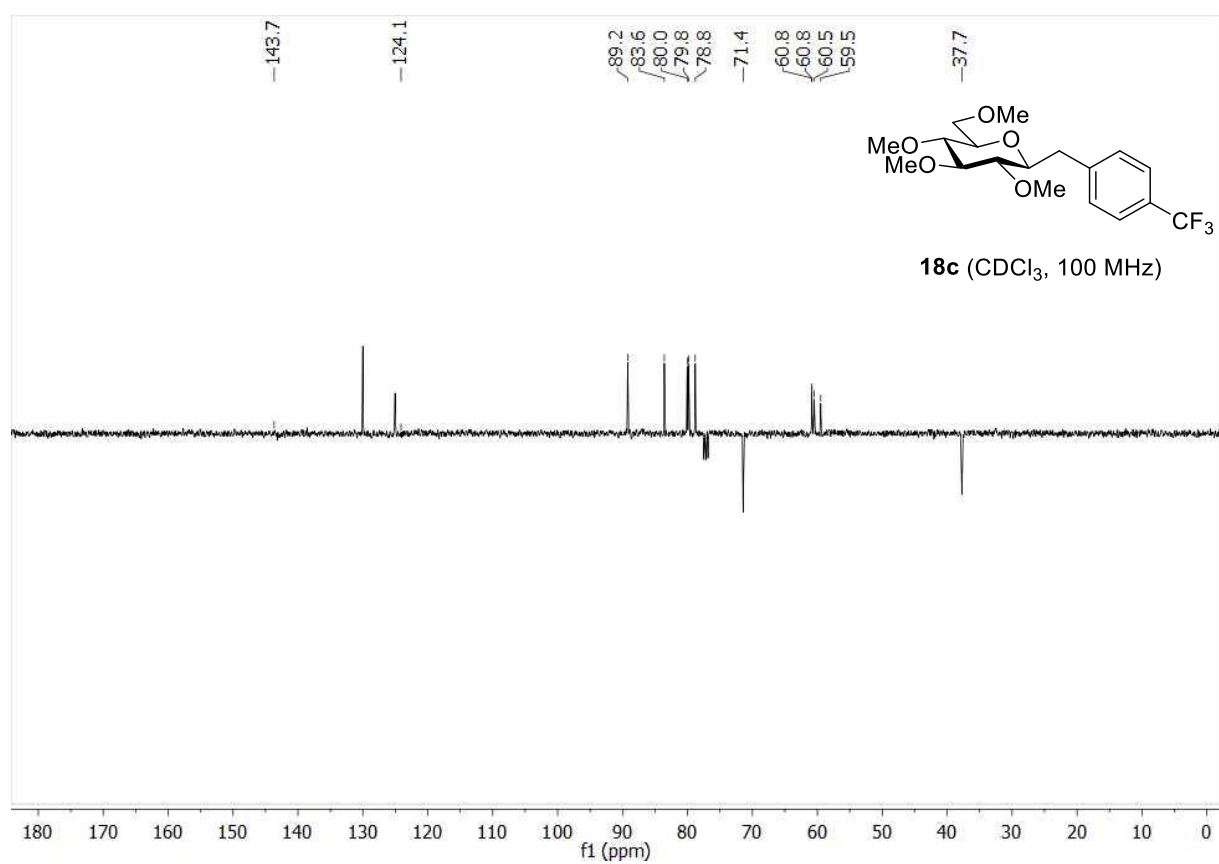

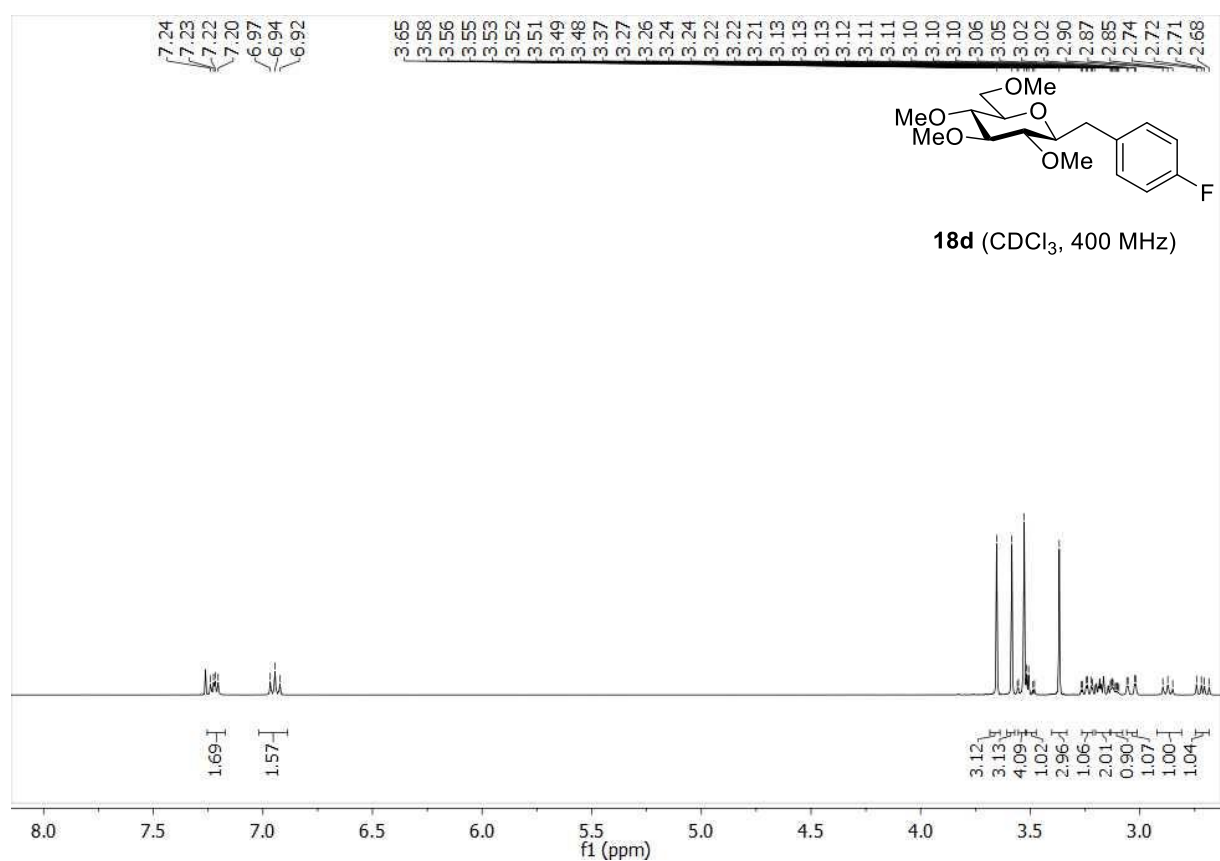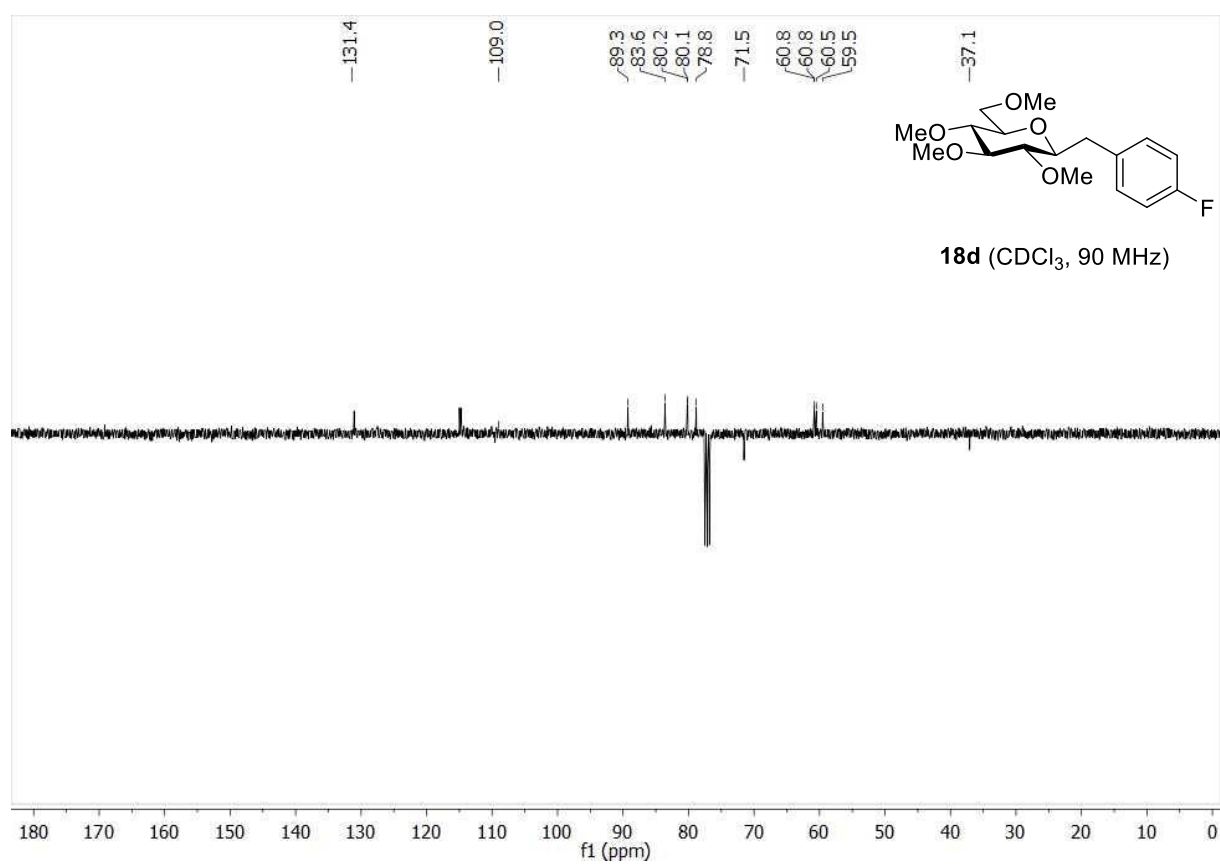

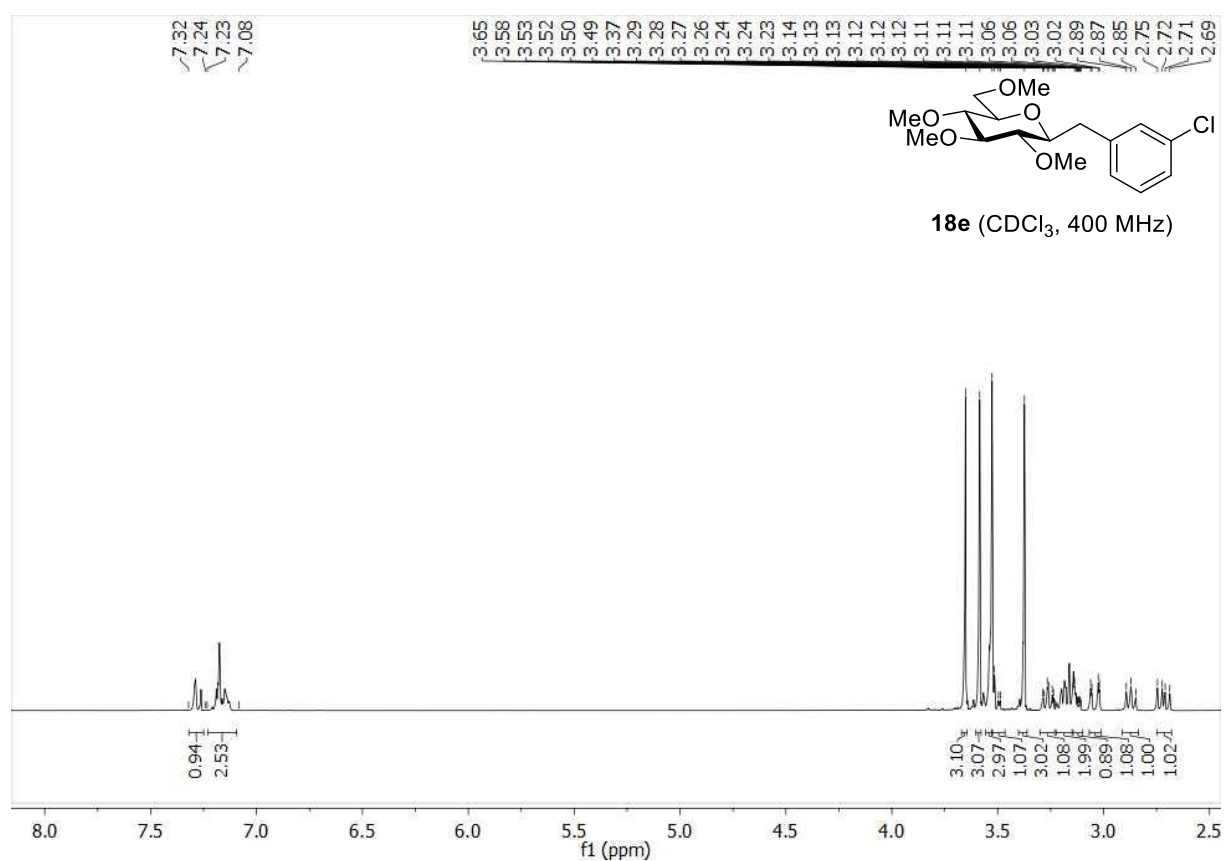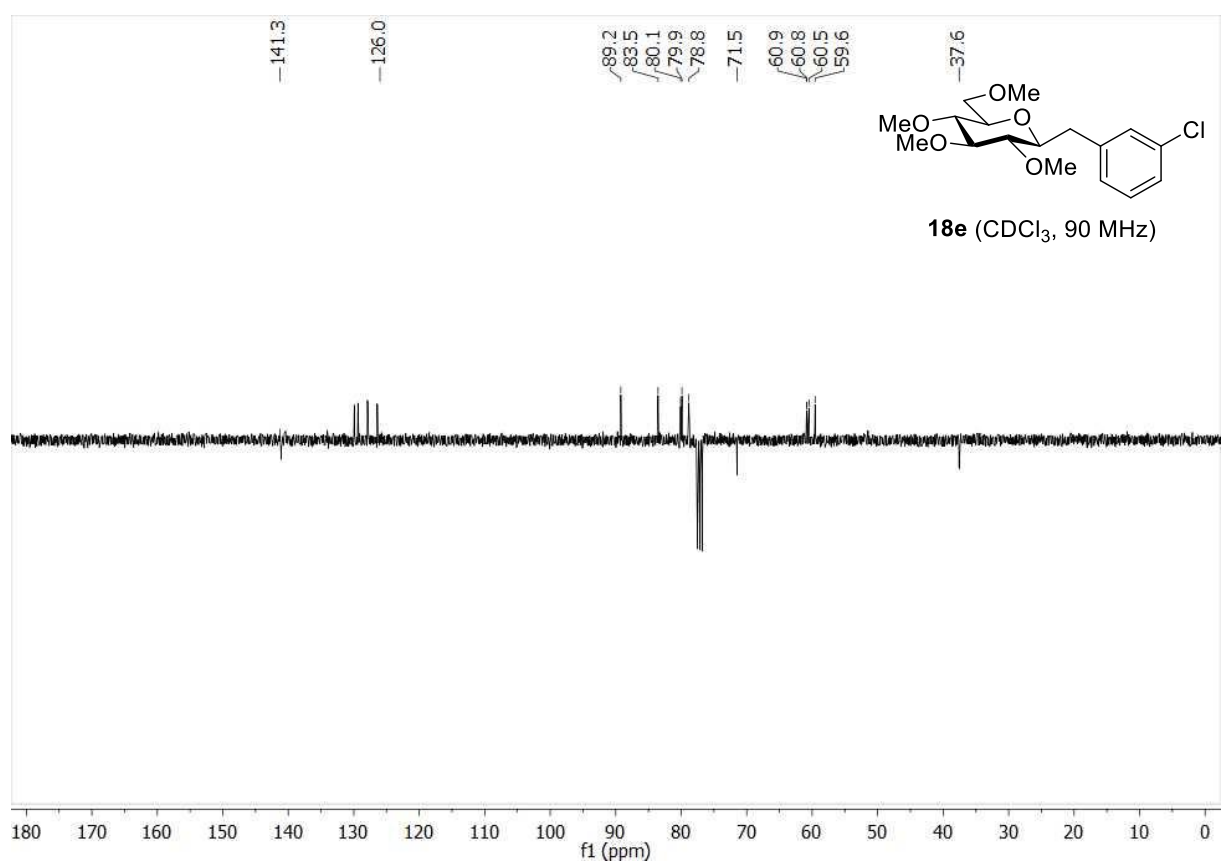

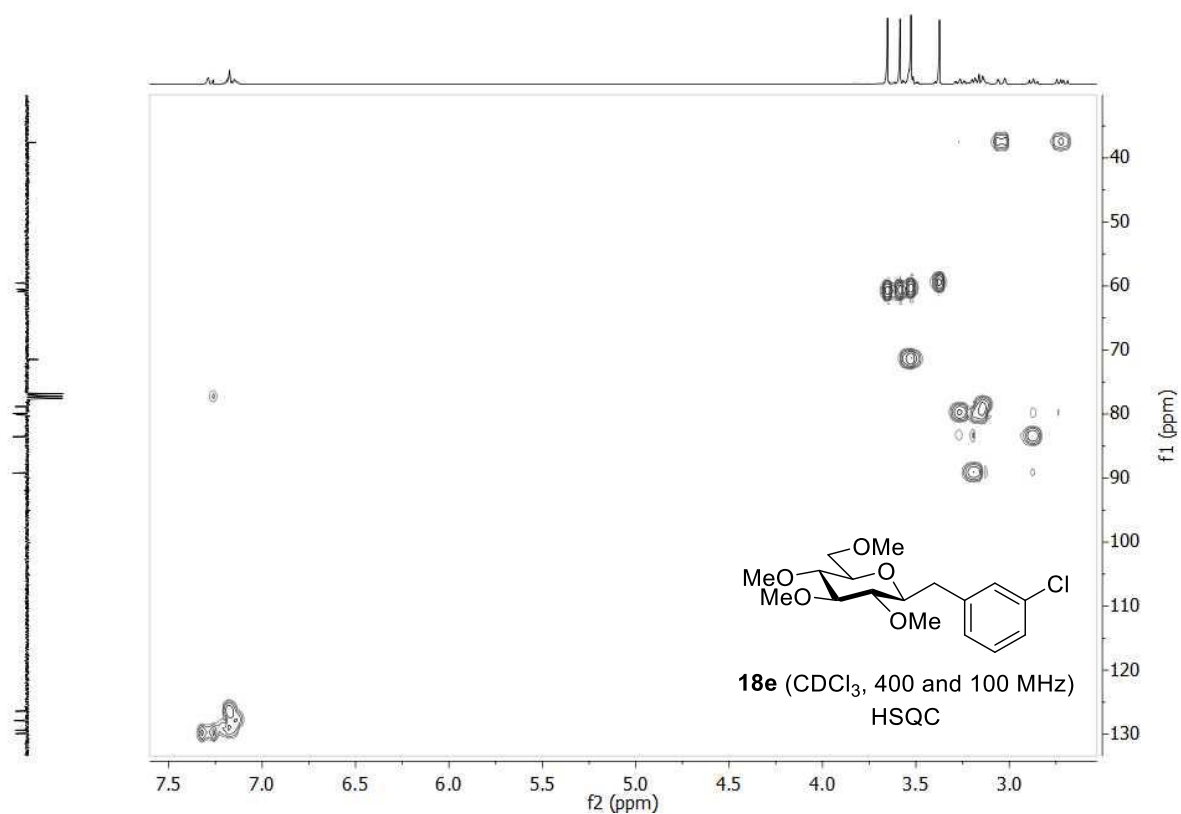

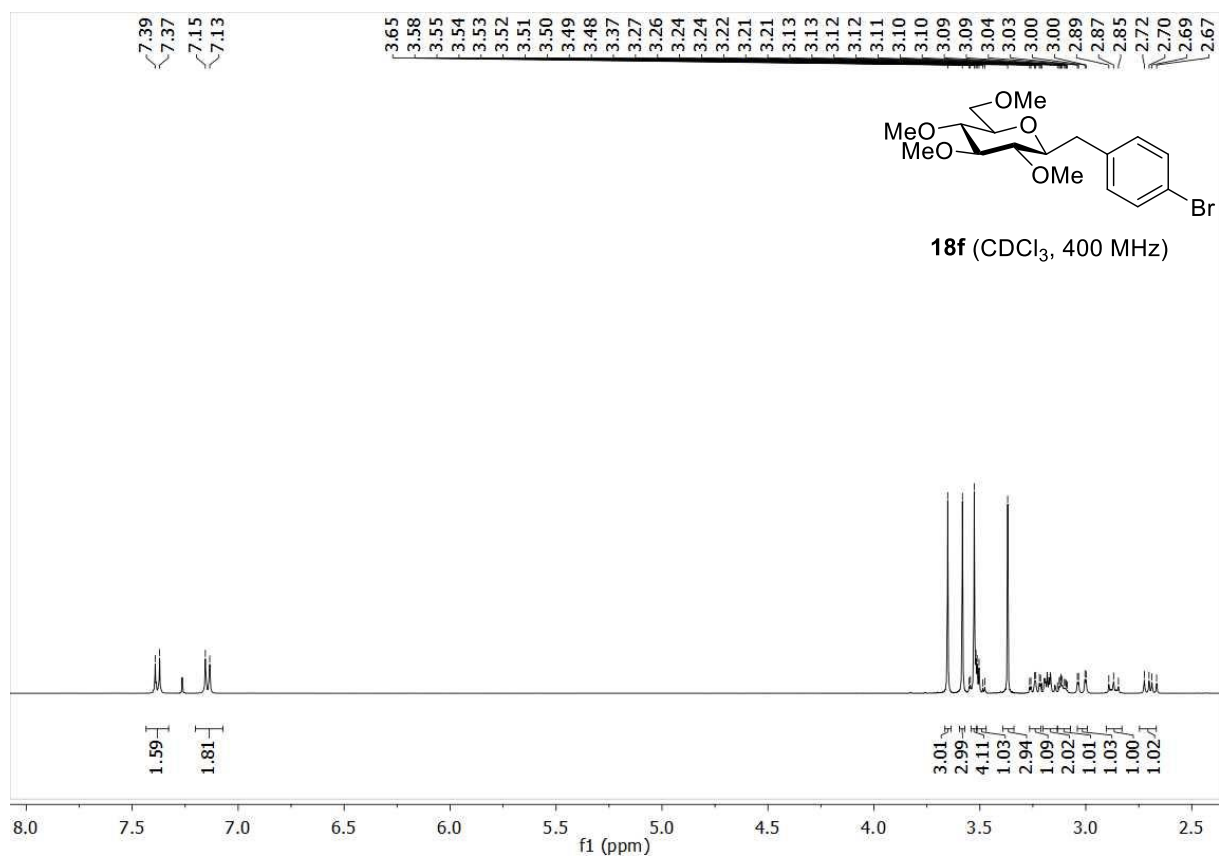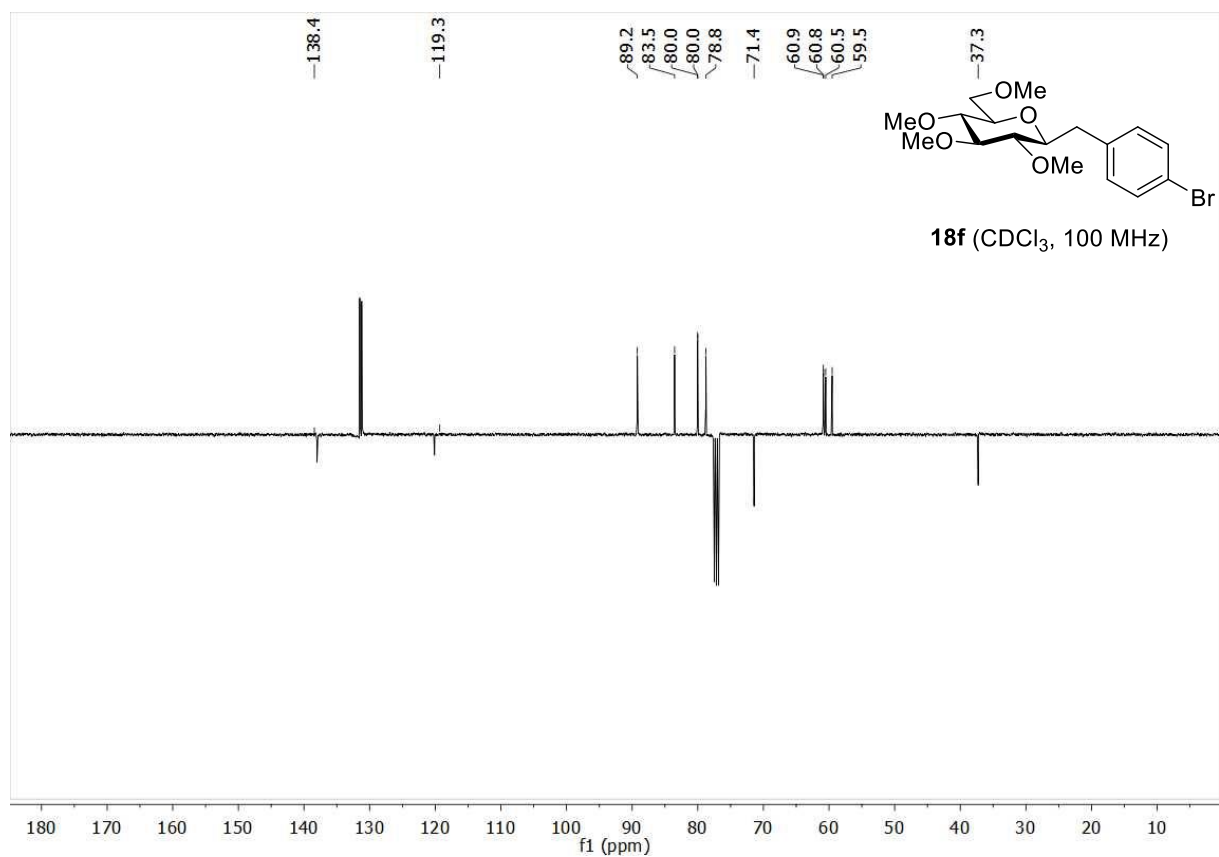

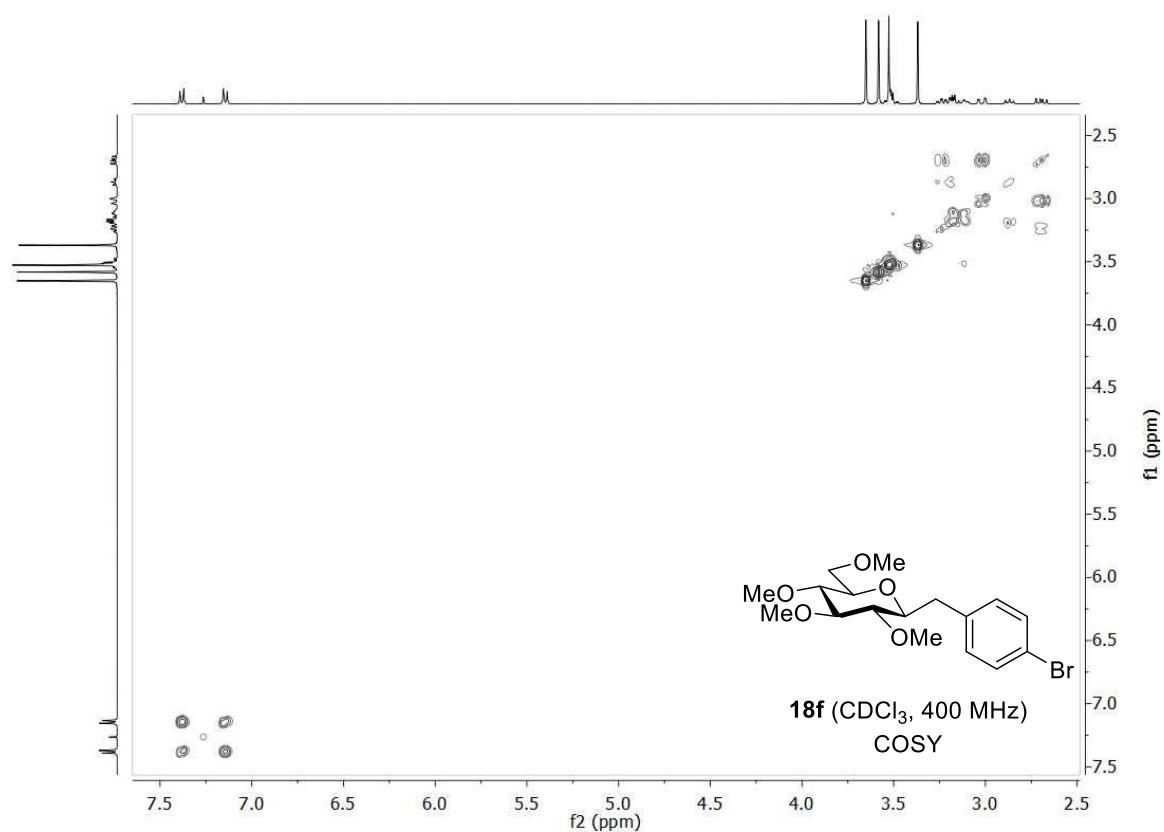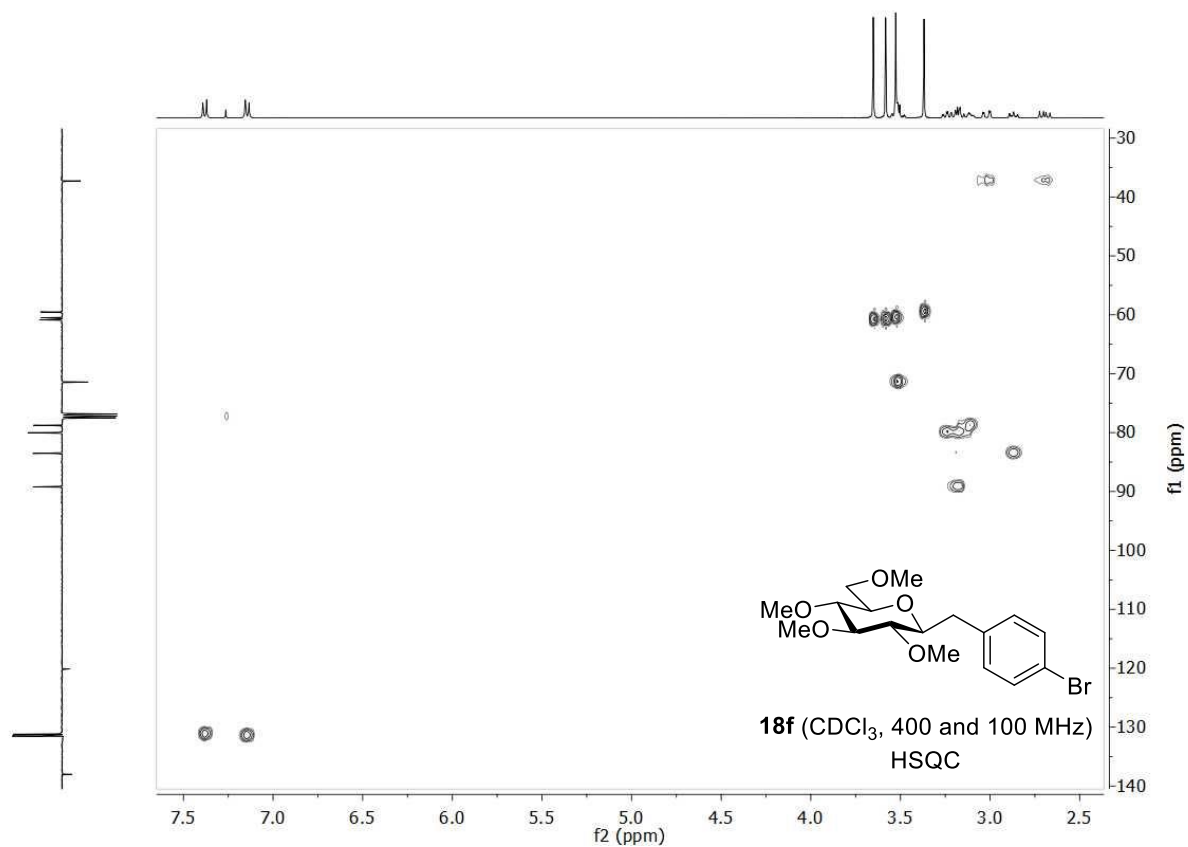

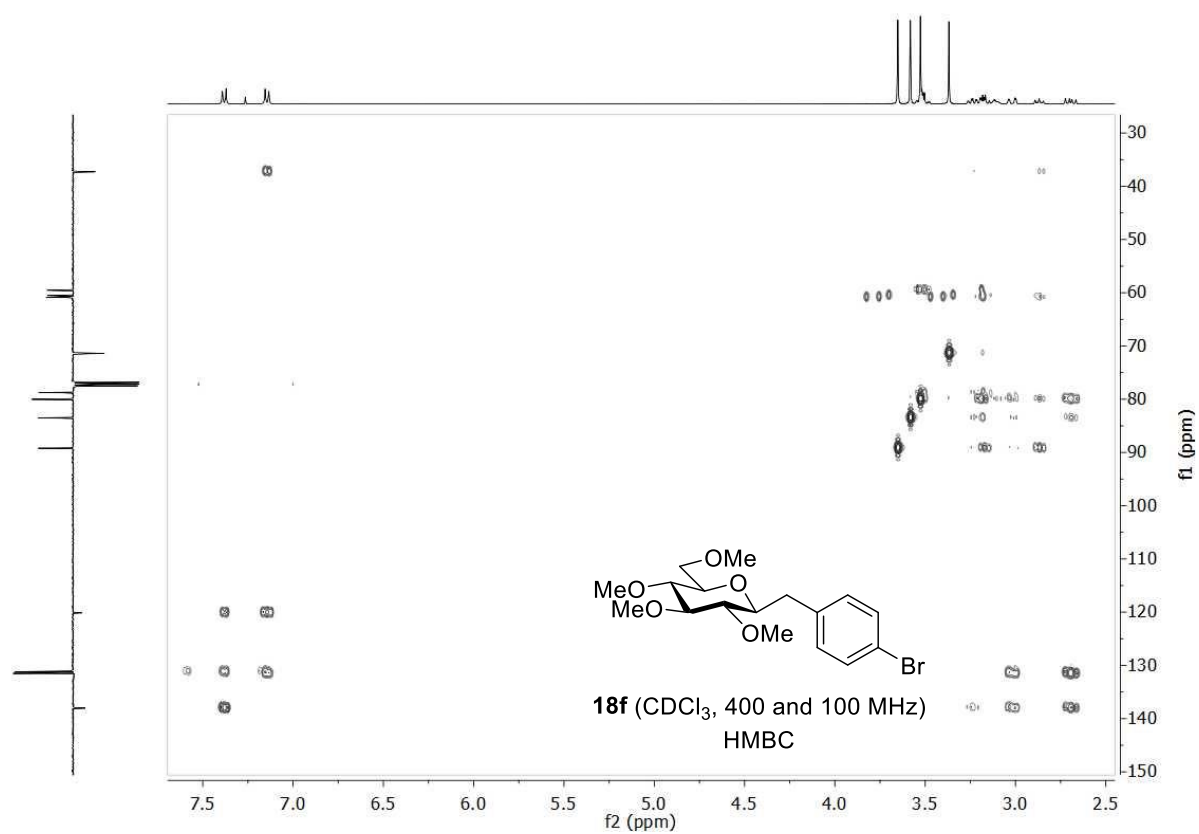

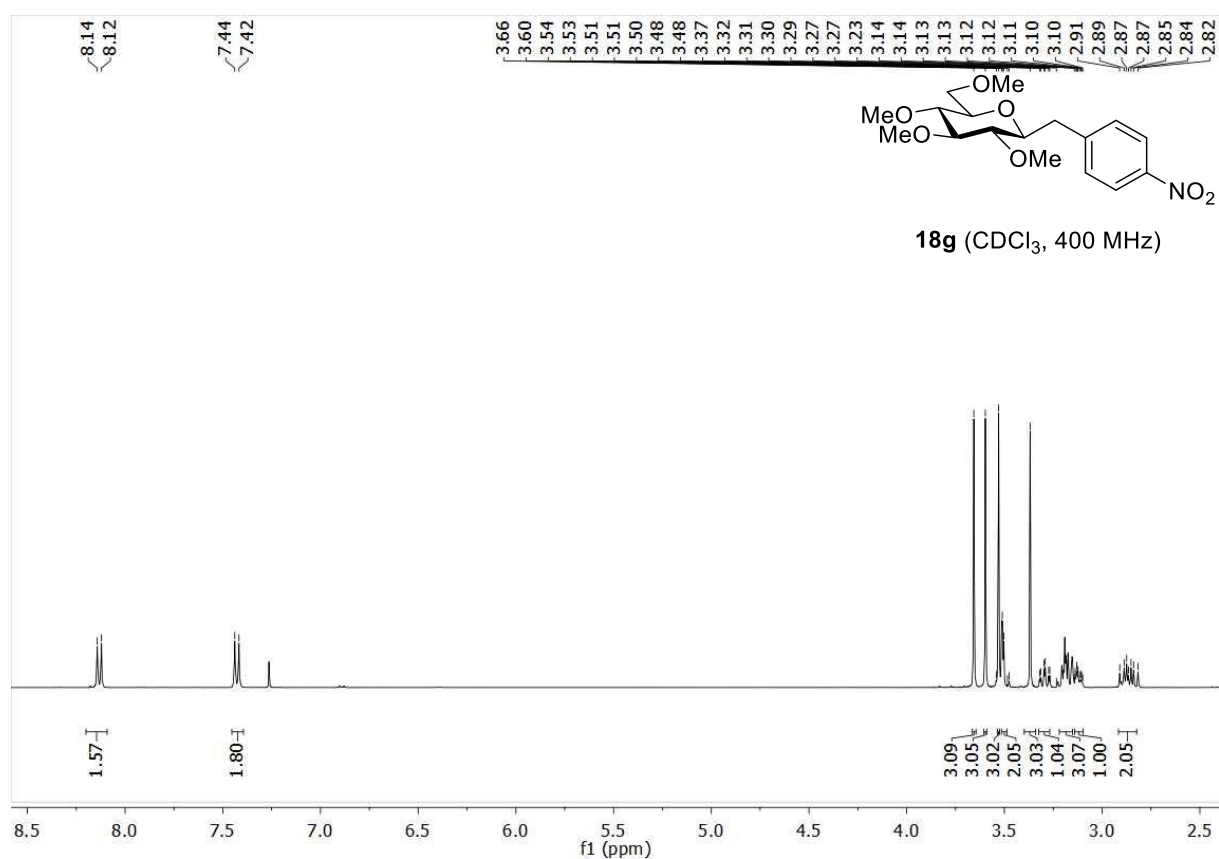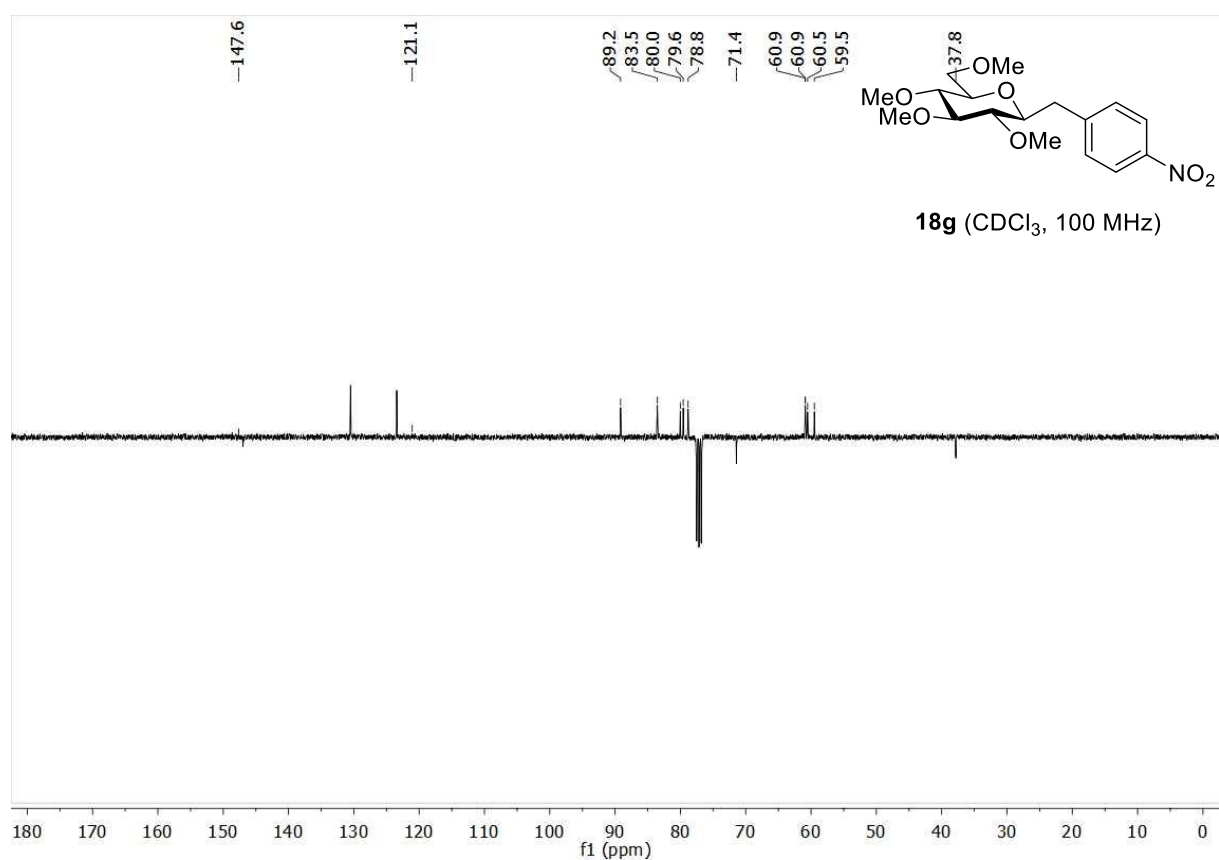

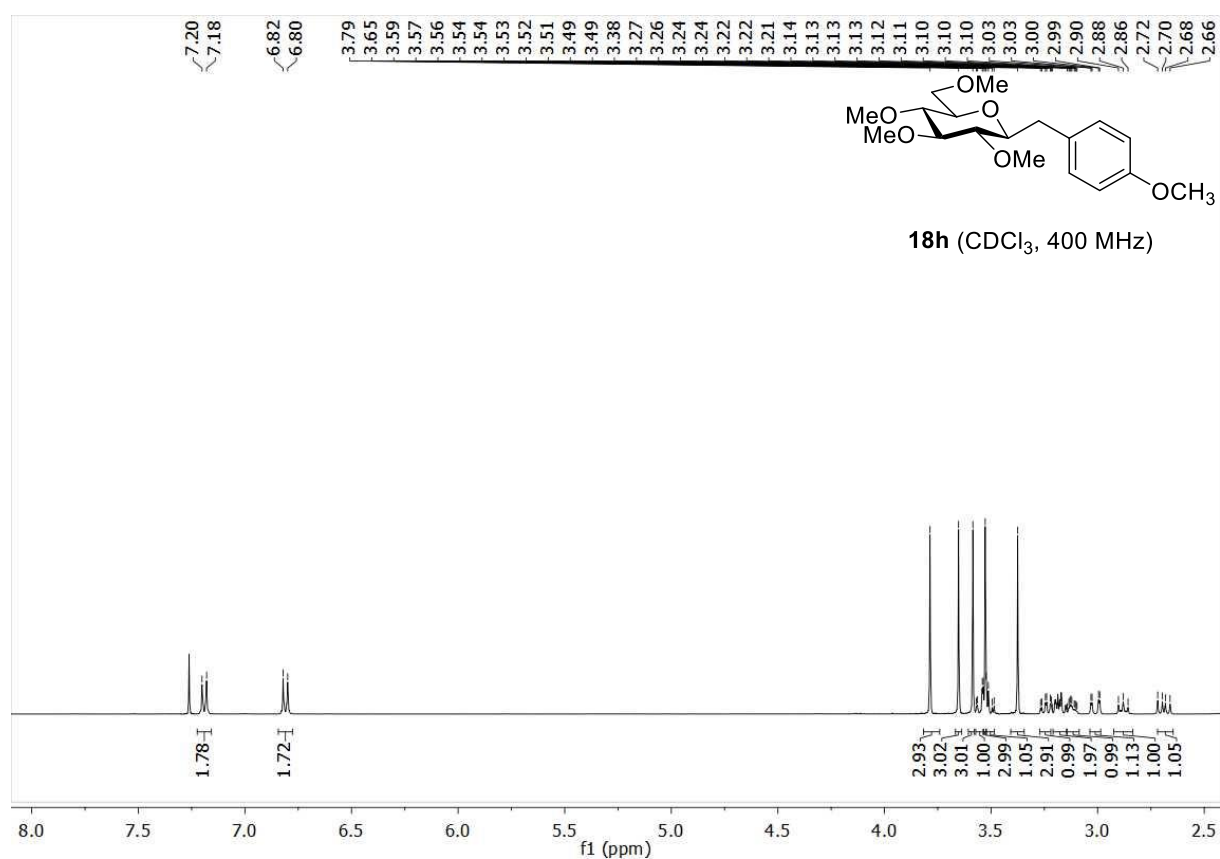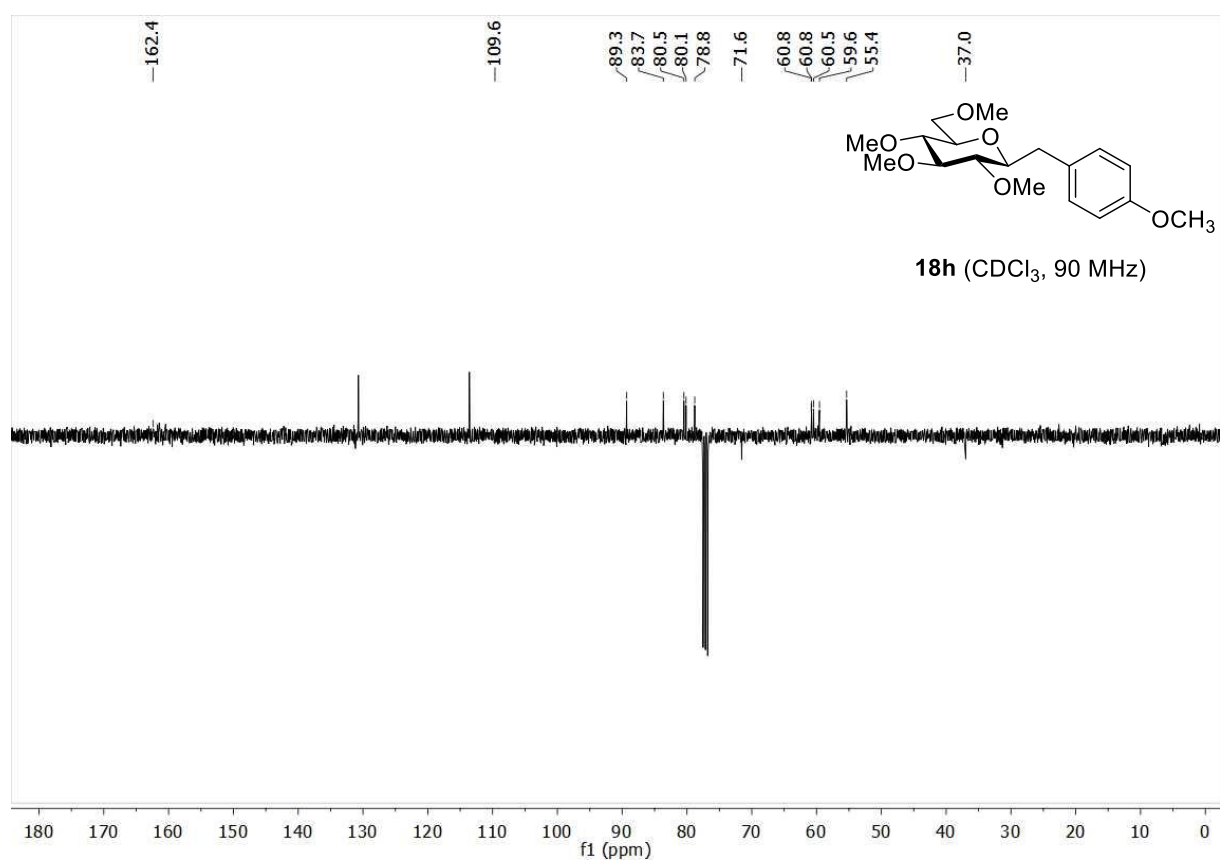

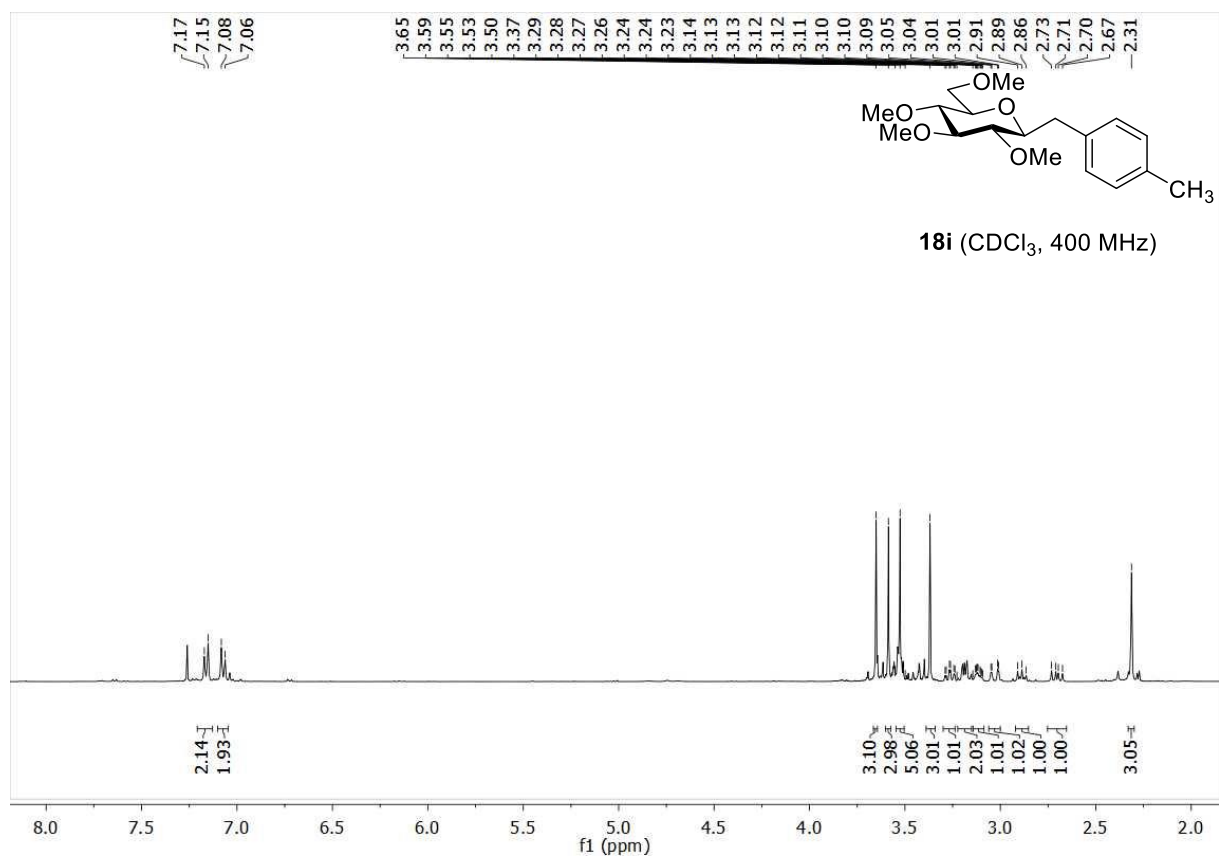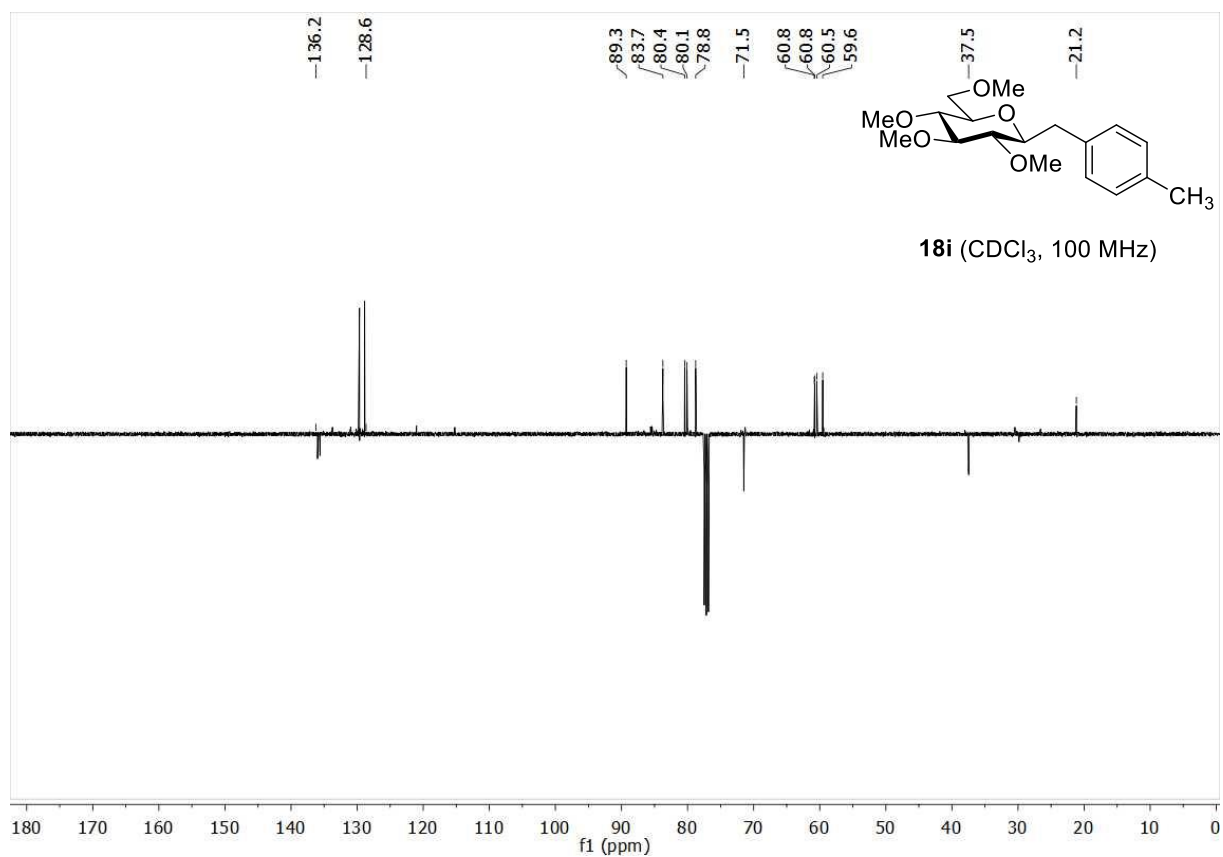

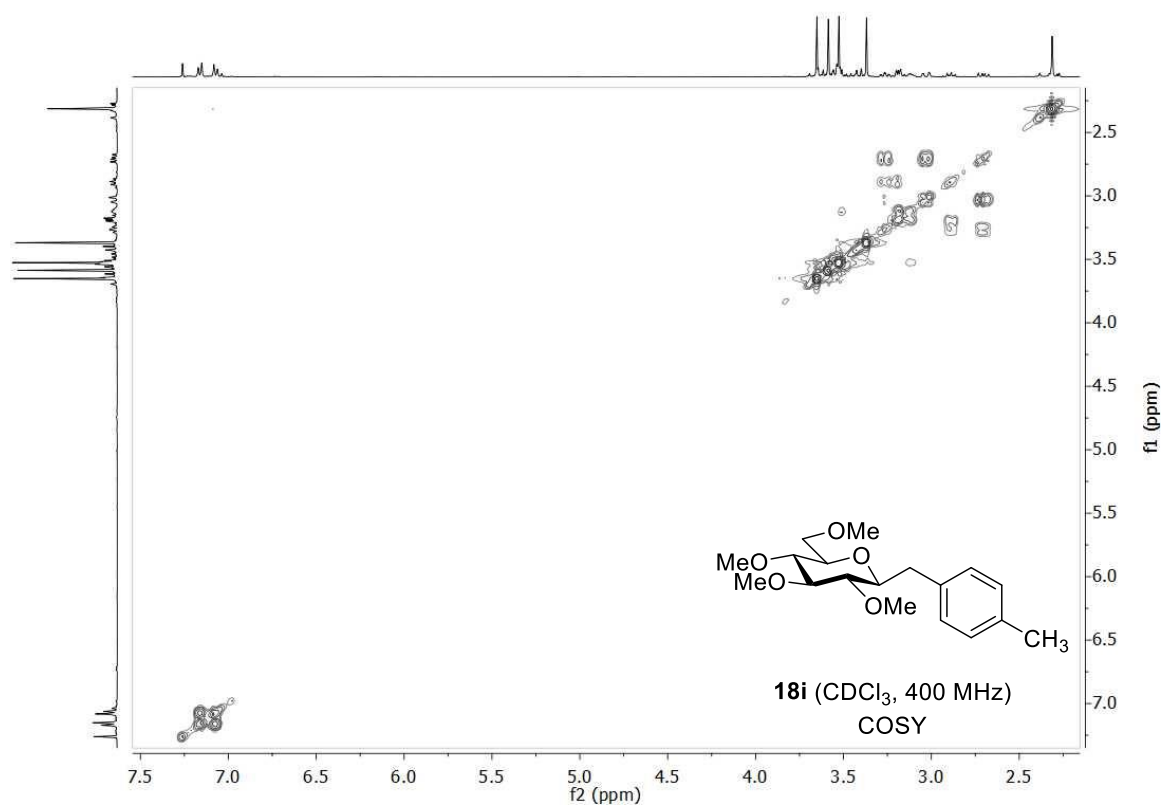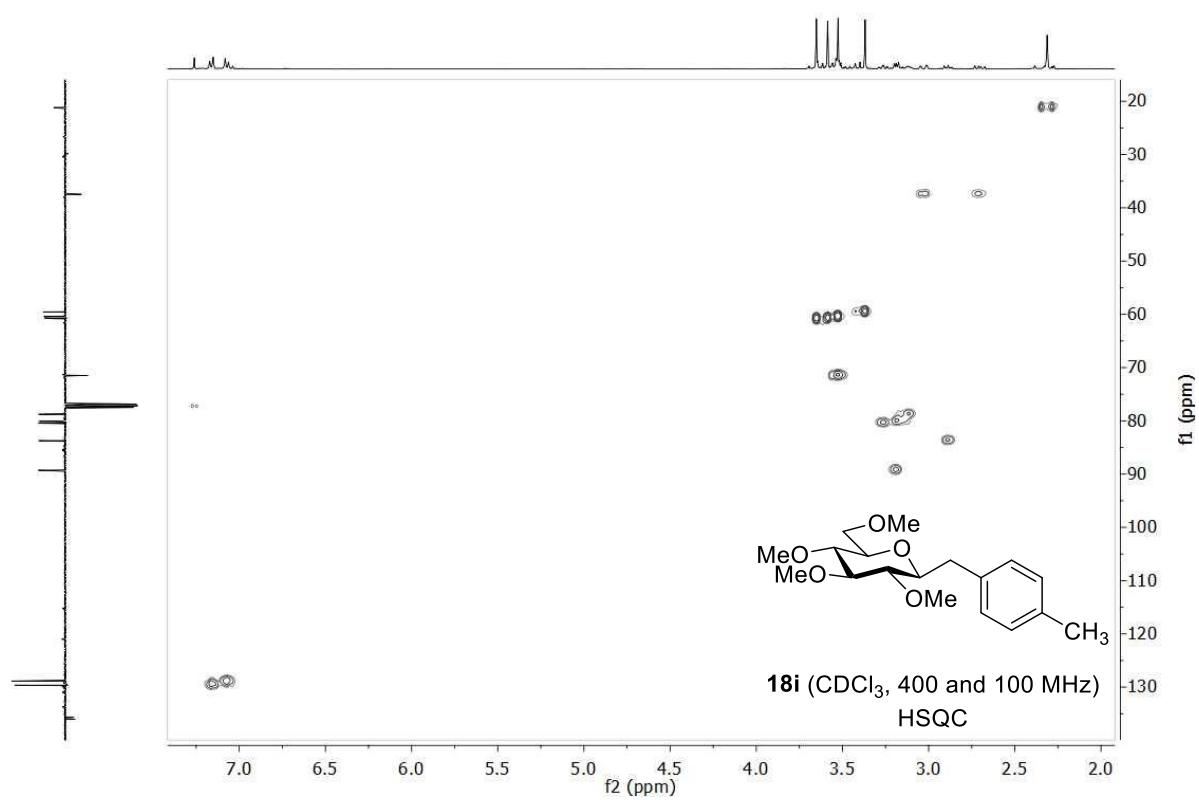

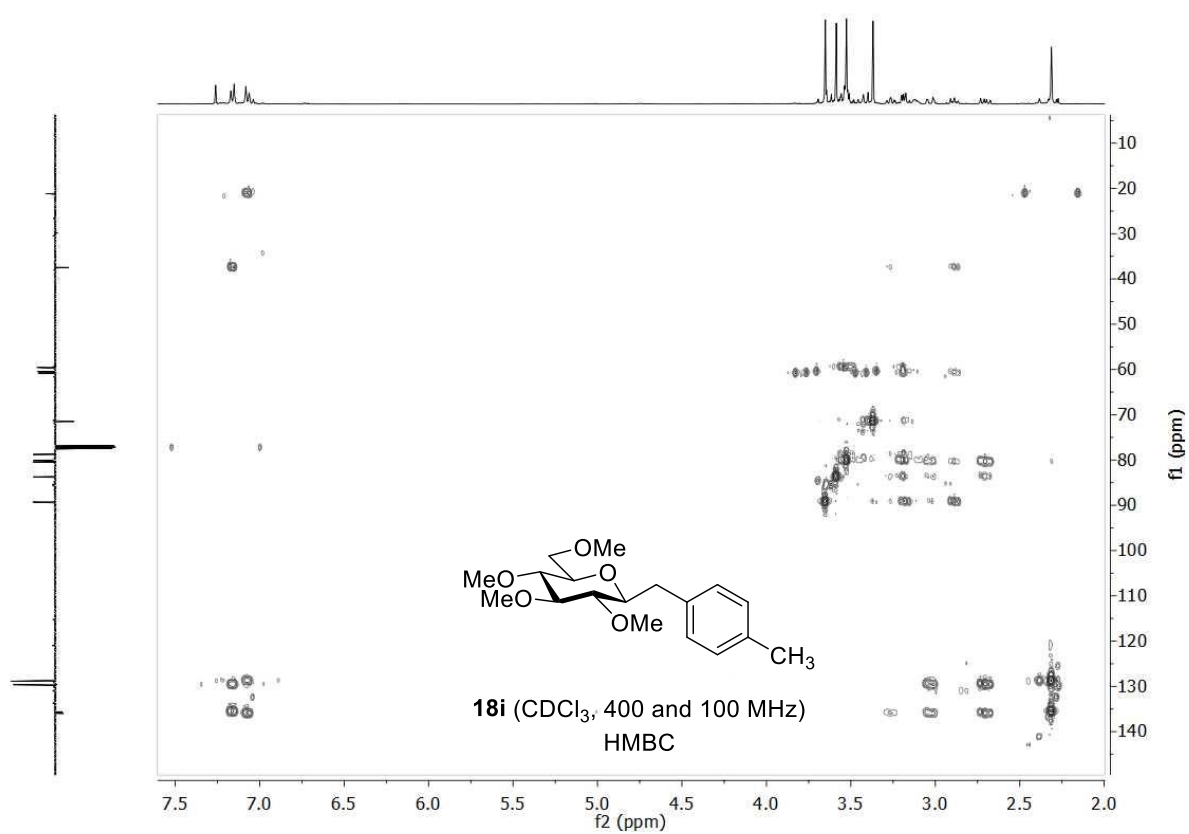

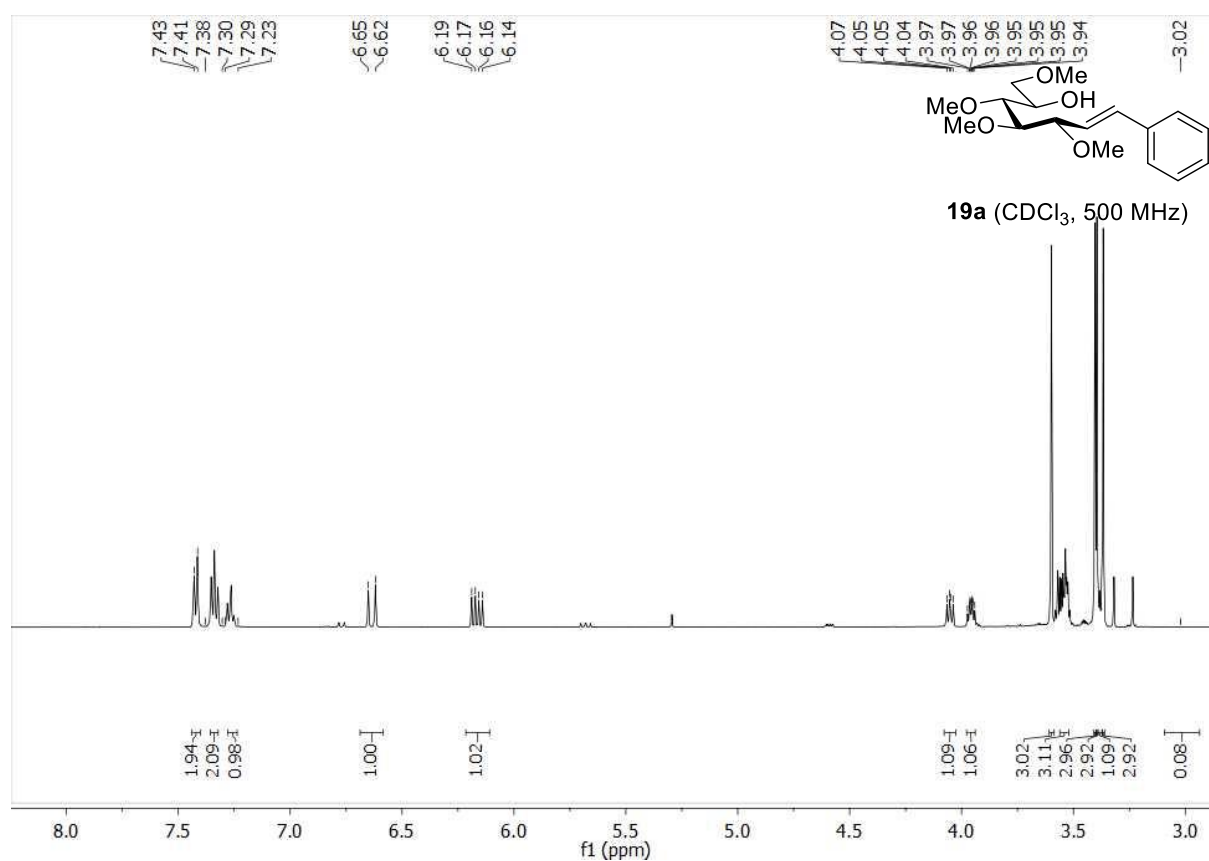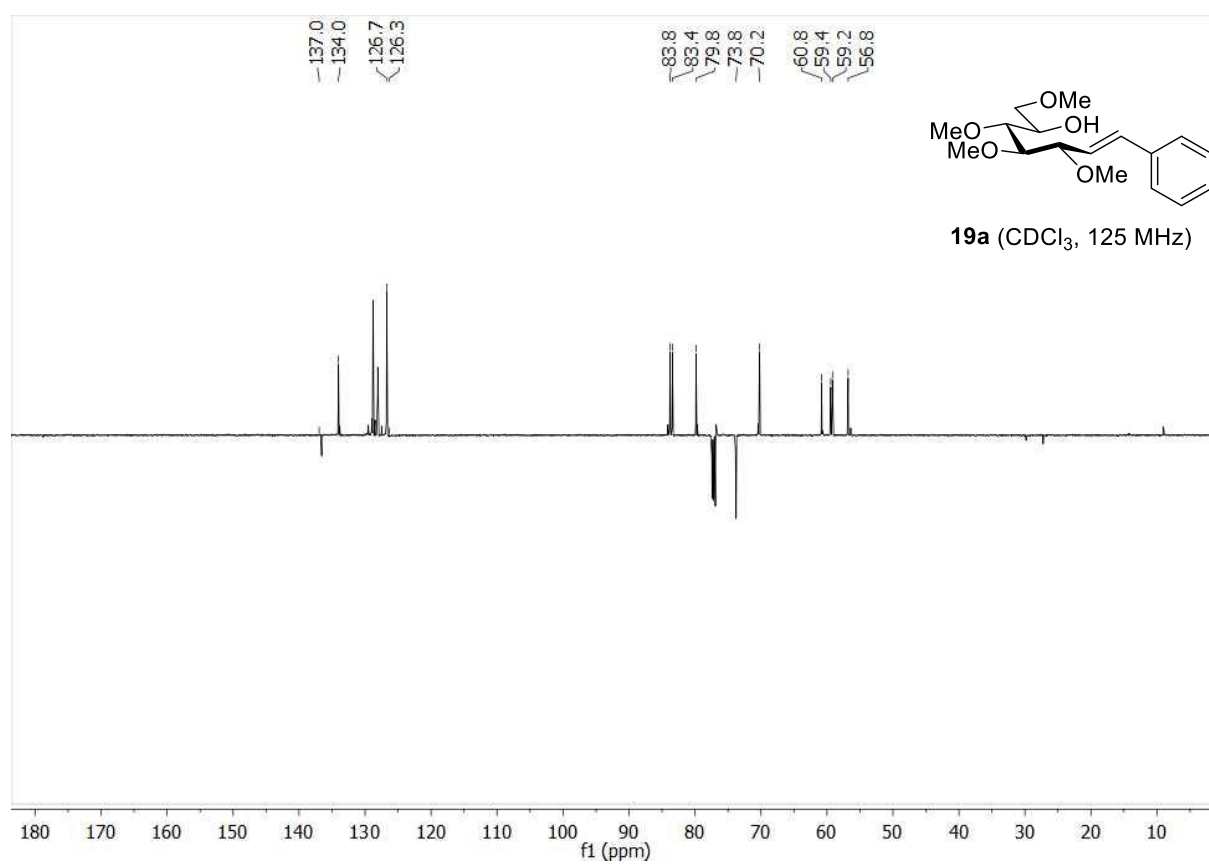

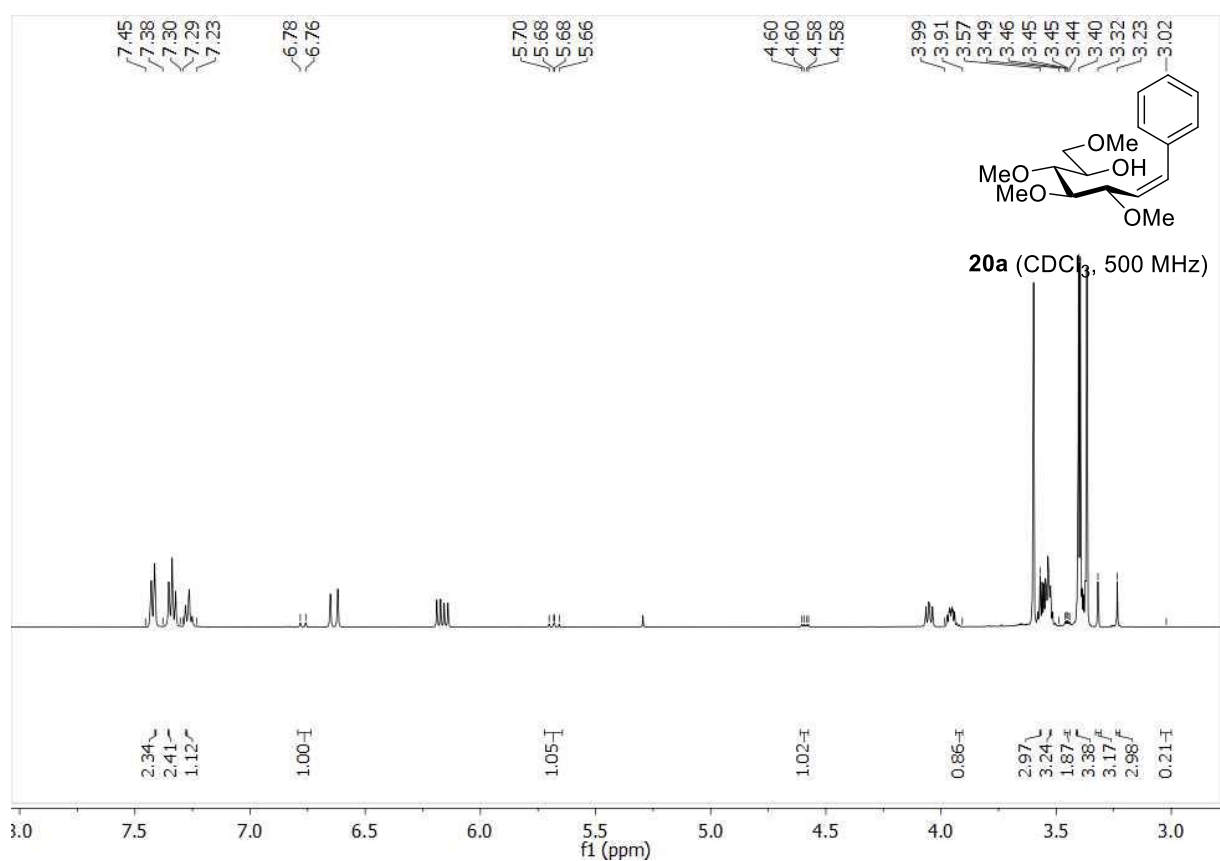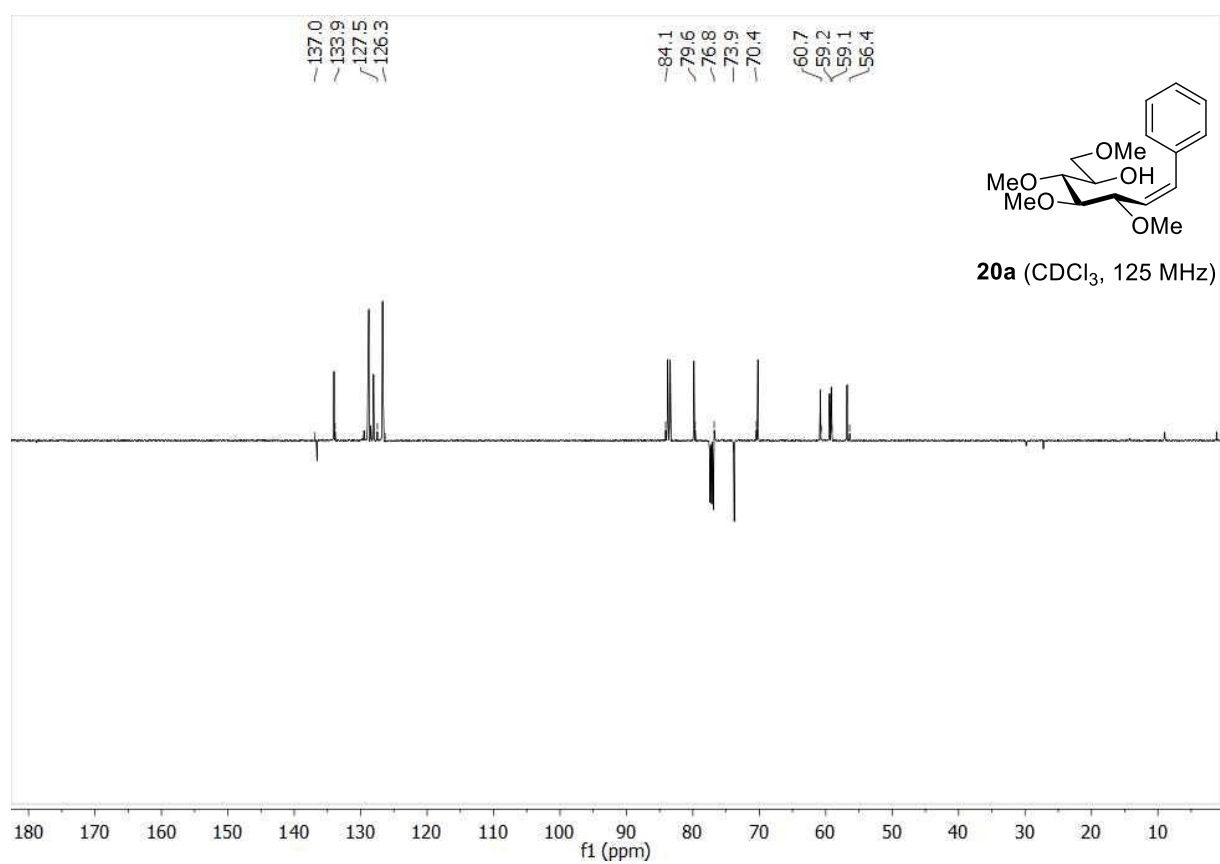

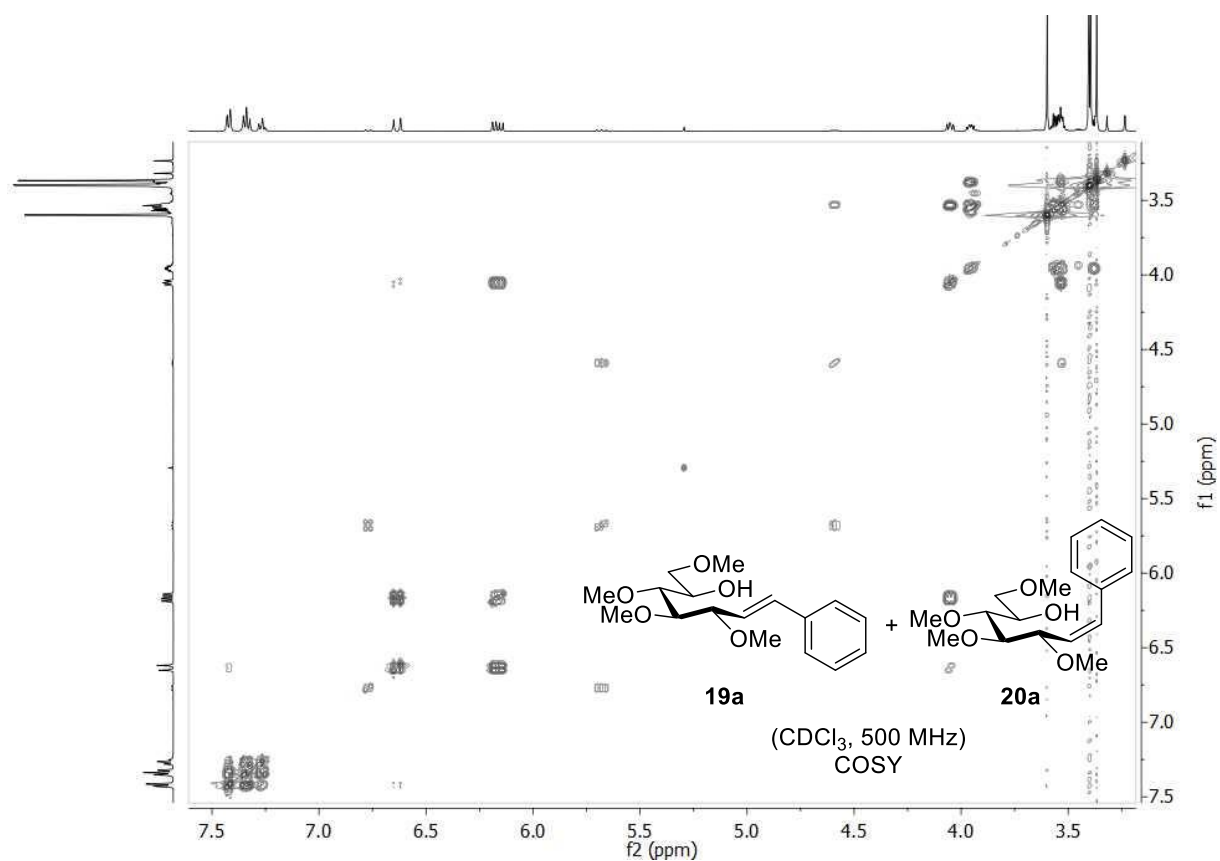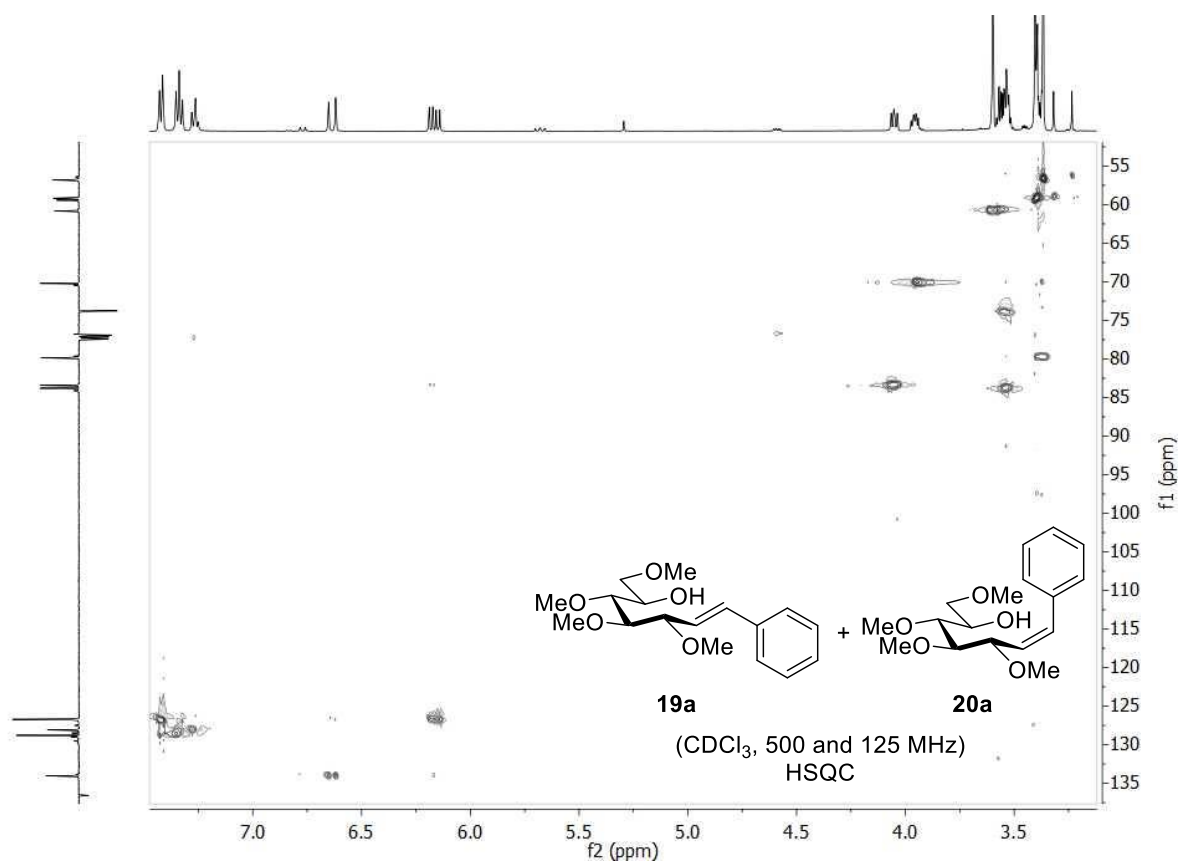

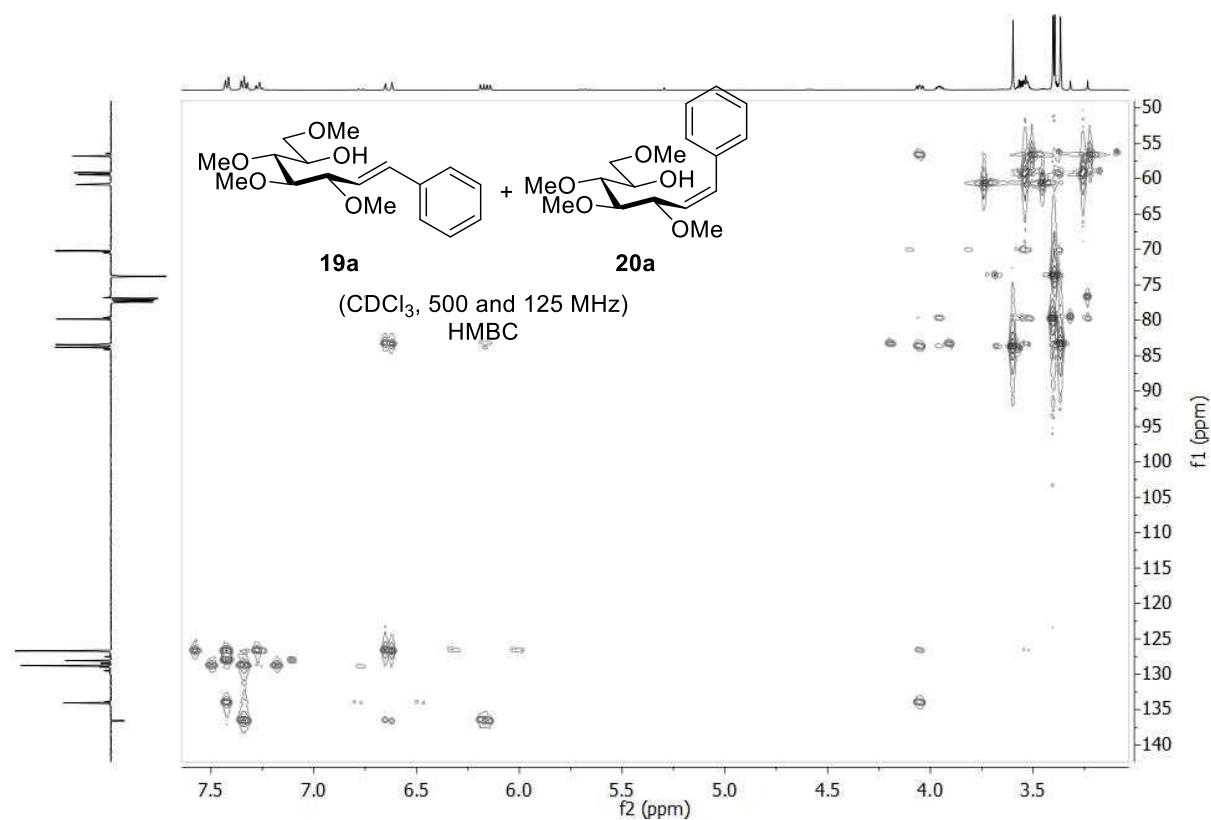

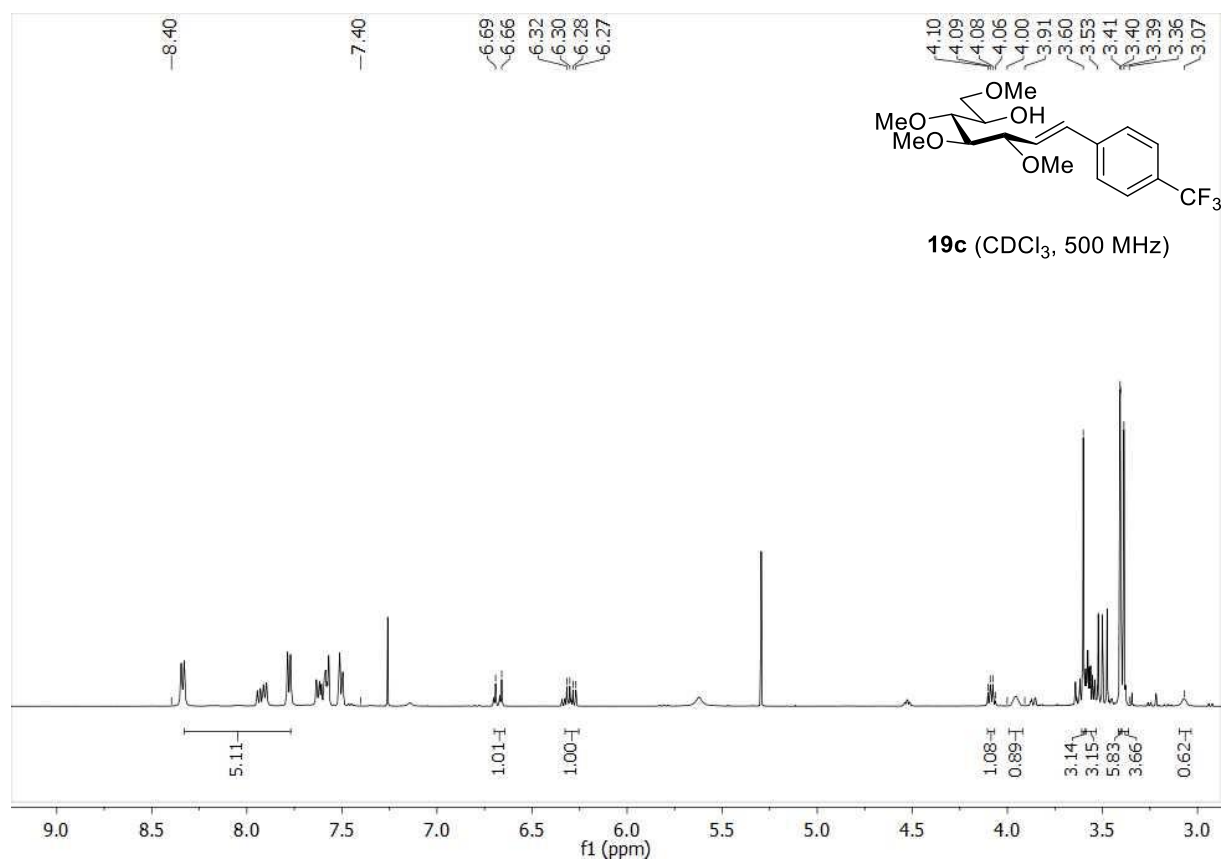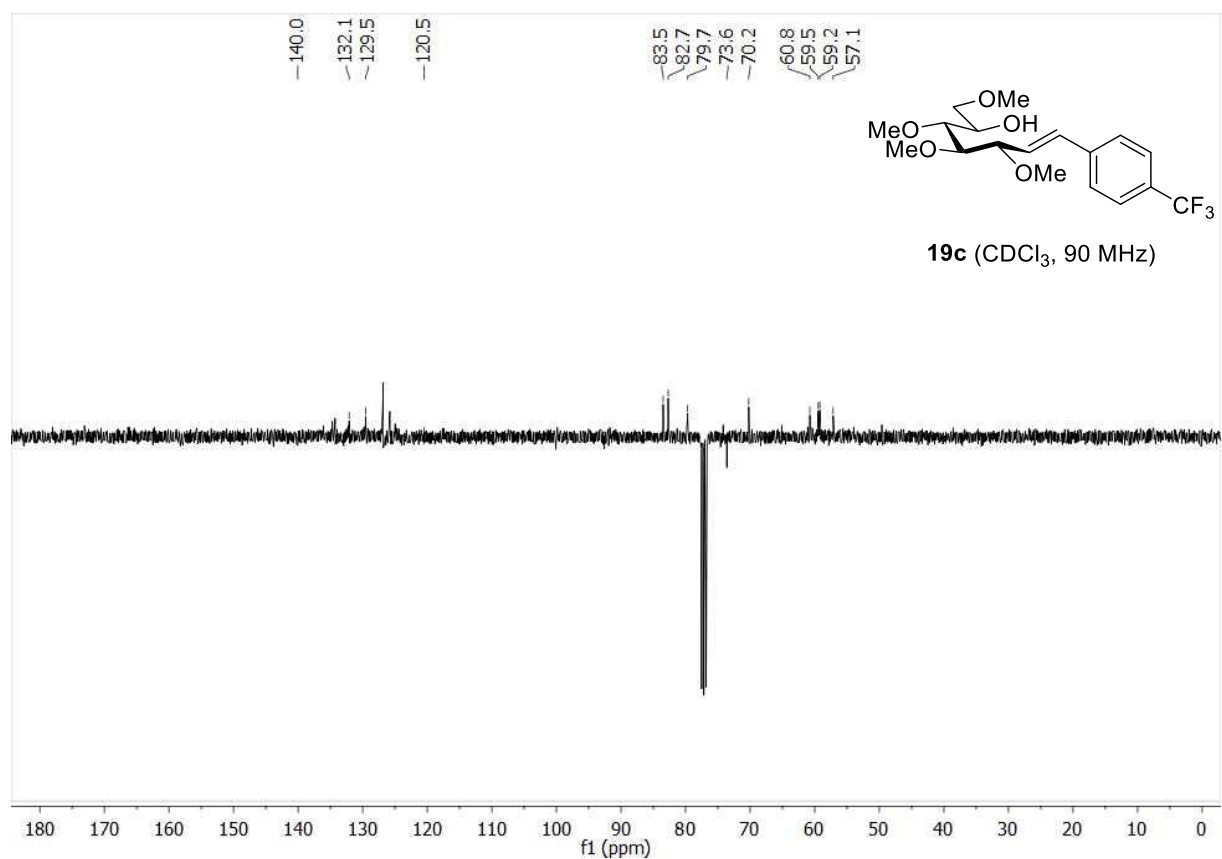

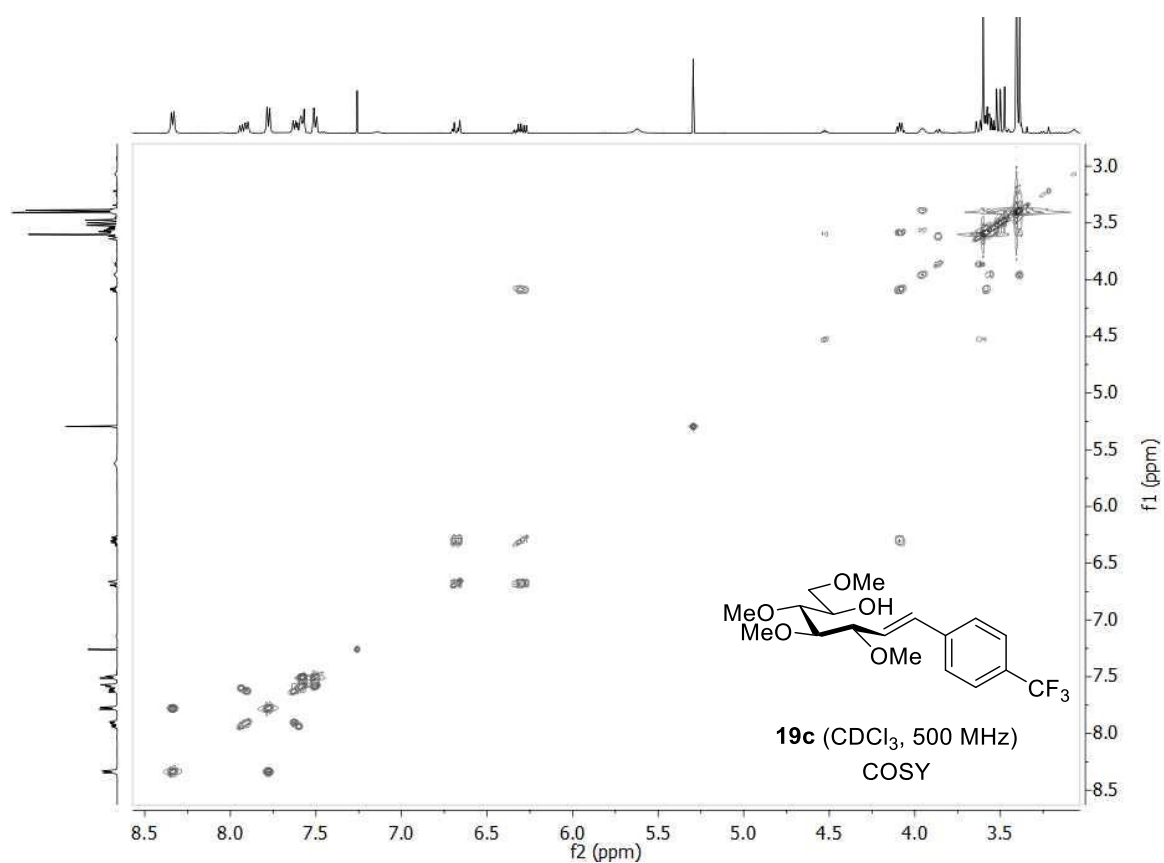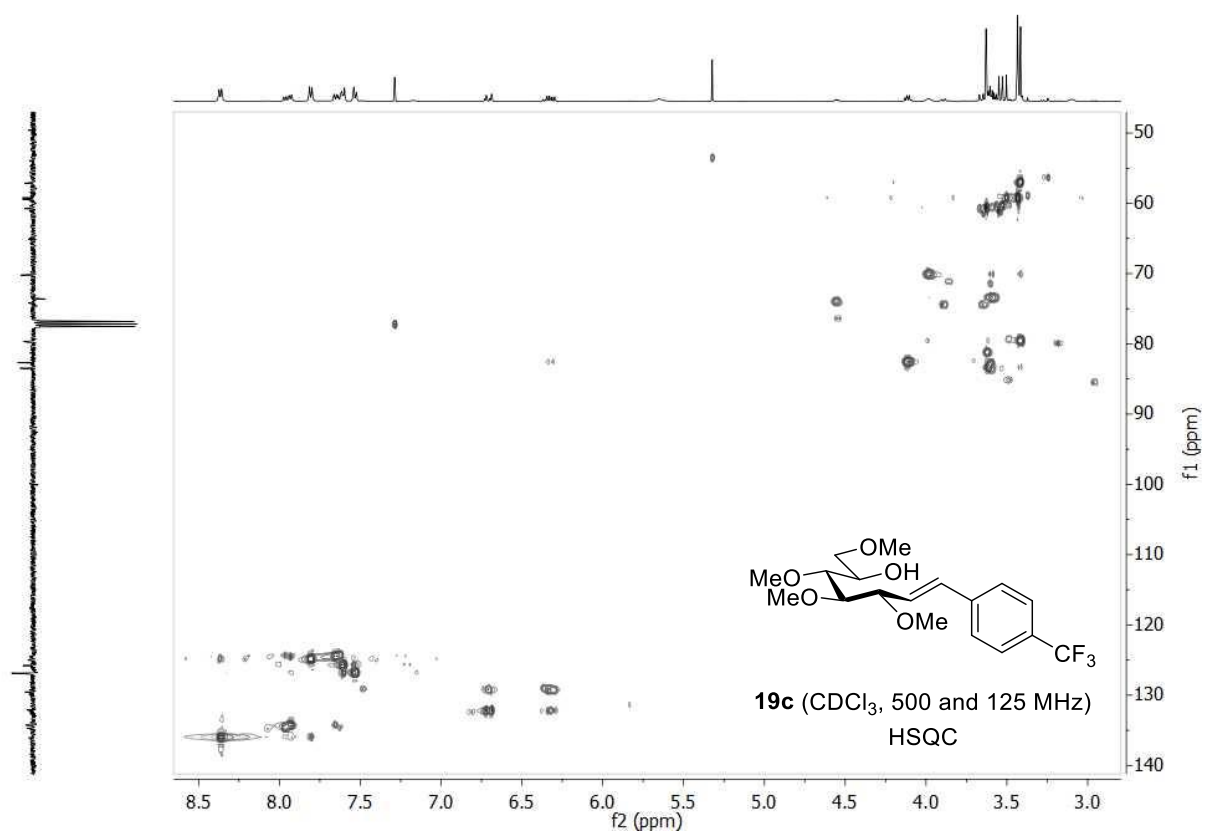

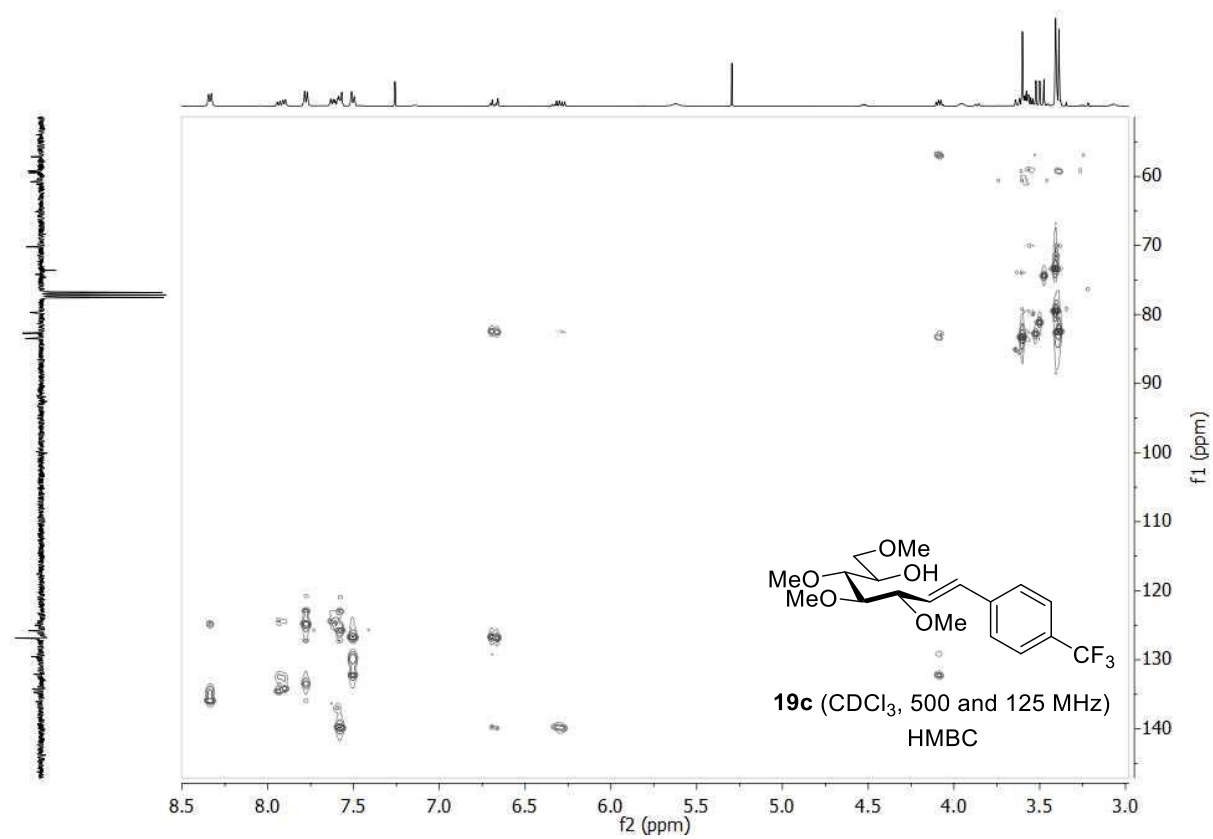

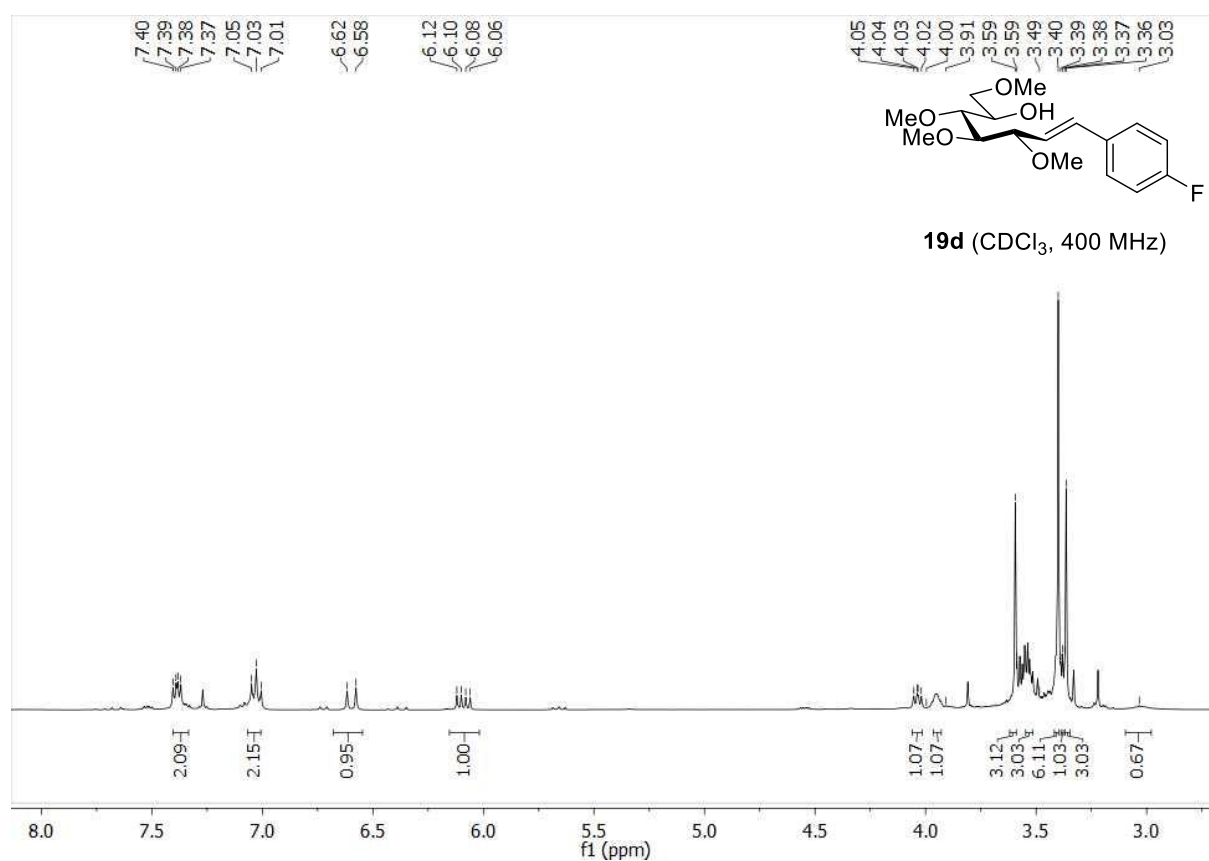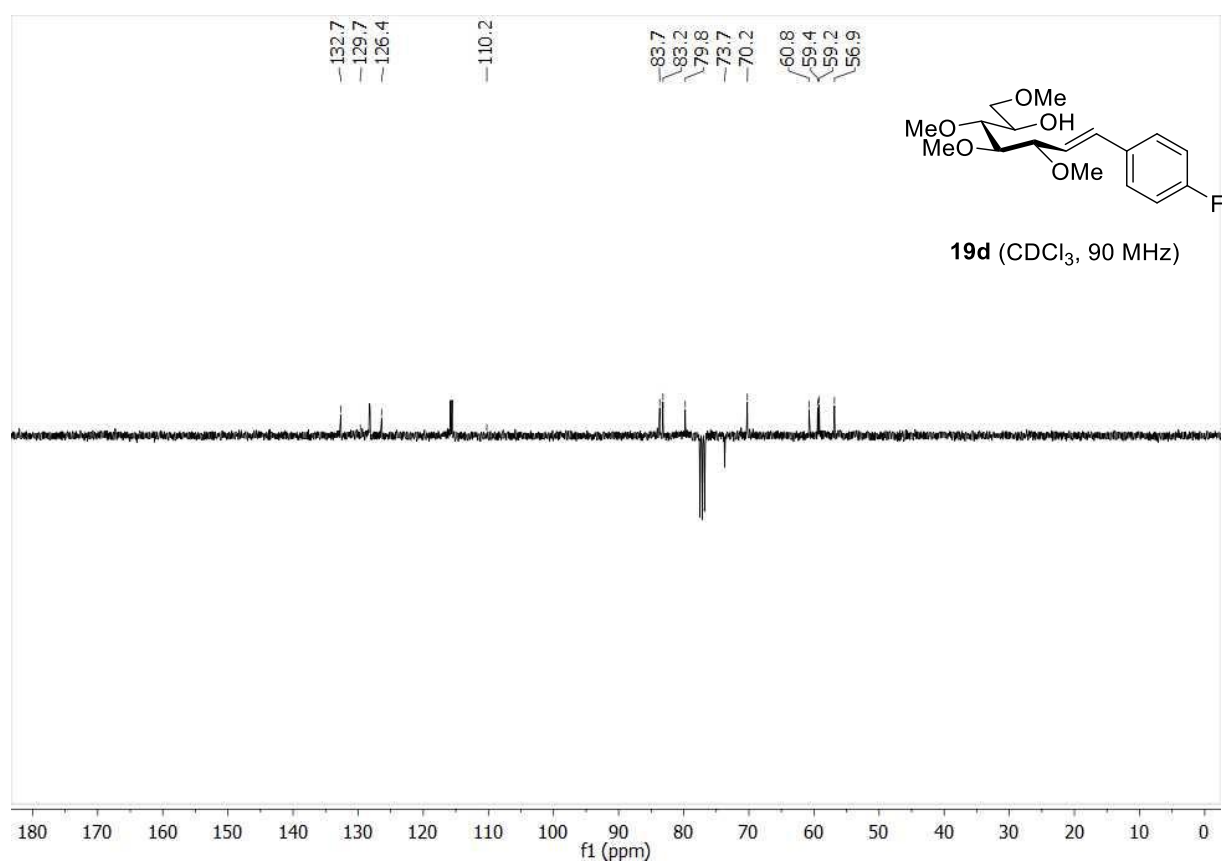

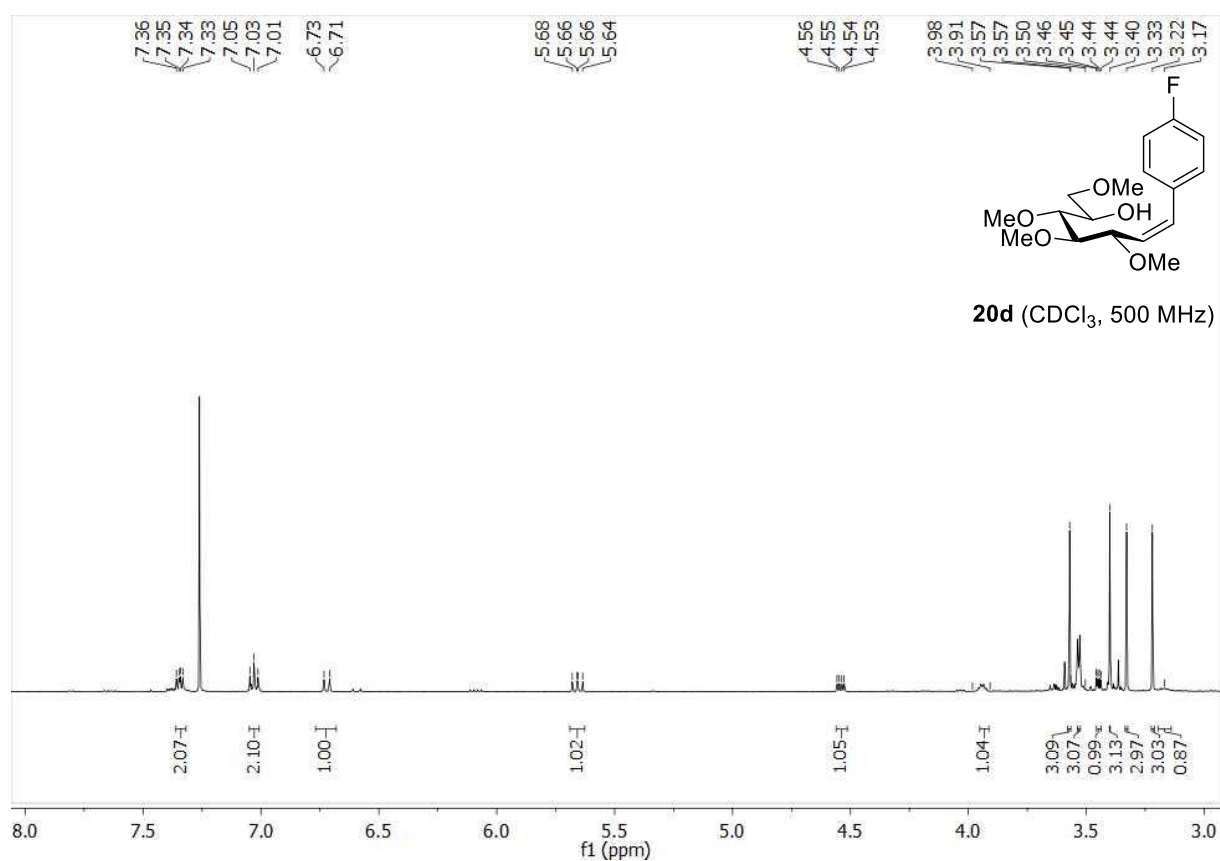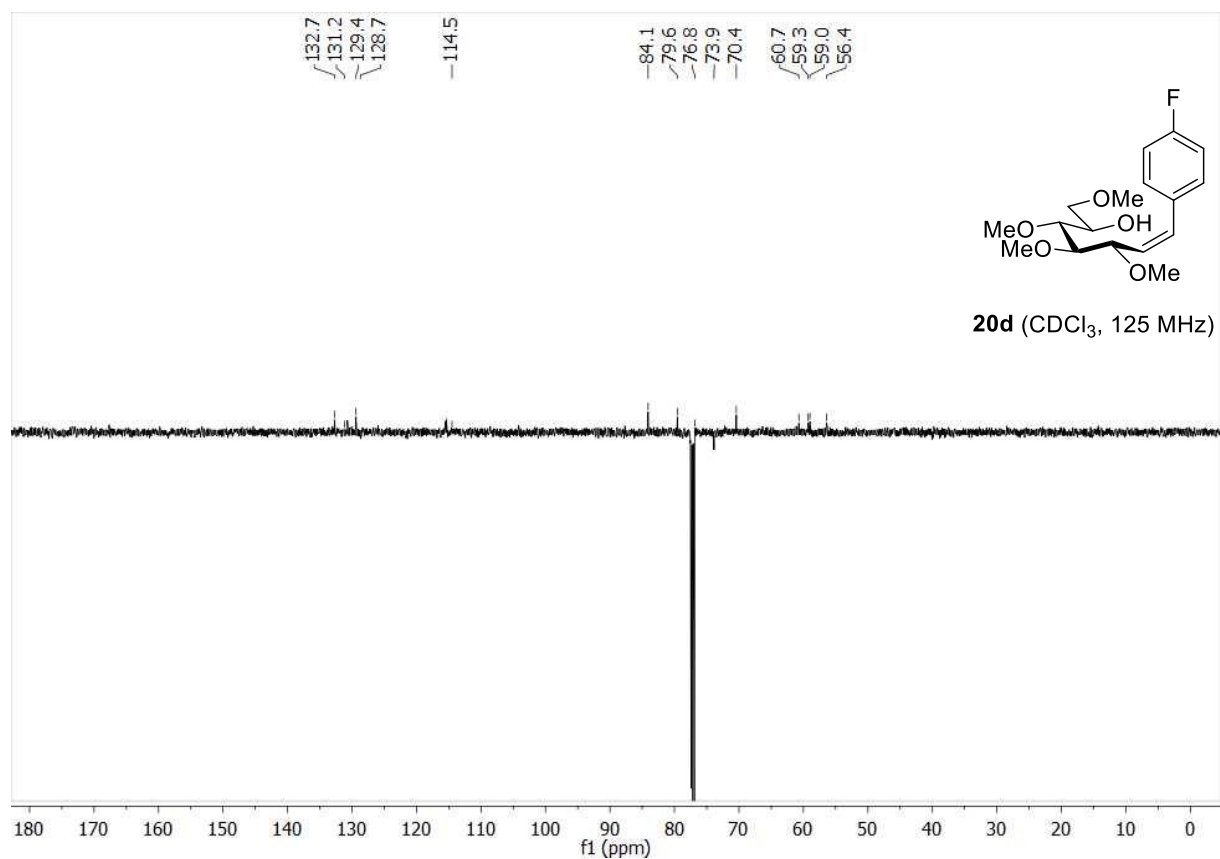

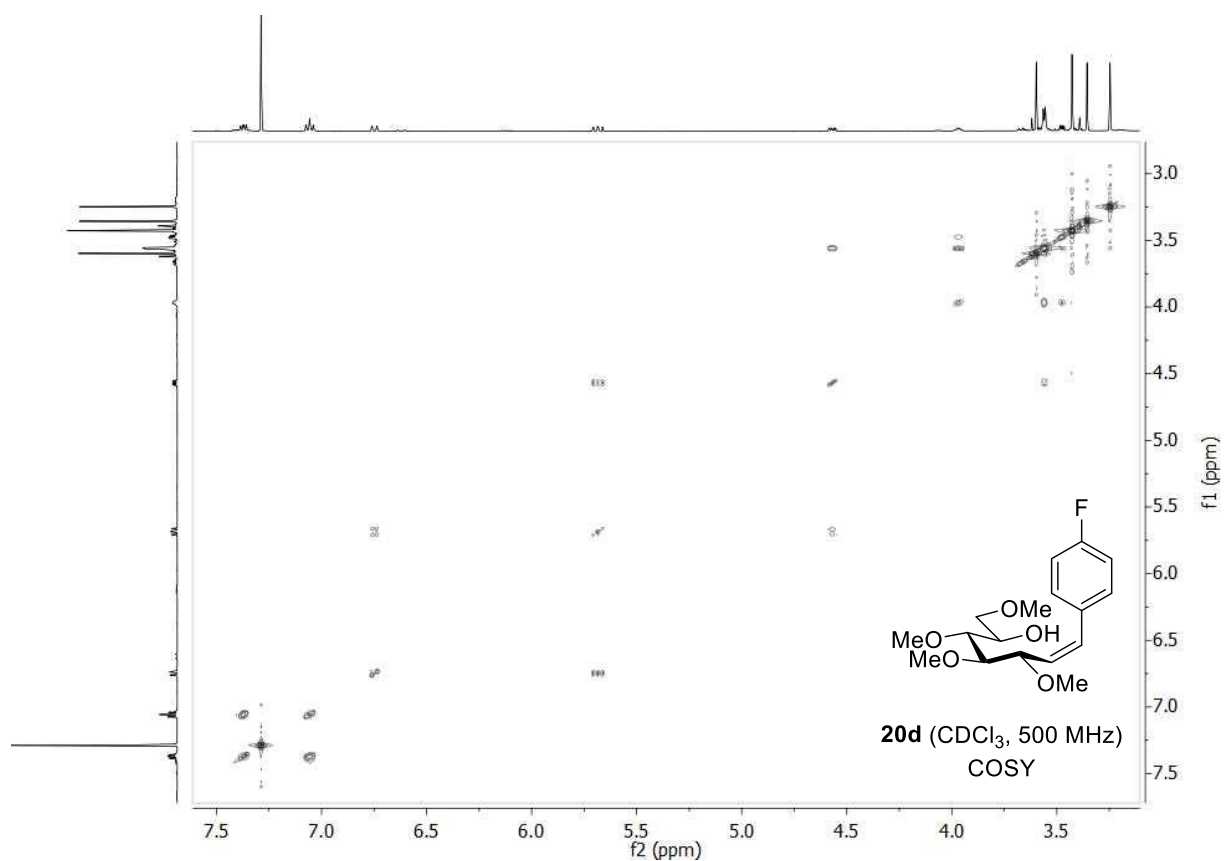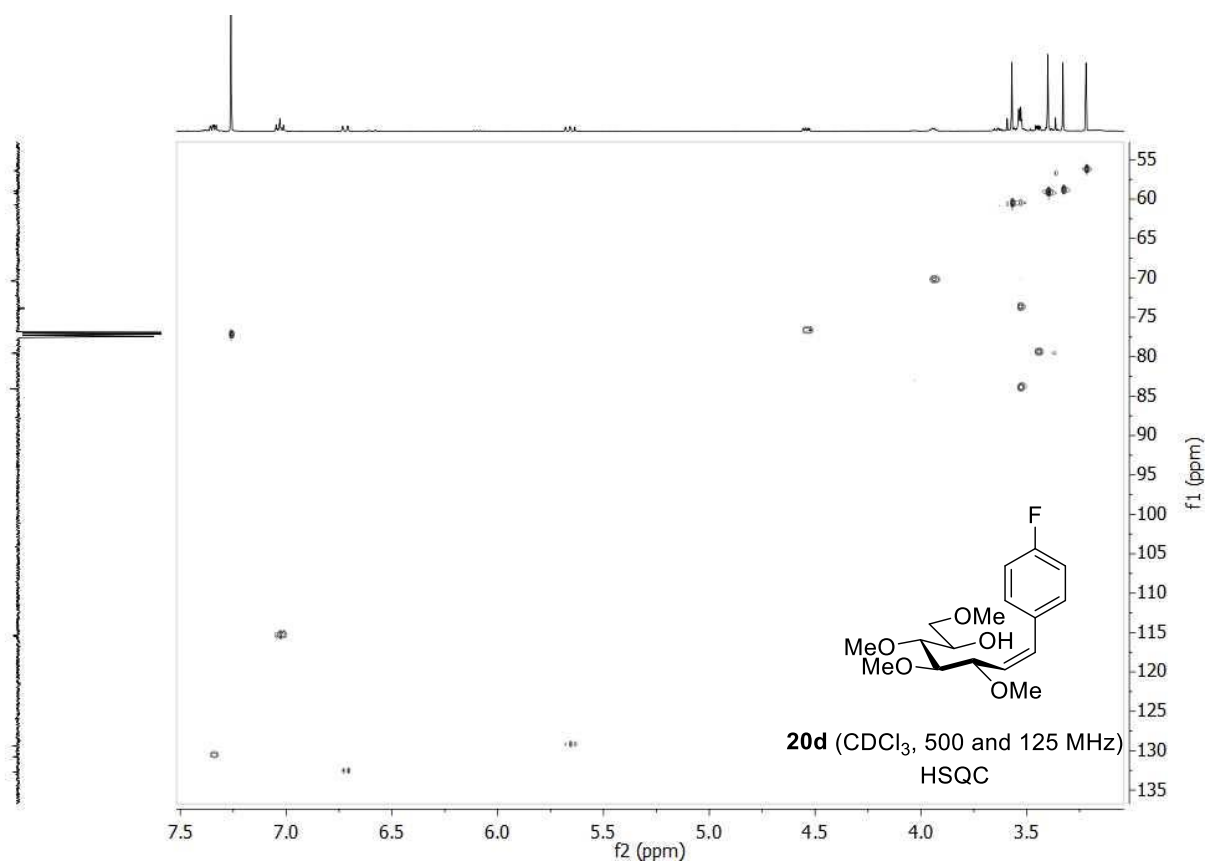

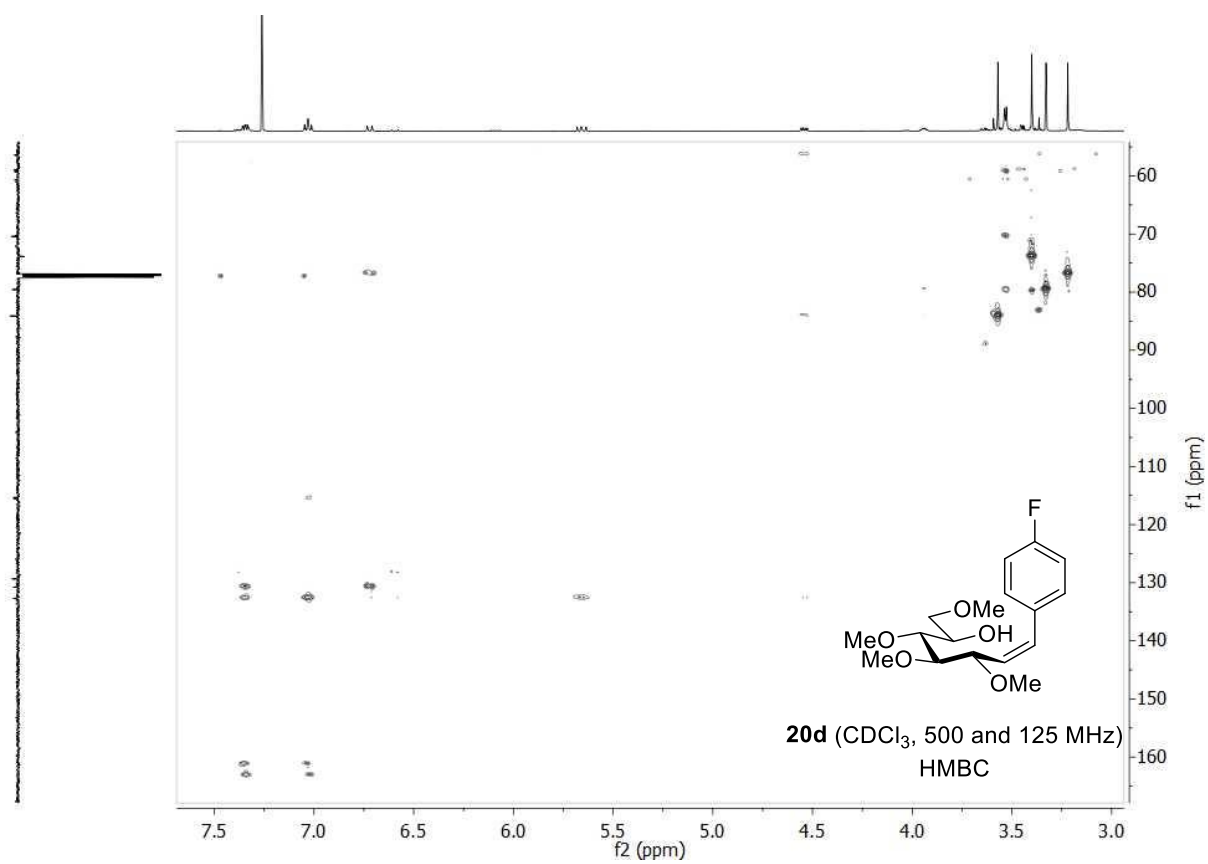

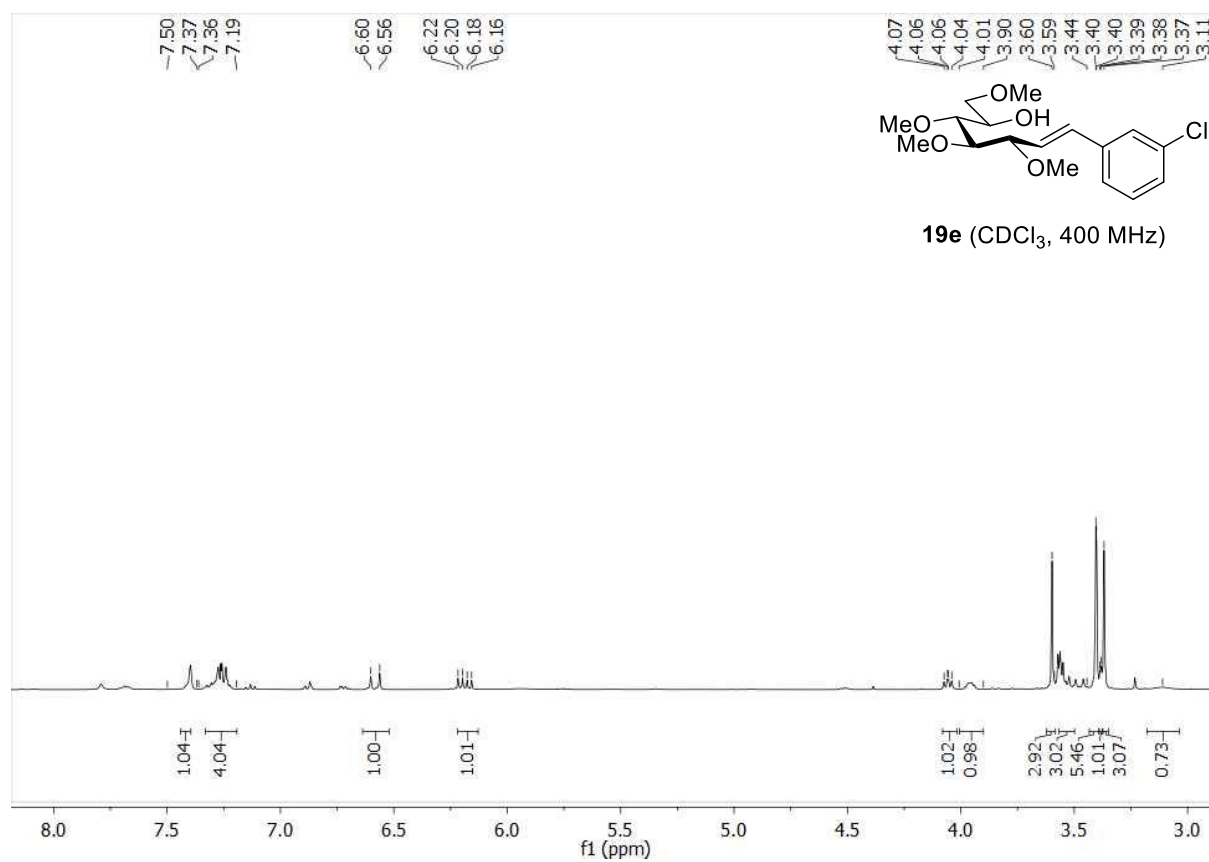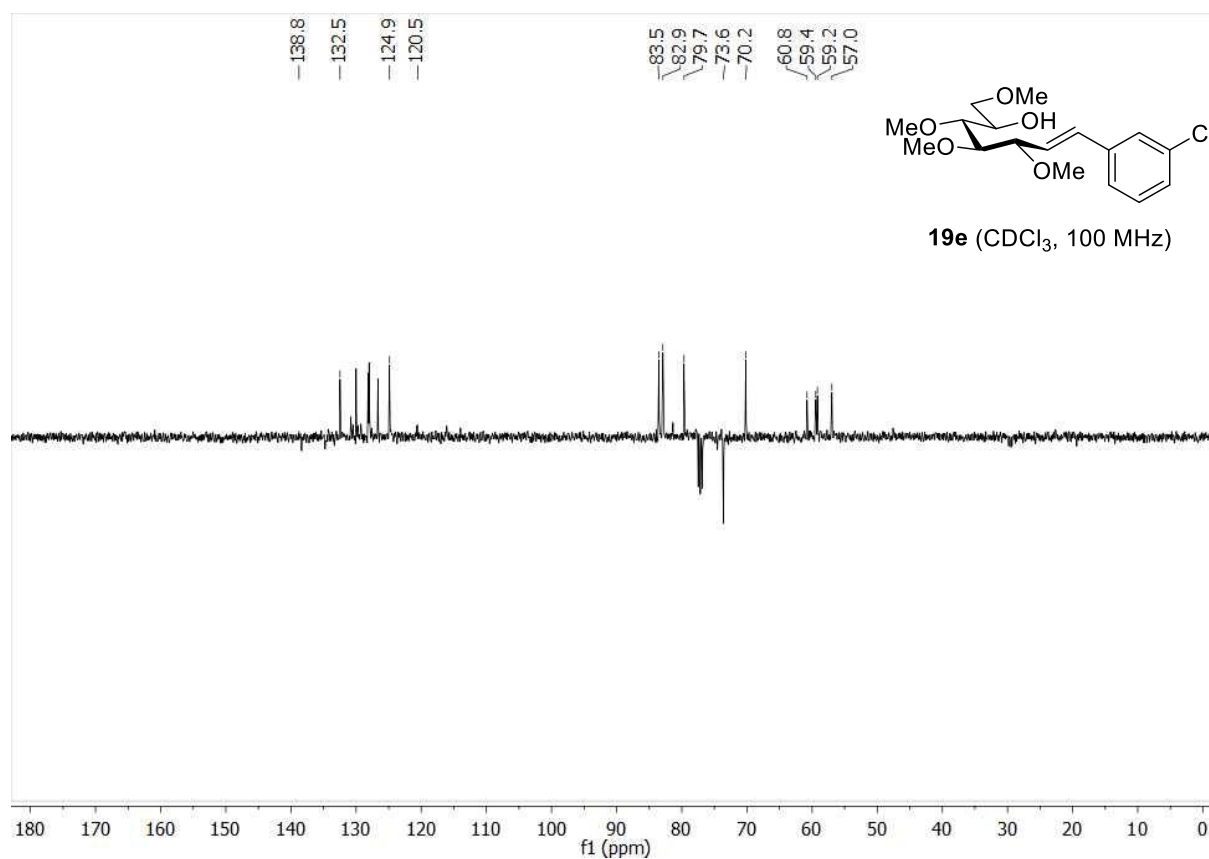

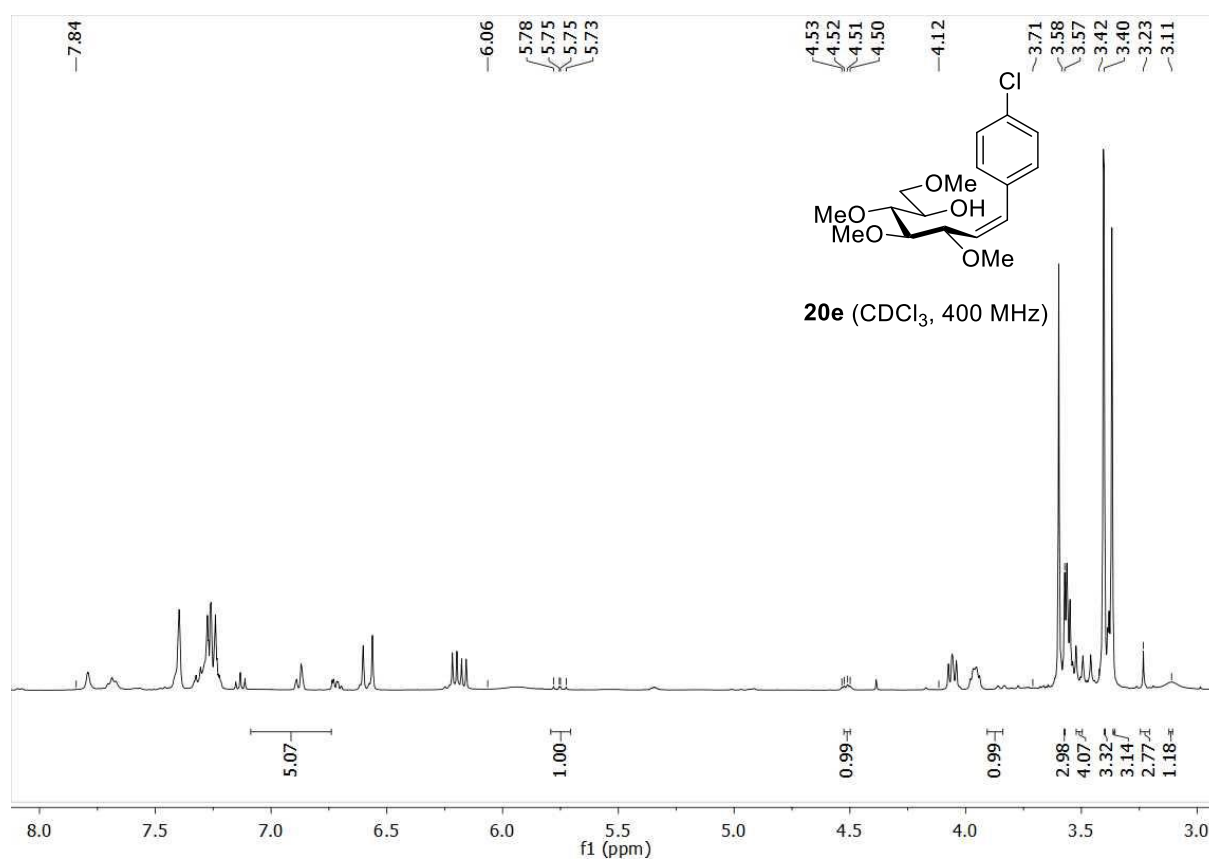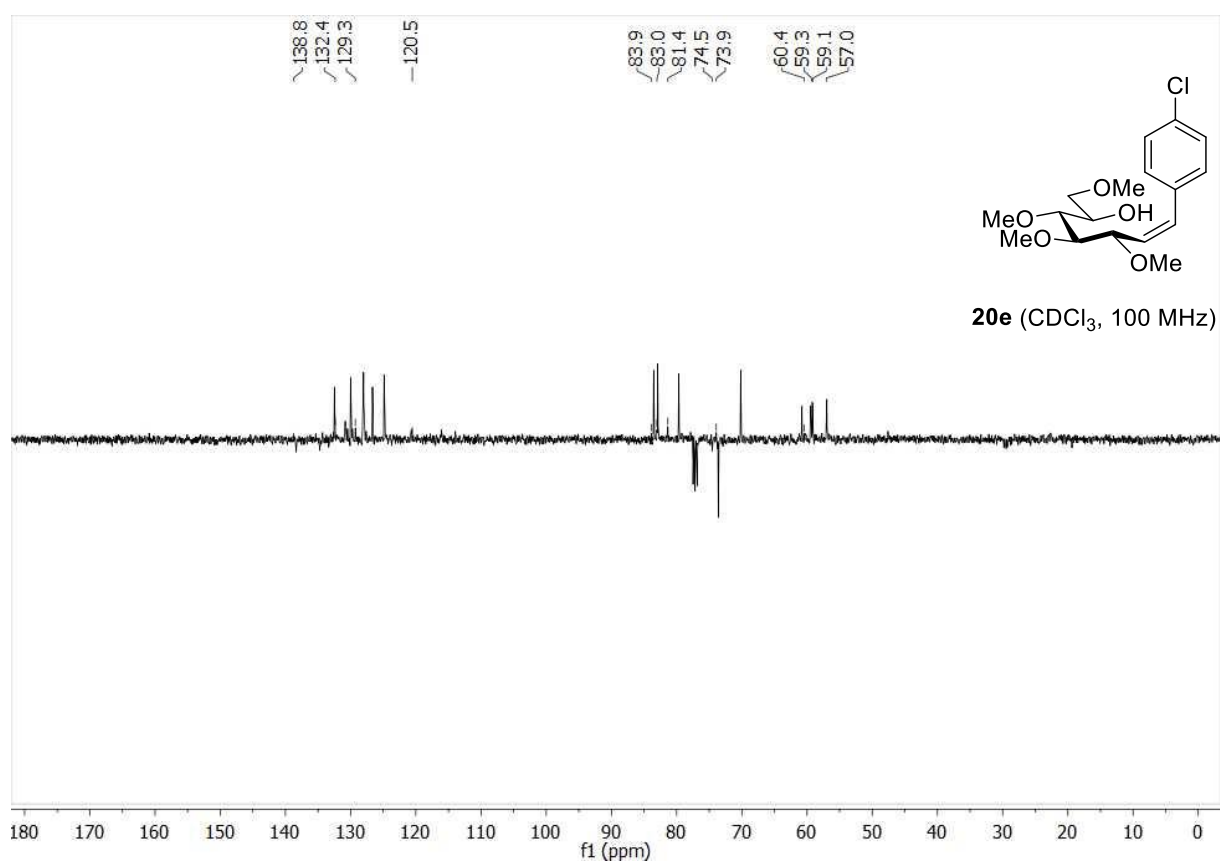

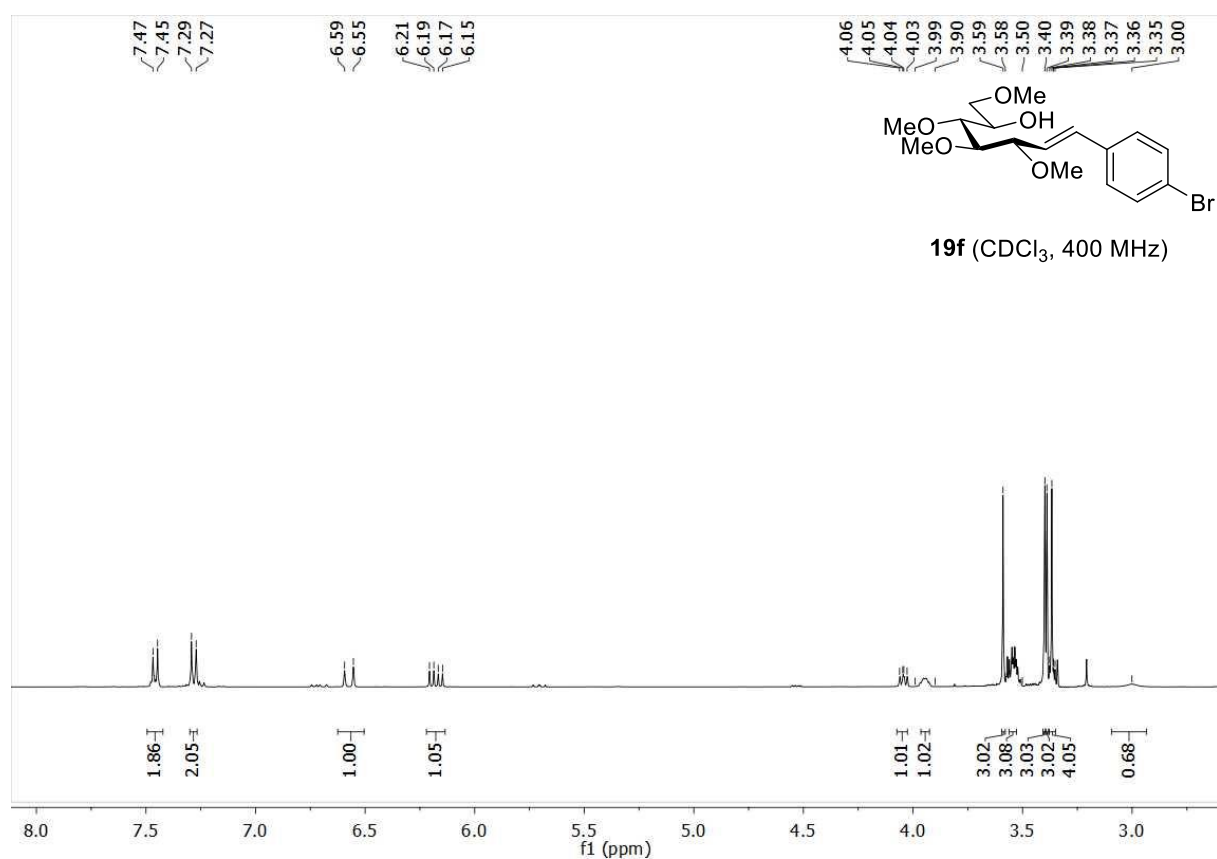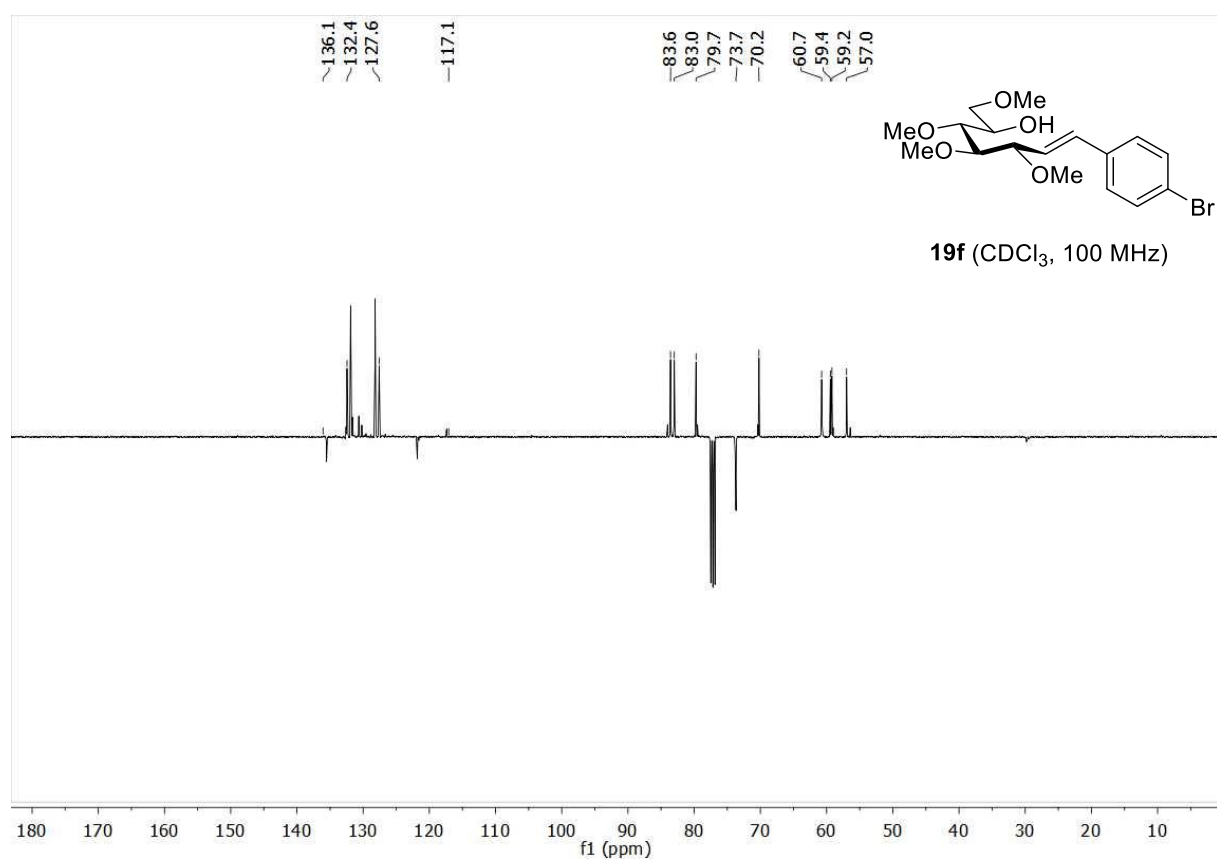

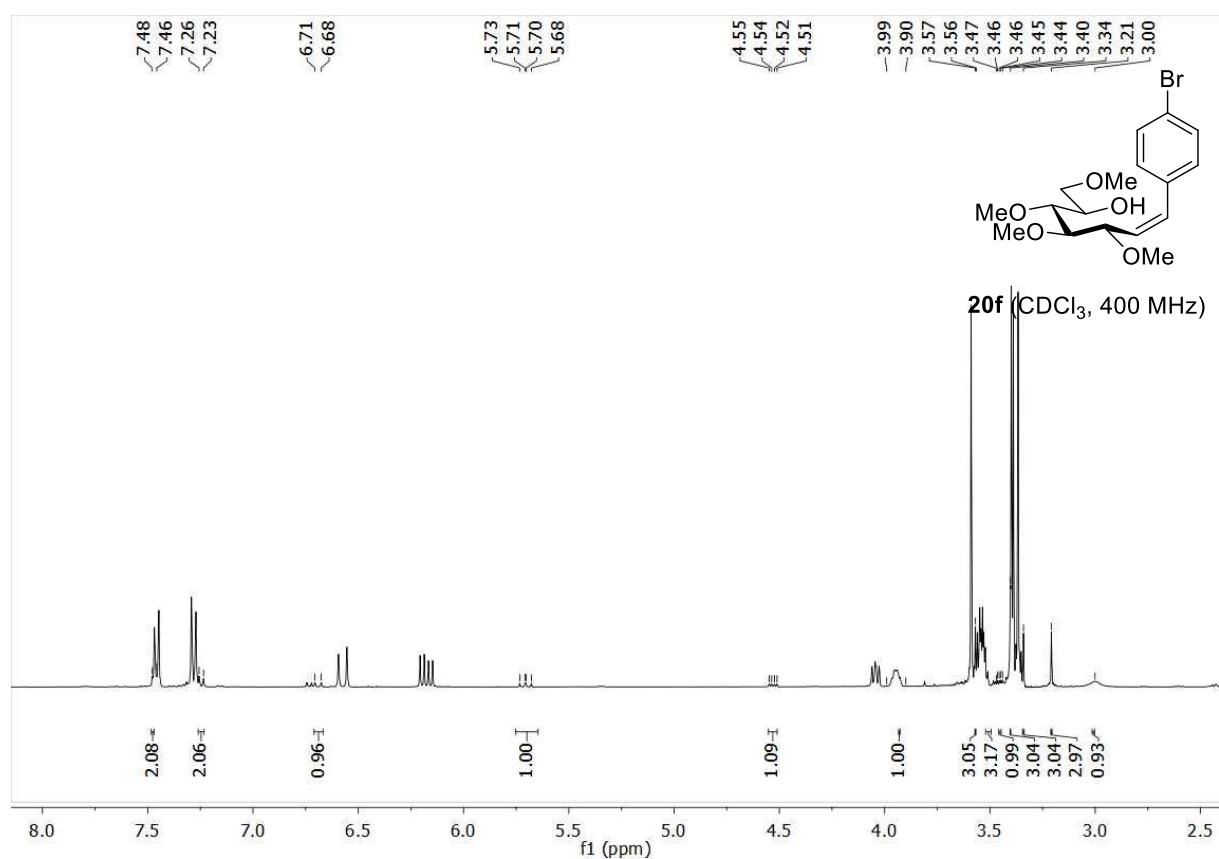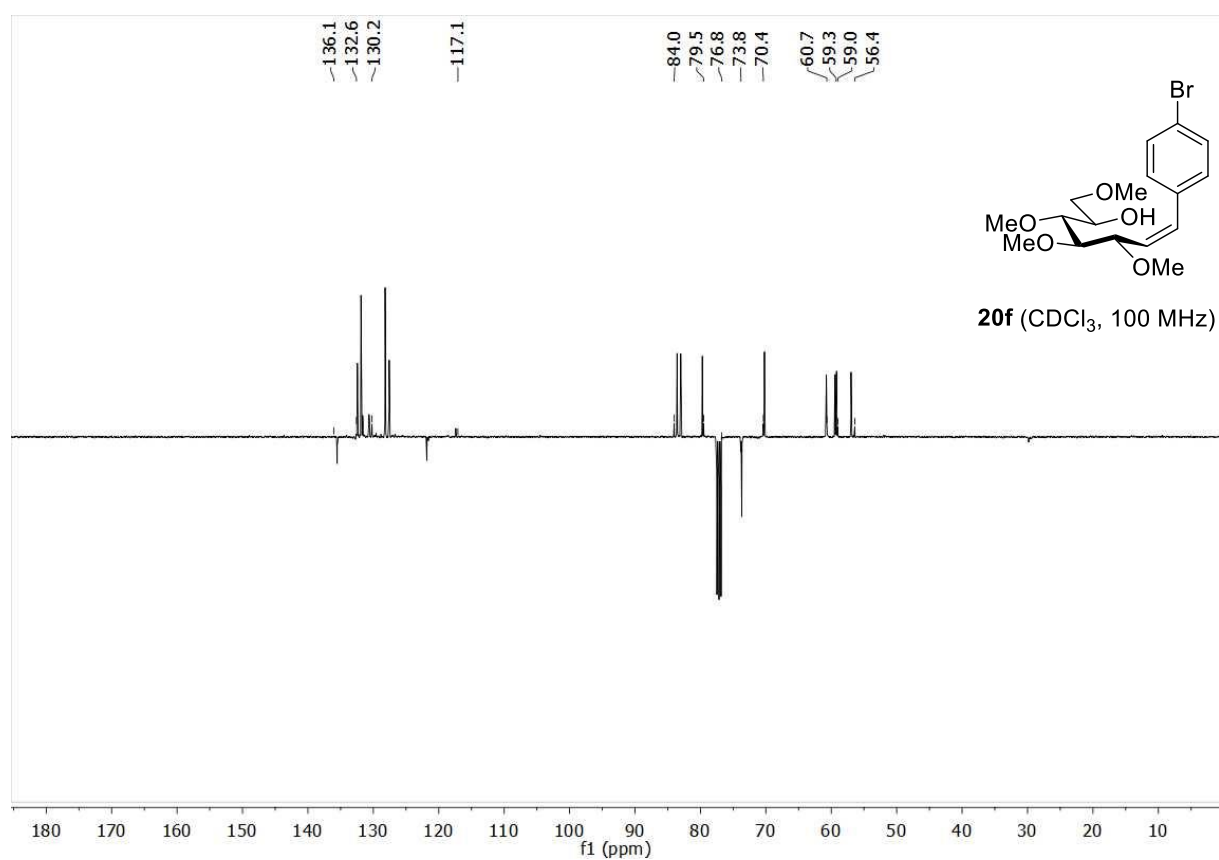

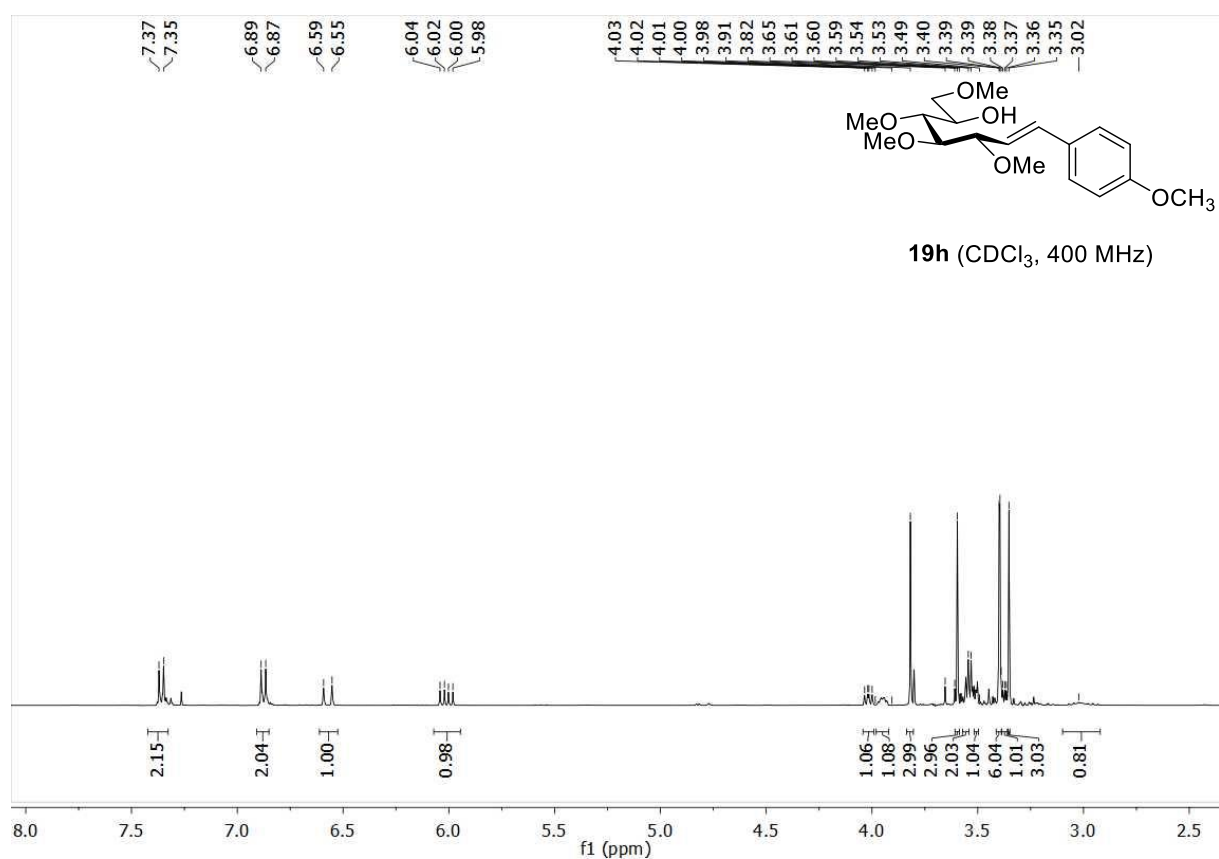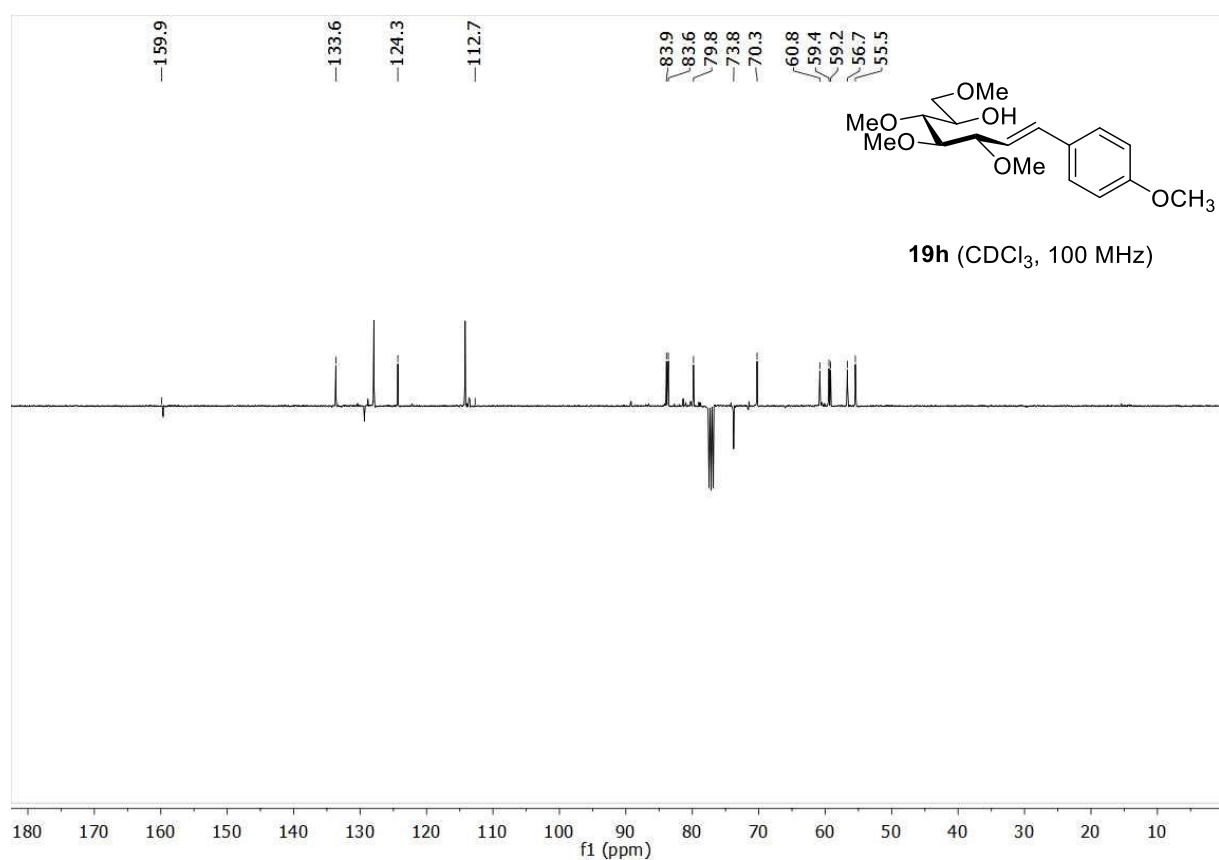

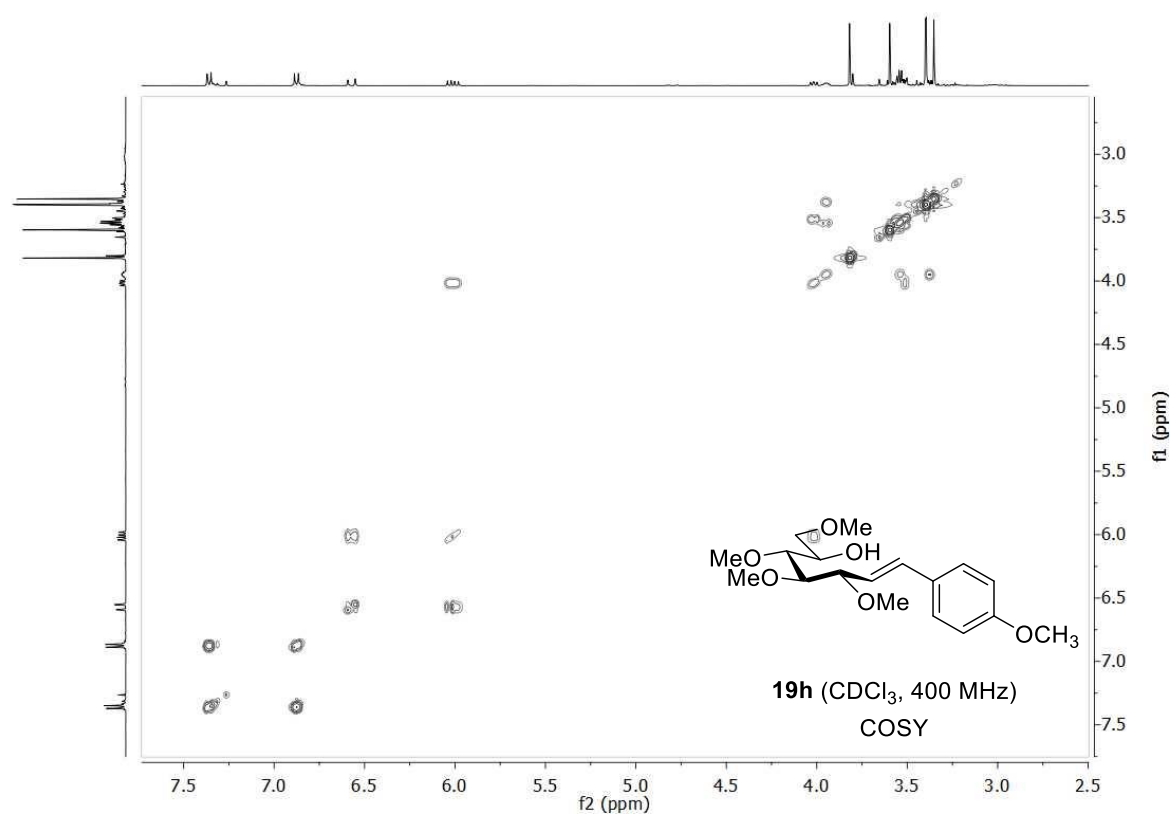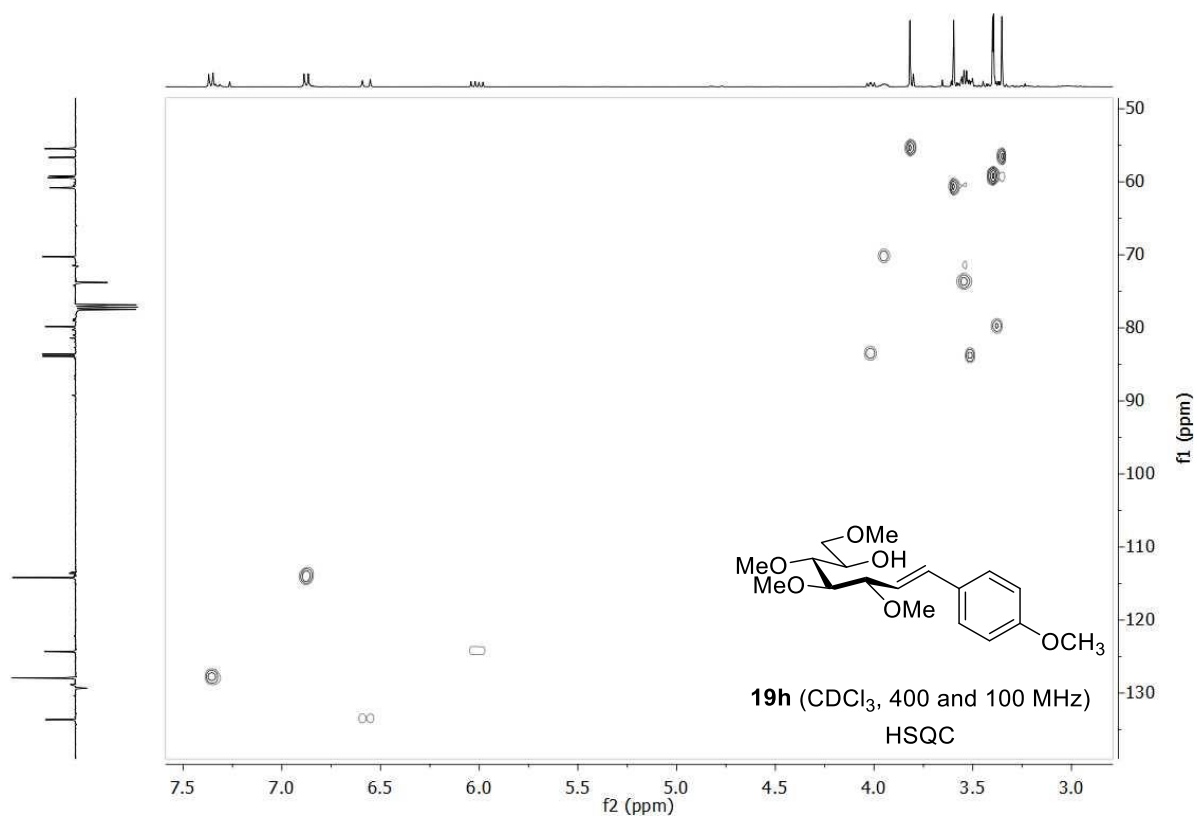

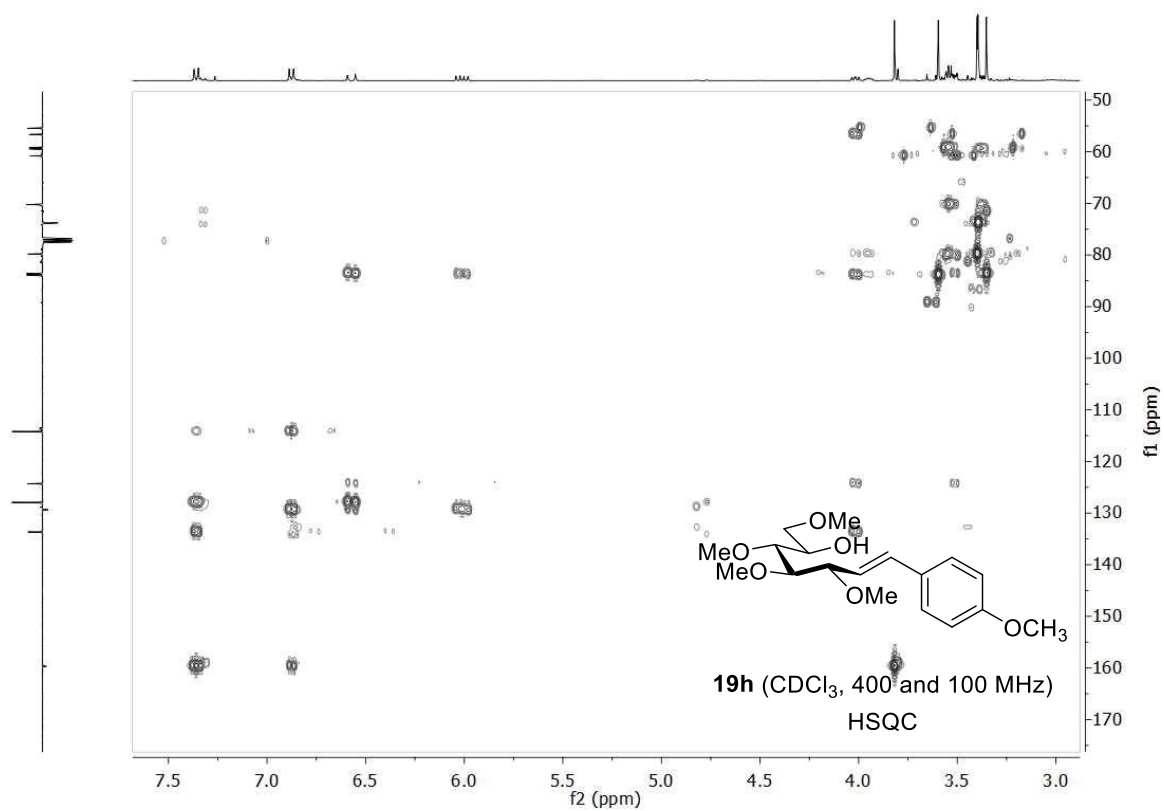

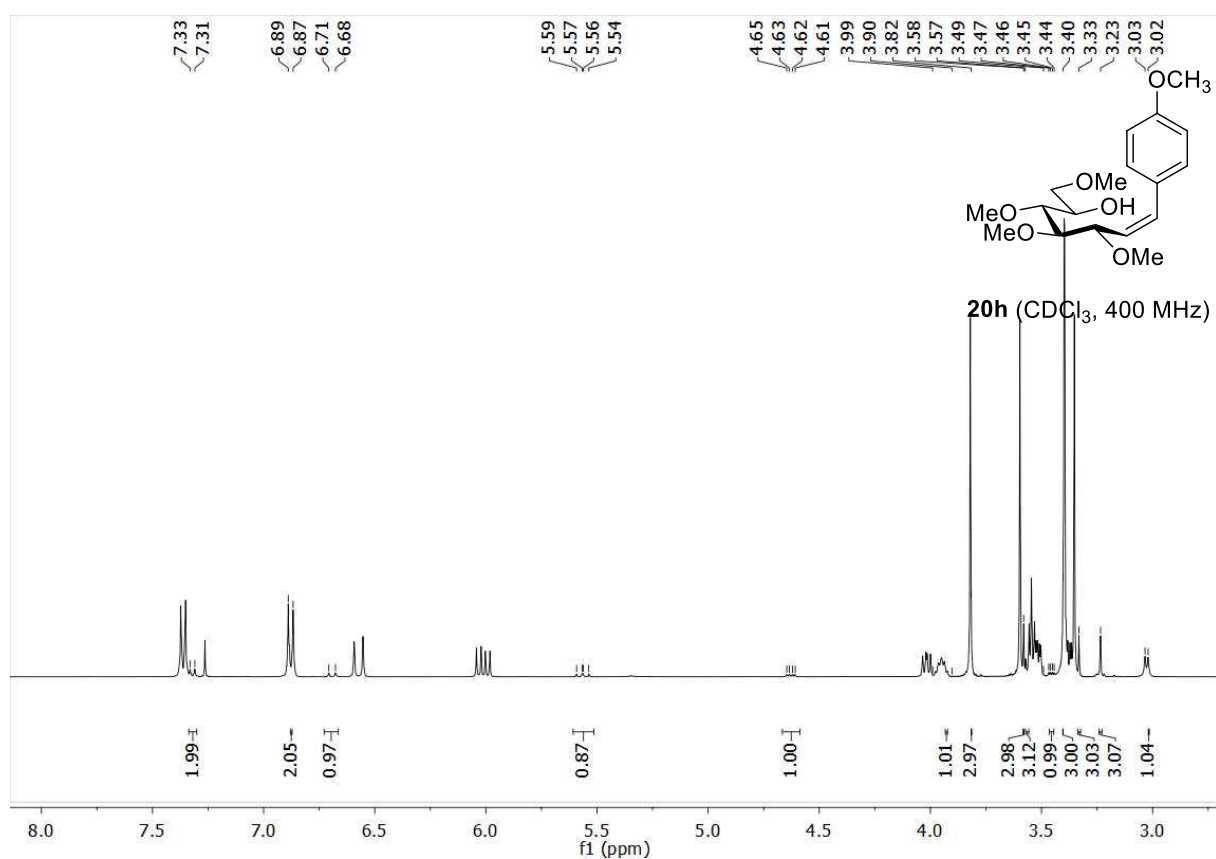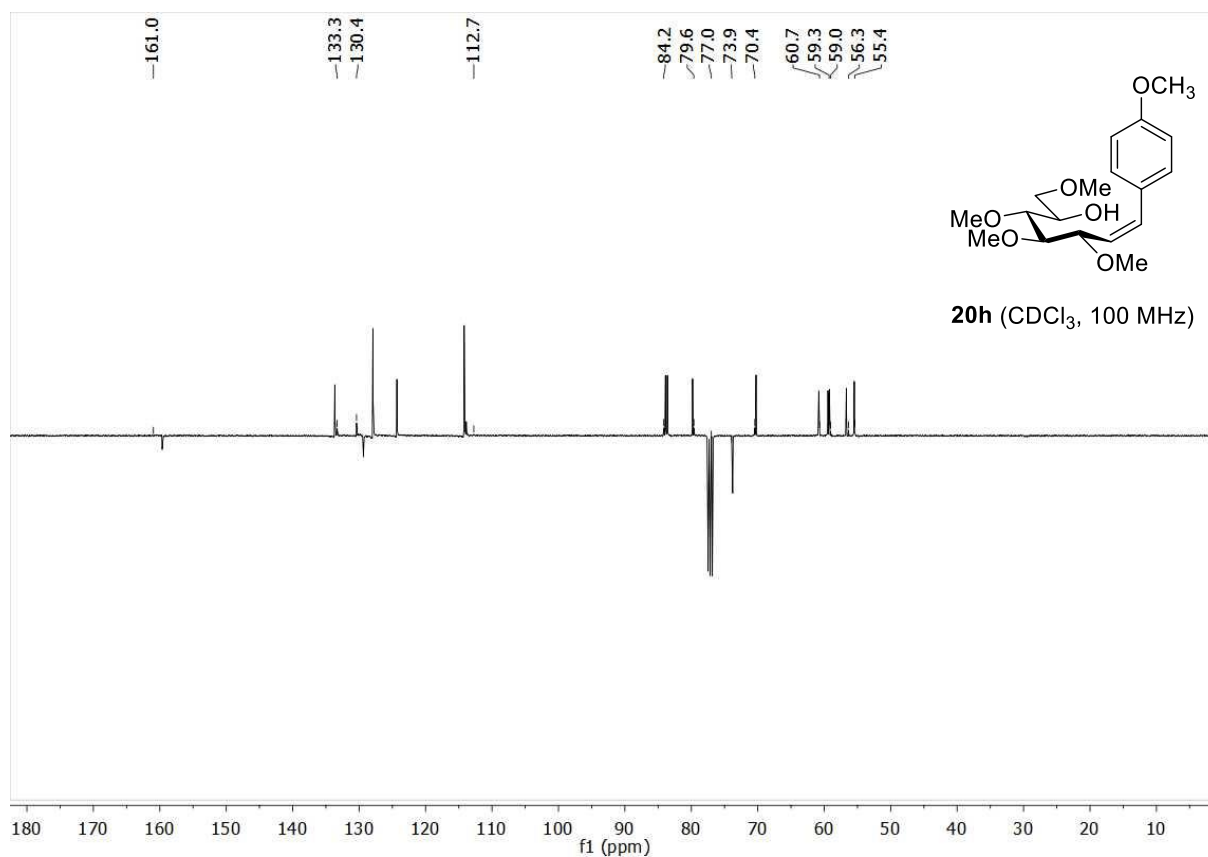

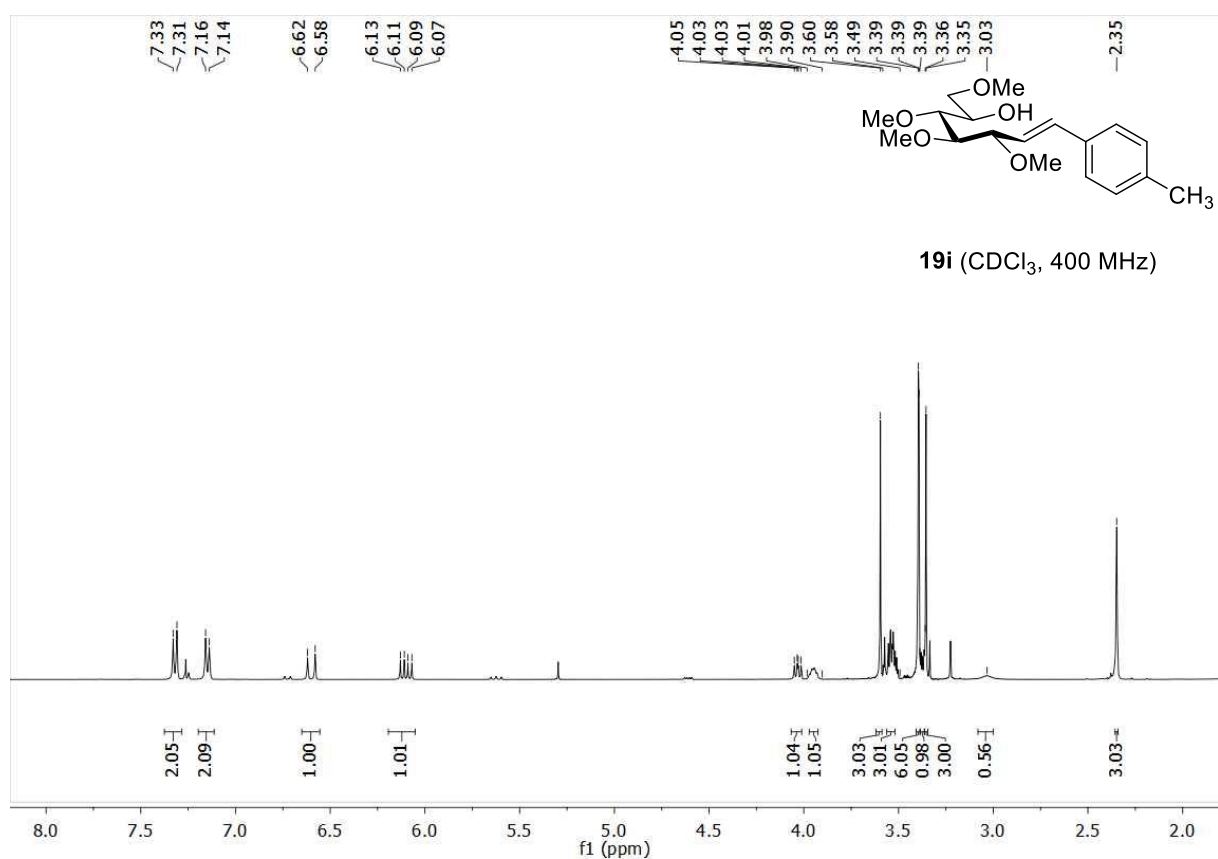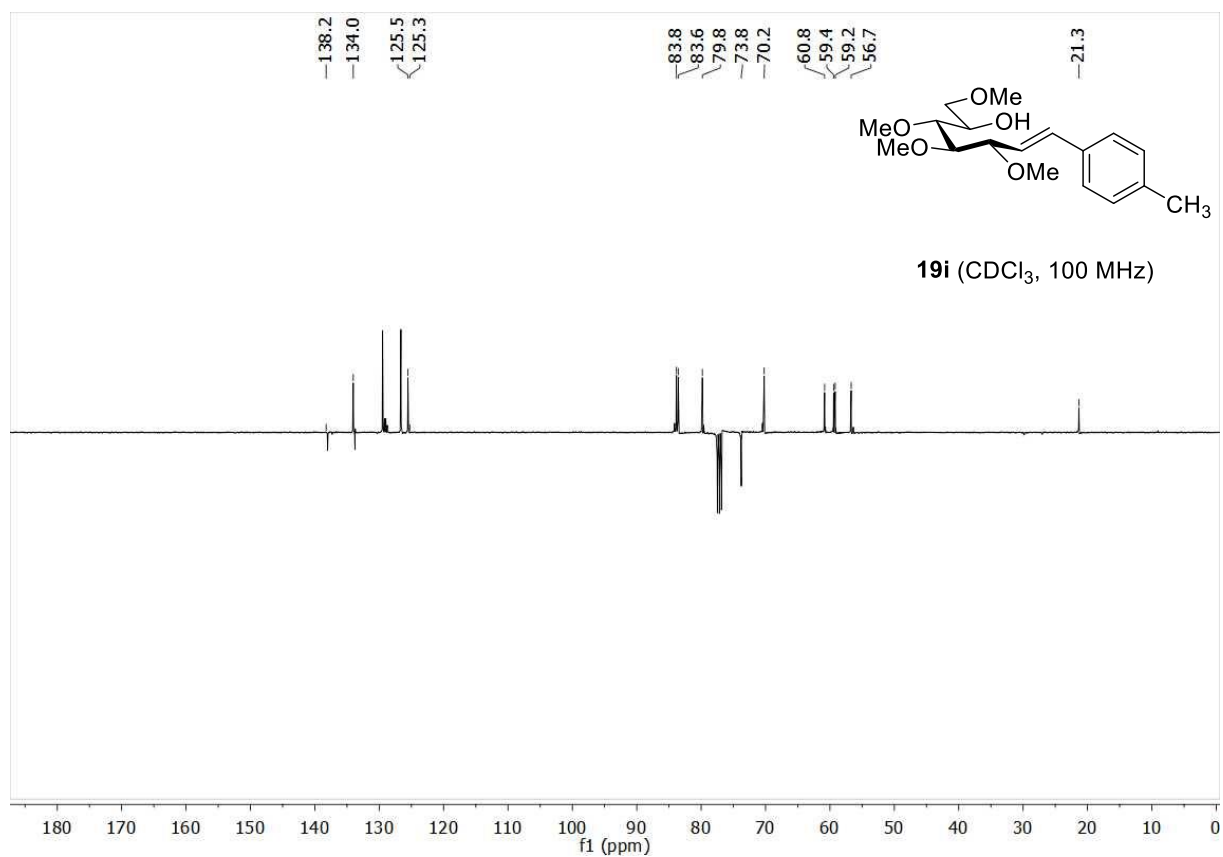

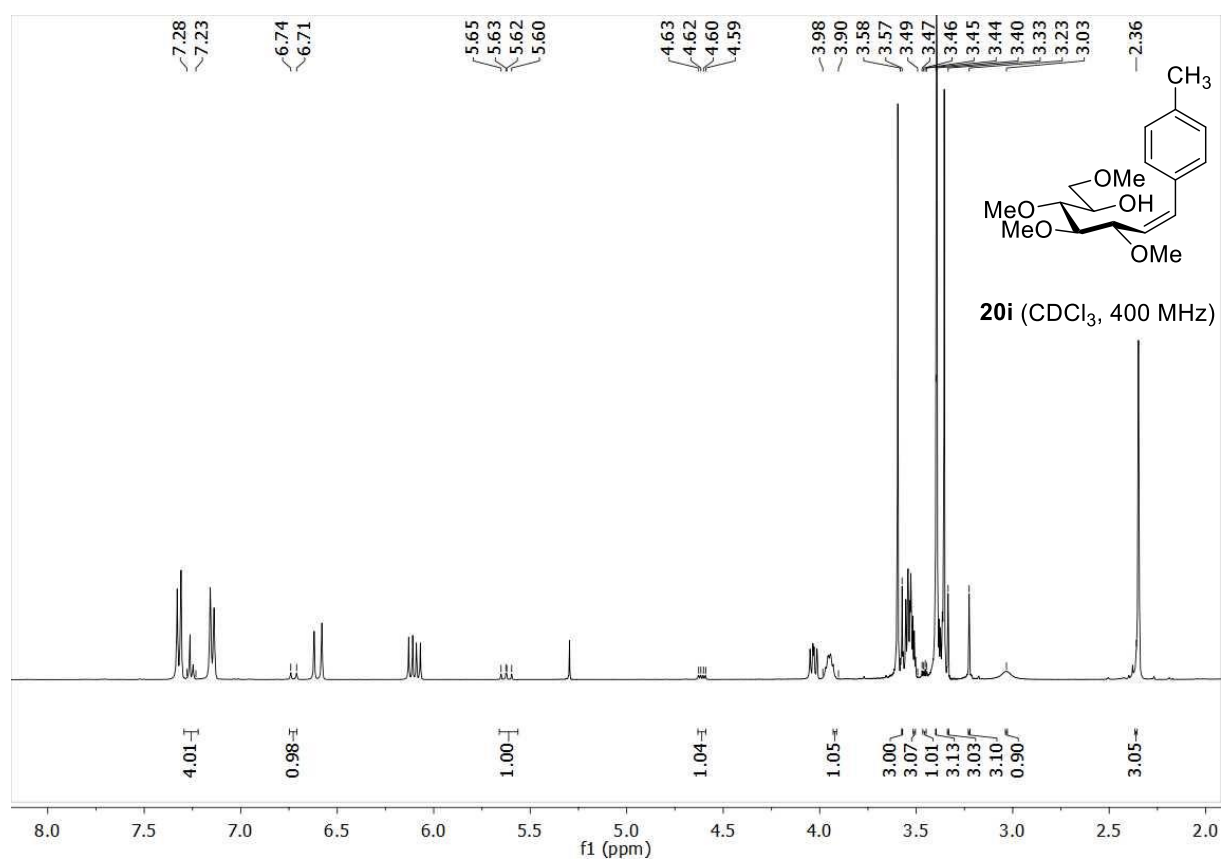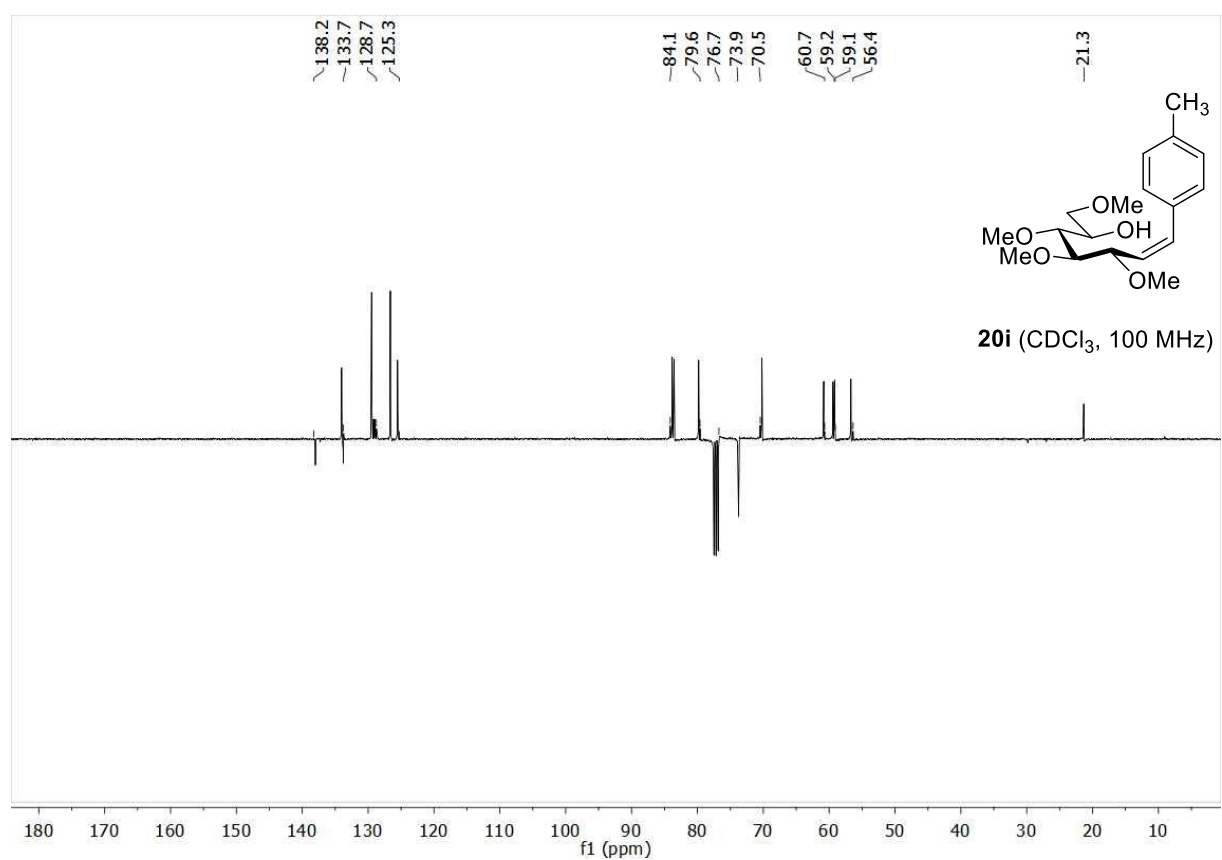

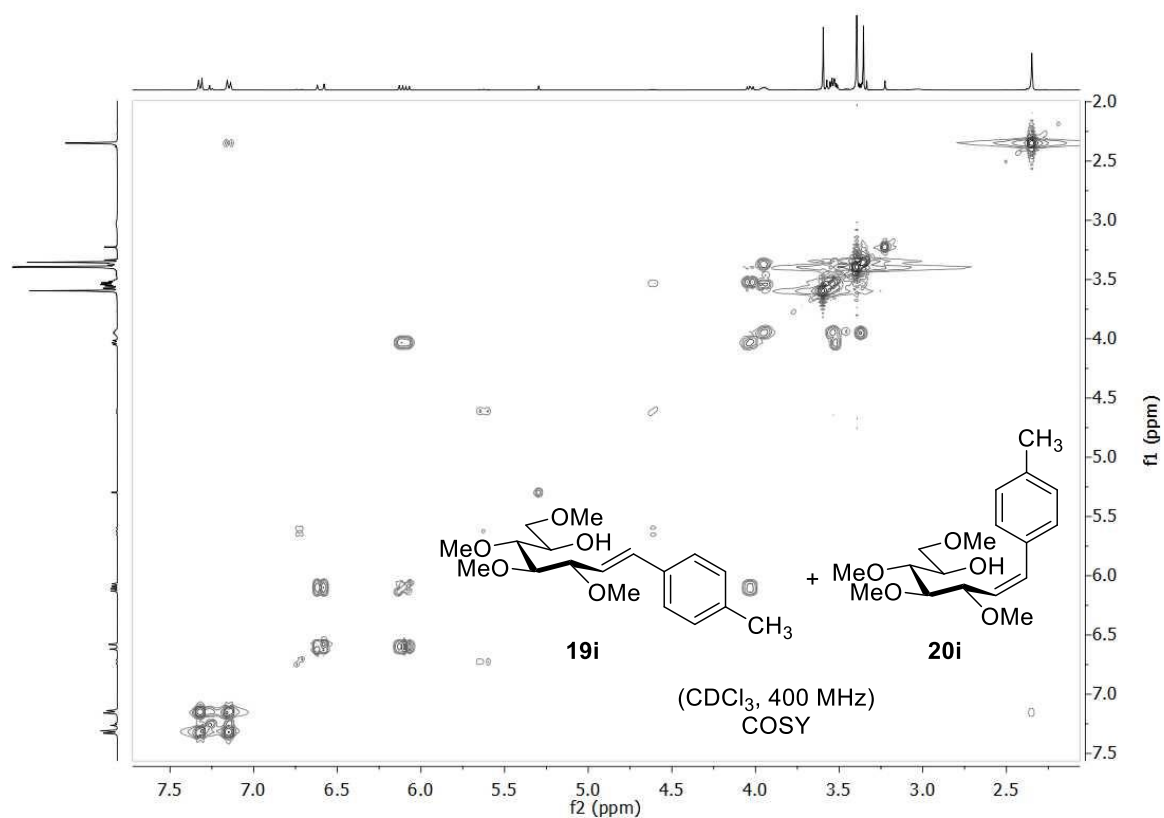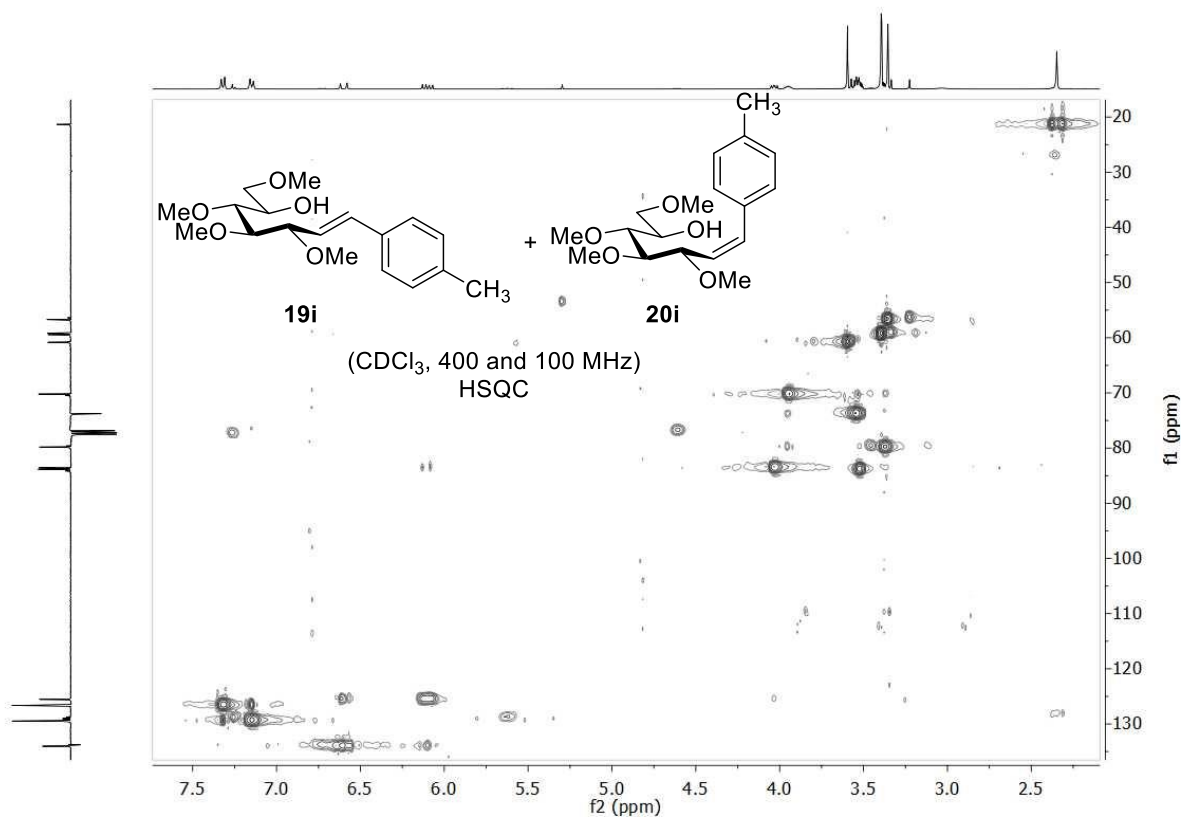

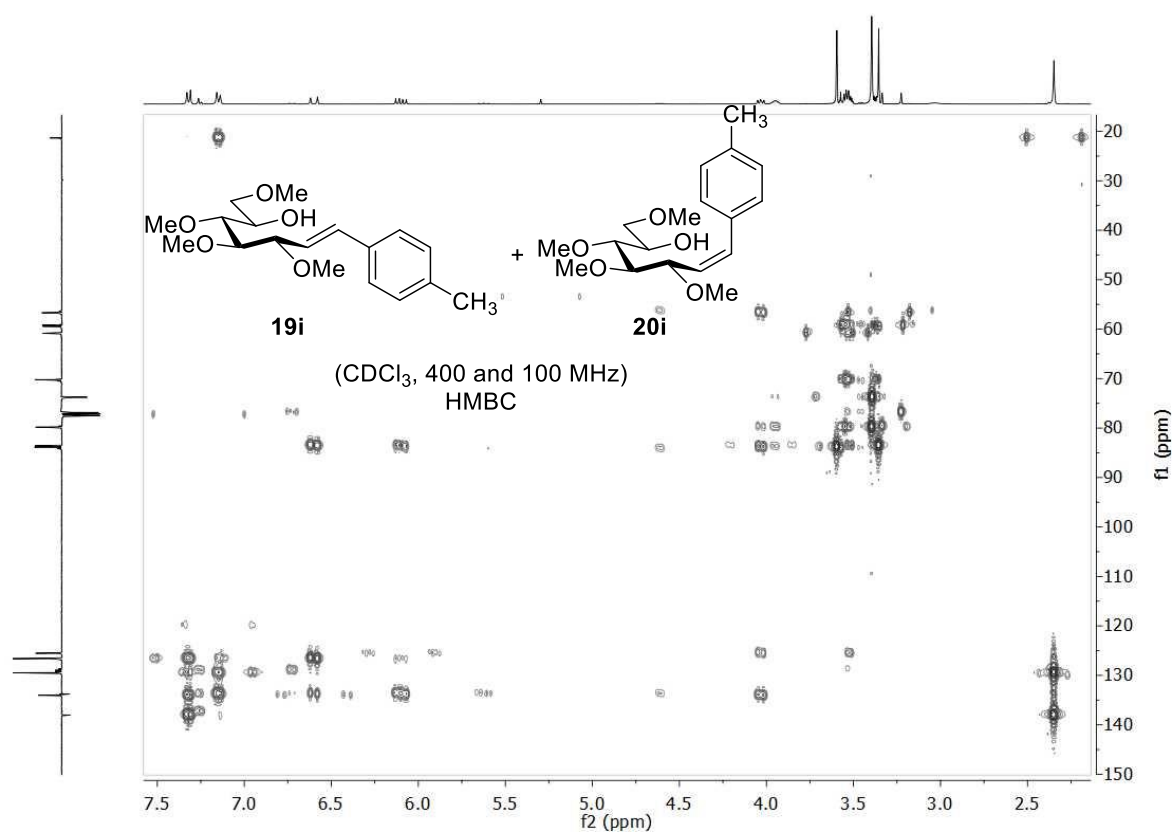

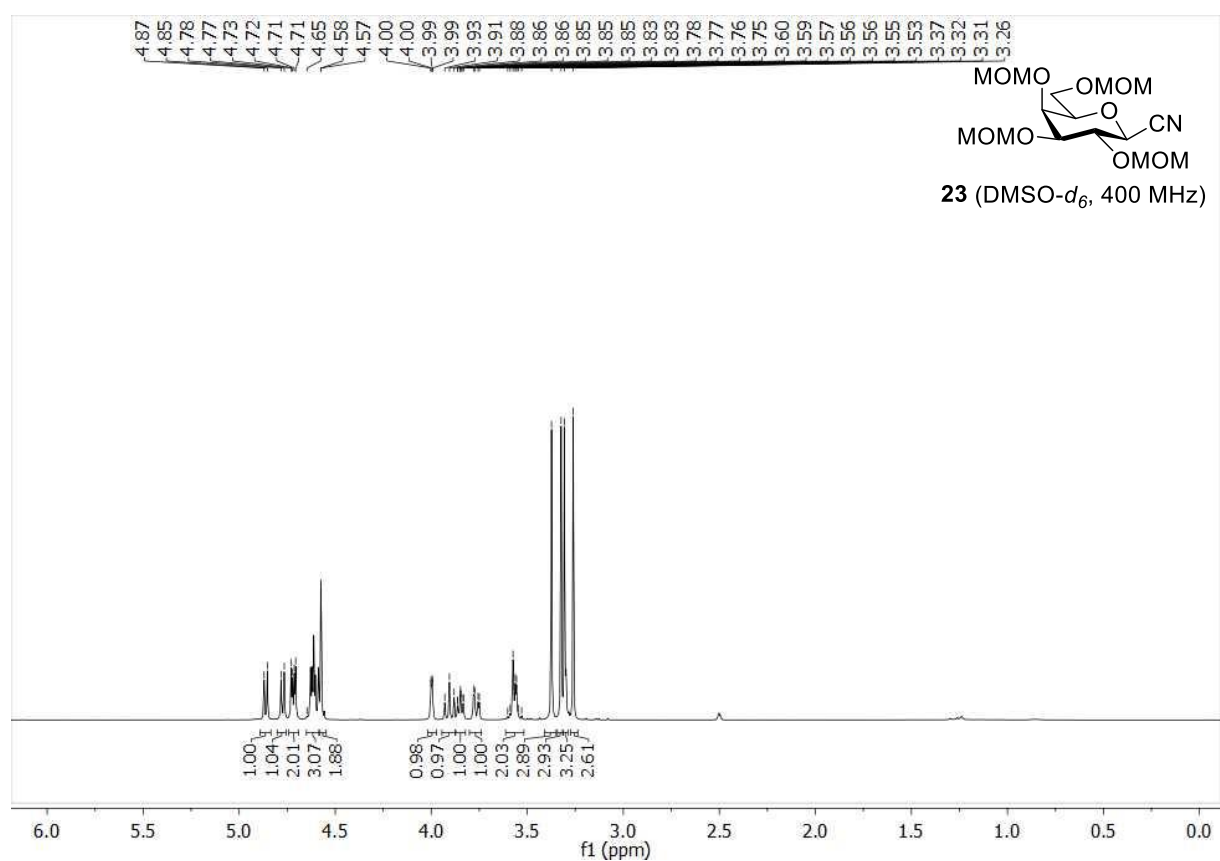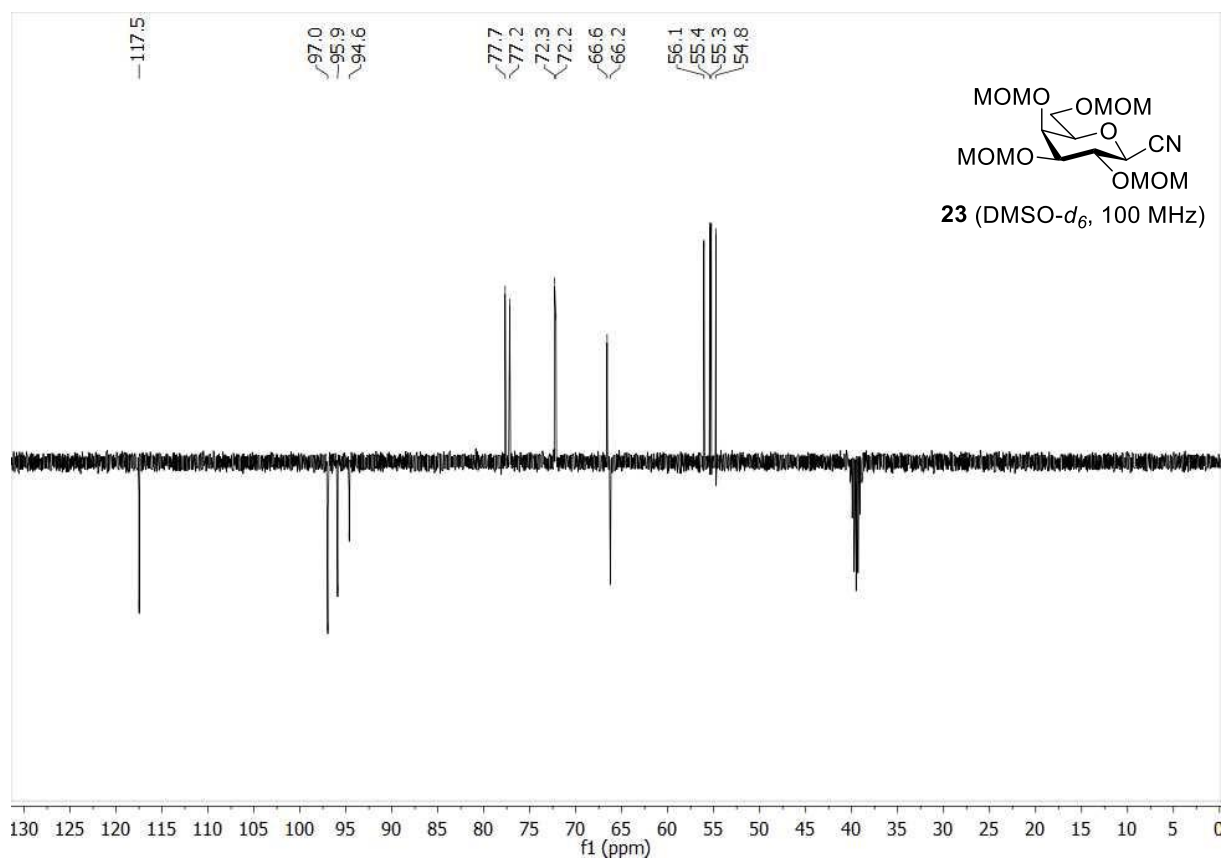

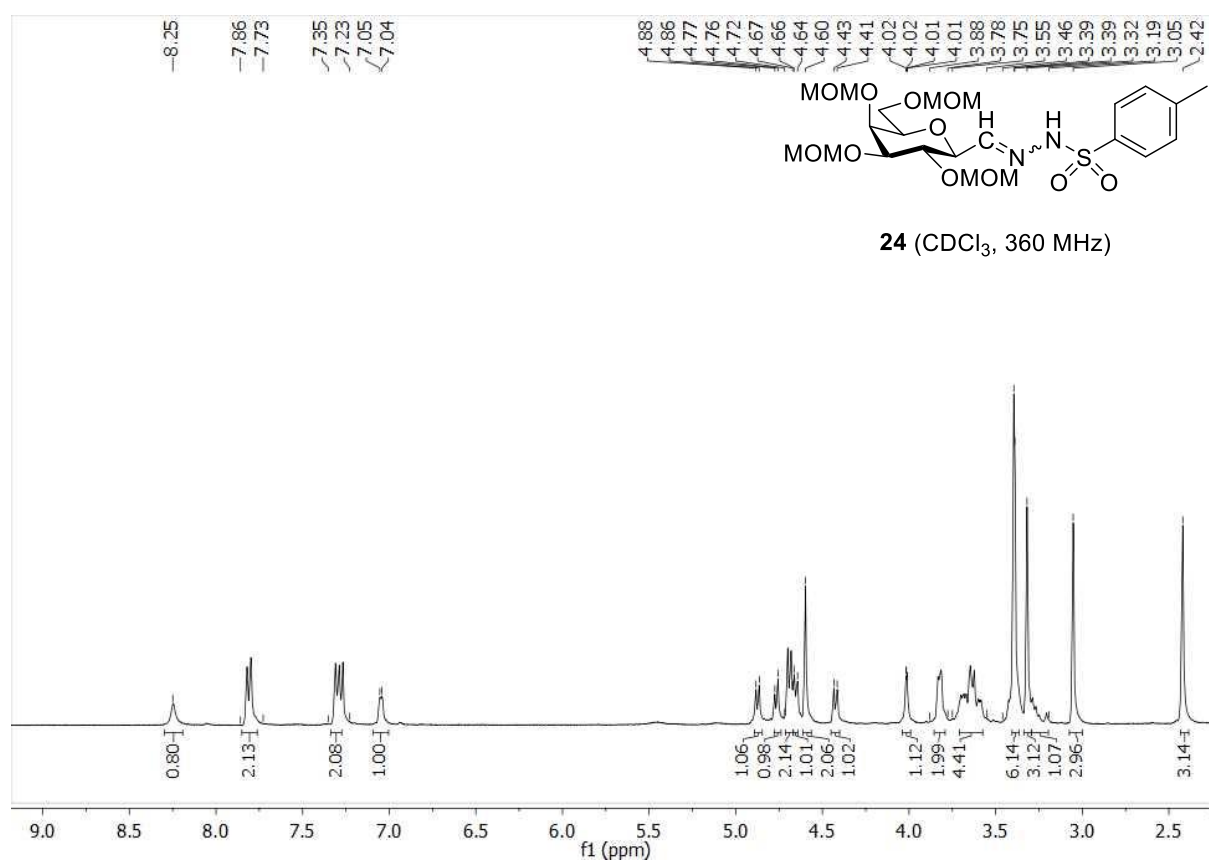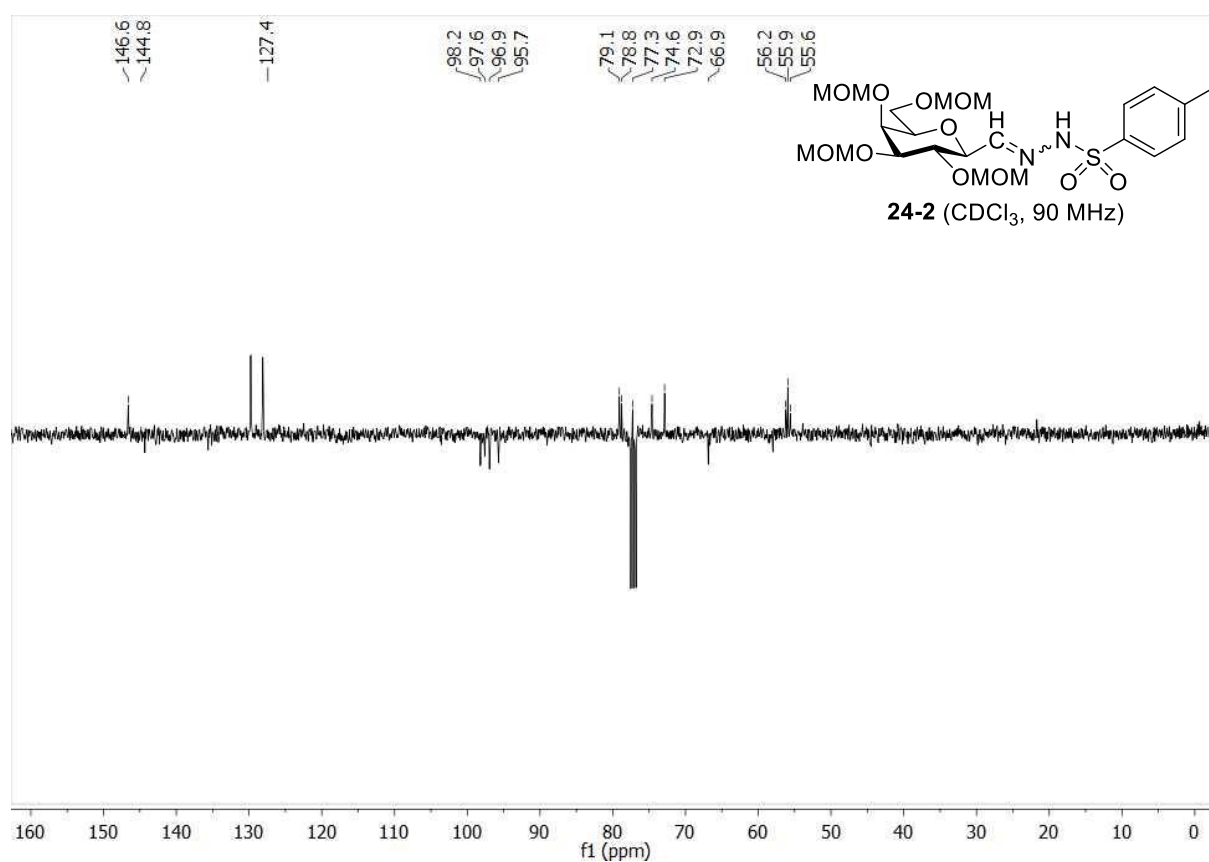

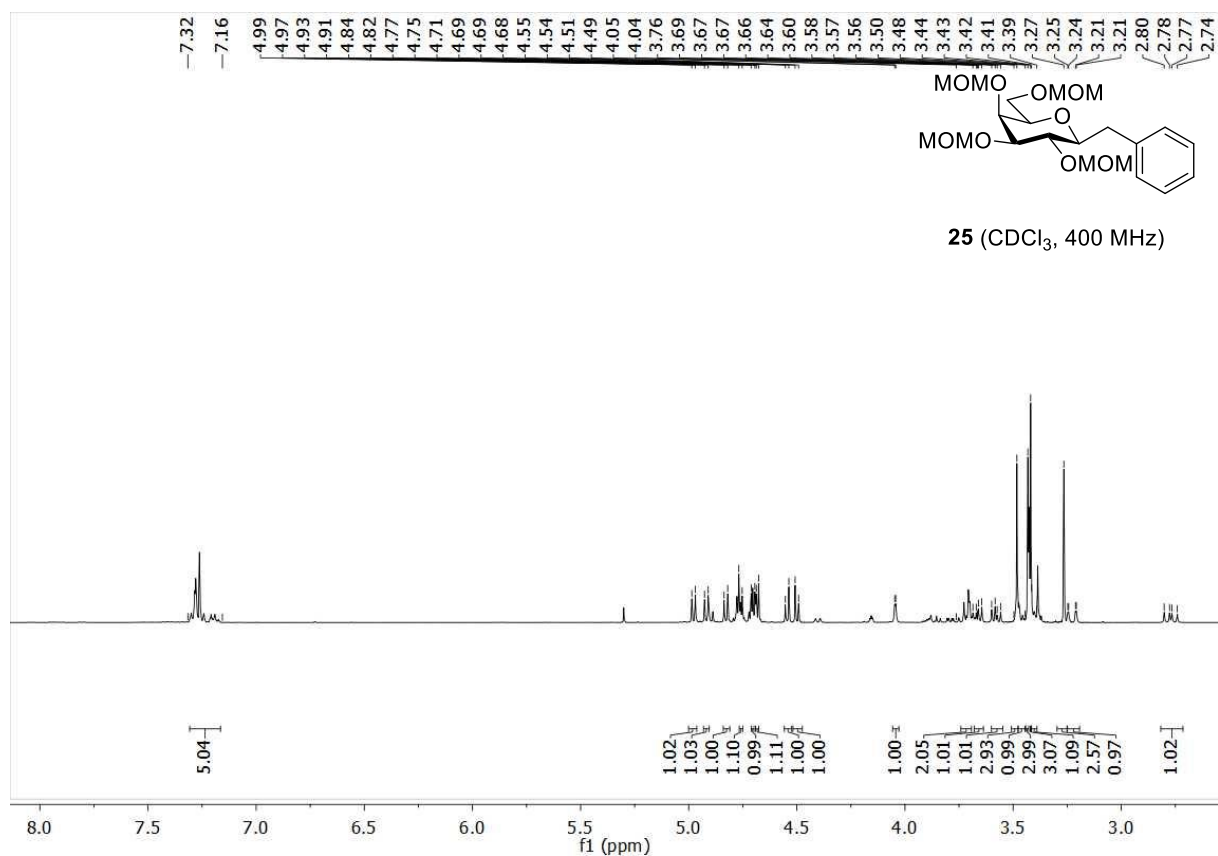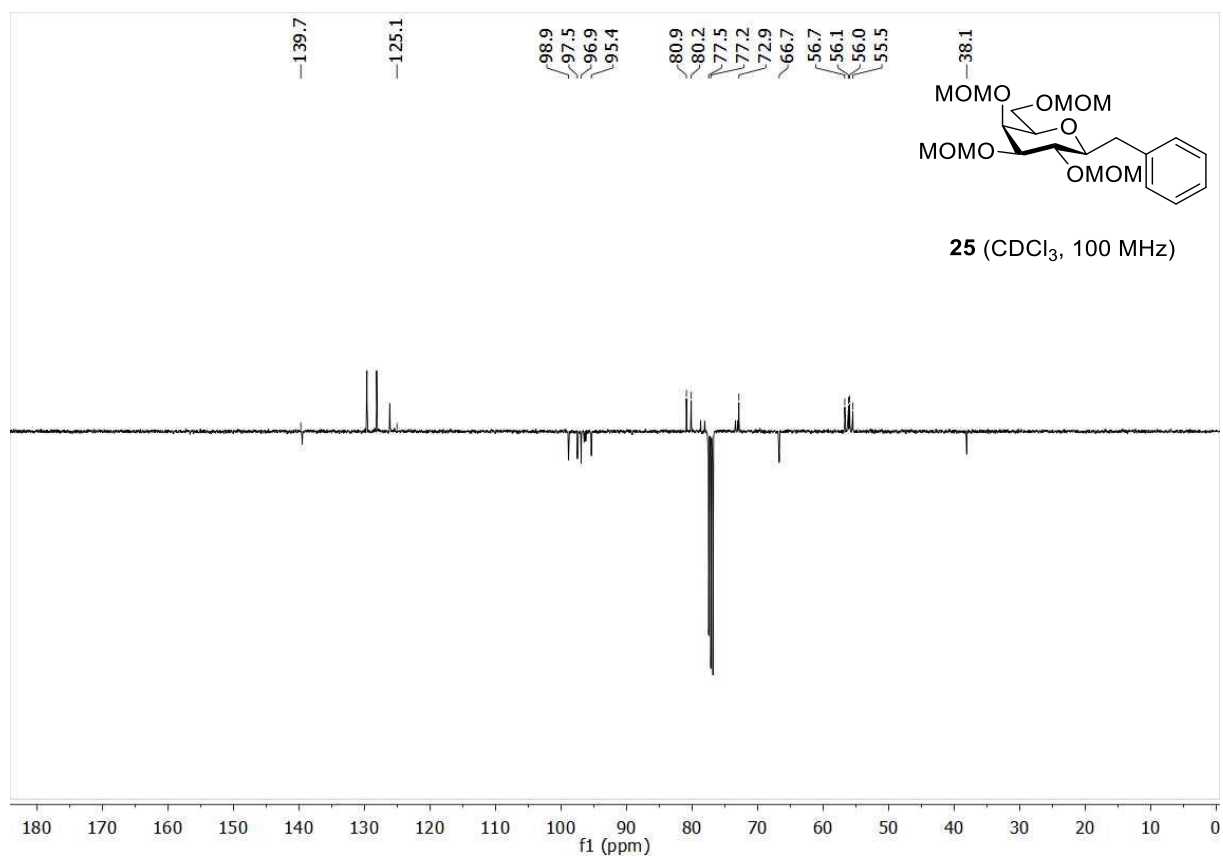

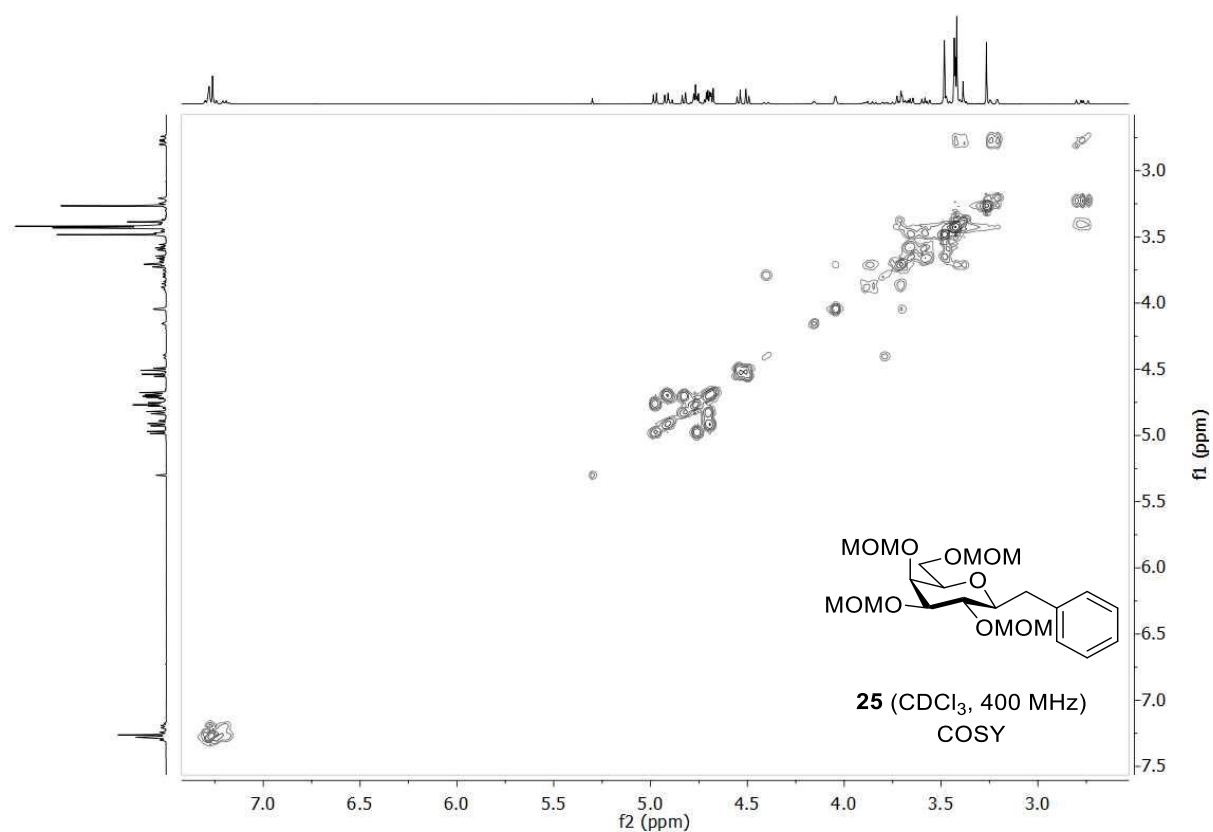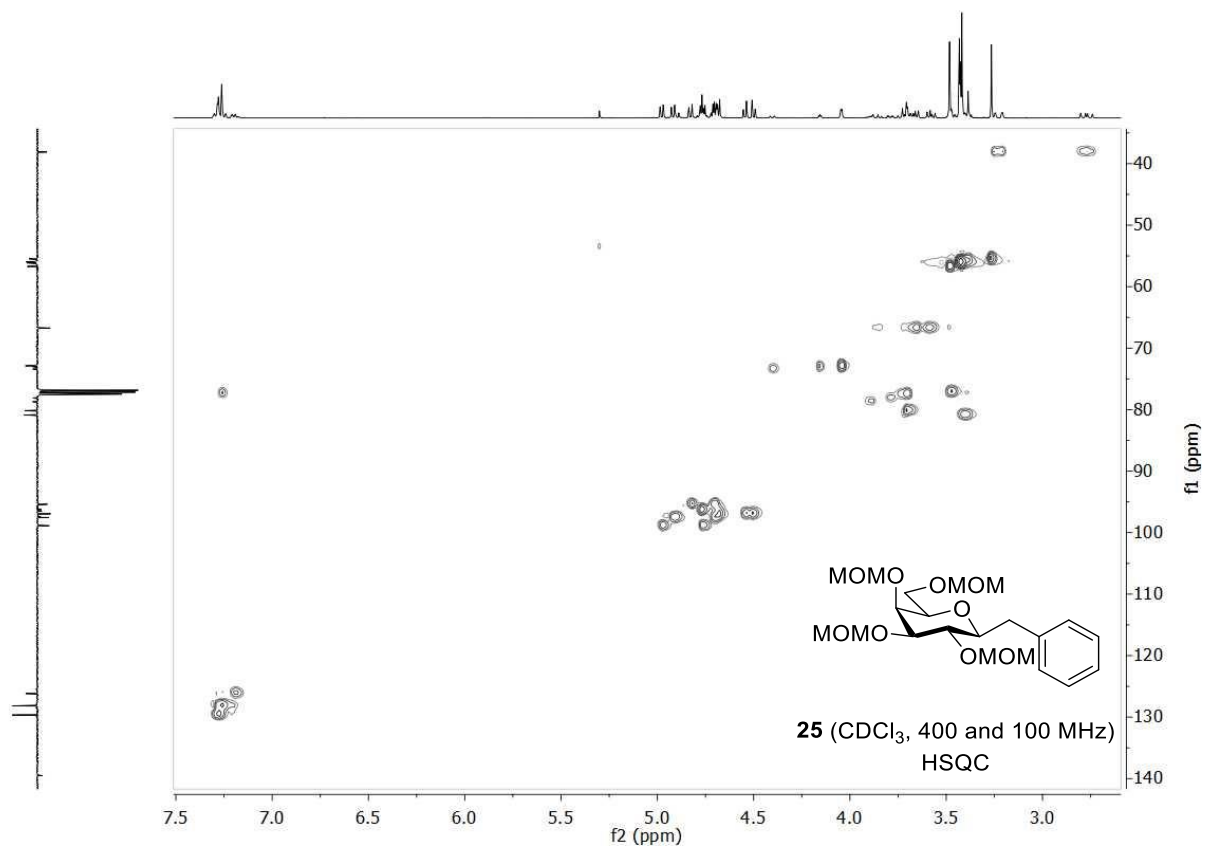

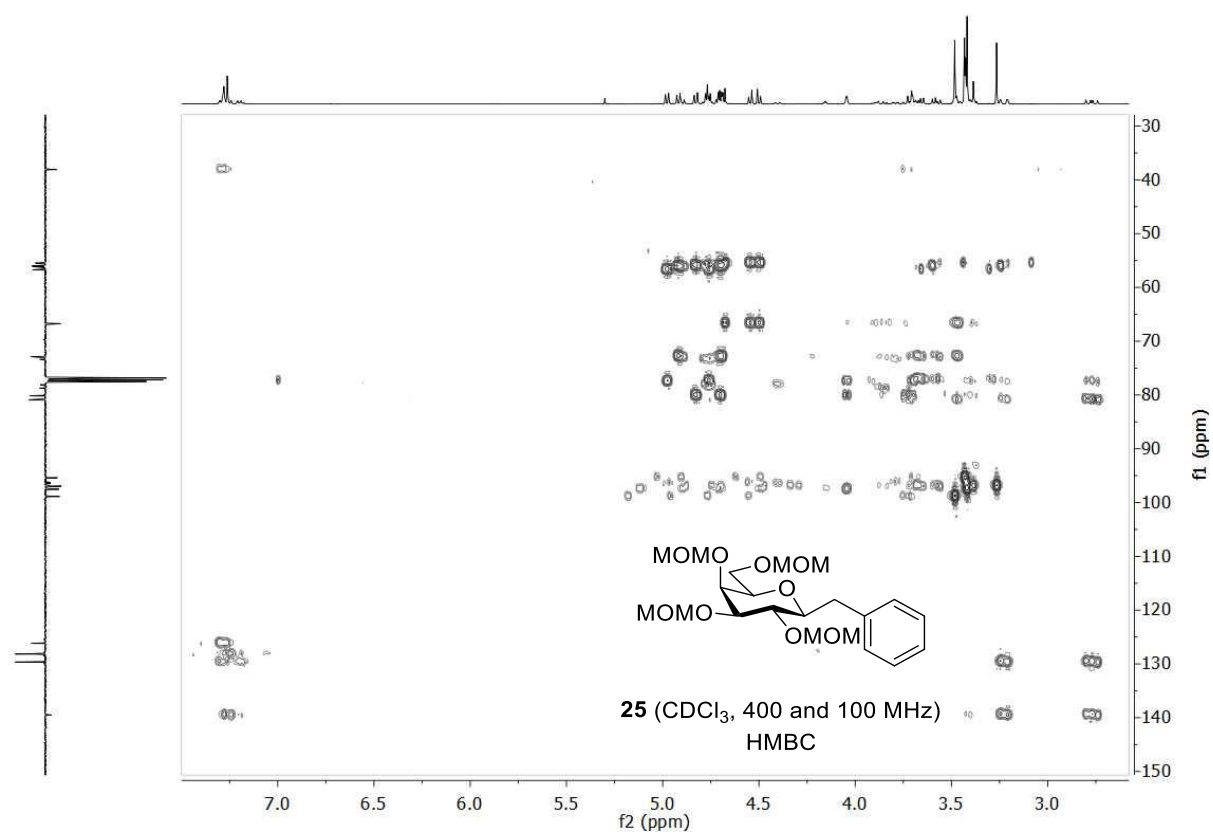

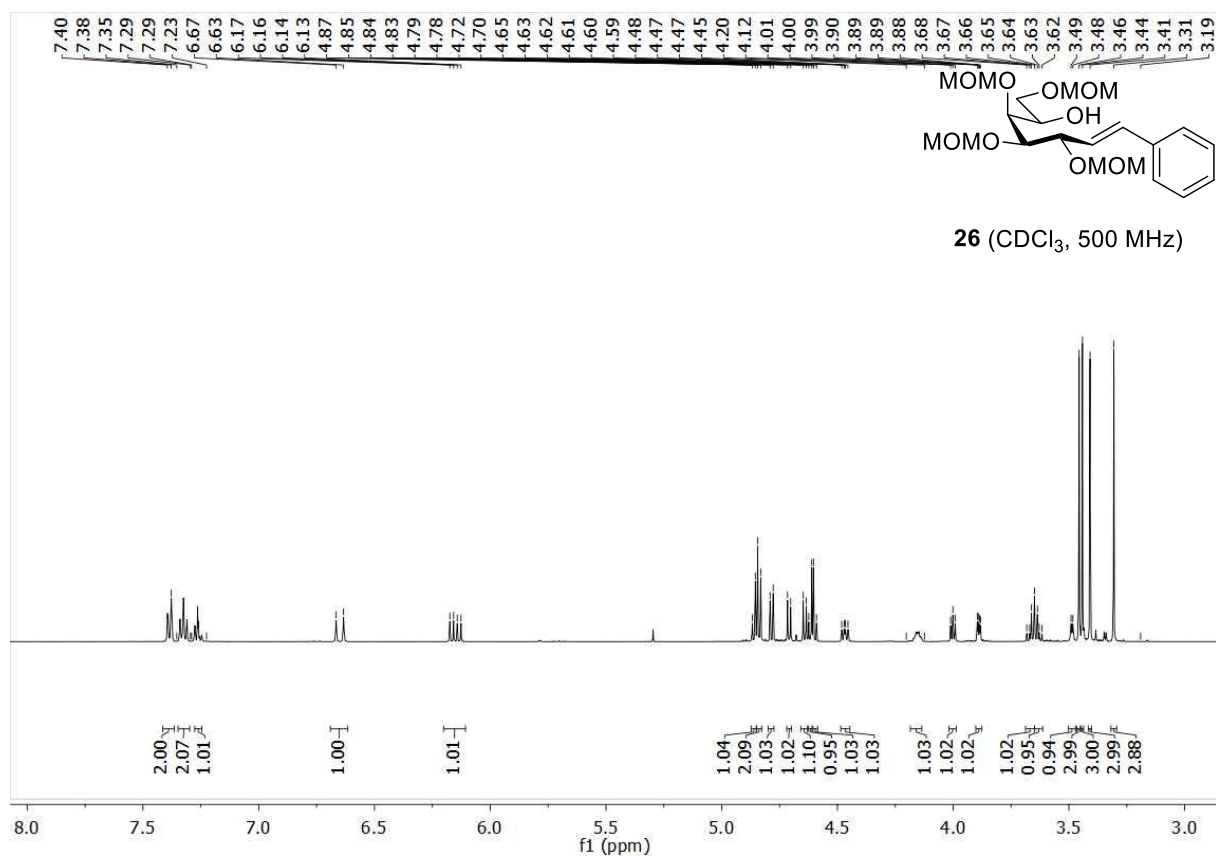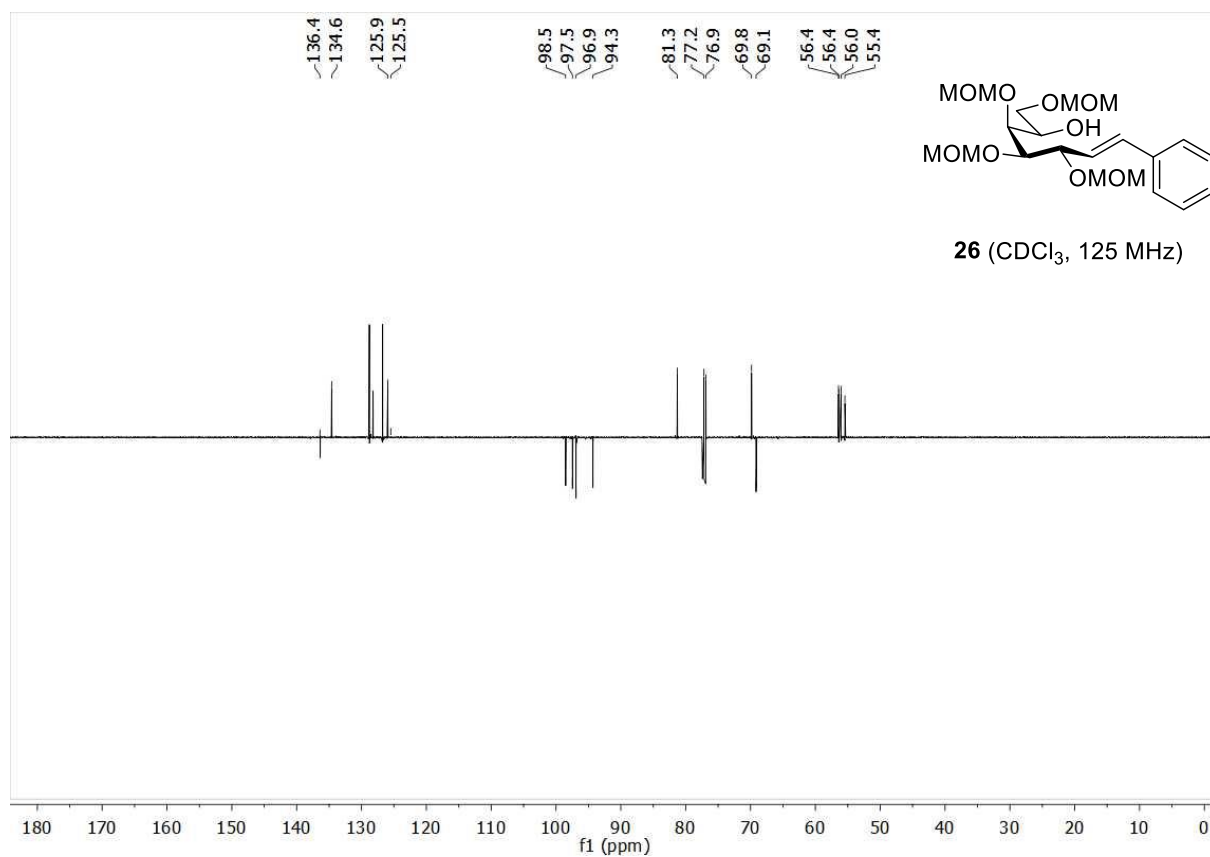

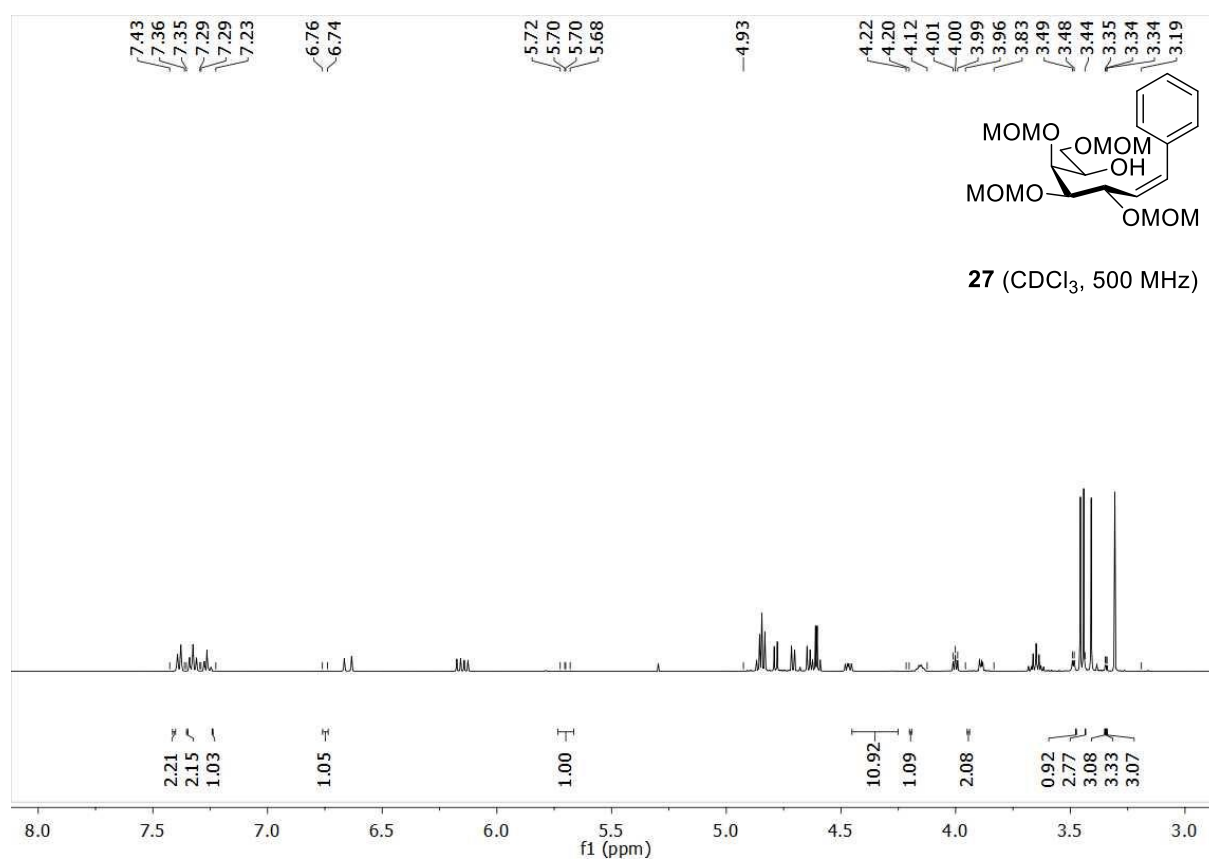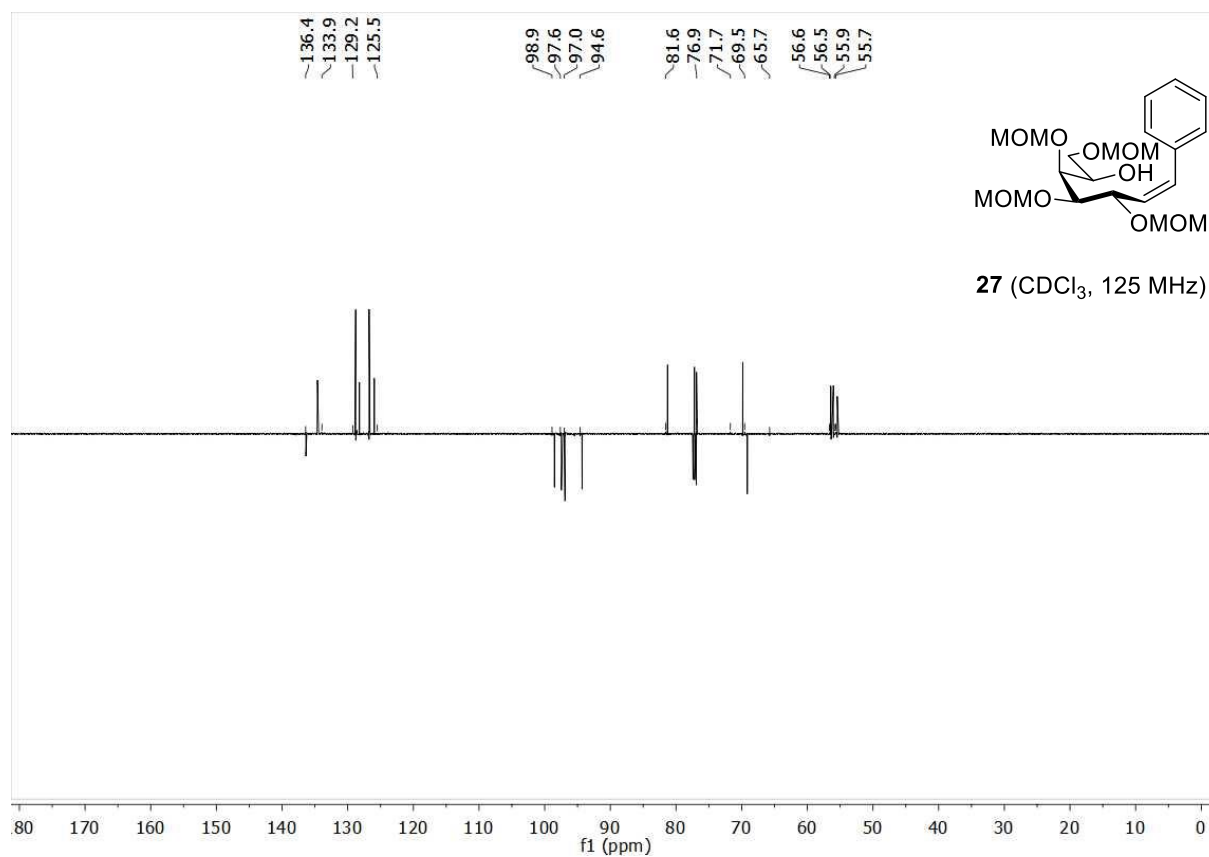

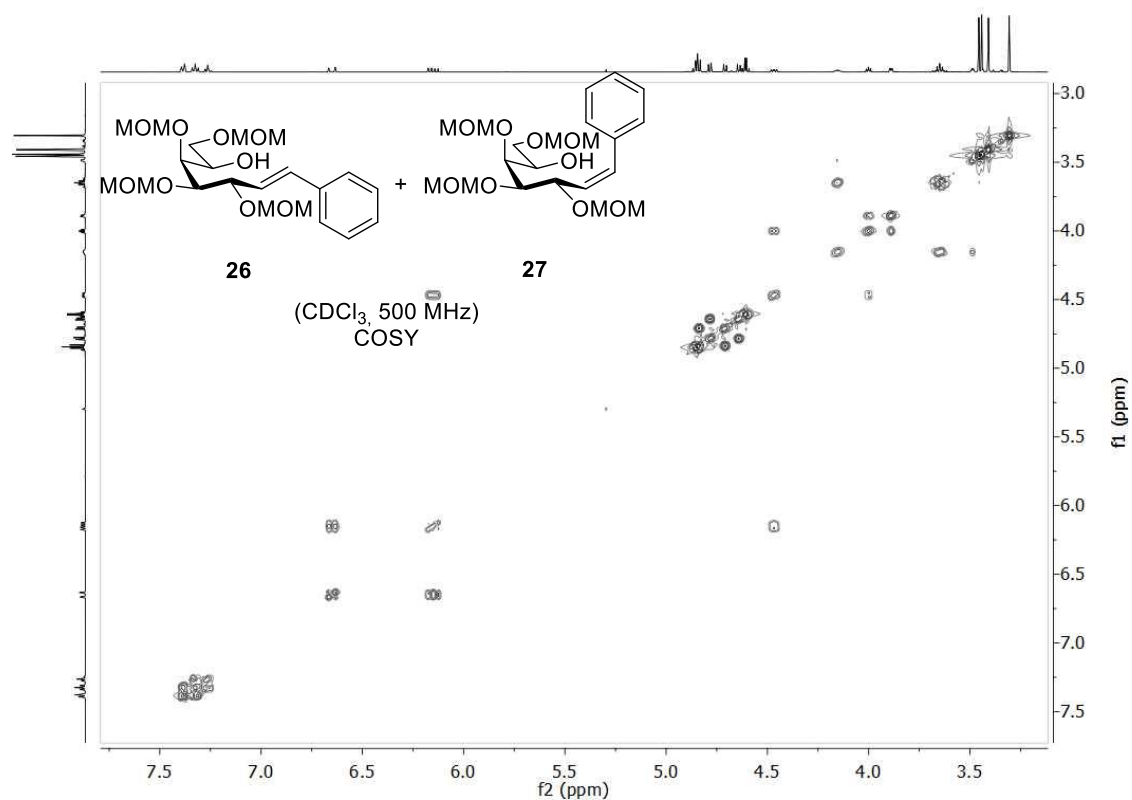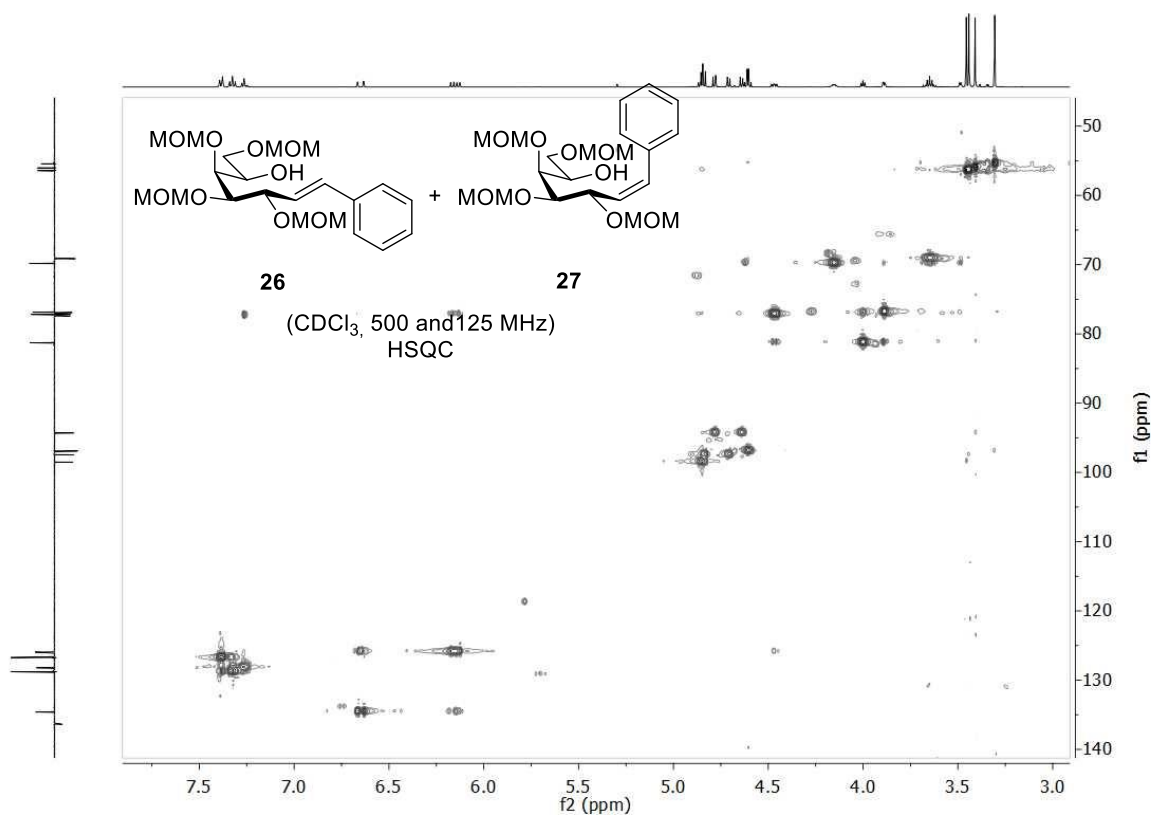

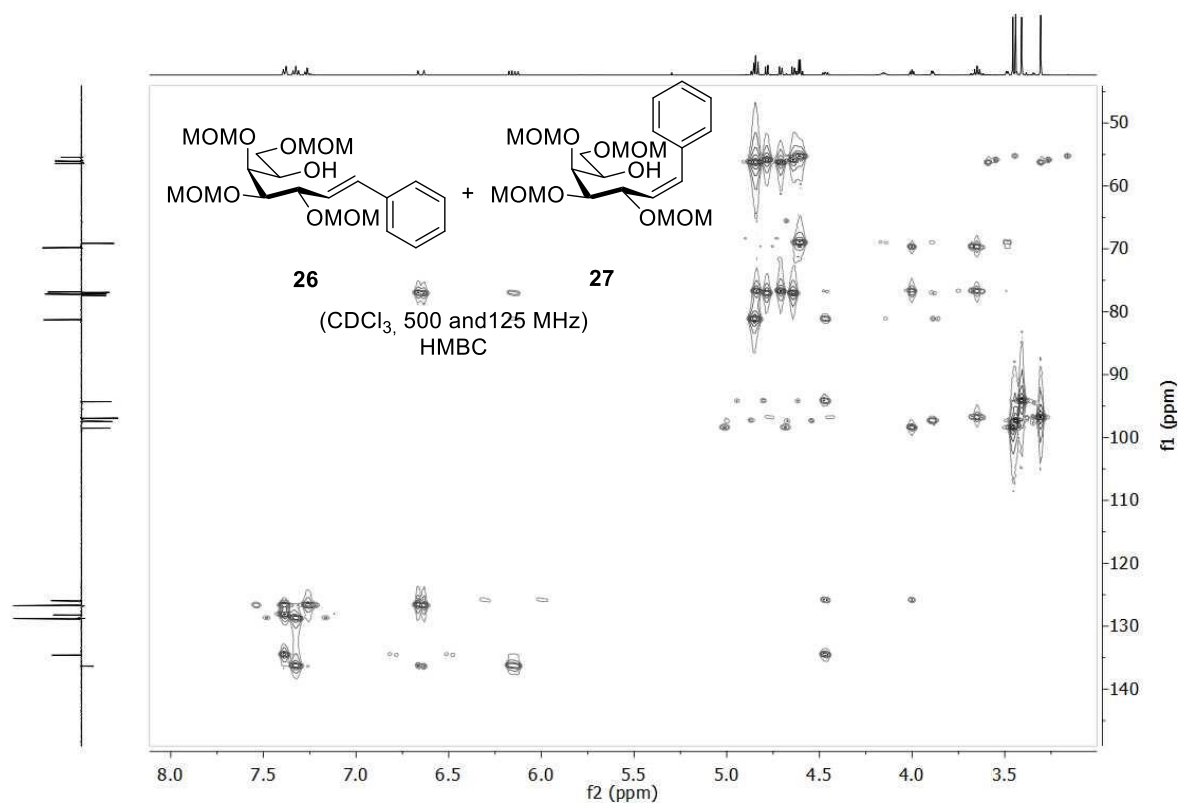

Supplement: Supplementary file 1 [file molecules-27-01795-s001.zip › molecules-1606654-supplementary.pdf]
